# Coulombic control of charge transfer in radicals with quartet recycling luminescence

**DOI:** 10.1038/s41467-026-72487-5

**Published:** 2026-05-22

**Authors:** Lujo Matasovic, Petri Murto, Shilong Yu, Wenzhao Wang, James D. Green, Giacomo Londi, Weixuan Zeng, Laura Brown, William K. Myers, Lars van Turnhout, Konstantina Armadorou, Avik Bhanja, Sergiu Petrusca, David Beljonne, Yoann Olivier, Feng Li, Hugo Bronstein, Timothy J. H. Hele, Richard H. Friend, Sebastian Gorgon

**Affiliations:** 1 Cavendish Laboratory, University of Cambridge, Cambridge, UK; 2 Yusuf Hamied Department of Chemistry, University of Cambridge, Cambridge, UK; 3 Department of Chemistry and Materials Science, Aalto University, Espoo, Finland; 4 State Key Laboratory of Supramolecular Structure and Materials, Jilin University, Changchun, PR China; 5 Department of Chemistry, University College London, London, UK; 6 Department of Chemistry and Industrial Chemistry, University of Pisa, Pisa, Italy; 7 Centre for Advanced ESR, Department of Chemistry, University of Oxford, Oxford, UK; 8 Department of Chemical Engineering & Biotechnology, University of Cambridge, Cambridge, UK; 9 Laboratory for Chemistry of Novel Materials, University of Mons, Mons, Belgium; 10 Laboratory for Computational Modelling of Functional Materials, University of Namur, Namur, Belgium; 11 Department of Chemistry, The University of Tokyo, Tokyo, Japan

**Keywords:** Photochemistry, Optical spectroscopy, Optical materials

## Abstract

Excitons in organic materials are emerging as an attractive platform for tunable quantum technologies. Structures with near-degenerate doublet and triplet excitations in linked trityl radical, acene and carbazole units can host quartet states. These high spin states can be coherently manipulated, and later decay radiatively via the radical doublet transition. However, this requires controlling the deexcitation pathways of all metastable states. Here we establish design rules for efficient quartet generation and recycling to luminescence, using different connection arrangements of the molecular units. We discover that electronic coupling strength between these units dictates quartet formation and delayed emission yields, particularly through a Coulombically tuned acene-radical charge transfer state. This state acts as a source of non-radiative decay when acene-radical separation is small, but facilitates reversible doublet-quartet interconversion when acene-radical separation is large. Using these rules we report a material with 55% luminescence yield, where 94% of emitting excitons are recycled from the quartet with a 1.0 *μ*s lifetime. This reveals the central role of molecular topology in luminescent quantum materials.

## Abstract (web summary)

High-spin excitons can be coherently controlled, but rarely access radiative decay pathways. Here, organic radical-based materials where thermally activated quartet state recycling leads to efficient luminescence on microsecond times are reported.

## Introduction

Robust spin-optical interfaces are crucial to harnessing the quantum resources of materials, as light provides a facile way to interact with a system.^1^ Most interfaces to date utilise defects in inorganic materials, such as diamond.^2^ However, their excellent optical properties may be degraded through nanofabrication required for their miniaturisation.^3,4^ Control of interactions between multiple defects and their deterministic formation also remain challenging.^5^ An emerging alternative involves using molecules, offering vast tunability via synthetic modulation.^6,7^ Organic materials in particular support multi-spin interactions due to electron delocalisation via *π*-pathways.^8^ Incorporating stable radicals is attractive since these can contribute to states active in Electron Spin Resonance (ESR), both in the ground (doublet, *S* = 1/2) and excited (quartet, *S* = 3/2) state.^9^ Quartets rely on engineering exchange coupling between a pair of triplet spins and a third spin on the pendant radical.^10^ Critically, such compounds have thus far been non-luminescent when constructed from fully-organic units,^11,12^ limiting their potential.^13^

Luminescent organic radicals are a rapidly developing class of materials with a doublet emission channel offering unique spin-optical opportunities.^14^ While many molecular design strategies are being pursued,^15–18^ donor-substituted tris(2,4,6-trichlorophenyl)-methyl (TTM) radicals are currently the most performant platform, and have been utilised in organic light-emitting diodes with record efficiencies.^19–21^ Their high emission quantum yields and stability have been attributed to a donor-to-TTM charge transfer (CT) lowest excited state.^22^ We recently utilised a derivative of the TTM radical to engineer a luminescent pathway following quartet generation.^23^ Delayed luminescence was enabled by minimising the excited state energy gaps, through a resonance between the doublet and triplet excited states of the dyad units. As only a singular design of TTM radical-chromophore dyads with efficient delayed luminescence is currently known, the structure-property relationship remains unclear.

**Fig. 1:**
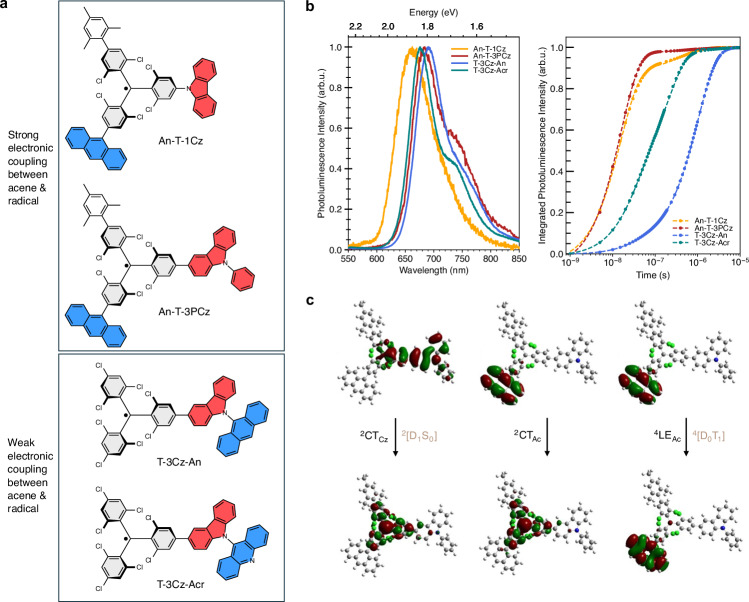
Effects of topology in radical-acene dyads. **a** Chemical structures. In An-T-1Cz and An-T-3PCz, the anthracene and carbazole are both coupled directly to the radical, providing strong radical-acene electronic coupling. The radical is decorated with mesityl groups, providing stabilisation via extra steric protection without affecting the photophysics.^67^ In T-3Cz-An and T-3Cz-Acr, the acene is linked to the radical via carbazole, providing weak radical-acene electronic coupling. **b** Normalised steady-state emission spectra (left) and integrated transient PL curves (right) performed in 0.1 mM toluene solutions at room temperature under near band gap excitation. Experimental data shown in dots, biexponential fits shown in dashed lines. **c** Hole-particle natural transition orbitals (NTOs) in An-T-3PCz: ^2^CT_Cz_ is the carbazole to radical doublet CT state, ^2^CT_Ac_ is the acene to radical doublet CT state and ^2,4^LE_Ac_ are the doublet/quartet locally excited states on the acene unit.

In this work, we establish general strategies for controlling quartet state generation in luminescent radicals. We synthesise new compounds with three distinct molecular building blocks: a TTM core (T), a carbazole, and an acene unit with a triplet energetically close to the emissive state (Fig. 1a). The acene is connected directly to the radical core (An-T-1Cz and An-T-3PCz), or separated from it by a carbazole bridge (T-3Cz-An and T-3Cz-Acr). Acridine is chosen as it has a higher triplet energy (1.96 eV) compared to anthracene (1.82 eV).^24^ The synthetic details are provided in Supplementary Note 1. Cyclic voltammetry shows that the radical site supports reversible oxidation and reduction in all compounds (Supplementary Fig. 10). We identify four key excited states (Fig. 1c): a bright carbazole-to-TTM charge-transfer state (^2^CT_Cz_); two local excitations on the triplet-bearing unit (^2^LE_Ac_ and ^4^LE_Ac_), and an acene-to-TTM charge-transfer state (^2^CT_Ac_). Direct radical-acene connectivity yields substantially larger Coulomb attraction between the radical electron and the acene-localised hole (strong electronic coupling), compared to the carbazole-bridged analogues (weak electronic coupling). The 0-0 energies of both CT states are within 1.9 ± 0.2 eV (Supplementary Table 2), and are sensitive to the molecular topology and environment polarity.^25^ The closely energetically spaced pair of LE states is pinned to the level of its constituent acene triplet.^11^ We show how the energetic ordering of these four states, set by the magnitude of the radical-acene electronic coupling, controls the efficiency of quartet formation and subsequent luminescence. Throughout this work, we also use the anthracene-free T-3PCz and the newly synthesised carbazole-free T-An as control molecules (Supplementary Fig. 11).^26^

## Results

### Optical properties

Experiments were performed at room temperature in 100 μM toluene solutions, unless stated otherwise. The compounds composed of all three fragments are luminescent and show steady-state spectra typical of donor-substituted TTM radicals (Fig. 1b and Supplementary Fig. 11).^15^ Absorption data in a series of solvents (Supplementary Figs. 12–14) show that their lowest transition has CT character (Supplementary Fig. 15). However, materials with strong radical-acene electronic coupling have photoluminescence quantum yields (*ϕ*_PL_) an order of magnitude lower than materials with weak radical-acene electronic coupling (Table 1). This stark difference cannot be explained by the existing understanding of luminescent high-spin radicals,^23^ as these four compounds all fulfil the doublet-triplet energetic resonance condition. Photothermal deflection spectroscopy (PDS) allows us to investigate the energetic ordering near the band edge (Supplementary Fig. 16). In T-An we resolve a weak band near 1.7 eV, indicating that the ^2^CT_Ac_ state is present below ^2^CT_Cz_ in compounds with direct acene-radical linkage.

**Table 1:** Photophysical characterisation

Compound	*λ*_PL_ [nm]	$\phi$_PL_ [%]	*τ*_1_ [ns]	$\phi$_1_ [%]	*τ*_2_ [ns]	$\phi$_2_ [%]	Δ*E*[meV]
An-T-1Cz	662	8	15.6	7.1	437	0.9	23 ± 4
An-T-3PCz	684	3	15.8	2.9	729	0.06	21 ± 6
T-3Cz-An	690	55	31.6	3.3	1030	51.7	15 ± 4
T-3Cz-Acr	676	50	31.8	18.0	216	32.0	11 ± 4

Photoluminescence peak wavelength (*λ*_PL_), quantum efficiency (*ϕ*_PL_), prompt (*τ*_1_) and delayed (*τ*_2_) emission lifetimes with their contributions to the quantum yield (*ϕ*_1_ + *ϕ*_2_ = *ϕ*_PL_) measured in 0.1 mM toluene solutions at 292 K. Effective activation energies (Δ*E*) were determined from temperature-dependent transient PL measurements in 5 wt% PMMA films.

The photoluminescence (PL) provides a powerful probe of excited-state dynamics. All emissive compounds exhibit time-gated spectra matching the steady-state lineshapes in solution (Supplementary Fig. 17). They show bi-exponential decay kinetics (Fig. 1b and Supplementary Fig. 18), but the delayed emission rates span over an order of magnitude (Supplementary Table 3). We calculated quantum yields of the two emission components, *ϕ*_1_ and *ϕ*_2_, taken as the product of *ϕ*_PL_, and the prompt or delayed fitted weight. Delayed emission dominates only in compounds with weak radical-acene electronic coupling. As this follows quartet-doublet reverse intersystem crossing (RISC), the dominance of this channel over prompt emission is desired to prolong access to the quartet exciton. The quartet is depleted 5 times more rapidly in T-3Cz-Acr than T-3Cz-An, making the latter the champion compound, with up to threefold improvement on the previously reported *ϕ*_2_ (18% in T-1Cz-An).^23^

To explore environment effects, we study T-3Cz-An in more polar solvents (Supplementary Fig. 19), and observe that emission is weaker and redshifted in tetrahydrofuran compared to toluene. The prompt emission also dominates over the delayed emission, indicating that the state reordering in a polar environment hinders quartet recycling. While T-3PCz also shows a redshift in polar solvents on ns timescales (Supplementary Fig. 20), Kerr-gated transient photoluminescence reveals important differences between the compounds (Supplementary Fig. 21). In T-3Cz-An, a redshifted luminescence band is seen on ps timescales in dichloromethane (DCM). This band is absent in toluene or in reference compound T-3PCz, suggesting it originates from a rapidly populated, solvent-stabilised ^2^CT_Ac_ state.

We use time-resolved PL measurements on films diluted in poly(methyl methacrylate) (PMMA) to study the solid-state performance. The effective lifetime extends to around 1.8 μs and 2.3 μs for films of T-3Cz-An and T-3Cz-Acr, respectively (Supplementary Table 4). In contrast, it shortens in films of An-T-3PCz, indicating the promotion of deactivation channels in a more rigid environment.^27^ Comparison of 0.5% and 5% film data confirms absence of major aggregation effects (Supplementary Fig. 22). We also detect faint luminescence at timescales of tens of μs in 0.5% films (Supplementary Fig. 23). Due to its energetics, we tentatively assign it to direct emission from the ^2^CT_Ac_ state. We study the temperature-dependence of the delayed emission in films at 5% loading (Supplementary Figs. 24–28). When the molecules are immobilised in a rigid medium, we observe small time-dependent hypsochromic shifts, which we ascribe to conformational disorder modulating the rates of RISC and non-radiative decay across the energy range of the ensemble.^28,29^. Using an Arrhenius-type model, we calculate the activation energies of the main delayed emission channel (Supplementary Fig. 29 and Supplementary Table 5), which are below *k*_B_*T* at room temperature.

**Fig. 2:**
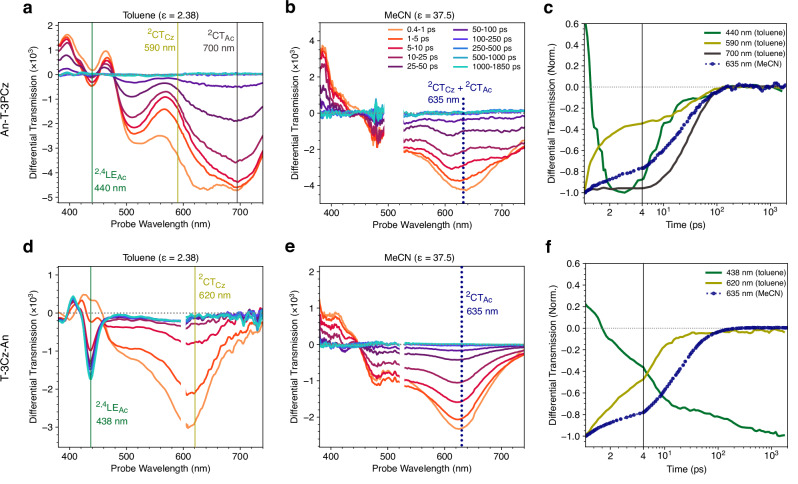
Dielectric control of non-radiative decay. Transient absorption spectra and kinetics in dilute toluene and acetonitrile (MeCN) solutions of An-T-3PCz (**a**–**c**) T-3Cz-An (**d**–**f**). The colour scale of the time slices is identical across all spectral panels. Extracted kinetics in toluene reveal sub-10 ps wavefunction localisation onto the anthracene unit in both compounds. *ϵ* is the solvent dielectric constant.

We use Transient Absorption (TA) spectroscopy to track the excited state dynamics of our materials across ps-μs times (Fig. 2 and Supplementary Fig. 30). In dilute toluene solutions, the prompt TA spectra contain a ground-state bleach (GSB) in the UV region, a D_1_ photoinduced absorption (PIA) around 600 nm, and a broad IR PIA corresponding to the CT states. In An-T-1Cz and An-T-3PCz, we also resolve a band near 700 nm, which we assign to ^2^CT_Ac_.^30^ A growing PIA at 430 nm is due to the acene triplet, arising from both ^2^LE_Ac_ and ^4^LE_Ac_ populations.^31^ For An-T-1Cz and An-T-3PCz, this band is convolved with the GSB.

Strong radical-acene electronic coupling facilitates rapid interconversion between ^2^CT_Ac_ and ^2^LE_Ac_ states (Supplementary Fig. 31). T-An, An-T-1Cz and An-T-3PCz display similar decay kinetics, with sub-50 ps decay lifetimes (Supplementary Table 6). For T-An, the dynamics follow a biexponential decay, with a prompt component of 3.5–5.5 ps and a delayed component of approximately 34 ps. An-T-1Cz and An-T-3PCz exhibit comparable kinetics across all bands, with the exception of PIAs associated with the ^2^CT_Cz_ state, where an additional sub-picosecond component is observed. We assign this ultrafast process to internal conversion from ^2^CT_Cz_ to ^2^CT_Ac_. In contrast, for a 5% An-T-3PCz in PMMA film, transient signals persist beyond 8 ns (Supplementary Fig. 32), indicating that non-radiative decay can be modulated by restricting molecular conformations in the solid state. For materials with weak radical-acene coupling, the decay of the initial ^2^CT_Cz_ PIA matches the rise of the ^2^LE_Ac_ signal, yielding ^2^LE_Ac_ formation lifetimes of *τ* = 3.8 ± 0.1 ps for T-3Cz-An and *τ* = 4.6 ± 0.1 ps for T-3Cz-Acr. However, we do not observe an accumulation of population in the ^2^CT_Ac_ band. In T-3Cz-An, the ^2,4^LE_Ac_ PIA subsequently decays with a 1.55 *μ*s lifetime in toluene (Supplementary Figs. 33–34), matching the delayed emission dynamics.

Finally, we turn to solvatochromic TA studies (Supplementary Figs. 35–37 and Supplementary Table 7). The ^2^CT_Ac_ state becomes the lowest excited state in An-T-3PCz in acetonitrile (MeCN), with a spectral profile matching that of T-An (Supplementary Fig. 38), allowing us to complete the species assignment. For T-3Cz-An, the ^2,4^LE_Ac_ PIA is suppressed in dichloromethane, and in MeCN it is no longer observed, showing that the energetic ordering of the states is inverted in a high-polarity medium, becoming the same as in compounds with strong electronic coupling. The broad PIA band at 625 nm resembles that of T-3PCz in MeCN with lifetime matching An-T-3PCz. This reveals that a high-polarity environment exerts a dual effect. It quenches ^2^CT_Cz_ emission, as seen in other TTM-donor radicals.^15^ Additionally, in high-spin radicals, it stabilises the CT states below the ^2,4^LE_Ac_ manifold, which suppresses quartet generation.

### Magnetic properties

**Fig. 3:**
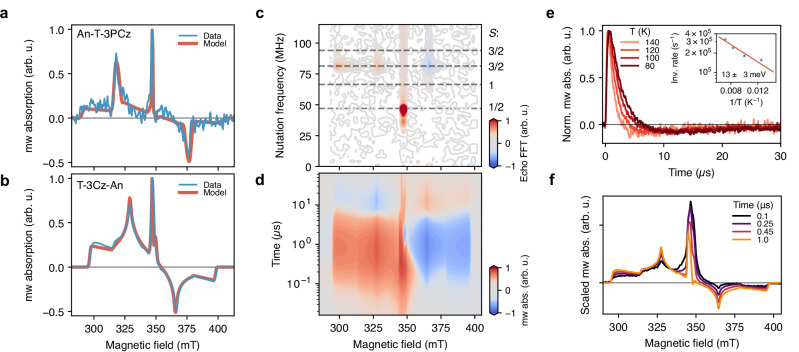
Quartet excited-state electron spin resonance. Performed on 100 μM toluene frozen solutions at 80 K at X-band. Prompt (0.5–1.5 μs after pump) transient ESR on **a** An-T-3PCz, and **b** T-3Cz-An. mw is microwave. **c** Transient nutation map confirming spin multiplicity of recorded transitions in T-3Cz-An, indicating the positions calculated based on the dark nutation signal and the state spin quantum number *S*. FFT is Fast Fourier Transform. **d** 2D trESR map in T-3Cz-An. **e** Temperature dependence of inversion rate of T-3Cz-An polarisation monitored at 328 mT. Inset shows Arrhenius analysis. **f** Rise time trESR spectra in T-3Cz-An. A broad absorptive feature is visible at 100 ns, which is buried below the rising quartet polarisation.

Transient ESR (trESR) performed at low microwave powers (Supplementary Fig. 39) at X-band reveals the ^4^LE_Ac_ states in compounds containing all three fragments (Fig. 3 and Supplementary Fig. 40). The state multiplicity is validated by resolving broad Δ*m*_*S*_ = 2 and narrow Δ*m*_*S*_ = 3 half-field signals in T-3Cz-An (Supplementary Fig. 41). The lineshapes of the prompt (averaged 0.5–1.5 μs after pump) full-field spectra are typical of an acene covalently tethered to a radical, within the strong triplet-radical exchange coupling regime.^23,32,33^ The dipolar coupling constants reveal the shape of the quartet spin density. We observe more axial symmetry and larger deviations from the acene triplet ZFS values in compounds with direct radical-acene coupling,^34^ suggesting that this linkage induces a saddle-shaped distortion of the anthracene. The polarisation patterns extracted from simulations (Supplementary Fig. 42) reveal that the m_*s*_ = ±1/2 zero-field quartet sublevels are favoured during ISC,^35^ due to the dominant z-component of spin orbit coupling (SOC) within the anthracene triplet (Supplementary Table 8).^36^ This suggests that the main quartet formation pathway in these four materials is radical-enhanced spin-orbit borrowing.^37^ We did not detect any excited or ground state polarised signals in the trESR of T-An, indicating that non-radiative relaxation outcompetes ISC, and quartet states are not formed.

Additionally, in T-3Cz-An we observe a homogeneously broadened absorptive feature centred at *g* = 2.004 with width Δ*ω* ≈ 5.5 mT (Fig. 3d). This feature narrows during the rise of the quartet signal (Fig. 3f). This may be associated with the overpopulation of the *m*_*s*_ = −1/2 sublevel of the accessible ^2^CT_Ac_ state.^38^ At later times, the polarisation of the broad signal flips, as previously observed in quartet-bearing molecules.^39^ T-3Cz-Acr shows the same behaviour (Supplementary Fig. 43). This indicates that decay rates of the quartet sublevels are asymmetric, which in our systems is due to RISC favouring the *m*_*s*_ = ±1/2 sublevels. The inversion occurs at earlier times at higher temperatures (Fig. 3e), reflecting the activated delayed emission dynamics. However, contributions from other temperature-dependencies (primarily spin-lattice relaxation times) hinder its quantitative analysis.

The molecules with longer quartet lifetimes are suitable for pulsed ESR spectroscopy. The phase memory time of the T-3Cz-An ^4^LE_Ac_ state at 80 K in frozen dilute protonated toluene is *T*_*m*_ = 0.90 μs (Supplementary Fig. 44). To confirm spin multiplicity across the whole spectrum, we apply a light-induced nutation pulse sequence as a function of field.^40^ This verifies that there are no uncoupled triplet excitons in molecular ensembles of T-3Cz-An (Fig. 3c) and T-3Cz-Acr (Supplementary Fig. 45).

### Theoretical insights

To rationalise the results above, we develop the energetic criteria for radical-acene dyads by combining electronic structure algebra for excited states of radicals with perturbation theory.^19,22,41–44^ The full derivations can be found in Supplementary Note 2. For both ISC and RISC to occur efficiently, ^4^LE_Ac_ must lie below ^2^CT_Cz_ by roughly the thermal energy. At zeroth order, if the *D*_1_ energy of the radical is similar to the *T*_1_ energy of the acene, the bright ^2^CT_Cz_ state will be of similar energy to the quartet state ^4^LE_Ac_ in the dyad. Higher levels of theory stabilise the quartet energy relative to the isolated triplet energy by approximately $\frac{1}{2}({K}_{A1,R0}+{K}_{R0,A{1}^{{\prime} }})$, i.e. half the exchange energy of an electron in the SOMO with one in the acene HOMO, plus half the exchange energy of an electron in the SOMO with one in the acene LUMO.^45^ The RISC is therefore efficient when the sum of this exchange stabilisation and the *D*_1_-*T*_1_ energy gap is comparable to *k*_B_*T*.

^2^CT_Ac_ is stabilised by the Coulomb stabilisation *J*_*A*1,*R*0_ between an electron in the radical SOMO and the hole in the acene HOMO. To avoid non-radiative decay via this state, we wish to minimise *J*_*A*1,*R*0_ by ensuring that the radical SOMO and acene HOMO are spatially separated. We find *J*_*A*1,*R*0_ values are approximately 40% lower in T-3Cz-An and T-3Cz-Acr than in An-T-3PCz and An-T-1Cz (Table 2), further indicating that modulating the Coulombic stabilisation of ^2^CT_Ac_ by altering the proximity of TTM and acene moieties modulates the efficiency of delayed emission.

**Table 2:** Interaction strength quantification

Compound	Coulomb stabilisation [eV]	Electronic coupling [meV]
An-T-1Cz	1.17	36.3
An-T-3PCz	1.17	29.6
T-3Cz-An	0.75	6.0
T-3Cz-Acr	0.67	12.7

Coulomb stabilisation and radical-acene electronic coupling terms for the investigated dyads were calculated using the semiempirical model described in Supplementary Note 2.

The electronic coupling between ^2^LE_Ac_ and ^2^ CT_Ac_ states is given by the one-electron term $\langle {\scriptstyle{{2}}\atop} \!{{{{\rm{C}}}}{{{\rm{T}}}}}_{{{{\rm{A}}}}{{{\rm{c}}}}}|{\hat{H}}^{(1)}|{\scriptstyle{{2}}\atop} \!{{{{\rm{L}}}}{{{\rm{E}}}}}_{{{{\rm{A}}}}{{{\rm{c}}}}}\rangle=\frac{3}{\sqrt{6}}{F}_{R0,A{1}^{{\prime} }}$, where *R*0 and $A1^{\prime}$ denote the radical SOMO and acene LUMO, respectively. We find that this radical-acene electronic coupling is between 2 and 6 times larger in An-T-1Cz and An-T-3PCz than in T-3Cz-An and T-3Cz-Acr.^43,45–50^ Further calculation results are shown in Supplementary Tables 9 and 10.

### Quantum-chemical calculations

We carried out excited-state calculations both in toluene and 2-methyltetrahydrofuran (MeTHF) by means of time-dependent density functional theory (TDDFT) calculations, as summarised for T-3Cz-An and An-T-3PCz in Table 3. For each system, we identify key excited states using natural transition orbitals (NTOs, Fig. 1c and Supplementary Figs. 47–52). ^2,4^LE_Ac_ and ^2^CT_Ac_ show a vanishing oscillator strength, in contrast to the brighter ^2^CT_Cz_ (Supplementary Tables 12–16). The lowest-lying CT state in compounds with weak radical-acene electronic coupling is the bright ^2^CT_Cz_. The trend is reversed in molecules with strong radical-acene electronic coupling, where the bright ^2^CT_Cz_ lies above the dark ^2^CT_Ac_. The low-lying ^2^CT_Ac_ competes with the generation of quartet states and delayed luminescence.

**Table 3:** Excited-state calculations

Toluene					MeTHF				
*E* [eV]	f	*R*_he_[*Å*]	*ϕ*_*s*_	State	*E* [eV]	f	*R*_he_[*Å*]	*ϕ*_*s*_	State
An-T-3PCz									
2.06	0.000	0.05	0.89	^2,4^LE_Ac_	2.05	0.001	0.05	0.83	^2,4^LE_Ac_
2.28	0.007	6.74	0.21	^2^CT_Ac_	2.11	0.004	5.90	0.13	^2^CT_Ac_
2.41	0.121	5.58	0.50	^2^CT_Cz_	2.26	0.115	6.78	0.42	^2^CT_Cz_
T-3Cz-An									
2.06	0.000	0.06	0.89	^2,4^LE_Ac_	2.05	0.000	0.06	0.89	^2,4^LE_Ac_
2.34	0.130	5.78	0.48	^2^CT_Cz_	2.14	0.113	7.18	0.39	^2^CT_Cz_
2.76	0.001	10.54	0.22	^2^CT_Ac_	2.38	0.007	11.38	0.14	^2^CT_Ac_

Vertical excitation energies (*E*), oscillator strengths (f), electron-hole separation distance (*R*_he_) and hole-electron density overlap (*ϕ*_*s*_) of low-lying transitions, calculated in toluene and MeTHF for representative molecules with strong (An-T-3PCz) and weak (T-3Cz-An) radical-acene electronic coupling.

In the higher polarity solvent, the energy differences between ^2^CT_Ac_ and ^2^CT_Cz_ states of T-3Cz-An are reduced. The stabilisation of ^2^CT_Ac_ is more significant than for the ^2^CT_Cz_ state, due to a larger electron-hole separation of ^2^CT_Ac_, as suggested by the calculated hole-electron separation distance *R*_*he*_ and by the hole-electron density overlap metric *ϕ*_*s*_. In contrast, the CT state energy difference in An-T-3PCz is not affected by solvent polarity, as its molecular topology dictates a comparable electron-hole separation in both CT states. For T-3Cz-Acr, the calculations show a large energy gap between the ^2^CT_Ac_ and ^2^CT_Cz_ states, making ^2^CT_Ac_ less accessible than in T-3Cz-An.

## Discussion

**Fig. 4:**
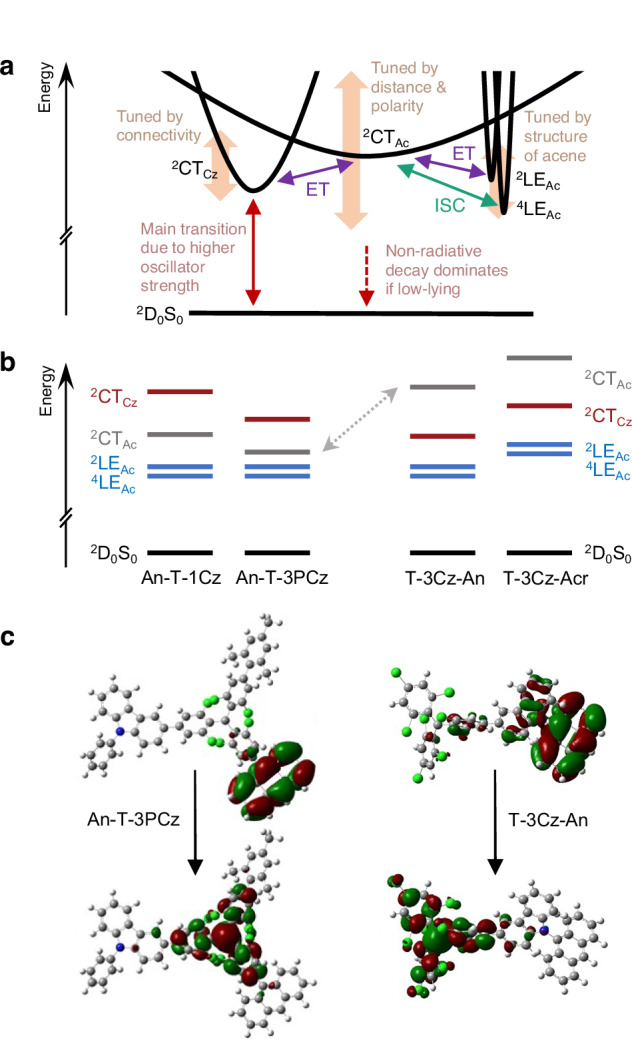
Excited state landscape. **a** State diagram for a luminescent radical with an accessible quartet, with principal energy flow pathways. Spin-conserving energy transfer (ET) as well as intersystem crossing (ISC) processes requiring a spin flip are indicated. The width of the parabolas indicates the energetic distribution of the corresponding states, and the length of the orange lines indicates the tuning range explored in the compounds presented here. **b** Relative energetic ordering of key states in a low dielectric constant environment. In An-T-3PCz and An-T-1Cz, the ^2^CT_Ac_ state lies below the strongly absorbing and emissive ^2^CT_Cz_, which limits emission yield. In T-3Cz-An and T-3Cz-Acr, the ^2^CT_Ac_ does not act as a trap and assists in reversibly linking the emissive ^2^CT_Cz_ and ^4^LE_Ac_. **c** Calculated ^2^CT_Ac_ state NTOs show that the extent of delocalisation is larger in T-3Cz-An than An-T-3PCz.

The contrast between the reported compounds shows that triplet-radical energy resonance alone does not guarantee efficient quartet formation in luminescent radicals. Covalent coupling of the chromophore and radical units inevitably introduces new states. In the case of radical-acene dyads, additional dark ^2^CT_Ac_ and ^2^LE_Ac_ states form. To minimise non-radiative decay via these states, tuning the energy difference between them and the bright ^2^CT_Cz_ state is crucial (Fig. 4a). When the energy gap between ^2^CT_Ac_ and ^2^LE_Ac_ is small, and the states are below the bright ^2^CT_Cz_, non-radiative decay to the ground state can be substantial, especially if the states mix.^26,51^

Direct conversion between ^2^LE_Ac_ and ^4^LE_Ac_ states is spin-forbidden to first order, but can be mediated by a state with a different spatial wavefunction.^52^ It has been previously demonstrated that a doublet charge transfer state between the dyad components can fulfil this role, provided it is energetically accessible.^23,37,53^ This condition is satisfied in T-3Cz-An, consistent with the high efficiency of delayed emission and the early-time signals in transient ESR measurements. However, as observed for An-T-3PCz and An-T-1Cz, when the ^2^CT_Ac_ state lies below both the quartet and the emissive ^2^CT_Cz_ states, non-radiative decay outcompetes ISC, suppressing quartet formation.

The TTM-acene electronic interactions play a dual role in these systems. Firstly, they dictate the energetic stabilisation of ^2^CT_Ac_ (Fig. 4b). The small electron-hole separation in An-T-1Cz and An-T-3PCz lowers its energy below that of ^2^CT_Cz_. In contrast, the molecular structure of carbazole-bridged molecules circumvents non-radiative decay through Coulombic interactions by maximising electron-hole separation of ^2^CT_Ac_. Secondly, they control the energy transfer rates between local acene and charge transfer excitations.^54,55^ Indirect attachment of acene to TTM via carbazole slows the relaxation from ^2^LE_Ac_ to the ground state, allowing ISC to kinetically outcompete non-radiative decay from the doublet states. Comparing An-T-3PCz and T-3Cz-An shows that molecular topology has greater consequences in modulating the electronic rather than exchange coupling landscape in these materials, since the strong exchange regime is reached already under a relatively small interaction. Due to the contribution of the TTM SOMO to the delocalised *π*-system,^13,56^ we expect that strong exchange coupling can be maintained in larger molecular structures.

More efficient RISC necessarily leads to a faster loss of spins on the ^4^LE_Ac_ sublevel manifold. This represents a trade-off between luminescence yield and spin coherence times in our designs, and calls for compounds with efficient but relatively slow delayed emission. T-3Cz-An is the first quartet recycling material with a delayed emission lifetime over 1 μs, and its coherence times are similar to those of TEMPO-based quartet molecules.^57^ The results presented here, together with the recently reported Radical Thermally Activated Delayed Fluorescence (TADF) molecules,^58^ show that quartet-derived emission can be achieved provided that any bright state is in energetic proximity to the high spin state.

We summarise the additionally identified design criteria for this class of materials. Firstly, the radical fragment should not be an alternant hydrocarbon. Secondly, ^2^CT_Ac_ should be high in energy, which can be achieved via spatial separation between the acene HOMO and radical SOMO, as well as a minimal energy gap between the SOMO and acene LUMO. Thirdly, strong exchange coupling can be obtained by minimising the energy gap between the SOMO and the acene frontier orbitals, as well as by linkage at sites that have large amplitudes of SOMO, HOMO and LUMO. We exemplify these rules with T-3Cz-An, where a high luminescence yield was achieved by maximally decoupling the acene from the radical.

This work presents a blueprint for compounds with microwave-addressable high-spin states which spontaneously access a delayed, radiative decay channel. The demonstrated chemical control of electronic and exchange coupling marks a crucial step in the development of molecular quantum technologies.

## Methods

### Steady-state spectroscopy

Ultraviolet/visible absorption spectra were measured with a Shimadzu UV-2550 spectrometer. Fluorescence spectra and photoluminescence quantum yields were measured on a home-built setup consisting of cw laser diodes (Thorlabs) routed onto samples placed in an integrating sphere (Newport 819C-SL-5.3). Emission was injected into a spectrometer (Andor Kymera 328i) and onto a silicon CCD detector.

### Photothermal deflection spectroscopy

Photothermal deflection spectroscopy (PDS) measurements were performed on thin films deposited on quartz substrates. The sample was illuminated with a monochromatic pump beam and immersed in FC-72 Fluorinert (3M Company). A fixed-wavelength continuous-wave laser probe beam passed through the region adjacent to the sample surface, where pump-induced heating generated a refractive-index gradient. The resulting deflection of the probe beam, proportional to the absorbed light at each pump wavelength, was detected using a photodiode and lock-in amplifier.

### Transient absorption spectroscopy

ps TA experiments were conducted on either a home-built system pumped by a regenerative Ti:sapphire amplifier (Solstice Ace, Spectra-Physics) emitting pulses centred at 800 nm at a rate of 1 kHz, or a commercial HARPIA (Light Conversion) system driven by Pharos (Light Conversion) centred at 1035 nm at a rate of 10 kHz. For the home-built system, non-collinear optical parametric amplifiers (NOPA) were tuned to output a desired probe region, and a further NOPA was tuned to provide narrowband pump pulses. Tunable pump pulses were generated in an Orpheus Neo (Light Conversion) unit. In both cases, one of the beams was optically delayed by a computer-controlled delay stage. The pump spectrum was filtered using appropriate band-pass filters to remove residual wavelengths, and its polarisation was set to the magic angle relative to the probe. After passing through the sample, the probe was dispersed with a grating spectrometer and measured with either a Si or InGaAs detector array. ns TA experiments were performed on a home-built setup with excitation by the second harmonic (532 nm) of a Q-switched Nd:YVO_4_ laser (Advanced Optical Technologies Ltd AOT-YVO-25QSPX, 500 Hz repetition rate) and the probe generated by LEUKOS Disco 1 supercontinuum laser (STM-1-UV, 1 kHz repetition rate). The time delay was varied using a Highland T560 delay generator. The probe was split with a 50:50 beam splitter to provide a pump-free reference beam. The transmitted probe and reference were collected with a silicon dual-line array detector read out by a custom board (Stresing Entwicklungsbüro).

### Transient photoluminescence spectroscopy

Time-resolved PL data in the ns-μs region were collected using an electrically-gated intensified CCD camera (Andor iStar DH740 CCI-010) after passing through a calibrated grating spectrometer (Andor SR303i). The samples were excited using pump pulses obtained from Orpheus-Lyra (Light Conversion) operating at 1 kHz. Overlapping time regions were used to compose the decays at multiple constant gate widths. Temperature-dependent measurements were performed using a closed-circuit helium cryostat (Oxford Instruments Optistat Dry BL4), compressor (Sumitomo HC-4E2) and temperature controller (Oxford Instruments Mercury iTC). Time-resolved PL data in the ps region were collected using an optically Kerr-gated HARPIA-TF system within the TA setup described above.

### Electron spin resonance

X-band ESR was acquired with a Bruker Biospin E680 or E580 EleXSys spectrometer using a Bruker ER4118-MD5-W1 resonator (9.7 GHz) in an Oxford Instruments CF935 cryostat. For pulsed ESR, an Applied Systems Engineering (ASE) amplifier with saturated powers of 1.5 kW was used. Temperature was maintained with an ITC-503S temperature controller and a CF-935 helium flow cryostat (both Oxford Instruments). For excited state ESR, photoexcitation was provided by Ekspla NT230 laser operating at a repetition rate of 50 Hz, with pulse energies of 0.5–1 mJ, pulse lengths of 3 ns transmitted at ca. 40% to the sample via the cryostat, mw shield and resonator windows. A liquid-crystal depolariser (DPP-25, ThorLabs) was placed in the laser path. The laser and spectrometer were synchronised with a delay generator (Stanford Research Systems, DG645). Quadrature was used in pulsed and cw detection. Unless otherwise specified, the trESR was collected at 40 dB microwave attenuation (ca. 20 μW mw power). Transient cw ESR spectra were simulated using EasySpin version 6.0.11,^59^ with the following Hamiltonian: $\widehat{H}={\widehat{H}}_{{{{\rm{Zeeman}}}}}+{\widehat{H}}_{{{{\rm{ex}}}}}+{\widehat{H}}_{{{{\rm{ZFS}}}},T}+{\widehat{H}}_{{{{\rm{dip}}}},RT}$, where ${\widehat{H}}_{{{{\rm{Zeeman}}}}}=\mu _{{{{\rm{B}}}}}{{{{\bf{B}}}}}_{0}\cdot ({g}_{T}{\widehat{{{{\bf{S}}}}}}_{T}+{g}_{R}{\widehat{{{{\bf{S}}}}}}_{R})$; ${\widehat{H}}_{{{{\rm{ex}}}}}=J({\widehat{{{{\bf{S}}}}}}_{T}\cdot {\widehat{{{{\bf{S}}}}}}_{R})$; ${\widehat{H}}_{{{{\rm{ZFS}}}},T}={D}_{T}({\widehat{S}}_{z,T}^{2}-\frac{2}{3})+ {E}_{T}({\widehat{S}}_{x,T}^{2}-{\widehat{S}}_{y,T}^{2})$; ${\widehat{H}}_{{{{\rm{dip}}}},RT}=\frac{{\mu }_{0}{g}_{T}{g}_{R}{\,\mu \,{{{\rm{}}}}}_{{{{\rm{B}}}}}^{2}}{4\pi {r}_{RT}^{3}}({\widehat{{{{\bf{S}}}}}}_{T}\cdot {\widehat{{{{\bf{S}}}}}}_{R}-3({\widehat{{{{\bf{S}}}}}}_{T}\cdot \widehat{{{{\bf{n}}}}})({\widehat{{{{\bf{S}}}}}}_{R}\cdot \widehat{{{{\bf{n}}}}}))$ and $\widehat{{{{\bf{n}}}}}=\sin {\theta }_{RT}\cos {\phi }_{RT}\widehat{{{{\bf{x}}}}}+\sin {\theta }_{RT}\sin {\phi }_{RT}\widehat{{{{\bf{y}}}}}+\cos {\theta }_{RT}\widehat{{{{\bf{z}}}}}$. A fixed value of isotropic exchange was used to set the strong exchange regime as determined from nutation experiments. The radical-triplet dipolar interaction strength is fixed using the distance obtained from TDDFT calculations and the point dipole approximation.^60^ The density matrix was populated in the triplet zero-field basis by explicitly constructing the projection operators, as shown in Supplementary Fig. 42. The spectra were fitted by optimising the triplet and radical populations, isotropic *g*-values, triplet dipolar constants, radical-triplet orientation angles and an additional weight *W*_*R*_ of a thermally polarised doublet component.

### Cyclic voltammetry

Electrochemistry was carried out on a PalmSens EmStat4X potentiostat in a three-electrode setup using a glassy carbon electrode (3.0 mm diameter) as the working electrode, platinum wire as the counter electrode and freshly activated silver wire as the Ag/Ag^+^ reference electrode. The silver wire was activated by immersing it in concentrated HCl solution to remove any silver oxides or other impurities, then rinsed with water and acetone and dried prior to each measurement. The reference electrode was calibrated against ferrocene/ferrocenium (Fc/Fc^+^) redox couple (the Fc/Fc^+^ half-wave potential was determined at 0.20 V vs. Ag/Ag^+^). The supporting electrolyte was 0.1 M solution of Bu_4_NPF_6_ in anhydrous THF, which was bubbled with Ar before each measurement to remove dissolved oxygen. Sample concentration was in the order of 10^−5^ M.

### Quantum chemical calculations

The doublet ground state structure of each molecule presented in this study was optimised at the Density Functional Theory (DFT) level in the unrestricted Kohn-Sham (UKS) formalism by using the *ω*B97X-D exchange-correlation functional and the 6-31G(d,p) basis set for all the atomic species. The excited-state properties were computed by means of time-dependent (TD) DFT calculations within the Tamm-Dancoff approximation (TDA)^61^, where the unrestricted LC-*ω*hPBE functional was used in conjunction with the Def2-TZVP basis set. For each molecule, the range-separation parameter *ω* was optimally tuned (OT) according to the “gap-tuning” procedure, as reported in earlier works^62,63^. To implicitly take into account the dielectric screening effects, the screened range-separated hybrid procedure (SRSH) was applied^64^. In this approach, the sum of the parameters *α* + *β*, defined within the DFT functional as the fraction of Hartree-Fock exchange included in the long-range domain, is set to be equal to 1/*ε*, where *α* equals the amount of Hartree-Fock exchange applied in the short-range domain (usually set at 0.2), *β* is an adjustable parameter varying as a function of *ε*, that is, the dielectric constant of the chosen solvent/environment. Calculations were carried out in toluene (TOL, *ε* = 2.37) and 2-methyltetrahydrofuran (MeTHF, *ε* = 6.97). We note that the unrestricted formalism does not allow for obtaining spin-contamination-free wave functions, and some excited states are inevitably affected by this issue. Hole-particle natural transition orbitals (NTOs) were produced to visually inspect the character of the relevant excitations for each group of radicals. Hole-electron separation distances and density overlaps were computed according to the detachment-attachment density formalism^65^. All the calculations were performed using the GAUSSIAN16 package suite^66^.

To estimate Coulomb integrals and electronic coupling strengths, semiempirical Pariser-Parr-Pople (PPP) theory calculations on molecular fragments were also performed.^43^. The radical-acene dyads are first divided into radical (TTM-carbazole) and acene fragments with separate molecular coordinate files starting from the optimised molecular geometries detailed above. The fragments are truncated to remove all *sp*^3^ atoms before being input into PPP calculations. The self-consistent electronic structures of these fragments are then solved separately using two versions of PPP theory: for acene fragments the conventional closed-shell PPP theory was used;^46–49^ and for radical fragments the recently devised ExROPPP method was used.^45,50^ Finally, perturbation theory is applied to calculate electronic couplings as detailed in Supplementary Note 2.

### Reporting summary

Further information on research design is available in the Nature Portfolio Reporting Summary linked to this article.

## Supplementary information

Supplementary Information

Reporting Summary

Transparent Peer Review File

## Source data

Source Data

## Data Availability

Source data are provided with this paper, and have been additionally deposited in the University of Cambridge Apollo repository and can be freely accessed at: 10.17863/CAM.128325.
