# Supplementary material for: Coulombic control of charge transfer in radicals with quartet recycling luminescence
Source: Nat Commun. 2026 May 22;17:7203. doi: 10.1038/s41467-026-72487-5 (PMC13396479; doi:10.1038/s41467-026-72487-5)
Supplement: Supplementary file 1 — Supplementary Information [file 41467_2026_72487_MOESM1_ESM.pdf]

Supplementary Information

for

Coulombic Control of Charge Transfer in Radicals with Quartet Recycling Luminescence

Lujo Matasovic,[1] Petri Murto,[1,2,3] Shilong Yu,[4] Wenzhao Wang,[4] James D. Green,[5] Giacomo Londi,[6] Weixuan Zeng,[2] Laura Brown,[2] William K. Myers,[7] Lars van Turnhout,[1] Konstantina Armadorou,[8] Avik Bhanja,[3] Sergiu Petrusca,[2] David Beljonne,[9] Yoann Olivier,[10] Feng Li,[*,4] Hugo Bronstein,[*,2] Timothy J. H. Hele,[*,5] Richard H. Friend,[*,1] and Sebastian Gorgon[*,1,7,11]

[1]*Cavendish Laboratory, University of Cambridge, Cambridge, UK*

[2]*Yusuf Hamied Department of Chemistry, University of Cambridge, Cambridge, UK*

[3]*Department of Chemistry and Materials Science, Aalto University, Espoo, Finland*

[4]*State Key Laboratory of Supramolecular Structure and Materials, Jilin University, Changchun, P. R. China*

[5]*Department of Chemistry, University College London, London, UK*

[6]*Department of Chemistry and Industrial Chemistry, University of Pisa, Pisa, Italy*

[7]*Centre for Advanced ESR, Department of Chemistry, University of Oxford, Oxford, UK*

[8]*Department of Chemical Engineering & Biotechnology, University of Cambridge, Cambridge, UK*

[9]*Laboratory for Chemistry of Novel Materials, University of Mons, Mons, Belgium*

[10]*Laboratory for Computational Modelling of Functional Materials, University of Namur, Namur, Belgium*

[11]*Department of Chemistry, The University of Tokyo, Tokyo, Japan*

* Corresponding Authors. E-mail addresses: lifeng01@jlu.edu.cn; hab60@cam.ac.uk; t.hele@ucl.ac.uk; rhf10@cam.ac.uk; sg911@cam.ac.uk

Contents

Suppl. Note 1. Synthesis and Characterisation **3**

S1.1 T-An, An-T-1Cz and An-T-3PCz 3

S1.2 T-3Cz-An and T-3Cz-Acr 14

Electrochemistry **19**

Photophysics **21**

Electron Spin Resonance **47**

Suppl. Note 2. Theoretical Considerations **51**

S2.1 Relevant states and their energies 51

S2.2 Perturbation Theory 54

S2.3 Design Rules 63

S2.4 Computational Details for ExROPPP calculations. 68

Quantum Chemical Calculations **73**

NMR Spectra **79**

Supplementary References **83**

Suppl. Note 1. Synthesis and Characterisation

S1.1 T-An, An-T-1Cz and An-T-3PCz

Characterisation and techniques.

NMR spectra were recorded on a 400 MHz Bruker Avance III HD (^{1}H, 400 MHz; ^{13}C, 100 MHz) spectrometer and on a 700 MHz Bruker TXO Cryoprobe (^{1}H, 700 MHz; ^{13}C, 175 MHz) spectrometer. Chemical shifts are reported in δ (ppm) relative to the solvent peak: chloroform-d ($CDCl_3$: ^{1}H, 7.26 ppm; ^{13}C, 77.16 ppm) and dichloromethane-d2 (CD_2Cl_2: ^{1}H, 5.32 ppm; ^{13}C, 53.84 ppm). High-resolution mass spectra were obtained by the Mass Spectrometry service at Yusuf Hamied Department of Chemistry, University of Cambridge. Flash chromatography was carried out using Biotage® Isolera™ Four System and Biotage® SNAP/Sfär Silica flash cartridges.

Materials and synthesis.

Synthesis of tris(2,4,6-trichlorophenyl)methane (α-HTTM) monomer has been reported previously by us and others.[1–3] Preparation of mesityl-substituted α-HTTM derivatives, 4'-(bis(2,4,6-trichlorophenyl)methyl)-3',5'-dichloro-2,4,6-trimethyl-1,1'-biphenyl (α-HM$_1$TTM), 4',4'''-((2,4,6-trichlorophenyl)methylene)bis(3',5'-dichloro-2,4,6-trimethyl-1,1'-biphenyl) (α-HM$_2$TTM) and 2-(4-(bis(3,5-dichloro-2',4',6'-trimethyl-[1,1'-biphenyl]-4-yl)methyl)-3,5-dichlorophenyl)-4,4,5,5-tet -ramethyl-1,3,2-dioxaborolane (M$_2$TTM-Bpin) is described in our recent work.[1] Other reagents, catalysts and (anhydrous) solvents were purchased from Merck, Fluorochem, Alfa Aesar and Acros Organics and used as received.

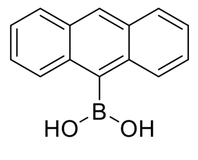

9-Anthraceneboronic acid (S1).

Adapted from a literature procedure,[4] to an oven-dried flask was added 9-bromoanthracene (1.00 g, 3.9 mmol, 1 equiv.) and the flask was subjected to three vacuum/Ar gas refill cycles. An-

hydrous THF (30 mL) was added and the mixture was cooled to −78 °C. A solution of n-BuLi (1.6 M in hexanes, 2.9 mL, 4.7 mmol, 1.2 equiv.) was added dropwise to the mixture. After the addition, the mixture was stirred at −78 °C for 30 min, then allowed to warm to room temperature and it was stirred another 30 min. The mixture was cooled again to −78 °C and trimethyl borate (0.56 mL, 5.1 mmol, 1.3 equiv.) was added dropwise. After stirring for 30 min at −78 °C, the mixture was allowed to warm to room temperature and it was stirred another 30 min. 1 M HCl solution was added to acidify the reaction mixture. Then, the mixture was diluted with hexane and extracted three times with water. The organic phase was dried over anhydrous $MgSO_4$ and solvent was removed. The crude product was purified by recrystallization from hexane/DCM mixture. The solids were collected by filtration and washed with cold hexane. After drying *in vacuo*, the target compound was collected as pale yellow solid (0.30 g, 34%). ^{1}H NMR (400 MHz, CD_2Cl_2) δ 8.48 (s, 1H), 8.15-8.07 (m, 2H), 8.07-8.00 (m, 2H), 7.55-7.43 (m, 4H), 5.25 (s, 2H). ^{13}C NMR (100 MHz, CD_2Cl_2) δ 134.06, 131.54, 129.20, 128.59, 128.39, 126.23, 125.64. TOF-MS-ASAP$^+$ Calcd. for $[C_{14}H_{11}BO_2]^+$: 222.0852. Found: m/z = 222.0844.

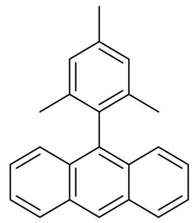

9-Mesitylanthracene (S2).

Adapted from a literature procedure,[5] to an oven-dried flask equipped with a condenser was added 9-bromoanthracene (4.50 g, 17.5 mmol, 1 equiv.), mesitylboronic acid (4.31 g, 26.3 mmol, 1.5 equiv.), $Pd(OAc)_2$ (0.118 g, 0.53 mmol, 0.03 equiv.), SPhos (0.431 g, 1.05 mmol, 0.06 equiv.) and K_3PO_4 (9.29 g, 43.8 mmol, 2.5 equiv.), and the flask was subjected to three vacuum/Ar gas refill cycles. Anhydrous toluene (100 mL) was added and the mixture was degassed by bubbling with Ar gas for 15 min and then heated to 100 °C in an oil bath for 48 h.

After cooling to room temperature, the mixture was diluted with chloroform and extracted three times with water. The organic phase was dried over anhydrous $MgSO_4$. Solvent was removed and

the crude product was purified with column chromatography over silica gel by gradually increasing the eluent polarity from hexane to 2 % DCM in hexane. Finally the solvent was removed and the solids were dried *in vacuo*. The target compound was collected as pale yellow crystals (3.76 g, 73 %). **^{1}H NMR**(400 MHz, $CDCl_3$) δ 8.50 (s, 1H), 8.07 (d, J = 8.5 Hz, 2H), 7.60-7.42 (m, 4H), 7.41-7.28 (m, 2H), 7.11 (s, 2H), 2.48 (s, 3H), 1.74 (s, 6H). **^{13}C NMR** (100 MHz, $CDCl_3$) δ 137.71, 137.24, 135.91, 134.64, 131.78, 129.91, 128.75, 128.39, 126.20, 126.13, 125.68, 125.30, 21.39, 20.11. FTMS-ESI$^+$ Calcd. for $[C_{23}H_{20}]^+$: 296.1560. Found: m/z = 296.1561.

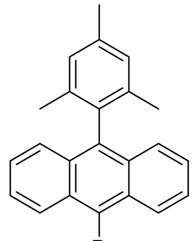

9-Bromo-10-mesitylanthracene (S3).

Adapted from literature procedures,[5,6] to an oven-dried flask equipped with a condenser and a dropping funnel was added compound S2 (3.00 g, 10.1 mmol, 1 equiv.) and the flask was subjected to three vacuum/Ar gas refill cycles. Anhydrous DCM (400 mL) was added. Through the dropping funnel, bromine (0.55 mL, 10.6 mmol, 1.05 equiv.) was added dropwise during and the mixture was stirred for 1 h at room temperature. The reaction mixture was treated with saturated sodium thiosulfate solution, and then extracted with hexane and washed three times with water. The organic phase was dried over anhydrous $MgSO_4$. Solvent was removed and the crude product was purified with column chromatography over silica gel by gradually increasing the eluent polarity from hexane to 5 % DCM in hexane. Finally the solvent was removed and the solids were dried *in vacuo*. The target compound was collected as pale yellow solid (3.15 g, 83 %). **^{1}H NMR** (700 MHz, $CDCl_3$) δ 8.62 (d, J = 8.8 Hz, 2H), 7.65-7.56 (m, 2H), 7.51 (d, J = 8.6 Hz, 2H), 7.41-7.33 (m, 2H), 7.10 (s, 2H), 2.46 (s, 3H), 1.70 (s, 6H). **^{13}C NMR** (175 MHz, $CDCl_3$) δ 137.59, 137.55, 136.70, 134.20, 130.68, 130.62, 128.48, 128.22, 127.20, 126.51, 126.01, 122.37, 21.40, 20.15. **TOF-MS-ASAP$^+$** Calcd. for $[C_{23}H_{19}Br]^+$: 374.0670. Found: m/z = 374.0653.

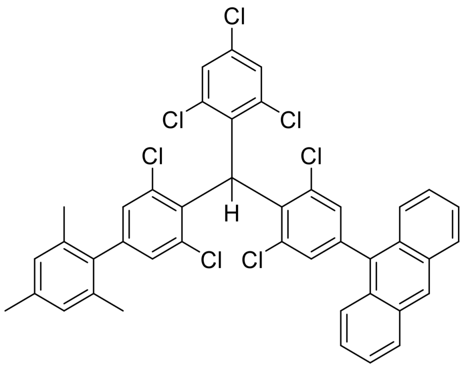

9-(3,5-Dichloro-4-((3,5-dichloro-2’,4’,6’-trimethyl-[1,1’-biphenyl]-4-yl) (2,4,6-trichlorophenyl)methyl)phenyl)anthracene (S4).

To an oven-dried flask equipped with a condenser was added αHM$_1$TTM (0.500 g, 0.78 mmol, 1 equiv.), compound S1 (0.174 g, 0.78 mmol, 1 equiv.), Pd(OAc)$_2$ (0.0035 g, 0.016 mmol, 0.02 equiv.), SPhos (0.0161 g, 0.039 mmol, 0.05 equiv.) and K_3PO_4 (0.266 g, 1.25 mmol, 1.6 equiv.), and the flask was subjected to three vacuum/Ar gas refill cycles. Anhydrous 1,4-dioxane (24 mL) was added and the mixture was degassed by bubbling with Ar gas for 15 min and then heated to 80 °C in an oil bath for 20 h. After cooling to room temperature, the mixture was diluted with hexane and extracted three times with water. The organic phase was dried over anhydrous $MgSO_4$. Solvent was removed and the crude product was purified with column chromatography over silica gel by gradually increasing the eluent polarity from hexane to 10 vol% DCM in hexane. Finally the solvent was removed and the solids were dried in vacuo. The target compound was collected as off-white solid (0.216 g, 35%). ^{1}H NMR (400 MHz, CD_2Cl_2) δ 8.56 (s, 1H), 8.13–8.03 (m, 2H), 7.70–7.61 (m, 2H), 7.55–7.41 (m, 6H), 7.41–7.29 (m, 2H), 7.23 (dd, J = 15.3, 1.8 Hz, 1H), 7.10 (dd, J = 24.7, 1.8 Hz, 1H), 7.05 (s, 1H), 6.98–6.91 (m, 2H), 2.31 (d, J = 3.2 Hz, 3H), 2.06 (s, 6H). 13C NMR (100 MHz, CD2Cl2) δ 143.01, 140.41, 138.57, 138.11, 137.98, 137.91, 137.80, 137.34, 137.28, 137.21, 137.15, 136.30, 135.96, 135.90, 135.54, 135.47, 135.20, 135.13, 133.91, 133.62, 133.35, 133.21, 131.77, 131.68, 131.65, 131.58, 130.55, 130.27, 130.24, 129.98, 128.85, 128.55, 127.94, 126.56, 126.51, 126.38, 126.33, 125.75, 50.96, 21.18, 20.72, 20.67. TOF-MS-ASAP$^+$ Calcd. for $[C_{42}H_{27}Cl_7]^+$: 775.9932. Found: m/z = 775.9946.

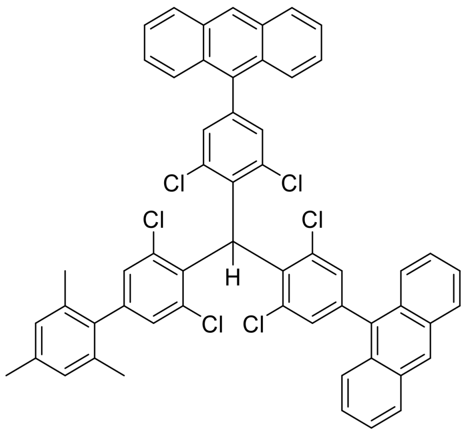

9,9'-(((3,5-Dichloro-2',4',6'-trimethyl-[1,1'-biphenyl]-4-yl)methylene) bis(3,5-dichloro-4,1-phenylene))dianthracene (S5).

In the reaction above, a doubly anthracene-coupled compound was collected as a by-product (0.040 g, 6 %). **^{1}H NMR** (400 MHz, CD_2Cl_2) δ 8.57 (d, J = 3.0 Hz, 2H), 8.16-8.03 (m, 4H), 7.78-7.67 (m, 4H), 7.58-7.37 (m, 12H), 7.29 (s, 1H), 7.25 (dd, J = 49.3 Hz, 1.7 Hz, 2H), 6.97 (s, 2H), 2.33 (s, 3H), 2.10 (d, J = 7.3 Hz, 6H). **^{13}C NMR** (100 MHz, CD_2Cl_2) δ 142.91, 140.32, 138.21, 138.00, 137.88, 137.51, 137.48, 137.36, 136.41, 136.02, 135.96, 135.77, 135.69, 134.45, 133.76, 133.34, 131.77, 131.70, 131.67, 131.60, 130.33, 130.30, 130.28, 130.01, 128.84, 128.56, 128.54, 127.91, 126.56, 126.52, 126.46, 126.43, 126.40, 125.77, 51.37, 21.19, 20.77, 20.73. **TOF-MS-ASAP^{+}** Calcd. for $[C_{56}H_{36}Cl_6]^+$: 918.0948. Found: m/z = 918.0994.

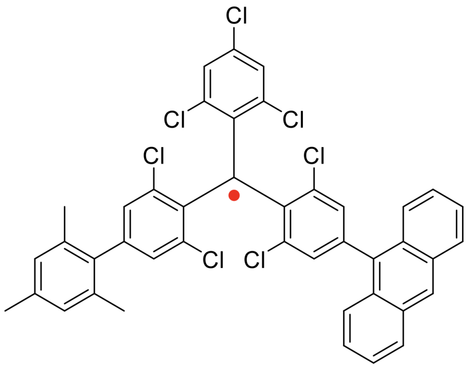

9-(3,5-Dichloro-4-((3,5-dichloro-2',4',6'-trimethyl-[1,1'-biphenyl]-4-yl) (2,4,6-trichlorophenyl) methyl)phenyl)anthracenyl radical (S6).

Following our general procedure for radical conversion (below), compound S4 (0.090 g, 0.12 mmol, 1 equiv.), anhydrous THF (4.5 mL), anhydrous DMSO (13.5 mL) and 40 % Bu_4NOH (aq) (0.15 mL, 0.23 mmol, 2 equiv.) were used in the reaction and the mixture was stirred at room temperature for 20 h. *p*-Chloranil (0.071 g, 0.29 mmol, 2.5 equiv.) was added and the mixture was stirred another 1 h. The mixture was diluted with hexane, extracted three times with water and the organic phase was dried over anhydrous $MgSO_4$. Solvent was removed and the crude product was purified with column chromatography as described above for compound S4. After drying *in vacuo*, the target compound was collected as red solid (0.073 g, 81 %). **^{1}H NMR** (400 MHz, CD_2Cl_2) broadened aromatic signals and mesityl CH_3 signals not resolved. **TOF-MS-ASAP$^+$** Calcd. for $[C_{42}H_{26}Cl_7]^{H+}$: 774.9854. Found: m/z = 774.9856.

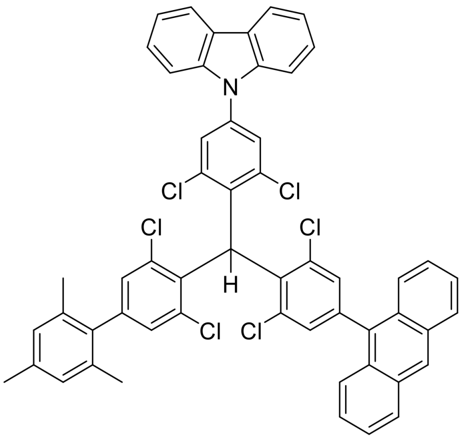

9-(4-((4-(Anthracen-9-yl)-2,6-dichlorophenyl)(3,5-dichloro-2',4',6'-trimethyl-[1,1'-biphenyl]-4-yl)methyl)-3,5-dichlorophenyl)-9H-carbazole (αH-An-T-1Cz).

Using a modified literature procedure,[7] to an oven-dried microwave vial was added compound S6 (0.050 g, 0.06 mmol, 1 equiv.), 9H-carbazole (0.016 g, 0.10 mmol, 1.5 equiv.), $Pd(OAc)_2$ (0.0014 g, 0.006 mmol, 0.1 equiv.), SPhos (0.0079 g, 0.019 mmol, 0.3 equiv.) and Cs_2CO_3 (0.063 g, 0.19 mmol, 3 equiv.), and the vial was subjected to three vacuum/Ar gas refill cycles. Anhydrous toluene (3 mL) was added and the mixture was degassed by bubbling with Ar gas for 15 min and then heated to 80 °C in an oil bath for 14 h. After cooling to room temperature, the mixture was diluted with hexane and extracted three times with water. The organic phase was dried over anhydrous $MgSO_4$.

Solvent was removed and the crude product was purified with column chromatography over silica gel by gradually increasing the eluent polarity from hexane to 10 vol% DCM in hexane. Finally the solvent was removed and the solids were dried in vacuo. The product was collected as green solid (0.054 g, 92%). The compound is a mixture of radical and α-hydrogenated product. ^{1}H NMR indicates the presence of αH species and the compound is therefore reported as αH-An-T-1Cz. 1H NMR (400 MHz, CD_2Cl_2) δ 8.08 (d, J = 7.7 Hz, 2H), 7.49–7.40 (m, 4H), 7.23 (t, J = 7.4 Hz, 2H). Remaining signals (27H) not resolved.

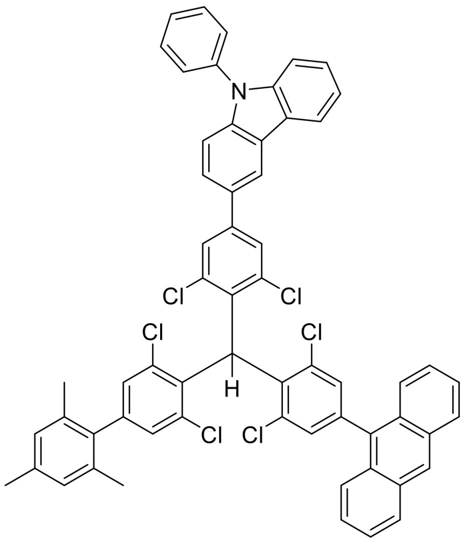

3-(4-((4-(Anthracen-9-yl)-2,6-dichlorophenyl)(3,5-dichloro-2',4',6'-trimethyl-[1,1'-biphenyl]-4-yl)methyl)-3,5-dichlorophenyl)-9-phenyl-9H-carbazole (αH-An-T-3PCz).

To an oven-dried microwave vial was added compound S4 (0.050 g, 0.06 mmol, 1 equiv.), (9-phenyl-9 H-carbazol-3-yl)boronic acid (0.028 g, 0.10 mmol, 1.5 equiv.), $Pd(OAc)_2$ (0.0003 g, 0.001 mmol, 0.02 equiv.), SPhos (0.0013 g, 0.003 mmol, 0.05 equiv.) and K_3PO_4 (0.033 g, 0.15 mmol, 2.4 equiv.), and the vial was subjected to three vacuum/Ar gas refill cycles. Anhydrous 1,4-dioxane (2 mL) was added and the mixture was degassed by bubbling with Ar gas for 15 min and then heated to 80 °C in an oil bath for 20 h. After cooling to room temperature, the mixture was diluted with hexane and extracted three times with water. The organic phase was dried over anhydrous $MgSO_4$. Solvent was removed and the crude product was purified with column chromatography over silica

gel by gradually increasing the eluent polarity from hexane to 10 % DCM in hexane. The product was further purified by recrystallization from EtOH/DCM mixture. The solids were collected by filtration. After drying *in vacuo*, the target compound was collected as white solid (0.047 g, 74 %). **^{1}H NMR** (400 MHz, CD_2Cl_2) δ 8.56 (s, 1H), 8.45 (dd, J = 4.5 Hz, 1.9 Hz, 1H), 8.22 (dd, J = 7.8 Hz, 3.4 Hz, 1H), 8.08 (d, J = 8.3 Hz, 2H), 7.87 (dd, J = 10.1 Hz, 2.1 Hz, 1H), 7.77-7.60 (m, 8H), 7.54-7.43 (m, 9H), 7.38-7.31 (m, 2H), 7.26 (dd, J = 12.5 Hz, 1.8 Hz, 1H), 7.18 (s, 1H), 7.14 (dd, J = 24.2 Hz, 1.8 Hz, 1H), 6.96 (t, J = 4.4 Hz, 2H), 2.32 (d, J = 4.0 Hz, 3H), 2.14-2.05 (m, 6H). **^{13}C NMR** (100 MHz, CD_2Cl_2) δ 143.01, 142.76, 141.88, 141.35, 140.16, 138.25, 138.21, 138.16, 138.02, 137.97, 137.84, 137.76, 137.65, 137.39, 137.27, 136.40, 135.99, 135.93, 135.78, 135.73, 134.51, 134.18, 134.11, 133.77, 133.29, 133.22, 131.68, 131.64, 131.54, 130.39, 130.31, 130.25, 130.15, 129.95, 129.89, 128.95, 128.82, 128.53, 128.10, 127.87, 127.39, 127.17, 126.85, 126.53, 126.45, 126.42, 125.74, 125.31, 124.38, 123.65, 120.74, 119.12, 110.72, 110.44, 51.16, 21.18, 20.75, 20.71. **TOF-MS-ASAP$^+$** Calcd. for $[C_{60}H_{39}Cl_6N]^+$: 984.1292. Found: m/z = 984.1273.

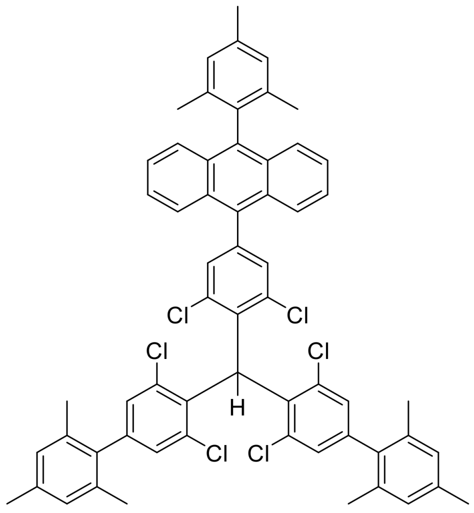

9-(4-(Bis(3,5-dichloro-2’,4’,6’-trimethyl-[1,1’-biphenyl]-4-yl)methyl) -3,5-dichlorophenyl)-10-mesitylanthracene (αH-T-An).

To an oven-dried microwave vial was added M_2TTM-Bpin (0.250 g, 0.20 mmol, 1 equiv.), compound S3 (0.115 g, 0.24 mmol, 1.2 equiv.), $Pd(OAc)_2$ (0.0011 g, 0.005 mmol, 0.02 equiv.),

SPhos (0.0050 g, 0.012 mmol, 0.05 equiv.) and K_3PO_4 (0.099 g, 0.47 mmol, 1.9 equiv.), and the vial was subjected to three vacuum/Ar gas refill cycles. Anhydrous 1,4-dioxane (2.5 mL) was added and the mixture was degassed by bubbling with Ar gas for 15 min and then heated to 80 °C in an oil bath for 24 h. After cooling to room temperature, the mixture was diluted with hexane and extracted three times with water. The organic phase was dried over anhydrous $MgSO_4$. Solvent was removed and the crude product was purified with column chromatography over silica gel by gradually increasing the eluent polarity from hexane to 10 vol% DCM in hexane. Finally the solvent was removed and the solids were dried in vacuo. The target compound was collected as off-white solid (0.189 g, 95%). 1H NMR (400 MHz, $CDCl_3$) δ 7.75 (t, J = 8.9 Hz, 2H), 7.61–7.51 (m, 3H), 7.48–7.40 (m, 3H), 7.38–7.31 (m, 2H), 7.27 (d, J = 1.7 Hz, 1H), 7.24 (d, J = 1.7 Hz, 1H), 7.18 (s, 1H), 7.16–7.07 (m, 4H), 6.97 (d, J = 3.3 Hz, 4H), 2.48 (s, 3H), 2.35 (d, J = 2.4 Hz, 6H), 2.10 (d, J = 8.4 Hz, 12H), 1.77 (d, J = 5.2 Hz, 6H). ^{13}C NMR (100 MHz, $CDCl_3$) δ 142.27, 140.14, 137.86, 137.73, 137.72, 137.55, 137.42, 137.13, 137.09, 137.04, 136.21, 135.91, 135.89, 135.85, 135.83, 135.39, 134.63, 134.38, 134.31, 133.20, 133.15, 131.45, 131.29, 129.94, 129.90, 129.52, 129.47, 129.44, 128.47, 128.34, 126.71, 126.64, 126.36, 126.01, 125.93, 125.58, 50.88, 21.40, 21.21, 20.79, 20.77, 20.24, 20.22. FTMS-ESI$^+$ Calcd. for $[C_{60}H_{48}Cl_6]^+$: 978.1882. Found: m/z = 978.1896.

General Procedure for Radical Conversion.

Following our previously reported procedure,[1,8] αH precursor (1 equiv.) was added into a microwave vial and the vial was subjected to three vacuum/Ar gas refill cycles. Anhydrous THF was added to dissolve all starting material followed by addition of anhydrous DMSO in a 1:3 (v/v) THF/DMSO ratio making a 0.5 wt% solution of the αH precursor. The mixture was bubbled with Ar gas for 15 min and covered from light. In the darkness, 40% Bu_4NOH (aq) (2 equiv.) (bubbled with Ar gas for 15 min prior to use) was added and the mixture was stirred at room temperature for 20 h. *p*-Chloranil (2.5 equiv.) was added, and the mixture was stirred another 1 h. The mixture was diluted with hexane, extracted three times with water and the organic phase was dried over anhydrous $MgSO_4$. Solvent was removed and the crude product was purified with column chro-

matography over silica gel by gradually increasing the eluent polarity from hexane to 5–20 vol% DCM in hexane. Finally, the solvent was removed and the solids were dried *in vacuo*. All radicals were stable under ambient air in both solution and solid state, but they were stored under inert gas in the dark as a standard procedure. Details of individual reactions and deviations from this procedure are provided below.

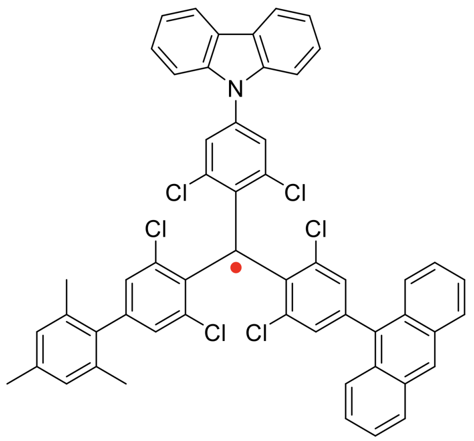

9-(4-((4-(Anthracen-9-yl)-2,6-dichlorophenyl)(3,5-dichloro-2',4',6'-trimethyl-[1,1'-biphenyl]-4-yl)methyl)-3,5-dichlorophenyl)-9H-carbazolyl radical (An-T-1Cz).

αH-An-T-1Cz (0.054 g, 0.06 mmol, 1 equiv.), 40% Bu_4NOH (aq) (0.08 mL, 0.12 mmol, 2 equiv.), *p*-chloranil (0.036 g, 0.15 mmol, 2.5 equiv.). The target compound was collected as green solid (0.043 g, 80%). ^{1}H NMR (400 MHz, CD_2Cl_2) broadened aromatic signals and mesityl CH_3 signals not resolved. TOF-MS-ASAP$^+$ Calcd. for $[C_{54}H_{34}Cl_6N]H^+$: 907.0901. Found: m/z = 907.0909.

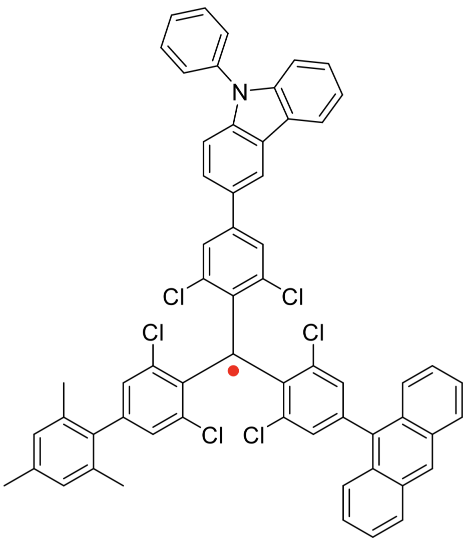

3-(4-((4-(Anthracen-9-yl)-2,6-dichlorophenyl)(3,5-dichloro-2',4',6'-trimethyl-[1,1'-biphenyl]-4-yl)methyl)-3,5-dichlorophenyl)-9-phenyl-9H-carbazolyl radical (An-T-3PCz).

αH-An-T-3PCz (0.020 g, 0.02 mmol, 1 equiv.), 40% Bu_4NOH (aq) (0.03 mL, 0.04 mmol, 2 equiv.), *p*-chloranil (0.012 g, 0.05 mmol, 2.5 equiv.). The following eluent system was used in column chromatography: the solvent polarity was gradually increased from hexane to 10 vol% DCM/toluene (in 1:1 ratio) in hexane. The target compound was collected as green solid (0.019 g, 95%). ^{1}H NMR (400 MHz, CD_2Cl_2) broadened aromatic signals and mesityl CH_3 signals not resolved. TOF-MS-ASAP$^+$ Calcd. for $[C_{60}H_{38}Cl_6N]^+$: 982.1135. Found: m/z = 982.1134.

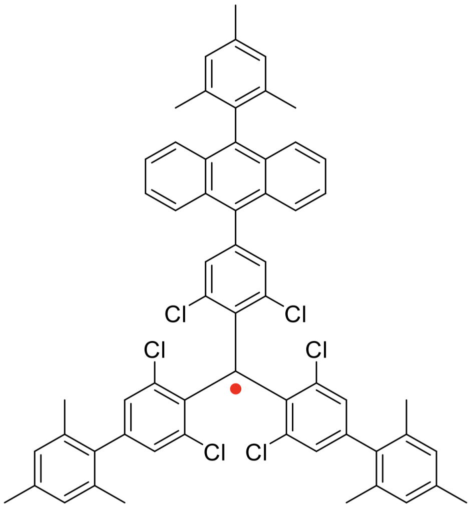

9-(4-(Bis(3,5-dichloro-2',4',6'-trimethyl-[1,1'-biphenyl]-4-yl)methyl)-3,5-dichlorophenyl) -10-mesitylanthracenyl radical (T-An).

αH-T-An (0.100 g, 0.10 mmol, 1 equiv.), 40% Bu_4NOH (aq) (0.14 mL, 0.20 mmol, 2 equiv.), *p*-chloranil (0.063 g, 0.25 mmol, 2.5 equiv.). The target compound was collected as red solid (0.091 g, 91%). ^{1}H NMR (400 MHz, $CDCl_3$) δ 2.52 (s, 3H), 1.85 (s, 6H). Aromatic signals (20H) and remaining mesityl CH_3 signals (18H) not resolved. FTMS-ESI$^+$ Calcd. for $[C_{60}H_{47}Cl_6]^+$: 977.1803. Found: $m/z = 977.1804$.

S1.2 T-3Cz-An and T-3Cz-Acr

All chemical reagents and solvents were purchased from commercial suppliers and used without further purification (unless otherwise stated). Tetrahydrofuran (THF) was distilled before being used. Column chromatography was performed with silica gel (200–300 mesh).

The ^{1}H nuclear magnetic resonance (NMR) spectra were recorded with a Bruker AVANCE-III 500 NMR spectrometer for deuterated dimethyl sulfoxide (DMSO) samples at ambient temperature. MALDI-TOF mass spectra were recorded on a Bruker Autoflex Speed TOF/TOF mass spectrometer with a DCTB matrix. Elemental analysis (EA) was performed on an Elementar Vario micro cube elemental analyser. EPR spectra were recorded in toluene solution on a Bruker ELEXSYS-II E500 CW-EPR spectrometer at room temperature.

HTTM and TTM were prepared according to our previous report (Suppl. Fig. 1). A general procedure for the synthesis of radicals 5–6 is given in Suppl. Fig. 2.

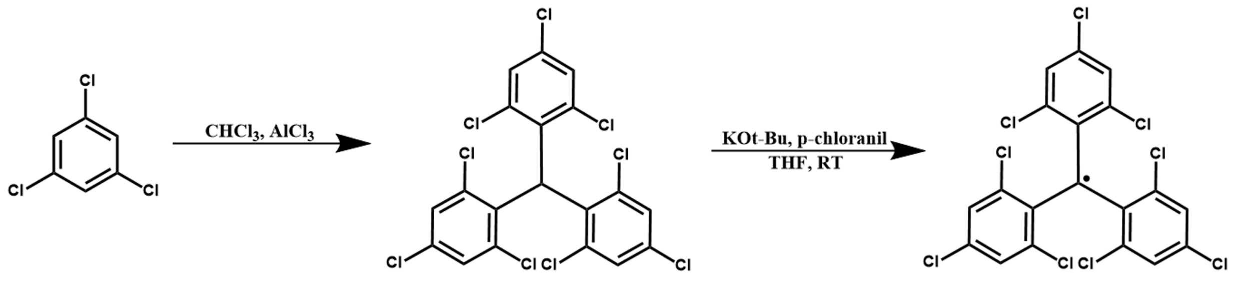

Suppl. Fig. 1: Synthesis of HTTM and TTM.

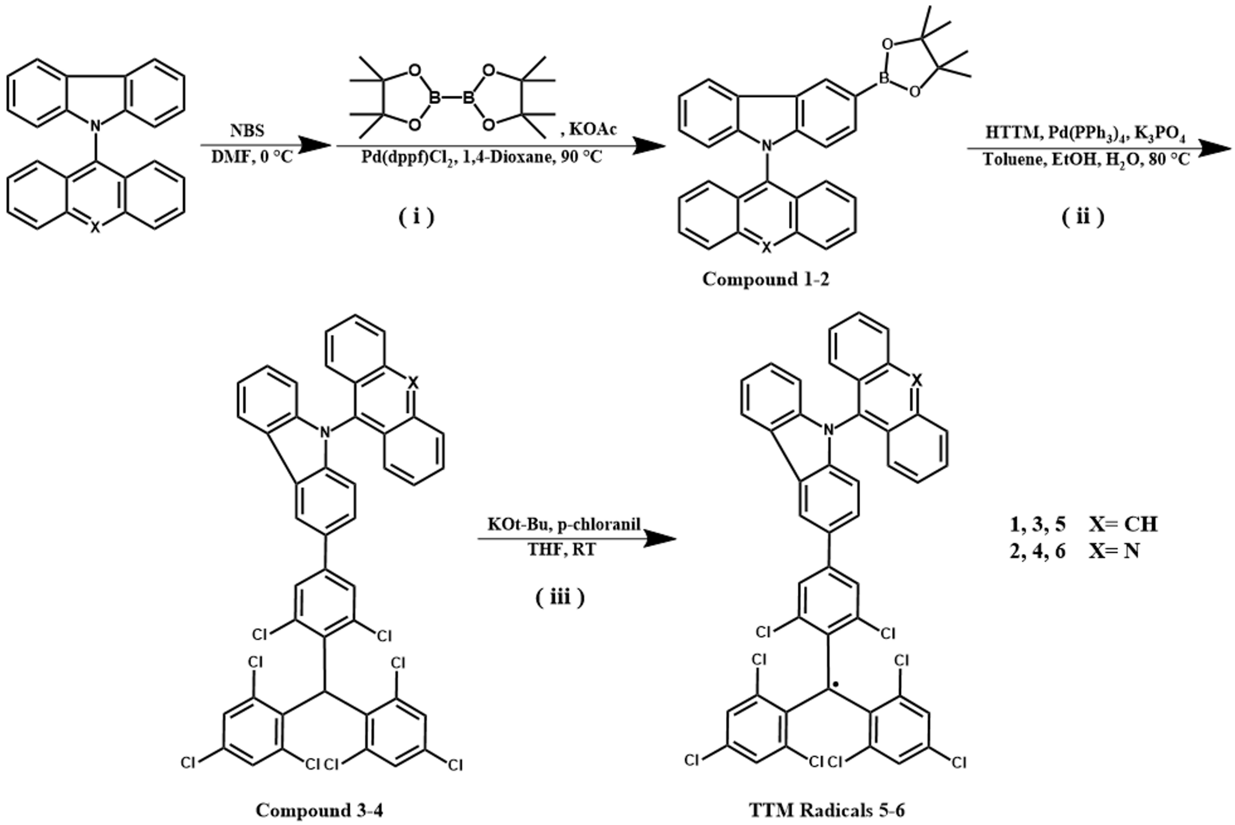

Suppl. Fig. 2: Synthesis of T-3Cz-An (Radical 5) and T-3Cz-Acr (Radical 6).

(i): Under an argon atmosphere, 9-(anthracen-9-yl)-9H-carbazole (1.0 mmol) was dissolved in dry DMF (50 mL) at 0 °C. N-Bromosuccinimide (NBS, 1.0 mmol) was dissolved in dry DMF (10 mL) and dropwise added into the reaction system at 0 °C. After 30 min, the mixture was brought to room temperature and stirred overnight. The mixture was poured into water, and the crude solid product was collected by filtration. Under argon atmosphere, the crude product (0.29 g), 4,4,4’,4’,5,5,5’,5’-octamethyl-2,2’-bi(1,3,2-dioxaborolane) (1.37 mmol), KOAc (1.03 mmol), and $Pd(dppf)Cl_2$ (0.034 mmol) were dissolved in dry 1,4-dioxane (100 mL). The mixture was stirred at 90 °C for 48 h. After cooling to room temperature, the mixture was poured into saturated NH_4Cl aqueous solution and extracted three times with dichloromethane. The combined organic layers were dried and evaporated under vacuum. The crude product was purified by silica gel column chromatography (petroleum ether:dichloromethane = 10:1 v/v). The desired 3Cz-An-B (Compound 1) was obtained. The synthesis of 3Cz-Acr-B (Compound 2) follows the same procedure.

Compound 1: white powder (0.24 g, 48%); ^{1}H NMR (500 MHz, CD_2Cl_2) δ 8.80 (s, 2H), 8.38 (d, J = 7.7 Hz, 1H), 8.25 (d, J = 8.6 Hz, 2H), 7.72 (d, J = 7.3 Hz, 1H), 7.58–7.54 (m, 2H), 7.40–7.30 (m, 4H), 7.23 (d, J = 9.2 Hz, 2H), 6.74 (d, J = 8.2 Hz, 2H), 1.42 (s, 12H). MALDI-TOF-MS (m/z): Calcd. for $C_{32}H_{28}BNO_2$, 469.2213; Found: 469.4812 (Fig. S1).

Compound 2: white powder (0.25 g, 52%); ^{1}H NMR (500 MHz, CD_2Cl_2) δ 8.75 (s, 1H), 8.43 (d, J = 8.2 Hz, 2H), 8.33 (d, J = 7.5 Hz, 1H), 7.89–7.81 (m, 2H), 7.71 (d, J = 8.0 Hz, 1H), 7.36 (tt, J = 23.2, 7.2 Hz, 6H), 6.74 (d, J = 7.5 Hz, 2H), 1.38 (s, 12H). MALDI-TOF-MS (m/z): Calcd. for $C_{31}H_{27}BN_2O_2$, 470.2166; Found: 470.5429 (Fig. S2).

(ii): 3Cz-An-B (1.0 mmol) and HTTM (2.0 mmol) were dissolved in toluene (12 mL), K_3PO_4 aqueous solution (8 mL, 2 M), and ethanol (4 mL). $Pd(PPh_3)_4$ (0.05 mmol) was added under argon. The mixture was stirred at 80 °C for 48 h in the dark. After cooling to room temperature, the mixture was extracted with dichloromethane, dried over $MgSO_4$, and evaporated. Purification by silica gel column chromatography (petroleum ether:ethyl acetate = 5:1, v/v) yielded HTTM-3Cz-An (Compound 3). HTTM-3Cz-Acr (Compound 4) was synthesized similarly.

Compound 3: white powder (0.34 g, 40%); ^{1}H NMR (500 MHz, DMSO) δ 9.01 (s, 1H), 8.95 (d, J = 1.3 Hz, 1H), 8.54 (d, J = 7.6 Hz, 1H), 8.35 (d, J = 8.6 Hz, 2H), 8.02 (d, J = 1.8 Hz, 1H), 7.86–7.77 (m, 3H), 7.72 (dd, J = 8.6, 1.5 Hz, 1H), 7.64–7.56 (m, 4H), 7.42 (dd, J = 8.1, 7.2 Hz, 2H), 7.34 (dt, J = 22.8, 7.0 Hz, 2H), 7.09 (d, J = 8.7 Hz, 2H), 6.73 (s, 1H), 6.68 (dd, J = 15.8, 8.3 Hz, 2H). MALDI-TOF-MS (m/z): Calcd. for $C_{45}H_{23}Cl_8N$, 860.9280; Found: 861.3949 (Fig. S3).

Compound 4: light yellow powder (0.33 g, 38%); ^{1}H NMR (500 MHz, DMSO) δ 8.97 (s, 1H), 8.56 (d, J = 7.7 Hz, 1H), 8.42 (d, J = 8.7 Hz, 2H), 8.04 (s, 1H), 7.95 (t, J = 7.2 Hz, 2H), 7.79 (dd, J = 42.8, 14.4 Hz, 4H), 7.58 (d, J = 35.9 Hz, 4H), 7.38 (dt, J = 13.9, 6.4 Hz, 2H), 7.27 (d, J = 8.3 Hz, 2H), 6.93–6.67 (m, 3H). MALDI-TOF-MS (m/z): Calcd. for $C_{44}H_{22}Cl_8N_2$, 861.9232; Found: 862.4079 (Fig. S4).

(iii): Under argon and in the dark, KO*t*-Bu (2.33 mmol) was added to a THF solution of HTTM-3Cz-An (0.23 mmol). The solution was stirred for 4 h at room temperature. Then tetrachloro-*p*-benzoquinone (1.17 mmol) was added, and the reaction stirred for another 2 h. Solvent was removed

in vacuo, and the crude product purified by silica gel chromatography (petroleum ether:dichloromethane = 8:1, v/v). T-3Cz-An (Radical 5) was obtained. T-3Cz-Acr (Radical 6) followed the same route.

Radical 5: dark green powder (0.15 g, 74%); MALDI-TOF-MS (m/z): Calcd. for $C_{45}H_{22}Cl_8N\bullet$, 859.9201; Found: 860.5204 (Fig. S5). Elem. Anal.: Calcd. C 62.83, H 2.58, N 1.63; Found: C 62.48, H 2.77, N 1.60.

Radical 6: dark green powder (0.16 g, 82%); MALDI-TOF-MS (m/z): Calcd. for $C_{44}H_{21}Cl_8N_2\bullet$, 860.9154; Found: 861.4955 (Fig. S6). Elem. Anal.: Calcd. C 61.36, H 2.46, N 3.25; Found: C 61.64, H 2.66, N 3.11.

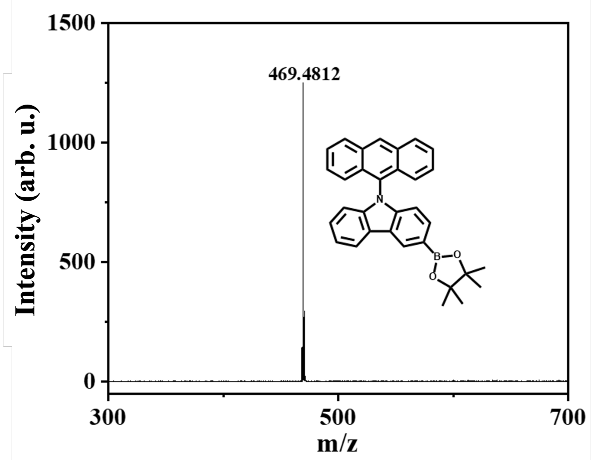

Suppl. Fig. 3: Mass Spectrum of 3Cz-An-B (Compound 1).

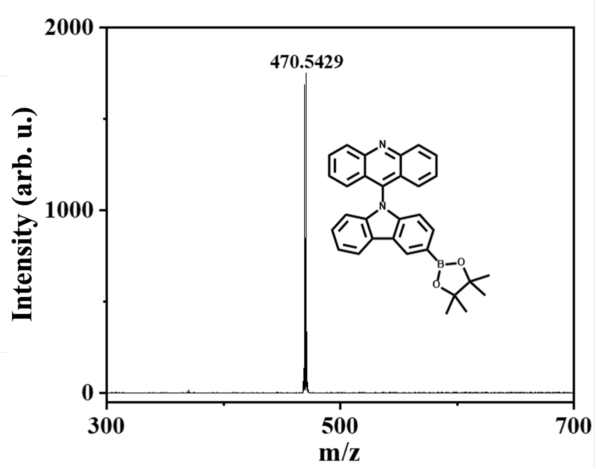

Suppl. Fig. 4: Mass Spectrum of 3Cz-Acr-B (Compound 2).

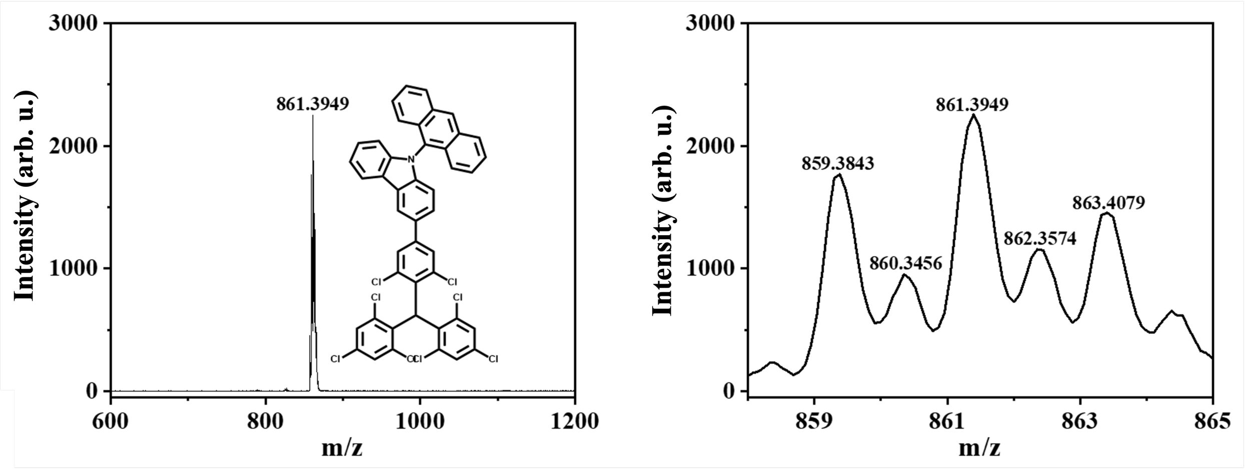

Suppl. Fig. 5: Mass Spectrum of HTTM-3Cz-An (Compound 3).

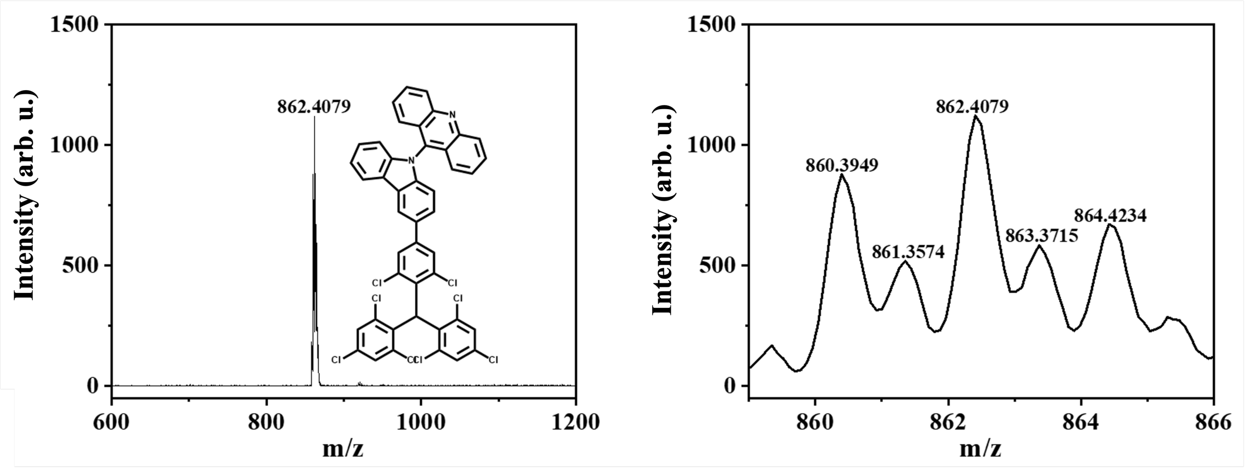

Suppl. Fig. 6: Mass Spectrum of HTTM-3Cz-Acr (Compound 4).

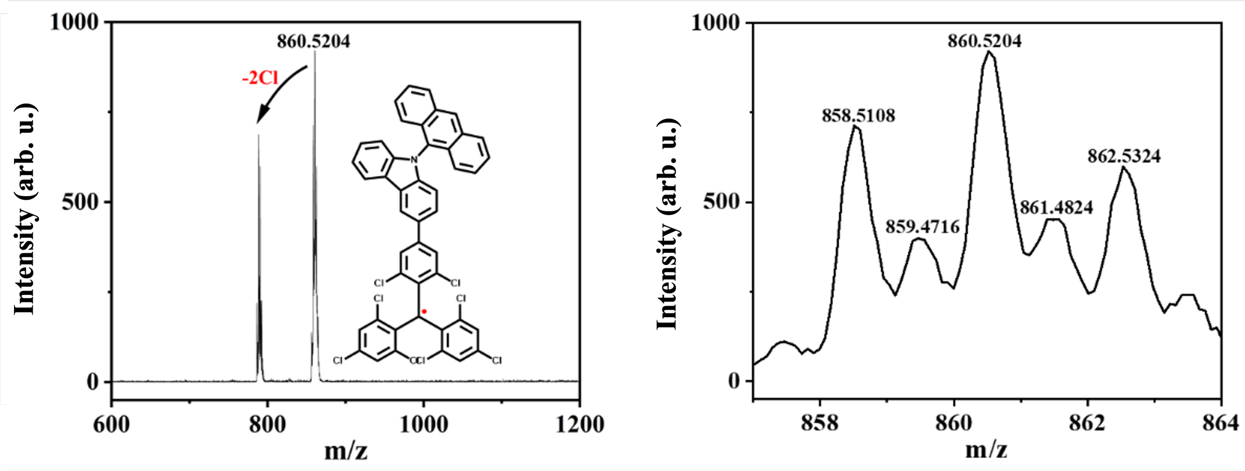

Suppl. Fig. 7: Mass Spectrum of T-3Cz-An (Radical 5).

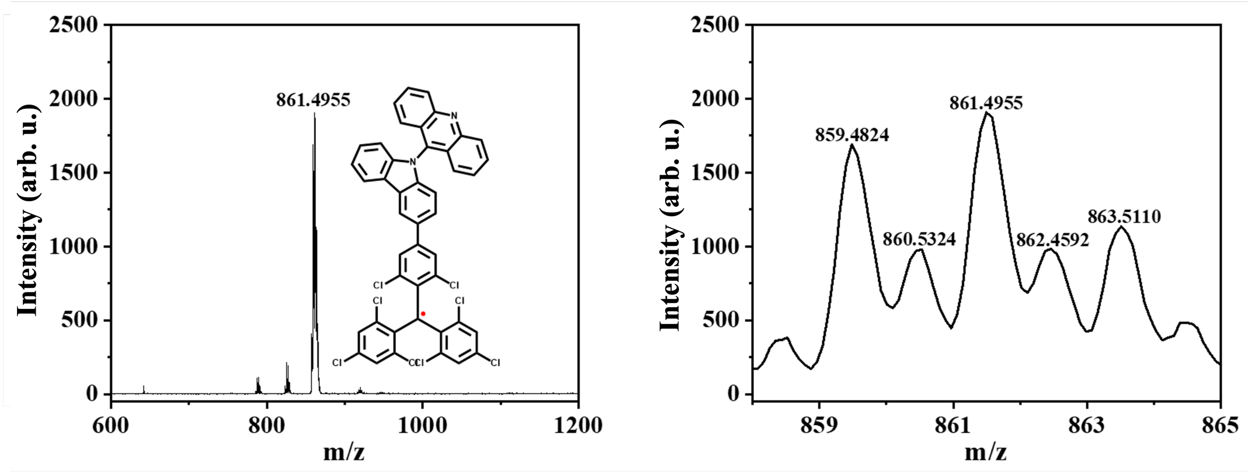

Suppl. Fig. 8: Mass Spectrum of T-3Cz-Acr (Radical 6).

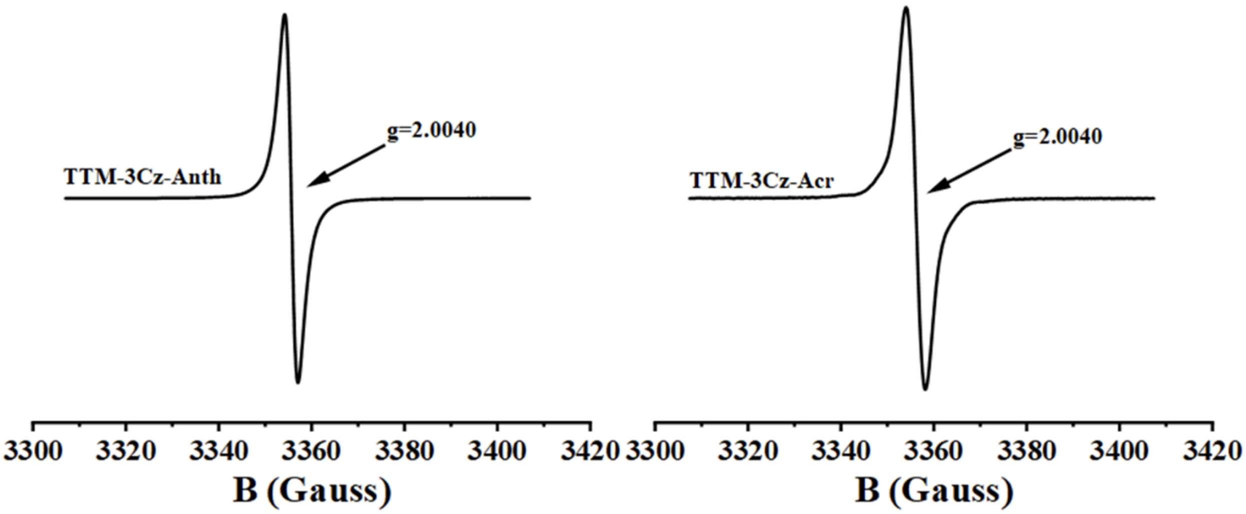

Suppl. Fig. 9: cw X-band EPR measured in toluene with a 10^{-3} M concentration at room temperature.

Electrochemistry

Suppl. Tab. 1: Redox potentials and the energy of the corresponding electron transfer process, estimated using the Weller equation.[9]

Material	E_{ox} [V]	E_{red} [V]	E(CT) [eV]
T-An	1.24	-1.02	2.24
An-T-1Cz	1.16	-0.95	2.09
An-T-3PCz	1.15	-1.04	2.09
T-3Cz-An	1.28	-0.93	2.33
T-3Cz-Acr	1.25	-0.90	2.27

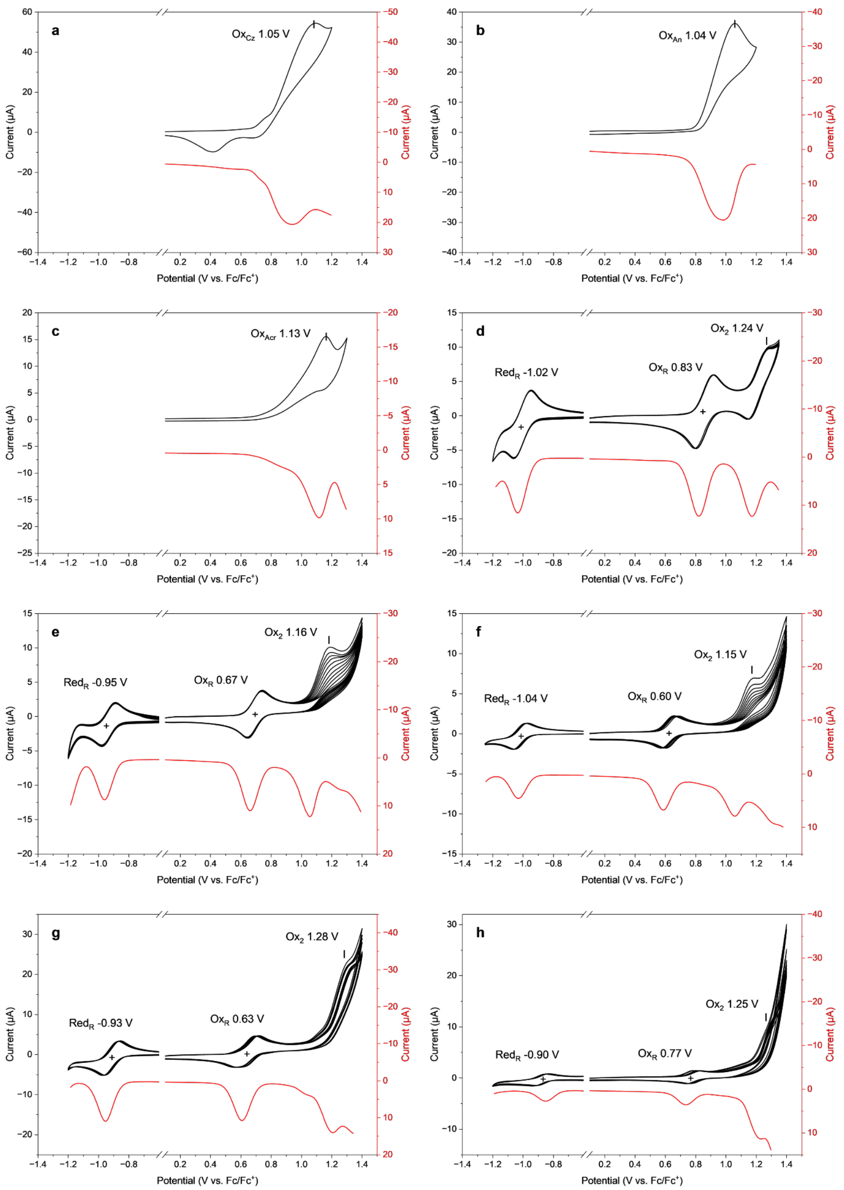

Suppl. Fig. 10: Electrochemistry for the radicals and their subunits. Cyclic voltammetry (black traces) and differential pulse voltammetry (red traces) for (a) carbazole, (b) anthracene and (c) acridine units and (d) T-An, (e) An-T-1Cz, (f) An-T-3PCz, (g) T-3Cz-An and (h) T-3Cz-Acr radicals. Cyclic voltammetry shows 10 reduction and oxidation cycles in the cathodic and anodic range, respectively, at a scan rate of 100 mVs^{-1}. The reversible reduction and oxidation waves are assigned to the radical site, R, in (d–h) and they are reported as half-wave potentials indicated by plus signs. The second, irreversible oxidation wave is that of carbazole (a), anthracene (b, d), acridine (c) or their combination (e–h) and it is reported as a peak potential indicated by a vertical line. Differential pulse voltammetry is scanned from the cathodic to the anodic range at a rate of 25 mVs^{-1} and the peaks align well with cyclic voltammetry.

Photophysics

Suppl. Tab. 2: Experimentally estimated 0–0 state energies in low polarity environments.

Material	E(^{2}CT$_{Cz}$) [eV]	E(^{2}CT$_{Ac}$) [eV]
T-3PCz	1.9	
T-An		1.8
An-T-1Cz	2.0	1.8
An-T-3PCz	1.9	1.8
T-3Cz-An	1.9	2.0
T-3Cz-Acr	2.0	2.1

Suppl. Tab. 3: Extended photophysical data of the investigated molecules in toluene solutions. τ_{eff} (effective lifetime) is defined as an intensity-weighted average of decay times:

$$\tau_{\text{eff}} = \frac{\sum_i A_i \tau_i^2}{\sum_i A_i \tau_i} = \sum_i f_i \tau_i$$

where A_i, τ_i and f_i are the amplitudes, lifetimes, and normalised weights of individual decay components, respectively.

Material	Φ_{PL} (%)	Φ_1 (%)	τ_1 (ns)	k_{r1} (10^6 s^{-1})	k_{nr1} (10^6 s^{-1})	Φ_2 (%)	τ_2 (ns)	k_{r2} (10^6 s^{-1})	k_{nr2} (10^6 s^{-1})	τ_{eff} (ns)
An-T-1Cz	8	7	15.6	4.6	64.1	1	437	0.1	2.3	68
An-T-3PCz	3	3	15.8	1.8	63.3	0.5	729	0.1	1.4	140
T-3Cz-An	55	3	31.6	1.0	31.6	52	1030	0.5	1.0	980
T-3Cz-Acr	50	18	31.8	5.7	31.4	32	216	1.5	4.6	150

Suppl. Tab. 4: Extended photophysical data of the investigated in 0.5% in PMMA films.

Material	Φ_{PL} (%)	Φ_1 (%)	τ_1 (ns)	k_{r1} (10^6 s^{-1})	k_{nr1} (10^6 s^{-1})	Φ_2 (%)	τ_2 (ns)	k_{r2} (10^6 s^{-1})	k_{nr2} (10^6 s^{-1})	τ_{eff} (ns)
An-T-1Cz	1	1	10	0.5	100	0.05	986	0.1	1.0	59
An-T-3PCz	6	5	9	0.6	111	1	221	0.1	4.5	44
T-3Cz-An	50	17	51	3.3	20	34	2640	0.1	0.4	1800
T-3Cz-Acr	58	24	43	5.6	23	35	3830	0.1	0.3	2300

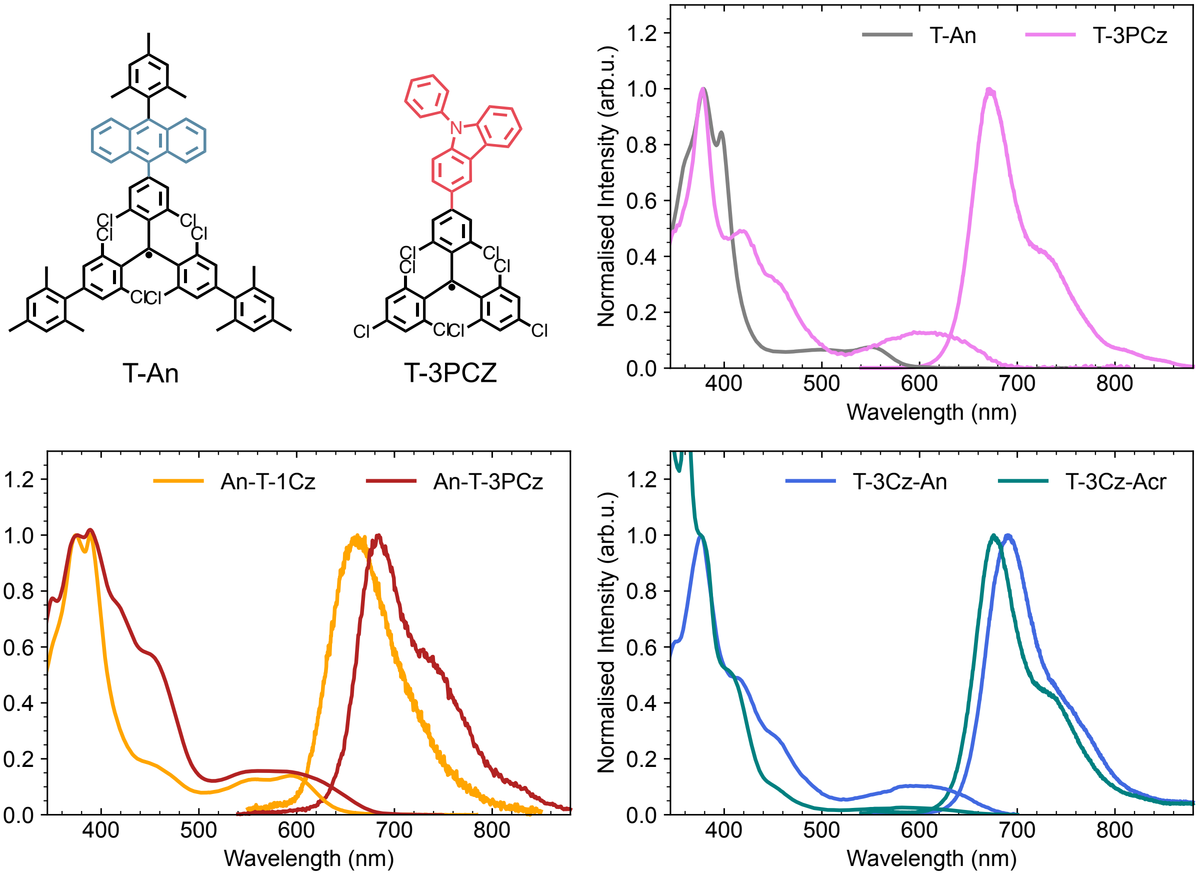

Suppl. Fig. 11: Structures of control molecules, T-An and T-3PCz (top left). Steady-state absorption and emission spectra of control molecules (top right), and the main investigated dyads (bottom panels). Spectra were recorded in 0.1 mM toluene solutions at 292 K. Emission profiles were obtained after exciting near the absorption edge, with fluences below 6 μJ/cm^2.

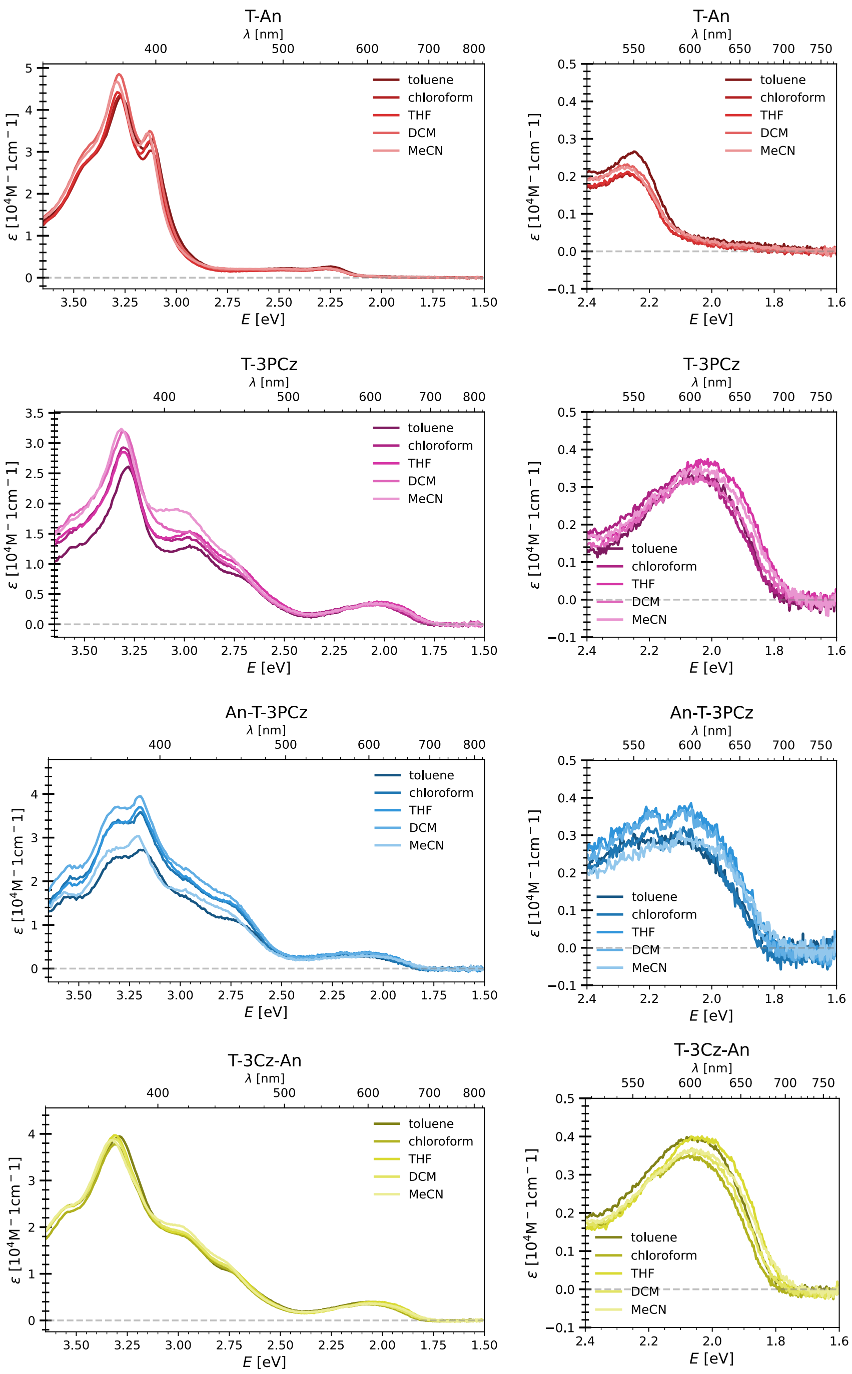

Suppl. Fig. 12: Steady-state absorption spectra for the solvent series (0.1 mM) of T-An, T-3PCz, An-T-3PCz and T-3Cz-An, measured using an integrating sphere at 292 K. In T-An, the band at 2.25 eV we assign to the $^2LE_{TTM}$ transition. The $^2CT_{Ac}$ transition has vanishing oscillator strength and is low in energy, as predicted by the TD-DFT calculations and validated by PDS (*vide infra*). In T-An, we assign it to a long weak tail spanning the energy range between 2.1 eV and 1.7 eV. In other molecules, the $^2CT_{CT}$ transition is bright.

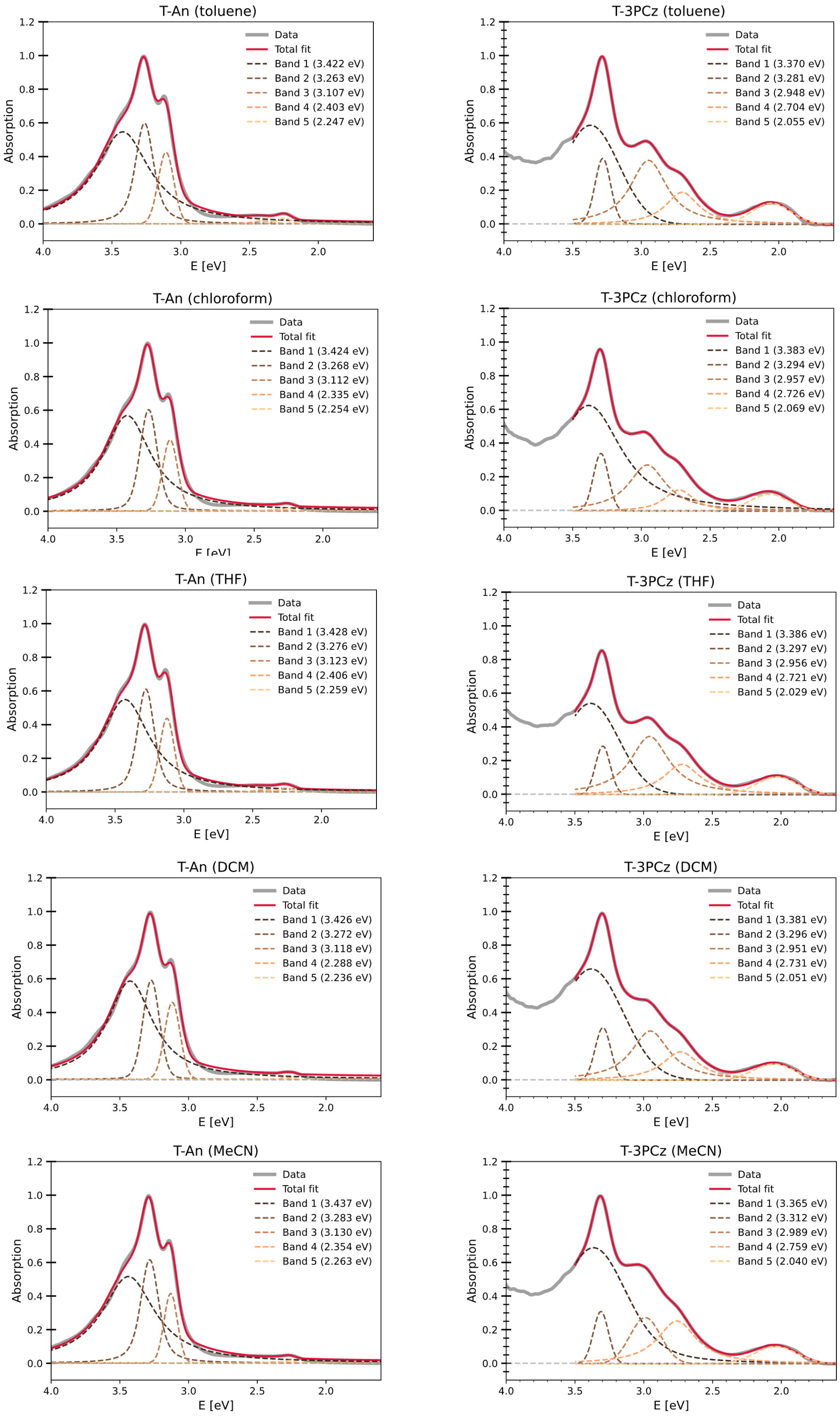

Suppl. Fig. 13: Voigt deconvolution of the absorption spectra for the solvent series of T-An and T-3PCz from Suppl. Fig. 12.

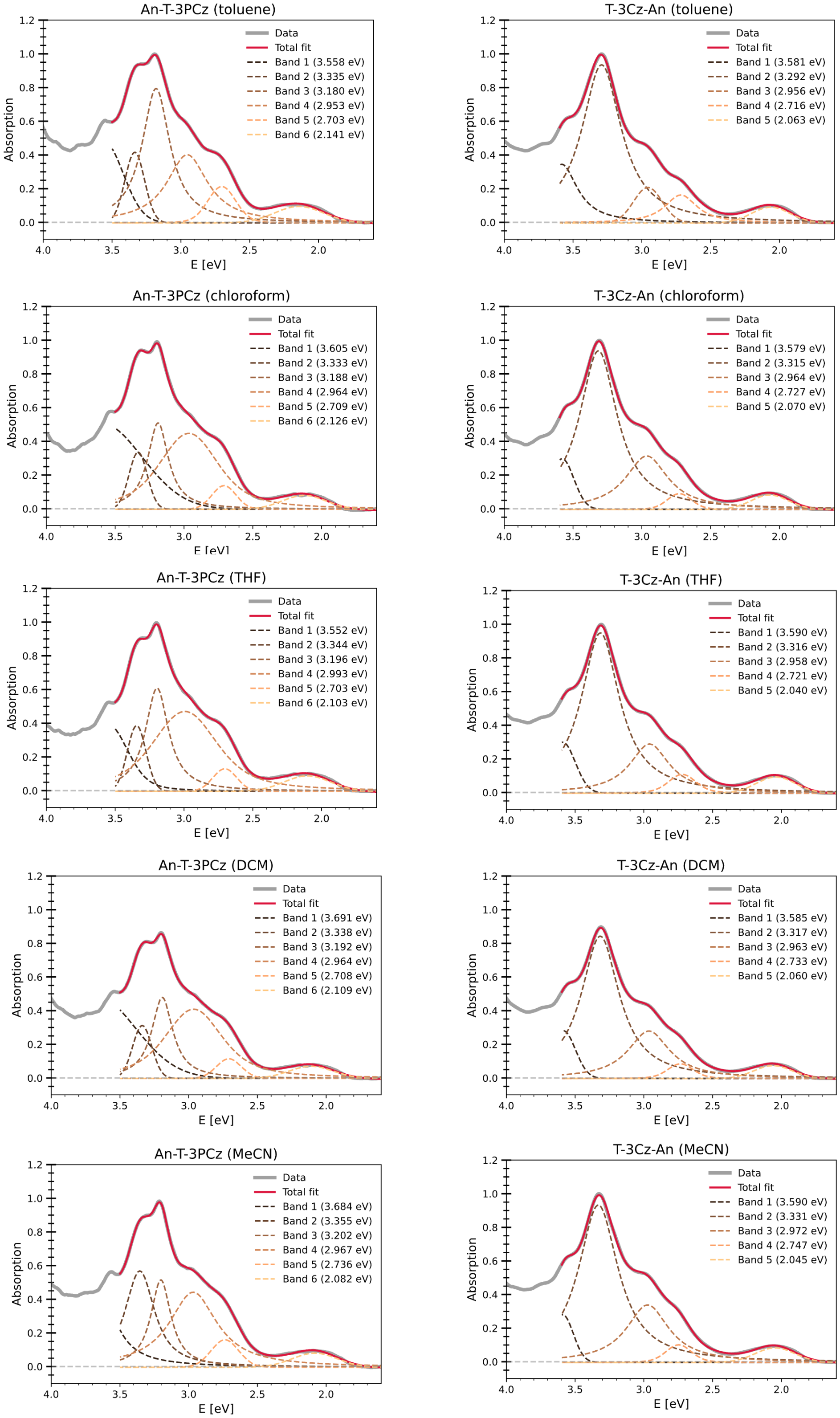

Suppl. Fig. 14: Voigt deconvolution of the absorption spectra for the solvent series of An-T-3PCz and T-3Cz-An from Suppl. Fig. 12.

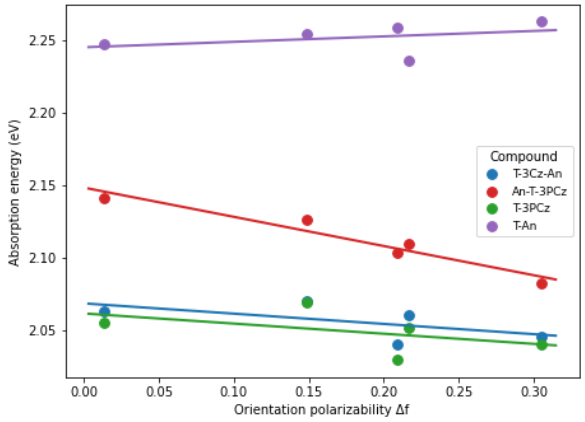

Suppl. Fig. 15: Lippert plot of lowest absorption band peak as a function of solvent polarizability. Band peaks are extracted from deconvolved bands obtained from Suppl. Fig. 13 and 14.

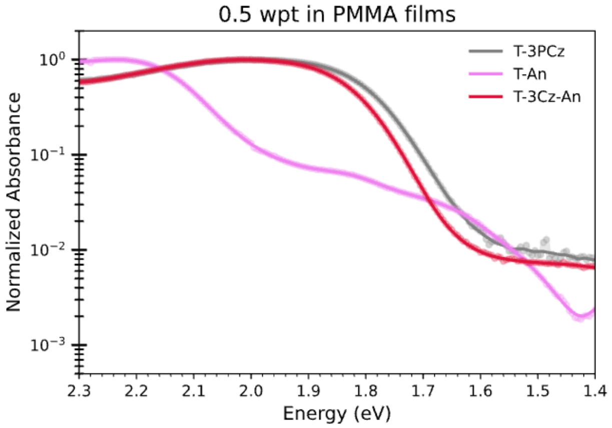

Suppl. Fig. 16: Photothermal deflection spectroscopy (PDS) of 0.5% in PMMA films.

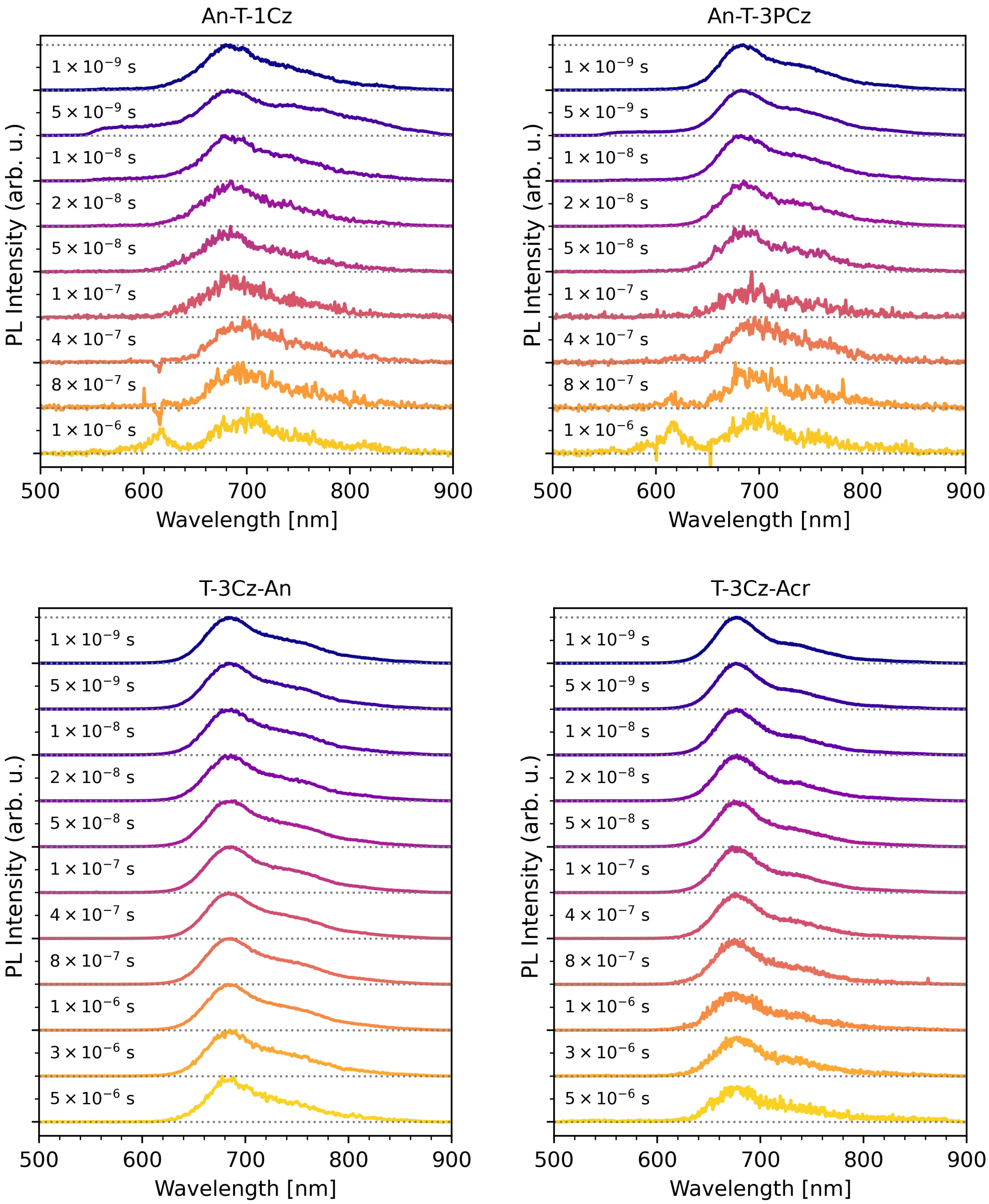

Suppl. Fig. 17: Time-resolved emission spectra recorded in 100 μM toluene solutions at 292 K. T-3Cz-Acr was excited using a 520 nm laser, while the remaining samples were excited with a 590 nm laser, with average fluences ranging from 0.8 to 6 μJ/cm^2.

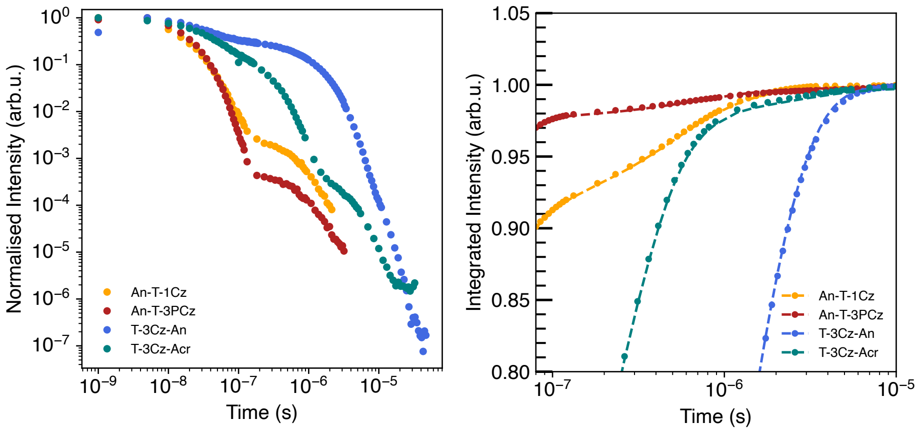

Suppl. Fig. 18: Emission traces (left) and integrated emission traces, zoomed in to show the delayed component (right) (100 µM, toluene, 292 K).

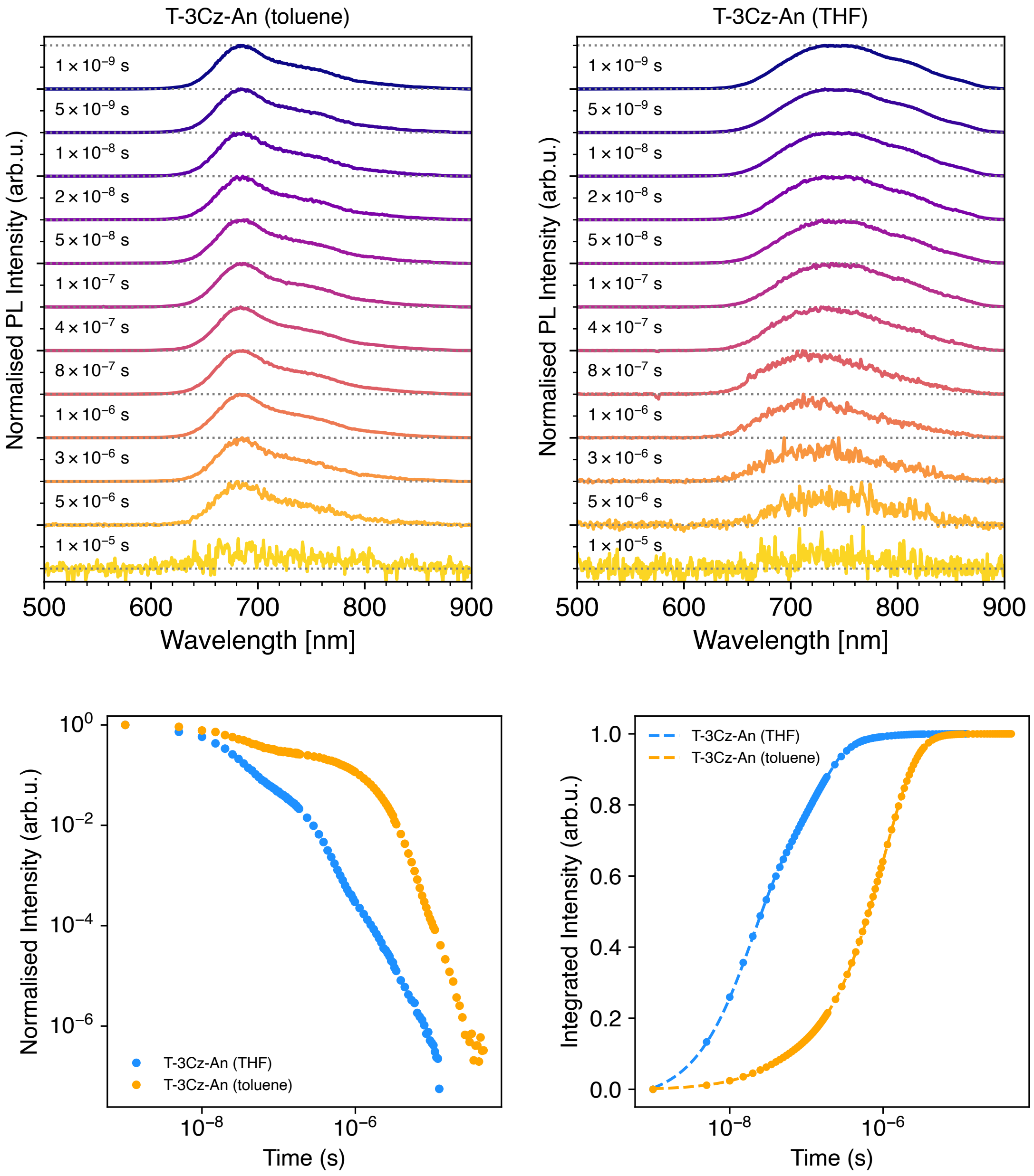

Solvent	Lifetime [ns] (weight)	Effective lifetime [ns]
Toluene ($\epsilon = 2.4$)	31.2 (6%)<br>1028 (94%)	968
THF ($\epsilon = 7.43$)	16.7 (55%)<br>136 (45%)	70

Suppl. Fig. 19: Time-resolved emission of T-3Cz-An in toluene and tetrahydrofuran (THF) recorded under 2 μJ/cm^2 590 nm excitation of 200 μM solutions at 292 K.

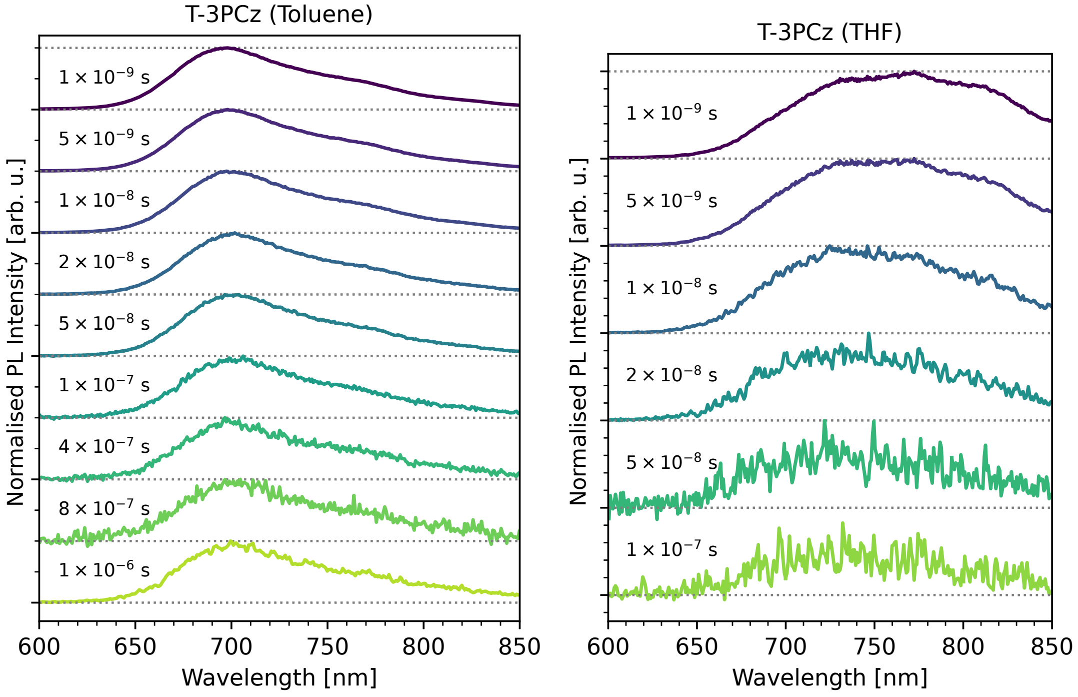

Suppl. Fig. 20: Time-resolved PL of the 0.1 mM T-3PCz solution in toluene (left) and THF (right) following 490 nm excitation at 292 K. Significant broadening and red shift of the emission profile in polar solvents is typical for TTM-type D_1 emission. We also observe small conformation-dependent blue shifts at late times.

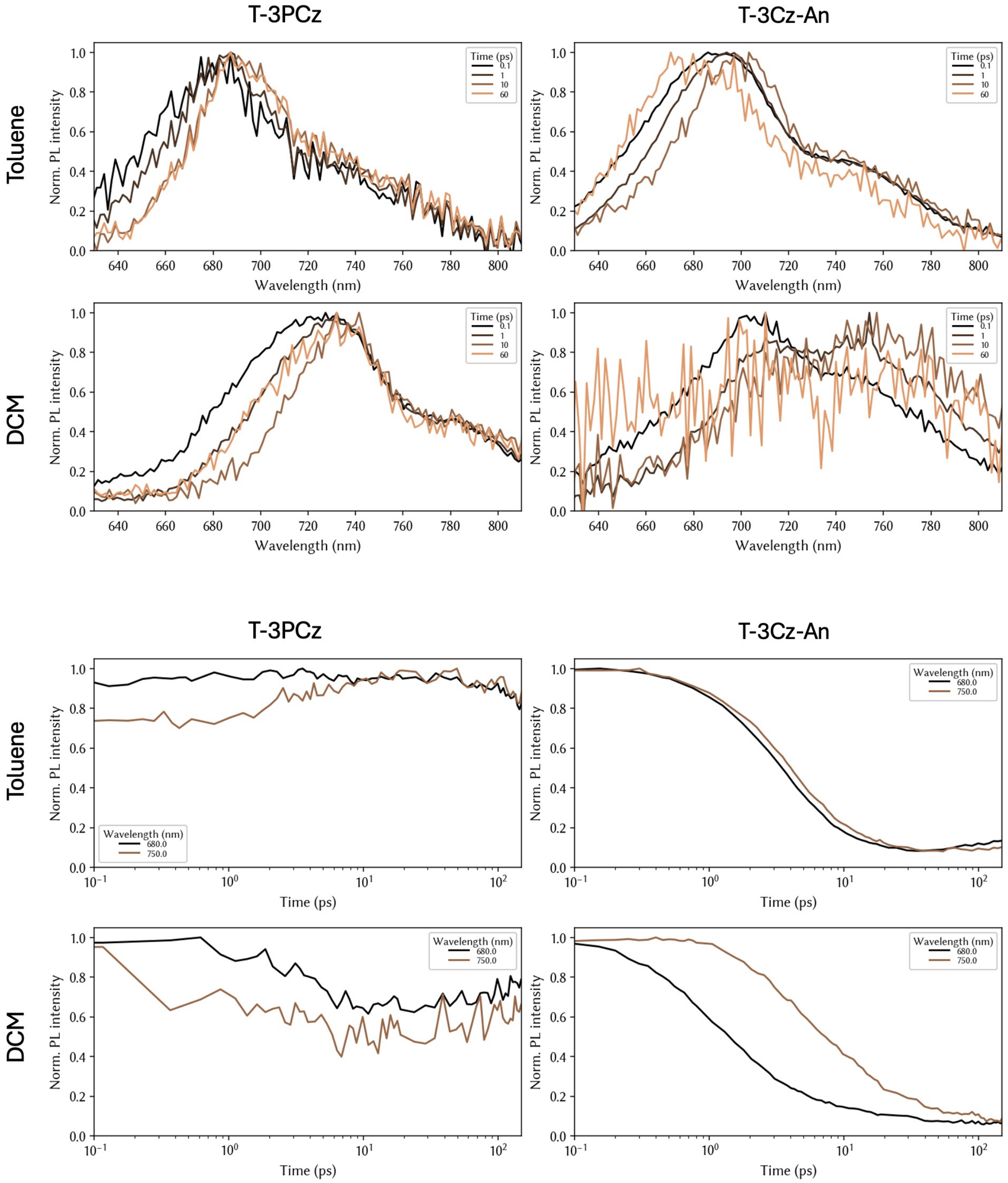

Suppl. Fig. 21: Kerr-gated transient PL spectra (top) and kinetics (bottom) of 0.1 mM toluene and DCM solutions of TTM-3PCz and T-3Cz-An following 460 nm excitation at 292 K.

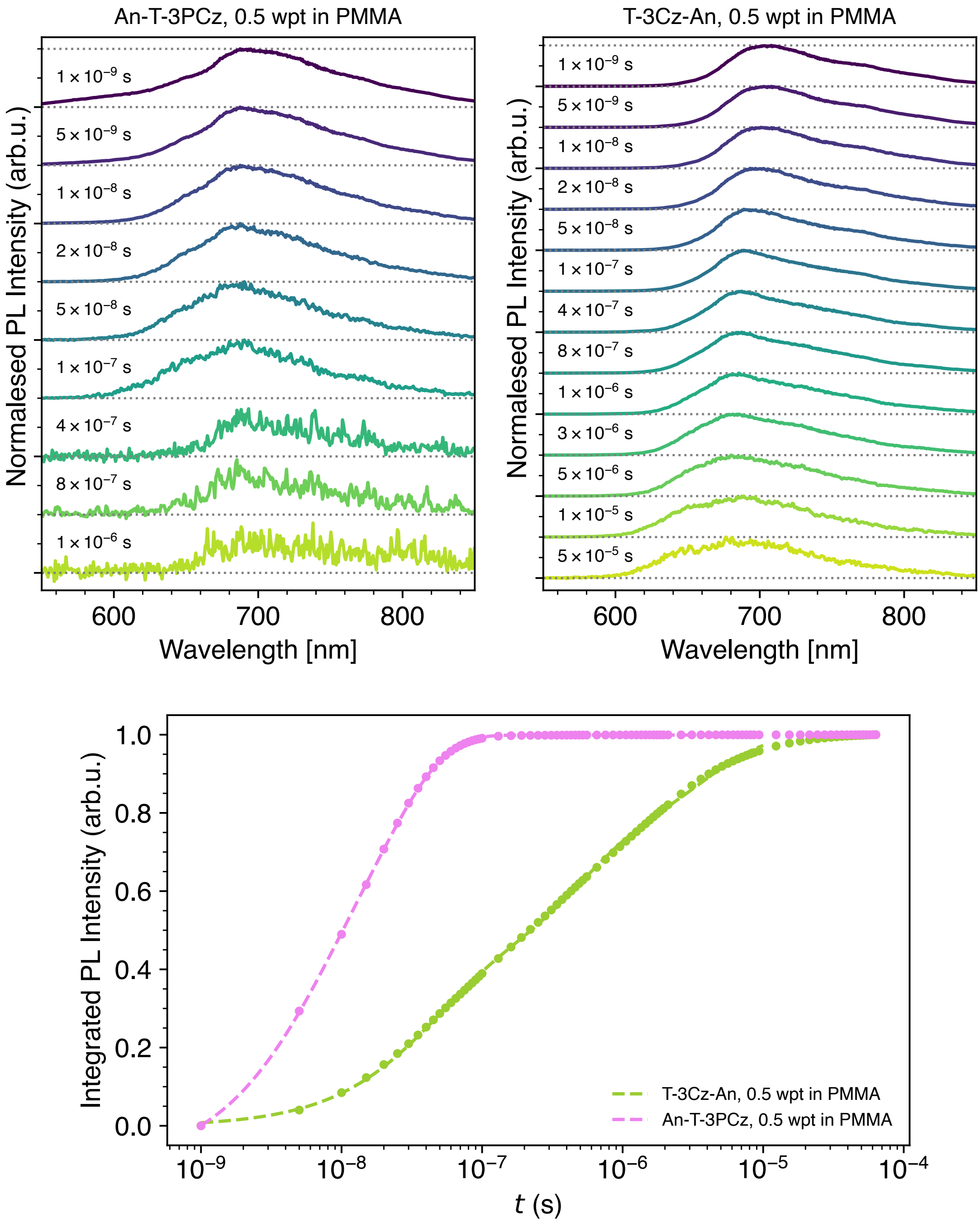

Suppl. Fig. 22: Time-resolved PL on 0.5% in PMMA films of An-T-3PCz and T-3Cz-An following 490 nm excitation at 292 K.

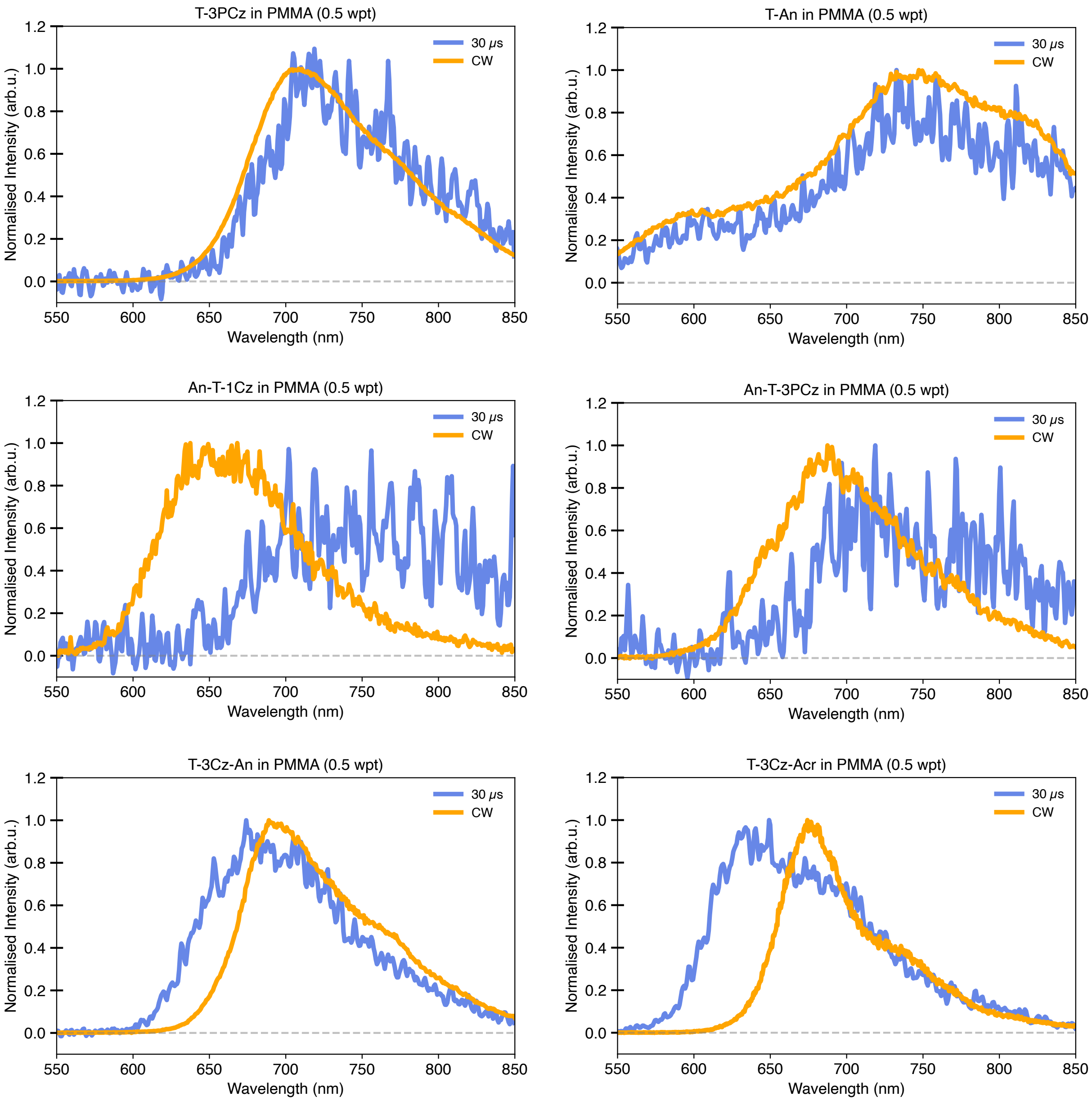

Suppl. Fig. 23: Steady-state and trailing emission profiles of each compound in 0.5% in PMMA films following 490 nm excitation at 292 K.

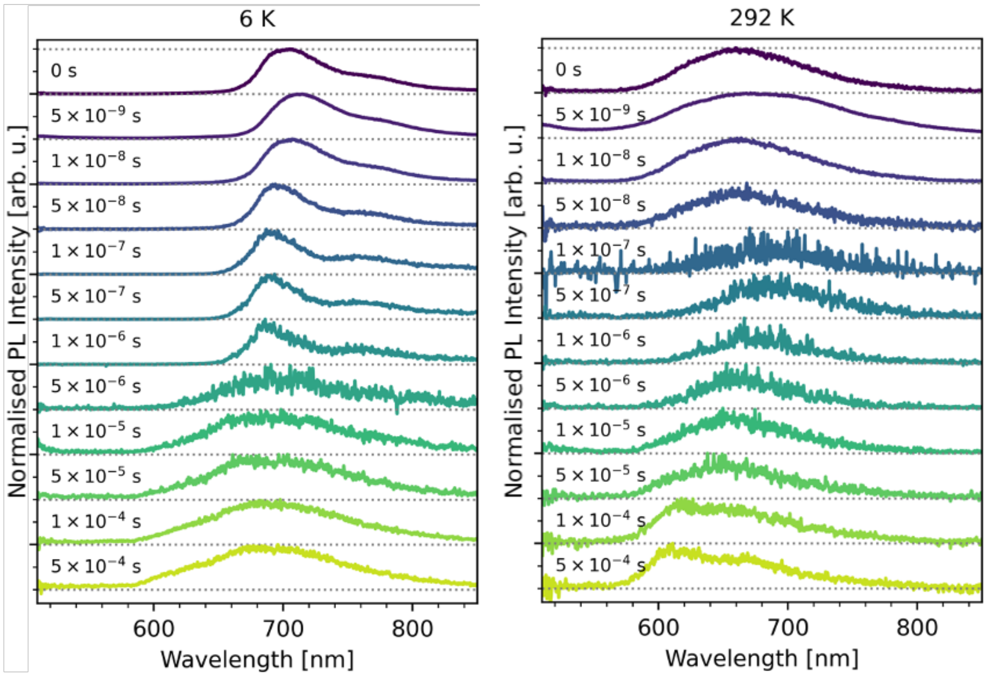

Suppl. Fig. 24: Photoluminescence profiles of An-T-1Cz (5 weight percentage in PMMA) thin film, following 532 nm excitation at the lowest, 6 K, and the highest, 292 K, measured temperatures.

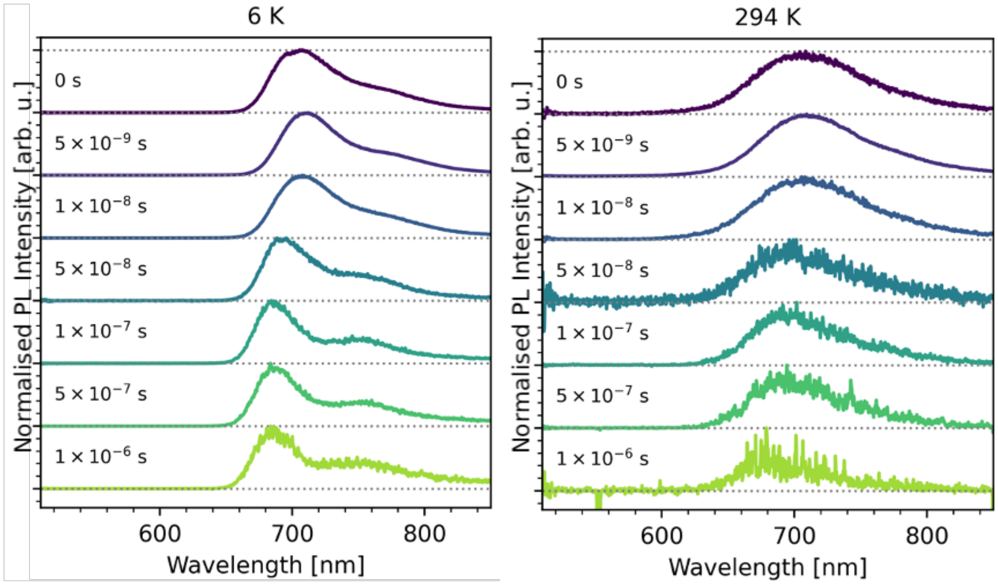

Suppl. Fig. 25: Photoluminescence profiles of An-T-3PCz (5 weight percentage in PMMA) thin film, following 532 nm excitation at the lowest, 6 K, and the highest, 294 K, measured temperatures.

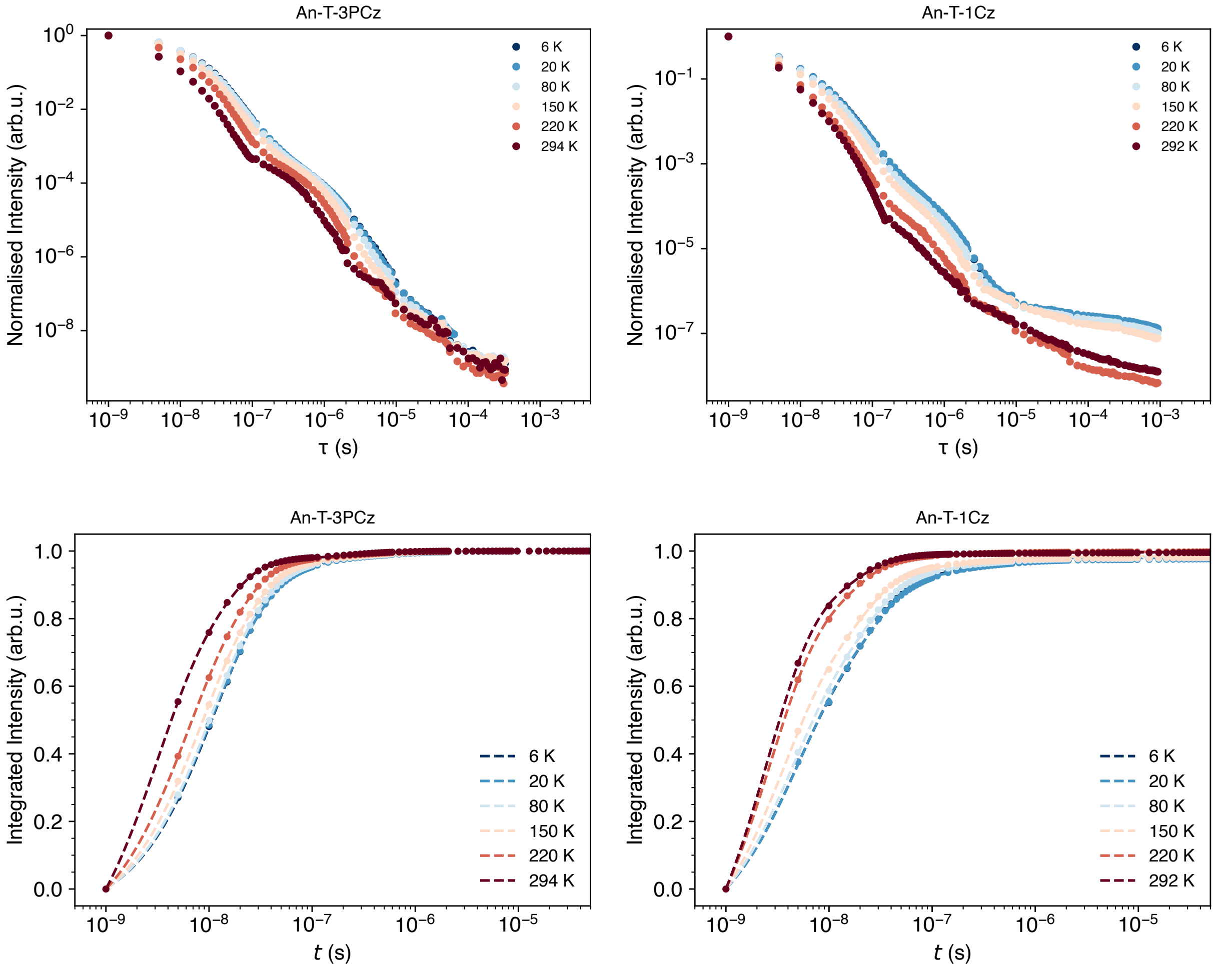

Suppl. Fig. 26: Raw (top) and integrated (middle, bottom) emission kinetics of 5% radical in PMMA thin films of An-T-3PCz (left), An-T-1Cz (right) following 532 nm excitation. The bottom panels show zoomed-in integrated kinetic traces, highlighting temperature dependence of delayed emission.

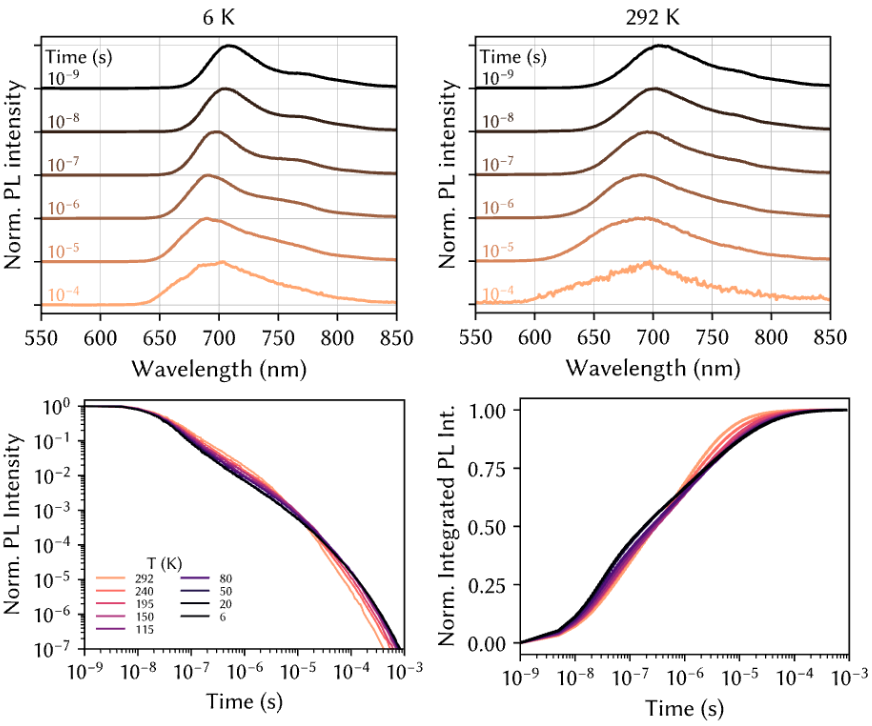

Suppl. Fig. 27: Temperature-dependent emission profile (top) and kinetics (bottom) of 5% T-3Cz-An in PMMA thin film following 532 nm excitation.

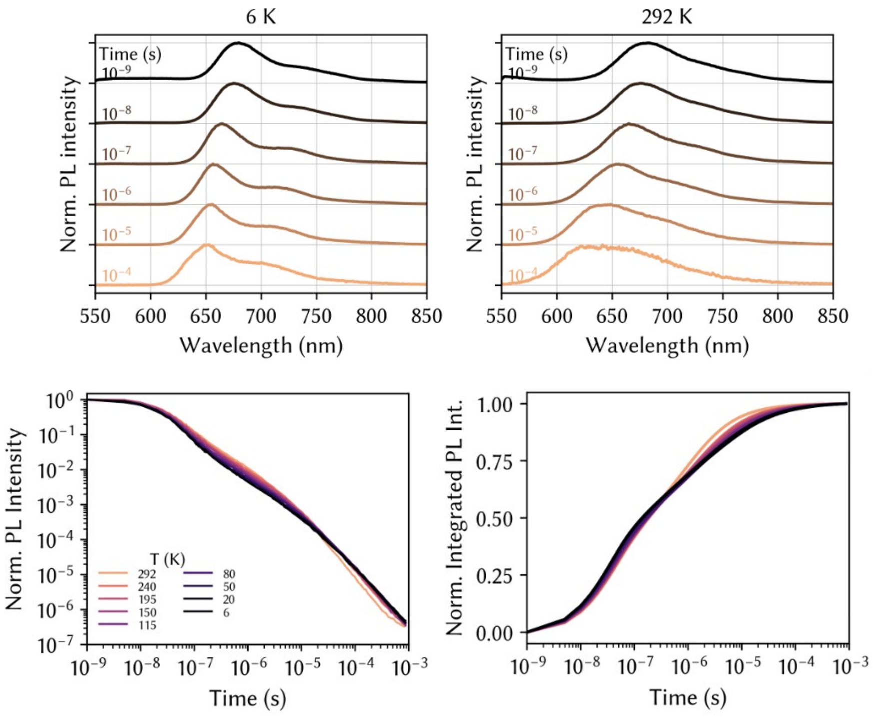

Suppl. Fig. 28: Temperature-dependent emission profile (top) and kinetics (bottom) of 5% T-3Cz-Acr in PMMA thin film following 532 nm excitation.

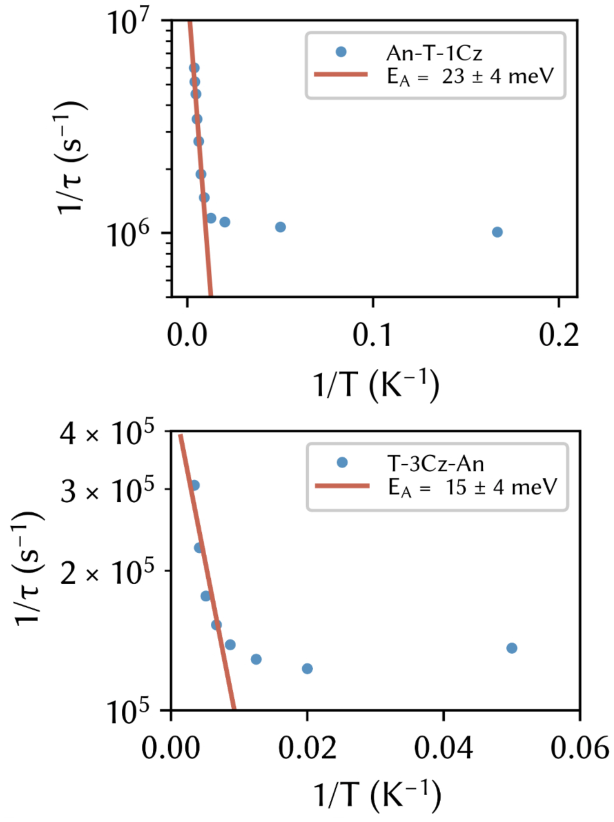

Suppl. Fig. 29: Arrhenius analysis of temperature-dependent transient photoluminescence data in Suppl. Fig. 24-28.

Suppl. Tab. 5: Fitted lifetimes and weights for temperature dependent trPL measurements of 5% in PMMA films.

	**An-T-1Cz**				**An-T-3PCz**				**T-3Cz-An**				**T-3Cz-Acr**			
Temp (K)	τ_1 (ns)	w_1 (%)	τ_2 (ns)	w_2 (%)	τ_1 (ns)	w_1 (%)	τ_2 (ns)	w_2 (%)	τ_1 (ns)	w_1 (%)	τ_2 (ns)	w_2 (%)	τ_1 (ns)	w_1 (%)	τ_2 (ns)	w_2 (%)
6	19	95	400	5	21	96	374	4	35	42	7320	58	33	48	9290	52
20	20	95	443	5	20	95	327	5	36	43	7240	57	33	48	8680	52
50	–	–	–	–	–	–	–	–	37	42	6850	58	34	47	8280	53
80	18	96	405	4	20	96	359	4	39	39	6720	61	34	45	8190	55
115	–	–	–	–	–	–	–	–	42	38	6110	62	37	43	7130	57
150	15	97	430	3	16	96	292	4	45	36	5640	64	38	42	6390	58
195	–	–	–	–	–	–	–	–	47	36	4780	64	40	42	5780	58
220	10	99	235	1	12	97	252	3	–	–	–	–	–	–	–	–
240	–	–	–	–	–	–	–	–	49	36	3760	64	43	41	5390	59
292	10	99	986	1	9	98	221	2	51	33	2640	67	43	41	3830	59

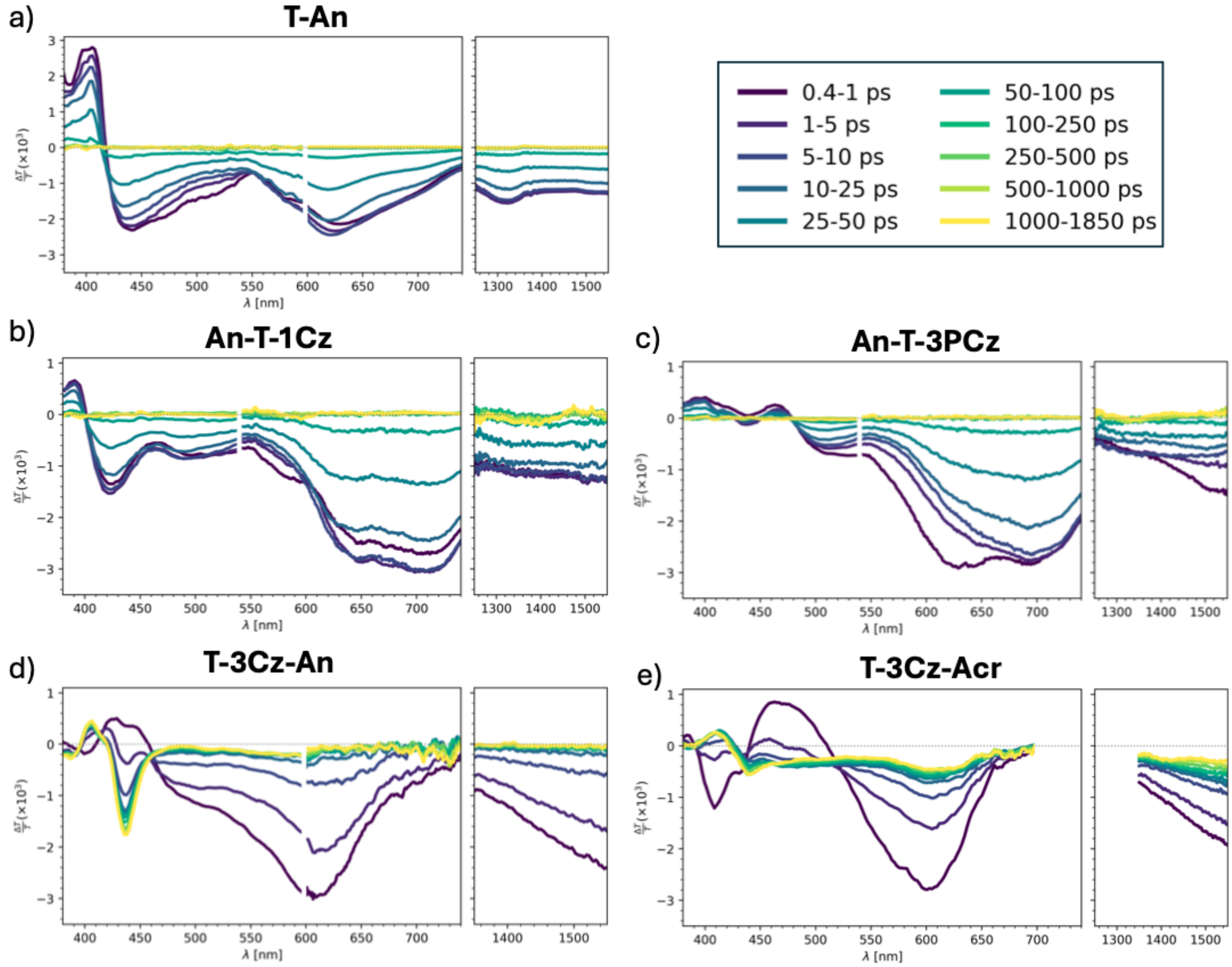

Suppl. Fig. 30: Picosecond transient absorption spectra for 0.1 mM toluene solutions of a) T-An, b) An-T-1Cz, c) An-T-3PCz, d) T-3Cz-An and e) T-3Cz-Acr. T-An was measured under 532 nm excitation, while the other materials were excited with 600 nm laser pulse, under moderate fluence (4 to 6 μJ/cm^2). Panels share the same time scale.

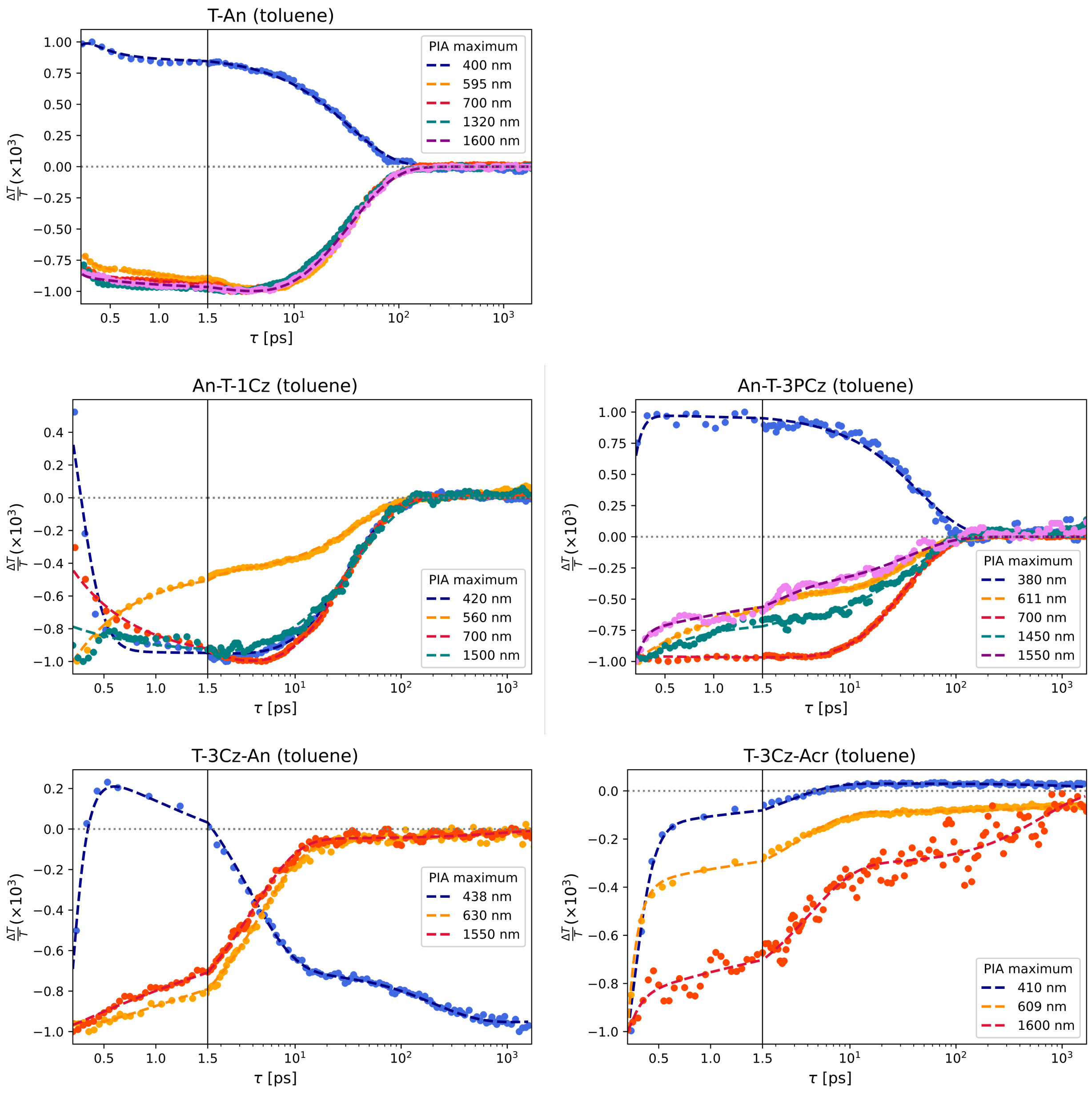

Suppl. Fig. 31: Kinetic traces extracted from picosecond transient absorption spectra of investigated materials in toluene, reported in Figure 30. Fitting parameters are reported in Table 6.

Suppl. Tab. 6: Fitting parameters of 0.1 mM toluene TA datasets.

System	λ (nm)	Comp.	τ [ps]
T-An	400	1	34.8 ± 0.1
	595	1	3.5 ± 0.1
		2	32.7 ± 0.1
	700	1	2.7 ± 0.3
		2	32.6 ± 3.4
	1320	1	3.6 ± 3.0
		2	32.8 ± 3.9
	1600	1	3.8 ± 0.9
		2	34.4 ± 5.5
An-T-1Cz	420	1	5.5 ± 2.9
		2	30.0 ± 3.0
	560	1	0.56 ± 0.2
		2	38.1 ± 4.1
	700	1	0.9 ± 0.6
		2	4.0 ± 1.3
		3	30.3 ± 1.4
	1500	1	0.9 ± 0.2
		2	39.5 ± 2.5
An-T-3PCz	380	1	45.1 ± 2.4
	610	1	0.95 ± 0.2
		2	8.9 ± 1.7
		3	28.6 ± 4.3
	700	1	5.7 ± 1.1
		2	30.0 ± 4.5
	1450	1	0.8 ± 0.4
		2	36.8 ± 2.2
	1550	1	1.6 ± 0.4
		2	35.8 ± 2.1
T-3Cz-An	438	1	3.7 ± 0.8
		2	>5e3
		3	>5e3
	630	1	4.6 ± 0.4
		2	>5e3
	1550	1	3.9 ± 0.6
		2	>5e3
T-3Cz-Acr	410	1	2.5 ± 0.1
		2	>5e3
	650	1	3.3 ± 0.9
		2	>5e3
	1600	1	4.2 ± 3.6
		2	>5e3

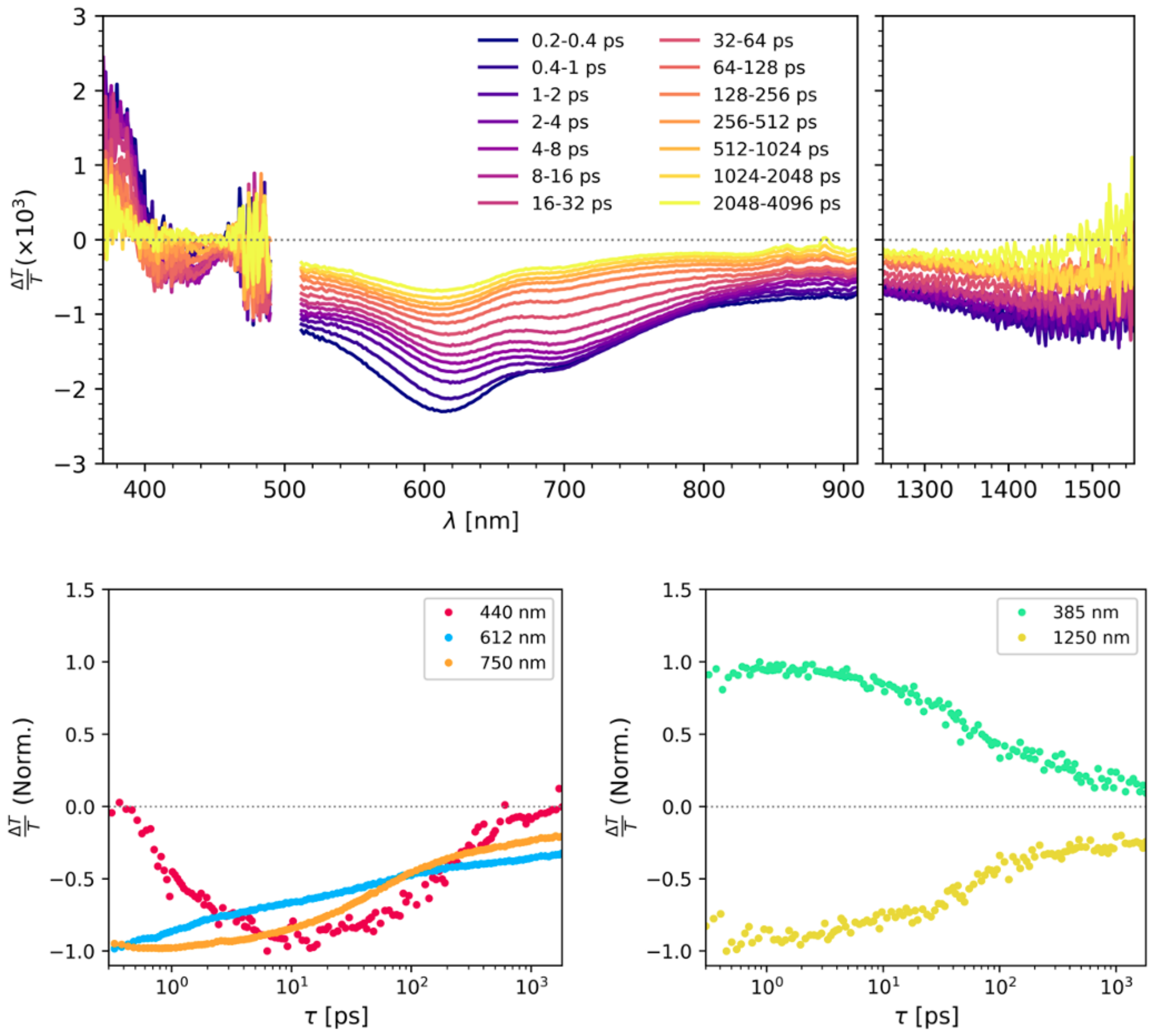

Suppl. Fig. 32: Dynamics of materials with strong radical-acene electronic coupling in rigid media. Top: Picosecond transient absorption spectral slices of An-T-3PCz thin film (5 weight per cent in PMMA), following a 20 μJ/cm^2 530 nm excitation. Bottom: extracted kinetic traces. We observe two correlated pairs of signals (blue/orange and green/yellow) and a PIA at 440 nm with a maximum around 10 ps.

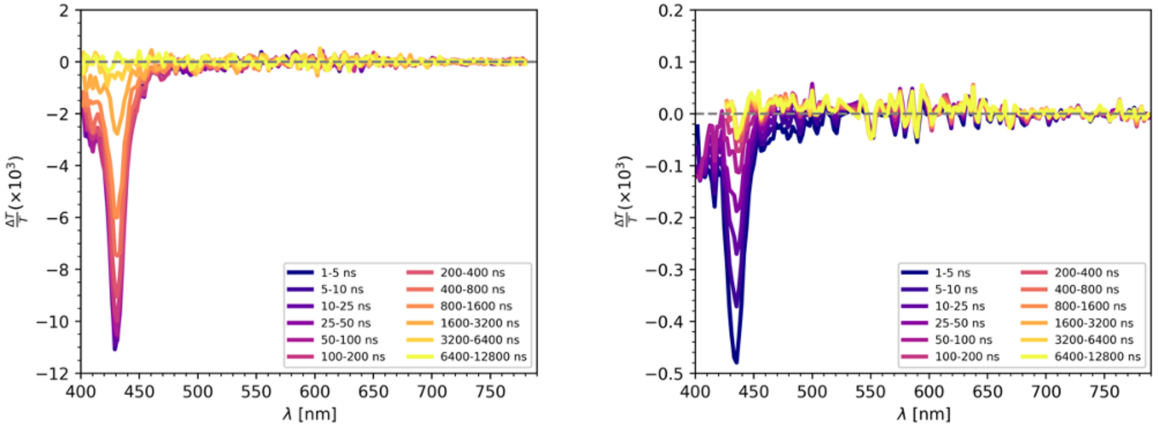

Suppl. Fig. 33: Nanosecond TA spectra of T-3Cz-An in (left) toluene and (right) dichloromethane (DCM) solutions (200 µM) under 532 nm excitation.

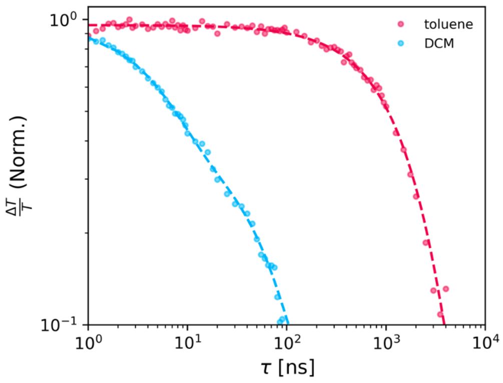

Solvent	Lifetime [ns] (weight)	Effective lifetime [ns]
Toluene ($\epsilon = 2.4$)	1550 (100%)	1550
DCM ($\epsilon = 8.9$)	5.3 (60%)<br>56.7 (39%)	25

Suppl. Fig. 34: Anthracene triplet PIA kinetics at 430 nm with corresponding mono- (toluene) and bi-exponential (DCM) fits, together with extracted lifetimes.

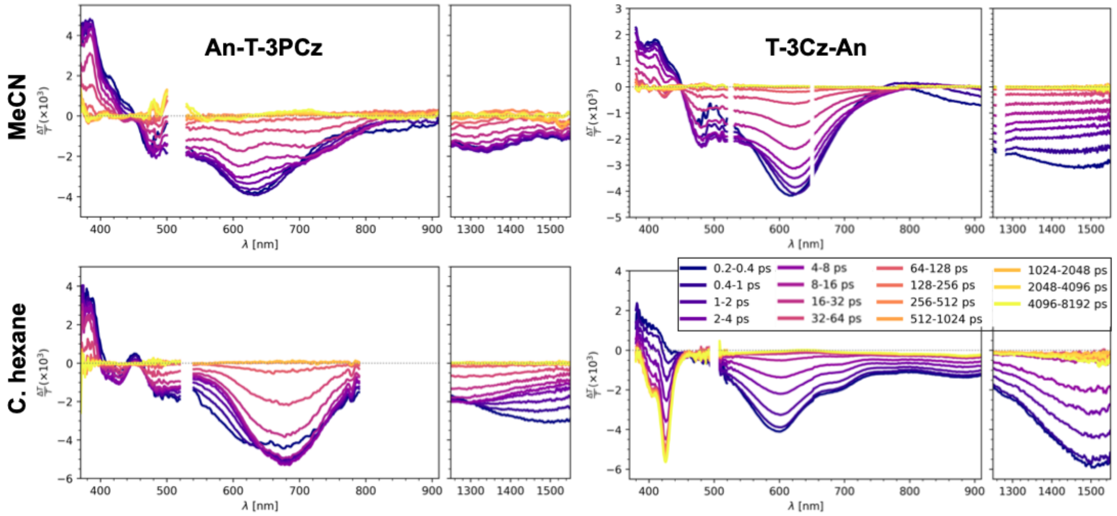

Suppl. Fig. 35: Solvatochromism in ps TA under 530 nm excitation, concentration of 0.1 mM for each solvent (cyclohexane, $\epsilon = 2.02$, acetonitrile $\epsilon = 37.5$). Time slices are the same for every panel. The 430 nm PIA characteristic of anthracene triplet is absent in a more polar environment indicating that $^{2}CT_{Cz}$ stabilisation below the $^{2,4}LE_{Ac}$ leads to quenching of the delayed component.

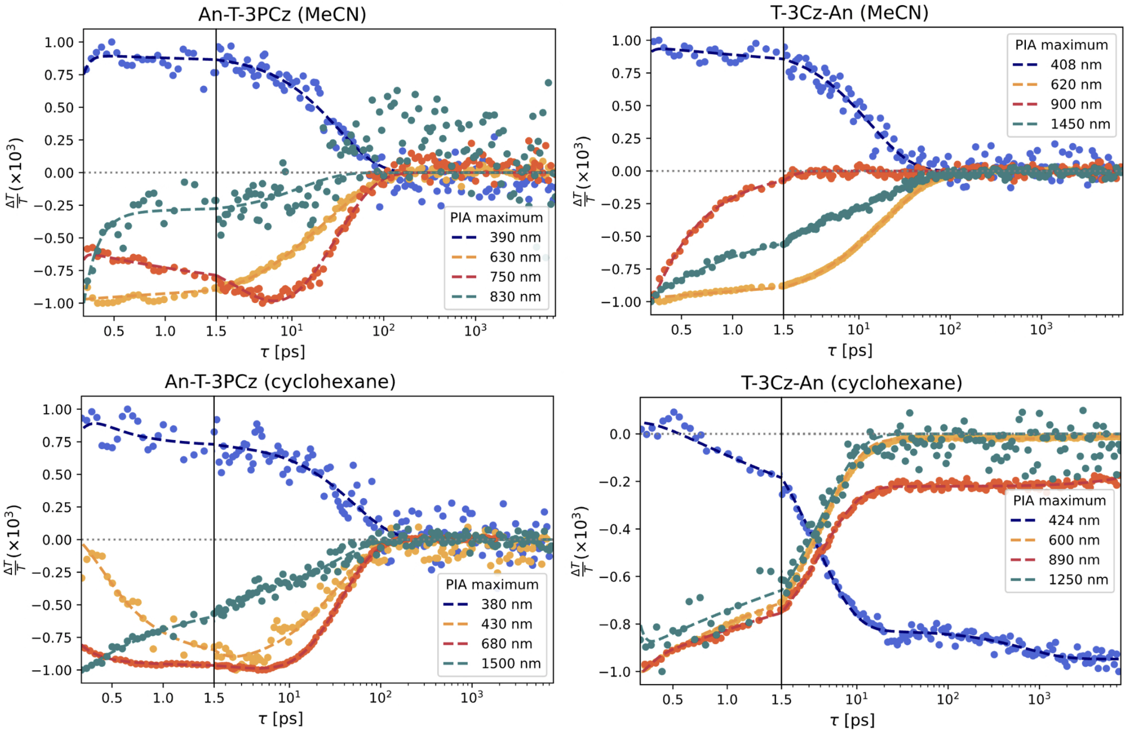

Suppl. Fig. 36: Kinetic traces extracted from picosecond transient absorption spectra of T-3Cz-An and An-T-3PCz in cyclohexane and acetonitrile (MeCN), reported in Figure 35. Fitting parameters are reported in Table 7.

Suppl. Tab. 7: Fitting parameters of 0.1 mM MeCN and cyclohexane TA datasets.

System	λ (nm)	Comp.	τ [ps]
An-T-3PCz, Cyclohexane	390	1	49.6 ± 6.8
	430	1	34.0 ± 3.4
	680	1	7.5 ± 3.2
		2	31.2 ± 4.0
	1500	1	31.7 ± 4.0
An-T-3PCz, MeCN	390	1	32.1 ± 3.4
	420	1	7.4 ± 1.8
	630	1	7.4 ± 3.0
		2	36.4 ± 5.6
	750	1	3.5 ± 0.7
		2	30.3 ± 2.6
	830	1	12.7 ± 11.0
T-3Cz-An, C.hexane	424	1	3.7 ± 1.1
		2	>5e3
	600	1	4.2 ± 0.3
		2	>5e3
	890	1	4.0 ± 0.6
		2	>5e3
	1250	1	4.1 ± 2.4
T-3Cz-An, MeCN	408	1	7.8 ± 0.4
		2	14.8 ± 2.8
	620	1	6.5 ± 5.0
		2	28.5 ± 7.5
	900	1	0.5 ± 0.1
	1450	1	1.0 ± 0.2
		2	22.9 ± 6.1

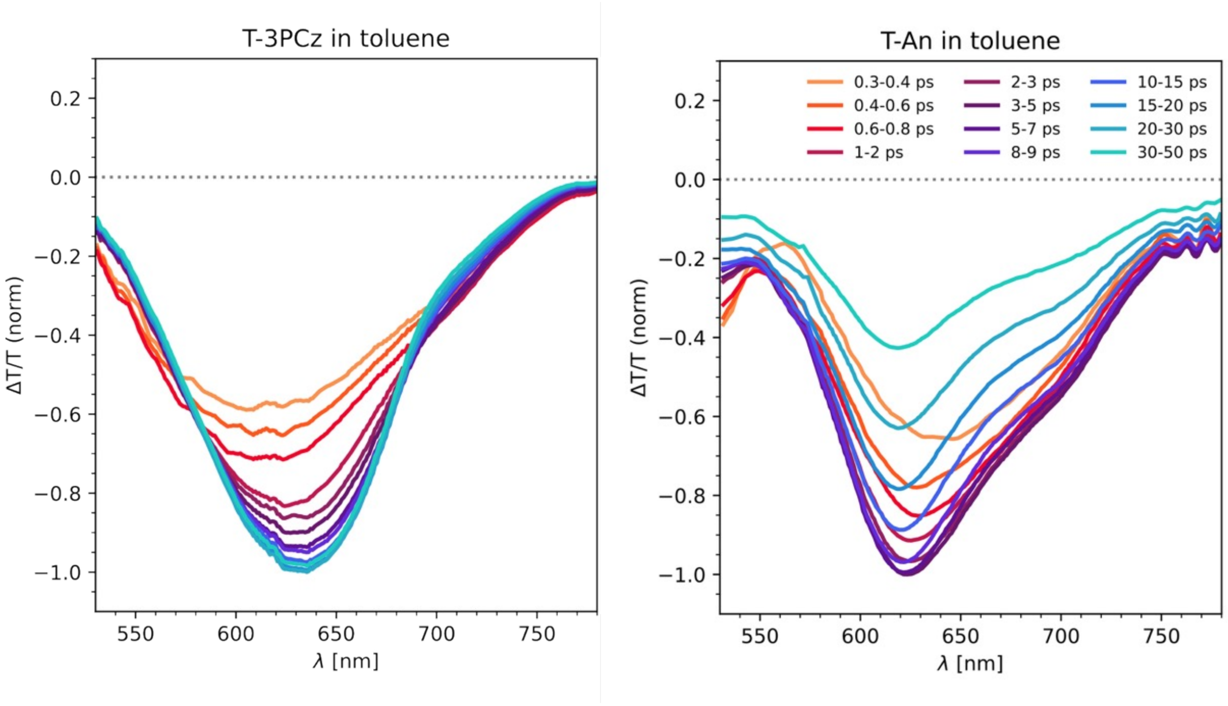

Suppl. Fig. 37: Early time spectral profile of 0.1 mM T-3PCz (left), and 0.2 mM T-An (right) in toluene, following 532 nm excitation. Time slices are identical across the panels.

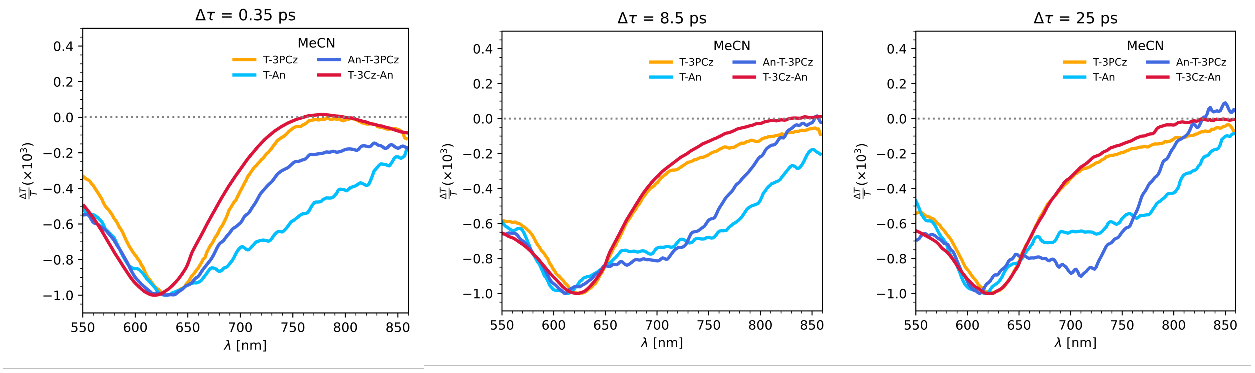

Suppl. Fig. 38: Normalised early time TA spectra of T-3PCz, T-An, An-T-3PCz and T-3Cz-An in MeCN. Presented slices are at 0.35 ps (left), 8.5 ps (middle), and 25 ps (right) following 490 nm photoexcitation.

Electron Spin Resonance

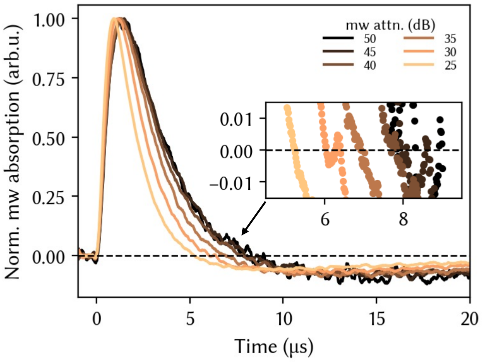

Suppl. Fig. 39: Microwave power dependence on recorded X-band trESR of T-3Cz-An at 80 K. Monitored at field of 328 mT, corresponding to the maximum of the Y polarisation.

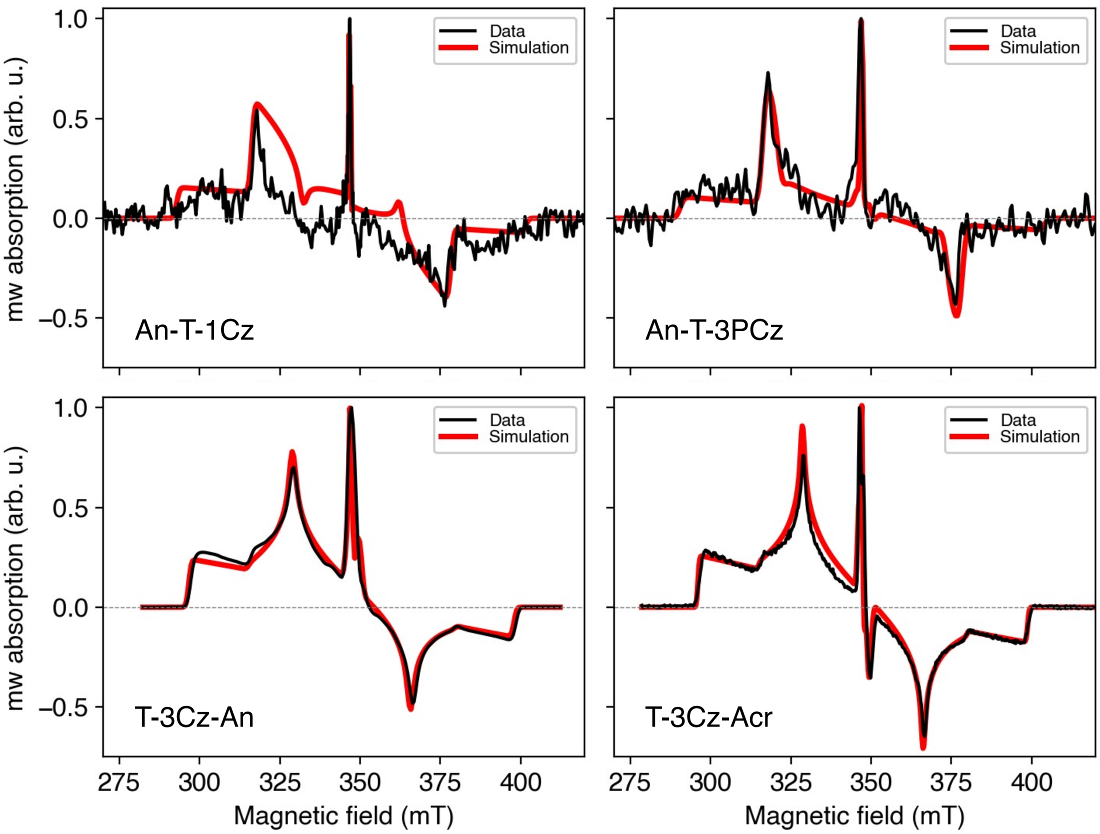

Suppl. Fig. 40: Simulations to the prompt (0.5-1.5 μs) X-band transient ESR spectra on 100 μM frozen toluene solutions at 80 K, acquired after 600 nm 1 mJ pulses. The same data is presented in the main text Fig. 3a,b.

Suppl. Tab. 8: Spin Hamiltonian parameters and populations found from trESR simulations.

Parameter	An-T-1Cz	An-T-3PCz	T-3Cz-An	T-3Cz-Acr
g_T, g_R	2.003, 2.004	2.003, 2.004	2.003, 2.004	2.003, 2.004
D_T, E_T (MHz)	2487, -100	2415, 0	2150, -203	2169, -201
r_{RT} (nm)	0.72	0.72	1.01	1.02
ϕ_{RT}, θ_{RT} (°)	91, 90	68, 61	35, 115	33, 114
W_R	0.40	0.55	0.55	0.31
D_β, D_α	0.522, 0.478	0.513, 0.487	0.529, 0.471	0.509, 0.491
T_z, T_y, T_x	0.37, 0.36, 0.27	0.37, 0.38, 0.25	0.41, 0.26, 0.33	0.39, 0.28, 0.33

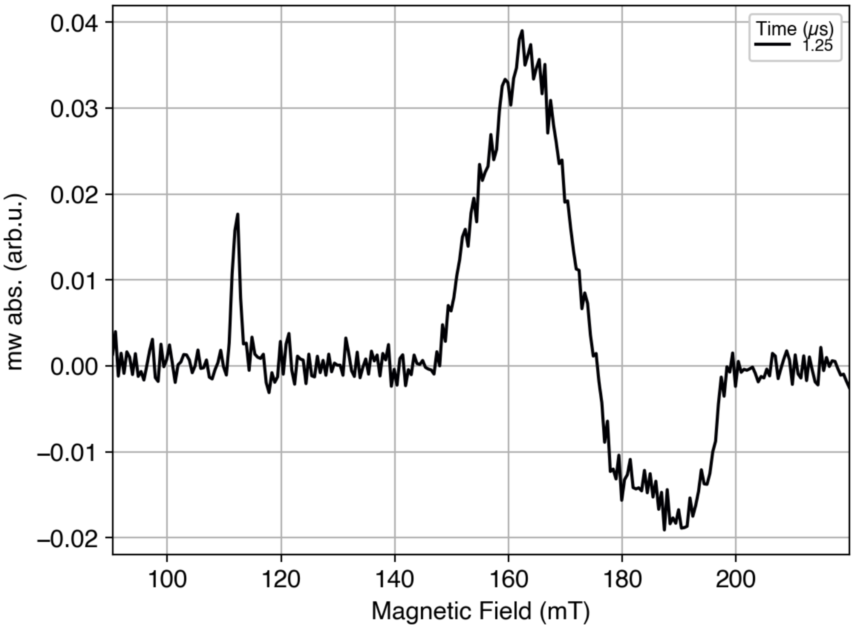

Suppl. Fig. 41: X-band Half Field transient ESR spectrum on 100 μM frozen toluene solution of T-3Cz-An at 80 K, acquired 1-2 μs after 450 nm 1 mJ pulses. Narrow $\Delta m_s = 3$ and broad $\Delta m_s = 2$ transitions observed.

```
Sys.S = [1 1/2];
Sys.g = [g_T; g_R];
Sys.D = [D_T E_T; 0 0];
Sys.J = -1e5;
Sys.lwpp = 1.2;
D_RT = (mu0/(4*pi)) * (g_T * g_R * muB^2 * 1e-6) / (h*r_RT^3);
Sys.dip = [-D_RT/2, -D_RT/2, D_RT];
Sys.eeFrame = [phi_RT theta_RT 0] * pi/180;

Sx_T = sop(Sys, 'x1'); Sy_T = sop(Sys, 'y1'); Sz_T = sop(Sys, 'z1');
Sz_R = sop(Sys, 'z2'); Id = eye(size(Sx_T));
P_Tx = Id - Sx_T^2; P_Ty = Id - Sy_T^2; P_Tz = Id - Sz_T^2;
P_RA = 0.5*Id + Sz_R; P_RB  = 0.5*Id - Sz_R;
Rho_T = (w_Tx * P_Tx) + (w_Ty * P_Ty) + (w_Tz * P_Tz);
Rho_R = (w_RA * P_RA) + (w_RB * P_RB);
Sys.initState = Rho_T * Rho_R;
[~, specDyad] = pepper(Sys, Exp);

Sys2.S = 1/2; Sys2.g = g_R; Sys2.lwpp = 0.6;
[~, specR] = pepper(Sys2, Exp);
specFull = ((1 - abs(W_R)) * specDyad) + (W_R * specR);
specFull = specFull / max(specFull);
```

Suppl. Fig. 42: Transient ESR EasySpin code snippet.

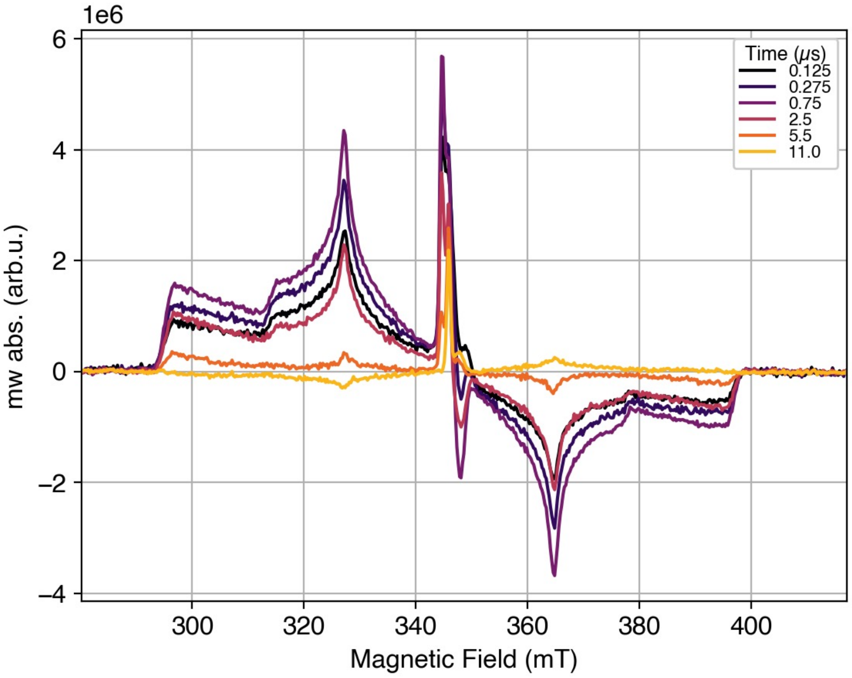

Suppl. Fig. 43: X-band Full Field transient ESR spectra on 100 μM frozen toluene solution of T-3Cz-Acr at 80 K, acquired after 600 nm 1 mJ pulses.

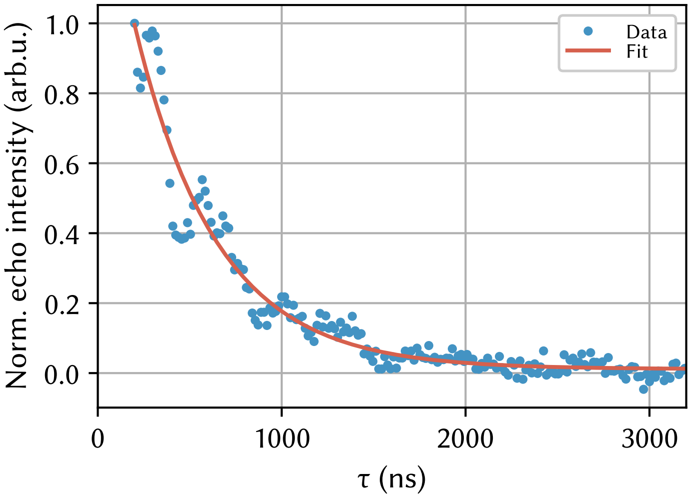

Suppl. Fig. 44: Phase coherence time of the quartet state a 100 μM frozen toluene solution of T-3Cz-An at 80 K, acquired at 328 mT at X-band 1.5 μs Delay After Flash after a 600 nm 1 mJ pulse. Fit to $I(\tau) \propto e^{-2\tau/T_m}$ yields $T_m = 0.90 \pm 0.03$ μs.

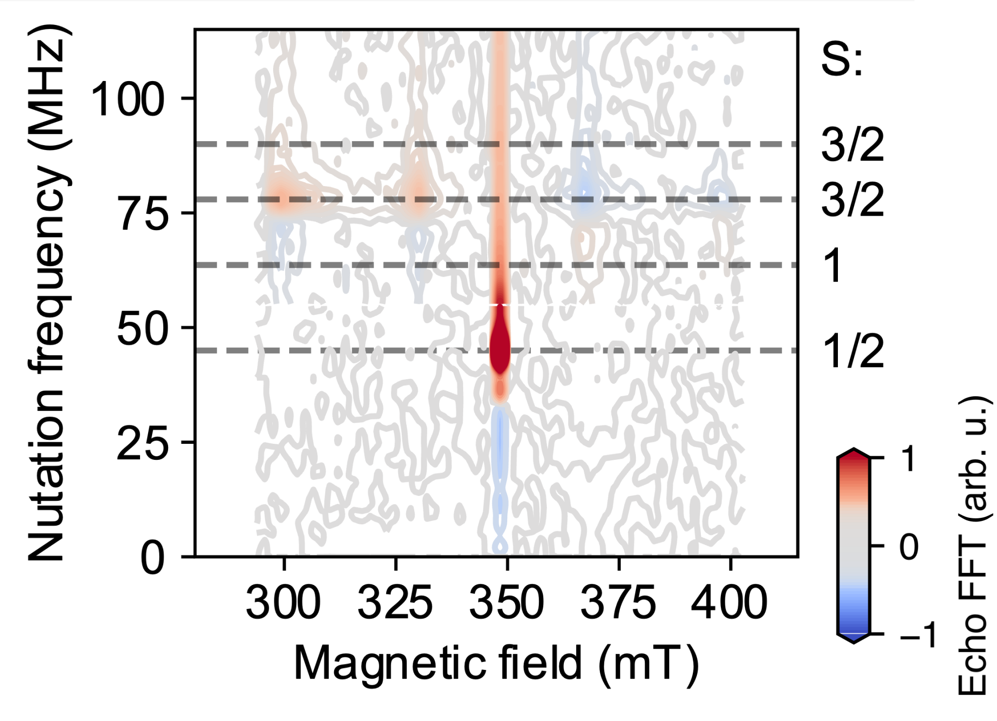

Suppl. Fig. 45: Transient nutation experiment on T-3Cz-Acr at 80 K.

Suppl. Note 2. Theoretical Considerations

This section contains a detailed analysis of the electronic structure theory of radical-acene systems used to derive the equations and design rules presented in the 'Theoretical Insights' section in the main article. Such systems consist of a 'radical' part and an 'acene' part which are covalently bonded. We will sometimes use the word 'dyad' to refer to radical-acene systems such as those presented in the main article.

Firstly, in Section S2.1 we define the electronic states of interest and give general expressions for their energies, assuming that the orbitals of interest can be identified as belonging mostly to either the radical or the acene, but not requiring that they are *exclusively* on the radical or acene.[10] We denote orbitals on the radical Rn where $n = 0$ is the SOMO, $n = 1$ is the HOMO, $n = 2$ the HOMO−1 and so on, $n = 1'$ is the LUMO, $n = 2'$ the LUMO+1 and so on. The acene orbitals are denoted similarly as An except there is no $A0$ orbital since the acene is closed-shell and therefore has no SOMO.

In Section S2.2 we then partition the molecule into radical and acene parts, where we assume the orbitals are solved separately for individual parts at infinite separation and are thus localised to either the radical or acene.[11] This gives the zeroth-order states. We then bring the two moieties together, and describe their action on each other as a perturbation. By analysing the effects of this perturbation and by comparing the results of the perturbation to the general expressions discussed above, we can formulate design rules for emissive radicals with accessible high spin states which are presented in Section S2.3.

S2.1 Relevant states and their energies

Here we define the relevant states of interest and give general expressions for their energies.[10,12]

S2.1.1 States

Ground state $|\Psi_0\rangle$ is the restricted open-shell ground state of the interacting system.

$$
\begin{aligned}
{}^2[D_0 S_0] &= |\Psi_0\rangle \\
&= |\cdots \Phi_{R1}\bar{\Phi}_{R1}\Phi_{A1}\bar{\Phi}_{A1}\Phi_{R0}\rangle
\end{aligned} \tag{1}
$$

Radical-centered and acene-centered excited states We consider four excited states resulting from excitation from one orbital on the radical to another orbital on the radical *or* excitation from one orbital on the acene to another orbital on the acene. These states are:

- ${}^2\mathrm{CT}_{\mathrm{Cz}}$ — the D_1 state on the radical and S_0 state on the acene [Eq. (2)]
- ${}^2\mathrm{LE}_{\mathrm{Ac}}$ — the D_0 state on the radical and T_1 state on the acene coupled to an overall *doublet* state [Eq. (3)]
- ${}^4\mathrm{LE}_{\mathrm{Ac}}$ — the D_0 state on the radical and T_1 state on the acene coupled to an overall *quartet* state [Eq. (4)]
- ${}^2[D_0 S_1]$ — the D_0 state on the radical and S_1 state on the acene Eq. (5)

See Fig. 46 for orbital assignments. Algebraically the states are given by

$$
{}^2\mathrm{CT}_{\mathrm{Cz}} = \left|\Psi_{R\bar{1}}^{R\bar{0}}\right\rangle \tag{2}
$$

$$
\begin{aligned}
{}^2\mathrm{LE}_{\mathrm{Ac}} &= \left|{}^{2T}\Psi_{A1}^{A1'}\right\rangle \\
&= \frac{1}{\sqrt{6}}\left(-\left|\Psi_{A1}^{A1'}\right\rangle + \left|\Psi_{A\bar{1}}^{A\bar{1}'}\right\rangle + 2\left|\Psi_{A\bar{1}R0}^{R\bar{0}A1'}\right\rangle\right)
\end{aligned} \tag{3}
$$

$$
\begin{aligned}
{}^4\mathrm{LE}_{\mathrm{Ac}} &= \left|{}^{4}\Psi_{A1}^{A1'}\right\rangle \\
&= \frac{1}{\sqrt{3}}\left(\left|\Psi_{A1}^{A1'}\right\rangle - \left|\Psi_{A\bar{1}}^{A\bar{1}'}\right\rangle + \left|\Psi_{A\bar{1}R0}^{R\bar{0}A1'}\right\rangle\right)
\end{aligned} \tag{4}
$$

$$
\begin{aligned}
{}^2[D_0 S_1] &= \left|{}^{2S}\Psi_{1A}^{1A'}\right\rangle \\
&= \frac{1}{\sqrt{2}}\left(\left|\Psi_{A1}^{A1'}\right\rangle + \left|\Psi_{A\bar{1}}^{A\bar{1}'}\right\rangle\right)
\end{aligned} \tag{5}
$$

The notation for the states is the same as that in Ref. 10.

Radical to acene charge transfer states We also consider the lowest energy radical to acene charge transfer state $^2\mathrm{CT}_{\mathrm{Ac}}$ which involves transfer of one electron from the acene HOMO to the radical SOMO resulting in the species R^-A^+ [Eq. (6)].[11]

$$^2\mathrm{CT}_{\mathrm{Ac}} = \left|\Psi_{A\bar{1}}^{R\bar{0}}\right\rangle \tag{6}$$

This state is shown to be the lowest energy from high level calculations and this is also evidenced by spectroscopic signals originating from the anthracene cation A^+.[13]

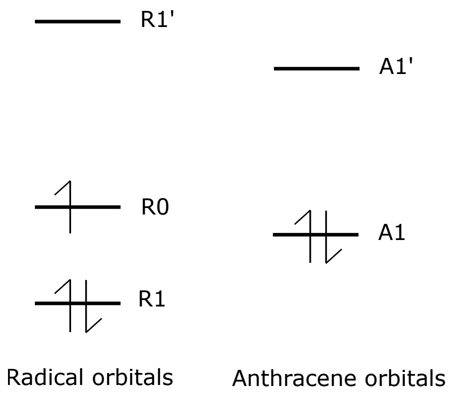

Suppl. Fig. 46: Ground state molecular orbital diagram of the radical-anthracene system.

S2.1.2 Energies

These energies are those which would be obtained by a full calculation on the dyad.

Ground state The energy of the ground state of the system is E_0.

$$\langle\Psi_0|\,\hat{H}\,|\Psi_0\rangle =: E_0 \tag{7}$$

Radical-centered and acene-centered excited states The energies of the four excited states from Eq. (2) – Eq. (5) are as follows:

$$E(^2\mathrm{CT_{Cz}}) = \left\langle \Psi^{R\bar{0}}_{R\bar{1}} \right| \hat{H} \left| \Psi^{R\bar{0}}_{R\bar{1}} \right\rangle = E_0 + F_{R0,R0} - F_{R1,R1} + \frac{1}{2} J_{R0,R0} - J_{R1,R0} + \frac{1}{2} K_{R1,R0} \tag{8}$$

$$E(^2\mathrm{LE_{Ac}}) = \left\langle {}^{2T}\Psi^{A1'}_{A1} \right| \hat{H} \left| {}^{2T}\Psi^{A1'}_{A1} \right\rangle = E_0 + F_{A1',A1'} - F_{A1,A1} + K_{R0,A1'} + K_{A1,R0} - J_{A1,A1'} \tag{9}$$

$$E(^4\mathrm{LE_{Ac}}) = \left\langle {}^{4}\Psi^{A1'}_{A1} \right| \hat{H} \left| {}^{4}\Psi^{A1'}_{A1} \right\rangle = E_0 + F_{A1',A1'} - F_{A1,A1} - \frac{1}{2} K_{R0,A1'} - \frac{1}{2} K_{A1,R0} - J_{A1,A1'} \tag{10}$$

$$E(^2[D_0S_1]) = \left\langle {}^{2S}\Psi^{A1'}_{A1} \right| \hat{H} \left| {}^{2S}\Psi^{A1'}_{A1} \right\rangle = E_0 + F_{A1',A1'} - F_{A1,A1} + 2K_{A1,A1'} - J_{A1,A1'} \tag{11}$$

Radical to acene charge transfer states The energy of the charge transfer state Eq. (6) is

$$E(^2\mathrm{CT_{Ac}}) = \left\langle \Psi^{R\bar{0}}_{A\bar{1}} \right| \hat{H} \left| \Psi^{R\bar{0}}_{A\bar{1}} \right\rangle = E_0 + F_{R0,R0} - F_{A1,A1} + \frac{1}{2} J_{R0,R0} + \frac{1}{2} K_{A1,R0} - J_{A1,R0} \tag{12}$$

S2.2 Perturbation Theory

We now consider partitioning the molecule into two parts, the 'radical' and the 'acene', and define the zeroth-order orbitals as being those solved independently for radical and acene parts at infinite separation. We then consider the interaction between the various states, from which we can construct design rules. As we do this, we ignore the polarisation terms (such as ΔE_0, $F^{(1)}_{Ri,Rj}$ and $F^{(1)}_{Ai,Aj}$) which would normally appear at first-order as small corrections to the energies of states (diagonal elements). Since these terms are affected by charge imbalance in the ground state and we believe this charge imbalance to be quite small, we can ignore them for the purposes of this discussion. A full discussion of first-order perturbation theory applied to covalently bonded dyads of closed-shell organic molecules, including polarisation effects, can be found in Ref. 11.

S2.2.1 Zeroth-order Hamiltonian

If we now consider a minimal basis of $^2\mathrm{CT}_{\mathrm{Cz}}$, $^2\mathrm{CT}_{\mathrm{Ac}}$, $^2\mathrm{LE}_{\mathrm{Ac}}$, $^2[D_0S_1]$ and $^4\mathrm{LE}_{\mathrm{Ac}}$ then at zeroth-order

$$E(^2\mathrm{CT}^{(0)}_{\mathrm{Cz}}) = F_{R0,R0} - F_{R1,R1} + \frac{1}{2}J_{R0,R0} - J_{R1,R0} + \frac{1}{2}K_{R1,R0} \tag{13a}$$

$$E(^2\mathrm{CT}^{(0)}_{\mathrm{Ac}}) = F_{R0,R0} - F_{A1,A1} + \frac{1}{2}J_{R0,R0} \tag{13b}$$

$$E(^2\mathrm{LE}^{(0)}_{\mathrm{Ac}}) = F_{A1',A1'} - F_{A1,A1} - J_{A1,A1'} \tag{13c}$$

$$E(^2[D_0S_1]^{(0)}) = F_{A1',A1'} - F_{A1,A1} + 2K_{A1,A1'} - J_{A1,A1'} \tag{13d}$$

$$E(^4\mathrm{LE}^{(0)}_{\mathrm{Ac}}) = F_{A1',A1'} - F_{A1,A1} - J_{A1,A1'}. \tag{13e}$$

There is no interaction between any of these states at zeroth-order.

S2.2.2 First-order Hamiltonian

We now perturb the excited states to first-order in the basis of zeroth-order orbitals. The first-order Hamiltonian in the basis of $^2\mathrm{CT}_{\mathrm{Cz}}$, $^2\mathrm{CT}_{\mathrm{Ac}}$, $^2\mathrm{LE}_{\mathrm{Ac}}$, $^2[D_0S_1]$ and $^4\mathrm{LE}_{\mathrm{Ac}}$ (neglecting polarisation effects) is

$$\mathbf{H}^{(1)} = \begin{pmatrix} 0 & -F_{R1,A1} & 0 & \sqrt{2}(R0R1|A1A1') & 0 \\ & -J_{A1,R0} & \frac{3}{\sqrt{6}}F_{R0,A1'} & \frac{1}{\sqrt{2}}F_{R0A1'} & 0 \\ & & 0 & 0 & 0 \\ & & & 0 & 0 \\ & & & & 0 \end{pmatrix} \tag{14}$$

As this matrix is Hermitian only the top right of the matrix is given. This matrix contains important information about the system:

1. The only state whose energy is perturbed at first-order is $^2\mathrm{CT}_{\mathrm{Ac}}$, which is lowered by the Coulombic attraction between an electron in the radical SOMO and a hole in the acene

HOMO.

2. The $^2\mathrm{CT}_{\mathrm{Cz}}$ state interacts with $^2\mathrm{CT}_{\mathrm{Ac}}$ via a Fock-matrix term which will depend on the amplitude of the acene HOMO and radical HOMO on the atoms whereby they are joined, and have a $\cos(\theta)$ dependence on the dihedral angle between the monomers.

3. The two bright states ($^2\mathrm{CT}_{\mathrm{Cz}}$ and $^2[D_0S_1]$) interact via a Förster interaction.

4. $^2\mathrm{CT}_{\mathrm{Ac}}$ interacts with $^2\mathrm{LE}_{\mathrm{Ac}}$ via a Fock matrix term which depends on the amplitude of the radical SOMO and acene LUMO on the atoms through which the moieties are joined, and has a $\cos(\theta)$ dependence on the dihedral angle between the monomers.

5. $^2\mathrm{CT}_{\mathrm{Ac}}$ interacts with $^2[D_0S_1]$ via a Fock matrix term which depends on the amplitude of the radical SOMO and acene LUMO on the atoms through which the moieties are joined, and has a $\cos(\theta)$ dependence on the dihedral angle between the monomers.

6. There is no direct interaction (at first-order) between the $^2\mathrm{LE}_{\mathrm{Ac}}$ state and either of the bright states ($^2\mathrm{CT}_{\mathrm{Cz}}$ or $^2[D_0S_1]$).

7. There is no interaction between the quartet state and any of the other states (this model does not include spin-orbit coupling).

In this simple picture the $^{2/4}\mathrm{LE}_{\mathrm{Ac}}$ states are degenerate at zeroth and first-order. This means that in practice the energy gap between them is likely to be extremely small relative to the energy gaps between the excited states of interest, which is experimentally found to be the case.

S2.2.3 Two-electron integrals

In this section we evaluate the exchange integrals $K_{R0,A1}$ between radical SOMO and acene HOMO, and $K_{R0,A1'}$ between the radical SOMO and acene LUMO by perturbing the orbitals to first-order. We describe the electronic structure of the system using the Pariser-Parr-Pople (PPP) model, which is similar to Hückel theory but including two-electron interactions for an accurate description of

excited states.[14–17] Usually one would perform the first-order perturbation by perturbing the excited states directly like in Section S2.2.2 using the zeroth-order orbitals instead of perturbing the orbitals themselves. At first-order, in the zeroth-order orbitals, some interactions between states on the two monomers are non-zero, for example those via Fock matrix elements $F^{(0)}_{Ri,Aj}$ or Coulomb integrals such as $J_{A1,R0}$. However, exchange integrals between orbitals on different monomers such as $K_{R0,A1}$ and $K_{R0,A1'}$ are strictly equal to zero even at first-order when calculated in the zeroth-order orbitals and when using a method such as PPP theory in which there is zero overlap between atomic orbitals on different centers (ZDO). Only by perturbing the orbitals, such that they are no longer spatially separated and may overlap slightly, may $K_{R0,A1}$ and $K_{R0,A1'}$ be non-zero in this model.

Definition of the exchange integral The exchange integral in chemists' notation is defined as[18]

$$\begin{aligned} K_{ij} &= (ij|ij) \\ &= \int dr_1 dr_2 \Phi_i(r_1)\Phi_j(r_1)\frac{1}{r_{12}}\Phi_i(r_2)\Phi_j(r_2) \end{aligned} \tag{15}$$

where Φ_i and Φ_j are molecular orbitals, r_1 and r_2 are the spatial coordinates of electron 1 and electron 2 and r_{12} is the scalar distance between the two electrons. If Φ_i and Φ_j are solved at zeroth-order and on different monomers then $K_{ij} = 0$ due to the orbitals being spatially separated, assuming ZDO.

Perturbing orbitals We consider the effect of a small perturbation from the zeroth-order orbitals on the exchange integral $K_{R0,A1}$. The relevant orbitals are Φ_{R0} and Φ_{A1}. Φ_{R0} becomes

$$\Phi^{(1)}_{R0} = \Phi^{(0)}_{R0} + \Phi^{(0)}_{A1}\frac{\langle A1|\hat{V}|R0\rangle}{E_{R0}-E_{A1}} + \Phi^{(0)}_{A1'}\frac{\langle A1'|\hat{V}|R0\rangle}{E_{R0}-E_{A1'}}, \tag{16}$$

and Φ_{A1} becomes

$$\Phi^{(1)}_{A1} = \Phi^{(0)}_{A1} + \Phi^{(0)}_{R1}\frac{\langle R1|\hat{V}|A1\rangle}{E_{A1}-E_{R1}} + \Phi^{(0)}_{R2}\frac{\langle R2|\hat{V}|A1\rangle}{E_{A1}-E_{R2}} + \Phi^{(0)}_{R0}\frac{\langle R0|\hat{V}|A1\rangle}{E_{A1}-E_{R0}}. \tag{17}$$

$\langle i|\hat{V}|j\rangle$ is the perturbation matrix element between orbitals Φ_i and Φ_j, and is equivalent to the Fock matrix element between those two orbitals

$$\langle i|\hat{V}|j\rangle = F_{i,j}. \tag{18}$$

We will also define the energy gap as

$$E_i - E_j = E_{ij}. \tag{19}$$

The two perturbation expressions now become

$$\Phi^{(1)}_{R0} = \Phi^{(0)}_{R0} + \Phi^{(0)}_{A1}\frac{F_{A1,R0}}{E_{R0,A1}} + \Phi^{(0)}_{A1'}\frac{F_{A1',R0}}{E_{R0,A1'}}, \tag{20a}$$

$$\Phi^{(1)}_{A1} = \Phi^{(0)}_{A1} + \Phi^{(0)}_{R1}\frac{F_{R1,A1}}{E_{A1,R1}} + \Phi^{(0)}_{R2}\frac{F_{R2,A1}}{E_{A1,R2}} + \Phi^{(0)}_{R0}\frac{F_{R0,A1}}{E_{A1,R0}}. \tag{20b}$$

Inserting Eq. (20a) and Eq. (20b) back into Eq. (15) we get for the exchange integral in the 1st order perturbed orbitals

$$\begin{aligned} K^{(1)}_{R0,A1} = \int dr_1 dr_2 &\left(\Phi^{(0)}_{R0} + \Phi^{(0)}_{A1}\frac{F_{A1,R0}}{E_{R0,A1}} + \Phi^{(0)}_{A1'}\frac{F_{A1',R0}}{E_{R0,A1'}}\right)(r_1)\cdot \\ &\left(\Phi^{(0)}_{A1} + \Phi^{(0)}_{R1}\frac{F_{R1,A1}}{E_{A1,R1}} + \Phi^{(0)}_{R2}\frac{F_{R2,A1}}{E_{A1,R2}} + \Phi^{(0)}_{R0}\frac{F_{R0,A1}}{E_{A1,R0}}\right)(r_1)\frac{1}{r_{12}}\cdot \\ &\left(\Phi^{(0)}_{R0} + \Phi^{(0)}_{A1}\frac{F_{A1,R0}}{E_{R0,A1}} + \Phi^{(0)}_{A1'}\frac{F_{A1',R0}}{E_{R0,A1'}}\right)(r_2)\cdot \\ &\left(\Phi^{(0)}_{A1} + \Phi^{(0)}_{R1}\frac{F_{R1,A1}}{E_{A1,R1}} + \Phi^{(0)}_{R2}\frac{F_{R2,A1}}{E_{A1,R2}} + \Phi^{(0)}_{R0}\frac{F_{R0,A1}}{E_{A1,R0}}\right)(r_2). \end{aligned} \tag{21}$$

The above expression Eq. (21) is quite messy and there are a large number of terms, however most of which can be ignored since they are either equal to zero or vanishingly small. Firstly, any term with a product $\Phi^{(0)}_{Ai}(r_1)\Phi^{(0)}_{Rj}(r_1)$ or $\Phi^{(0)}_{Ai}(r_2)\Phi^{(0)}_{Rj}(r_2)$ will automatically vanish due to neglect of differential overlap and spatially separated zeroth-order orbitals. This first and foremost means that the zeroth-order term $K^{(0)}_{R0,A1}$ vanishes. Any term of first-order in $F_{i,j}/E_{j,i}$ will also vanish, as will some but

not all higher order terms. We will ignore any term higher than second-order in $F_{i,j}/E_{j,i}$ as these terms will be very small. Therefore, the expression is dominated by the second-order terms. These terms are

$$\begin{aligned} K^{(1)}_{R0,A1} = &\left(\frac{F_{A1,R0}}{E_{R0,A1}}\right)^2 J_{A1,A1} + \left(\frac{F_{R0,A1}}{E_{A1,R0}}\right)^2 J_{R0,R0} - 2\left(\frac{F_{A1,R0}}{E_{R0,A1}}\right)^2 J_{A1,R0} \\ &+ \left(\frac{F_{A1',R0}}{E_{R0,A1'}}\right)^2 K_{A1',A1} + \left(\frac{F_{R1,A1}}{E_{A1,R1}}\right)^2 K_{R0,R1} + \left(\frac{F_{R2,A1}}{E_{A1,R2}}\right)^2 K_{R0,R2} \\ &+ 2\frac{F_{A1',R0}F_{A1,R0}}{E_{R0,A1'}E_{R0,A1}}(A1'A1|A1A1) + 2\frac{F_{R1,A1}F_{R0,A1}}{E_{A1,R1}E_{A1,R0}}(R1R0|R0R0) \\ &+ 2\frac{F_{R2,A1}F_{R0,A1}}{E_{A1,R2}E_{A1,R0}}(R2R0|R0R0). \end{aligned} \tag{22}$$

The first three terms will be largest since Coulomb integrals are larger than exchange integrals. The Fock matrix element $F_{Ri,Aj}$ between orbitals on R and A can be expressed in PPP theory as

$$F_{Ri,Aj} = C_{Ri,\mu^*} C_{Aj,\nu^*} t \cos\theta \tag{23}$$

where θ is the dihedral angle between the monomers R and A, C_{Ri,μ^*} and C_{Aj,ν^*} are the coefficients of the orbitals Ri and Aj on the joining atoms μ^* and ν^* on R and A respectively and t is the Hückel resonance parameter.[11]

As all the terms are second-order in $F_{Ri,Aj}$, we get the following relation

$$K^{(1)}_{R0,A1} \propto t^2 \cos^2\theta \tag{24}$$

Following the same steps as above for $K_{R0,A1'}$ we also find that $K^{(1)}_{R0,A1'} \propto t^2 \cos^2\theta$. Therefore,

$$\therefore K^{(1)}_{R0,A1} + K^{(1)}_{R0,A1'} \propto \beta^2 \cos^2\theta. \tag{25}$$

This model also predicts the exchange to be dependent on the coefficients of the radical and acene orbitals $C_{Ri,\mu^*}, C_{Aj,\nu^*}$ on the joining atoms.

S2.2.4 Coupling of $^2\mathrm{CT}_{\mathrm{Cz}}$, $^2\mathrm{CT}_{\mathrm{Ac}}$ and $^2\mathrm{LE}_{\mathrm{Ac}}$

First order coupling of $^2\mathrm{CT}_{\mathrm{Cz}}$ and $^2\mathrm{LE}_{\mathrm{Ac}}$ with $^2\mathrm{CT}_{\mathrm{Ac}}$: Examining the first-order Hamiltonian [Eq. (14)], again assuming the neglect of differential overlap, we find that $^2\mathrm{CT}_{\mathrm{Cz}}$ and $^2\mathrm{LE}_{\mathrm{Ac}}$ both interact with $^2\mathrm{CT}_{\mathrm{Ac}}$ via one-electron terms

$$\left\langle {}^2\mathrm{CT}^{(0)}_{\mathrm{Ac}} \right| \hat{H}^{(1)} \left| {}^2\mathrm{CT}^{(0)}_{\mathrm{Cz}} \right\rangle = -F_{R1,A1} \tag{26a}$$

$$\left\langle {}^2\mathrm{CT}^{(0)}_{\mathrm{Ac}} \right| H^{(1)} \left| {}^2\mathrm{LE}^{(0)}_{\mathrm{Ac}} \right\rangle = \frac{3}{\sqrt{6}} F_{R0,A1'} \tag{26b}$$

where Eq. (26a) can be thought of as the carbazole-acene coupling and Eq. (26b) the TTM-acene coupling. Evaluating both of these coupling terms in PPP theory for localised orbitals gives an interaction of localised orbitals via a covalent bond between the radical and acene

$$\left\langle {}^2\mathrm{CT}^{(0)}_{\mathrm{Ac}} \right| \hat{H}^{(1)} \left| {}^2\mathrm{CT}^{(0)}_{\mathrm{Cz}} \right\rangle = -C_{R1,\mu^*} C_{A1,\nu^*} t \cos\theta \tag{27a}$$

$$\left\langle {}^2\mathrm{CT}^{(0)}_{\mathrm{Ac}} \right| H^{(1)} \left| {}^2\mathrm{LE}^{(0)}_{\mathrm{Ac}} \right\rangle = \frac{3}{\sqrt{6}} C_{R0,\mu^*} C_{A1',\nu^*} t \cos\theta. \tag{27b}$$

Second order coupling of $^2\mathrm{CT}_{\mathrm{Cz}}$ and $^2\mathrm{LE}_{\mathrm{Ac}}$: Now we consider the coupling between $^2\mathrm{CT}_{\mathrm{Cz}}$ and $^2\mathrm{LE}_{\mathrm{Ac}}$. We notice that $^2\mathrm{CT}_{\mathrm{Cz}}$ and $^2\mathrm{LE}_{\mathrm{Ac}}$ are not directly mixed by the first-order Hamiltonian in the zeroth-order orbitals. In the dyad orbitals, however, these two states can be shown to interact directly via a Dexter term $-\frac{3}{\sqrt{6}}(R0A1'|A1R1)$. The two states can also interact indirectly, via $^2\mathrm{CT}_{\mathrm{Ac}}$ and this indirect coupling can be non-zero even in the zeroth-order orbitals. We consider the indirect coupling here.

We have already shown that $^2\mathrm{CT}_{\mathrm{Cz}}$ and $^2\mathrm{LE}_{\mathrm{Ac}}$ both interact with $^2\mathrm{CT}_{\mathrm{Ac}}$ at first-order. At first-

order, a small amount of $^2\mathrm{CT}_{\mathrm{Ac}}$ mixes into $^2\mathrm{CT}_{\mathrm{Cz}}$ and $^2\mathrm{LE}_{\mathrm{Ac}}$

$$\left|^2\mathrm{CT}^{(1)}_{\mathrm{Cz}}\right\rangle = \left|^2\mathrm{CT}^{(0)}_{\mathrm{Cz}}\right\rangle + \frac{\left\langle ^2\mathrm{CT}^{(0)}_{\mathrm{Cz}}\right| \hat{H}^{(1)} \left|^2\mathrm{CT}^{(0)}_{\mathrm{Ac}}\right\rangle}{E(^2\mathrm{CT}^{(0)}_{\mathrm{Ac}}) - E(^2\mathrm{CT}^{(0)}_{\mathrm{Cz}})} \left|^2\mathrm{CT}^{(0)}_{\mathrm{Ac}}\right\rangle \tag{28a}$$

$$\left|^2\mathrm{LE}^{(1)}_{\mathrm{Ac}}\right\rangle = \left|^2\mathrm{LE}^{(0)}_{\mathrm{Ac}}\right\rangle + \frac{\left\langle ^2\mathrm{LE}^{(0)}_{\mathrm{Ac}}\right| \hat{H}^{(1)} \left|^2\mathrm{CT}^{(0)}_{\mathrm{Ac}}\right\rangle}{E(^2\mathrm{CT}^{(0)}_{\mathrm{Ac}}) - E(^2\mathrm{LE}^{(0)}_{\mathrm{Ac}})} \left|^2\mathrm{CT}^{(0)}_{\mathrm{Ac}}\right\rangle . \tag{28b}$$

We now consider the second-order Hamiltonian element between $^2\mathrm{CT}_{\mathrm{Cz}}$ and $^2\mathrm{LE}_{\mathrm{Ac}}$ by taking the first order Hamiltonian element of the perturbed states $\left|^2\mathrm{CT}^{(1)}_{\mathrm{Cz}}\right\rangle$ and $\left|^2\mathrm{LE}^{(1)}_{\mathrm{Ac}}\right\rangle$ assuming that the orbitals are real

$$\begin{aligned}
\left\langle ^2\mathrm{CT}_{\mathrm{Cz}}\right| \hat{H}^{(2)} \left|^2\mathrm{LE}_{\mathrm{Ac}}\right\rangle &= \left\langle ^2\mathrm{CT}^{(1)}_{\mathrm{Cz}}\right| \hat{H}^{(1)} \left|^2\mathrm{LE}^{(1)}_{\mathrm{Ac}}\right\rangle \\
&= \left\langle ^2\mathrm{CT}^{(0)}_{\mathrm{Cz}}\right| \hat{H}^{(1)} \left|^2\mathrm{LE}^{(0)}_{\mathrm{Ac}}\right\rangle \\
&\quad + \frac{\left\langle ^2\mathrm{CT}^{(0)}_{\mathrm{Ac}}\right| \hat{H}^{(1)} \left|^2\mathrm{CT}^{(0)}_{\mathrm{Cz}}\right\rangle}{E(^2\mathrm{CT}^{(0)}_{\mathrm{Ac}}) - E(^2\mathrm{CT}^{(0)}_{\mathrm{Cz}})} \left\langle ^2\mathrm{CT}^{(0)}_{\mathrm{Ac}}\right| H^{(1)} \left|^2\mathrm{LE}^{(0)}_{\mathrm{Ac}}\right\rangle \\
&\quad + \frac{\left\langle ^2\mathrm{LE}^{(0)}_{\mathrm{Ac}}\right| \hat{H}^{(1)} \left|^2\mathrm{CT}^{(0)}_{\mathrm{Ac}}\right\rangle}{E(^2\mathrm{CT}^{(0)}_{\mathrm{Ac}}) - E(^2\mathrm{LE}^{(0)}_{\mathrm{Ac}})} \left\langle ^2\mathrm{CT}^{(0)}_{\mathrm{Cz}}\right| \hat{H}^{(1)} \left|^2\mathrm{CT}^{(0)}_{\mathrm{Ac}}\right\rangle \\
&\quad + \frac{\left\langle ^2\mathrm{CT}^{(0)}_{\mathrm{Ac}}\right| \hat{H}^{(1)} \left|^2\mathrm{CT}^{(0)}_{\mathrm{Cz}}\right\rangle \left\langle ^2\mathrm{LE}^{(0)}_{\mathrm{Ac}}\right| \hat{H}^{(1)} \left|^2\mathrm{CT}^{(0)}_{\mathrm{Ac}}\right\rangle}{\left\{E(^2\mathrm{CT}^{(0)}_{\mathrm{Ac}}) - E(^2\mathrm{CT}^{(0)}_{\mathrm{Cz}})\right\}\left\{E(^2\mathrm{CT}^{(0)}_{\mathrm{Ac}}) - E(^2\mathrm{LE}^{(0)}_{\mathrm{Ac}})\right\}} \\
&\quad \cdot \left\langle ^2\mathrm{CT}^{(0)}_{\mathrm{Ac}}\right| \hat{H}^{(1)} \left|^2\mathrm{CT}^{(0)}_{\mathrm{Ac}}\right\rangle .
\end{aligned} \tag{29}$$

The first term will be zero as there is no interaction between $^2\mathrm{CT}_{\mathrm{Cz}}$ and $^2\mathrm{LE}_{\mathrm{Ac}}$ at first-order. The last term will also be zero as the first-order Hamiltonian element $\left\langle ^2\mathrm{CT}^{(0)}_{\mathrm{Ac}}\right| \hat{H}^{(1)} \left|^2\mathrm{CT}^{(0)}_{\mathrm{Ac}}\right\rangle = 0$ (the

first-order correction to the energy of the $^{2}\mathrm{CT}_{\mathrm{Ac}}$ state). Therefore we end up with only two terms

$$\left\langle {}^{2}\mathrm{CT}_{\mathrm{Cz}}\right| \hat{H}^{(2)} \left|{}^{2}\mathrm{LE}_{\mathrm{Ac}}\right\rangle = \frac{\left\langle {}^{2}\mathrm{CT}^{(0)}_{\mathrm{Ac}}\right| \hat{H}^{(1)} \left|{}^{2}\mathrm{CT}^{(0)}_{\mathrm{Cz}}\right\rangle}{E({}^{2}\mathrm{CT}^{(0)}_{\mathrm{Ac}}) - E({}^{2}\mathrm{CT}^{(0)}_{\mathrm{Cz}})} \left\langle {}^{2}\mathrm{CT}^{(0)}_{\mathrm{Ac}}\right| H^{(1)} \left|{}^{2}\mathrm{LE}^{(0)}_{\mathrm{Ac}}\right\rangle + \frac{\left\langle {}^{2}\mathrm{LE}^{(0)}_{\mathrm{Ac}}\right| \hat{H}^{(1)} \left|{}^{2}\mathrm{CT}^{(0)}_{\mathrm{Ac}}\right\rangle}{E({}^{2}\mathrm{CT}^{(0)}_{\mathrm{Ac}}) - E({}^{2}\mathrm{LE}^{(0)}_{\mathrm{Ac}})} \left\langle {}^{2}\mathrm{CT}^{(0)}_{\mathrm{Cz}}\right| \hat{H}^{(1)} \left|{}^{2}\mathrm{CT}^{(0)}_{\mathrm{Ac}}\right\rangle . \tag{30}$$

Inserting the first-order terms Eq. (26a) and Eq. (26b) into Eq. (30), we get

$$\left\langle {}^{2}\mathrm{CT}_{\mathrm{Cz}}\right| \hat{H}^{(2)} \left|{}^{2}\mathrm{LE}_{\mathrm{Ac}}\right\rangle = -\frac{3}{\sqrt{6}} F^{(0)}_{R1,A1} F^{(0)}_{R0,A1'} \left(\frac{1}{E({}^{2}\mathrm{CT}^{(0)}_{\mathrm{Ac}}) - E({}^{2}\mathrm{CT}^{(0)}_{\mathrm{Cz}})} + \frac{1}{E({}^{2}\mathrm{CT}^{(0)}_{\mathrm{Ac}}) - E({}^{2}\mathrm{LE}^{(0)}_{\mathrm{Ac}})} \right). \tag{31}$$

Again using Eq. (27b) and Eq. (27b) for $F_{Ri,Aj}$, the Hamiltonian element becomes[11]

$$\left\langle {}^{2}\mathrm{CT}_{\mathrm{Cz}}\right| \hat{H}^{(2)} \left|{}^{2}\mathrm{LE}_{\mathrm{Ac}}\right\rangle = -\frac{3}{\sqrt{6}} C_{R1,\mu^*} C_{R0,\mu^*} C_{A1,\nu^*} C_{A1',\nu^*} \beta^2 \cos^2\theta \left(\frac{1}{E({}^{2}\mathrm{CT}^{(0)}_{\mathrm{Ac}}) - E({}^{2}\mathrm{CT}^{(0)}_{\mathrm{Cz}})} + \frac{1}{E({}^{2}\mathrm{CT}^{(0)}_{\mathrm{Ac}} - E({}^{2}\mathrm{LE}^{(0)}_{\mathrm{Ac}})} \right). \tag{32}$$

Therefore, the interaction between $^{2}\mathrm{CT}_{\mathrm{Cz}}$ and $^{2}\mathrm{LE}_{\mathrm{Ac}}$ at second order is directly proportional to the square of the cosine of the dihedral angle θ between the radical and acene

$$\therefore \left\langle {}^{2}\mathrm{CT}_{\mathrm{Cz}}\right| \hat{H}^{(2)} \left|{}^{2}\mathrm{LE}_{\mathrm{Ac}}\right\rangle \propto \beta^2 \cos^2\theta. \tag{33}$$

S2.3 Design Rules

S2.3.1 D_1–T_1 energy resonance criterion

The energies of the $^2\mathrm{CT}_{\mathrm{Cz}}$ and $^4\mathrm{LE}_{\mathrm{Ac}}$ states of the dyad, given in Eq. (8) and Eq. (10) are repeated below:

$$E(^2\mathrm{CT}_{\mathrm{Cz}}) = F_{R0,R0} - F_{R1,R1} + \frac{1}{2}J_{R0,R0} - J_{R1,R0} + \frac{1}{2}K_{R1,R0} \tag{34}$$

$$E(^4\mathrm{LE}_{\mathrm{Ac}}) = F_{A1',A1'} - F_{A1,A1} - \frac{1}{2}K_{R0,A1'} - \frac{1}{2}K_{A1,R0} - J_{A1,A1'}. \tag{35}$$

If we now consider the isolated molecules, the energy of the D_1 state of the radical is

$$E(^2[D_1]) = F_{R0,R0} - F_{R1,R1} + \frac{1}{2}J_{R0,R0} - J_{R1,R0} + \frac{1}{2}K_{R1,R0} \tag{36}$$

and the energy of the T_1 state on the acene is

$$E(^3[T_1]) = F_{A1',A1'} - F_{A1,A1} - J_{A1,A1'}. \tag{37}$$

Eq. (36) and Eq. (37) are the same as the zeroth-order energies of $^2\mathrm{CT}_{\mathrm{Cz}}$ and $^{2/4}\mathrm{LE}_{\mathrm{Ac}}$ from Eq. (13a) and Eq. (13c)/Eq. (13e) respectively (see Section S2.2.1). Comparing equations (35), (37) and (13e), the $-\frac{1}{2}(K_{R0,A1'} + K_{A1,R0})$ term in the quartet energy of the dyad [Eq. (35)] vanishes in the triplet energy of the acene [Eq. (37)] and the quartet energy of the dyad at zeroth-order [Eq. (13e)]. This term is half of the exchange energy of an electron in the radical SOMO with one in the acene HOMO, plus half of the exchange energy of an electron in the radical SOMO with one in the acene LUMO.

At zeroth-order:

1. The ${}^2\mathrm{CT}_{\mathrm{Cz}}$ energy of the dyad is equal to the D_1 energy of the isolated radical.
2. The ${}^4[D_0T_1]$ energy of the dyad is equal to the T_1 energy of the isolated acene.

We also notice that, ignoring polarisation effects, the D_1 energy of the radical is equal to the ${}^2\mathrm{CT}_{\mathrm{Cz}}$ energy of the dyad at first-order in Eq. (14) from Section S2.2.2. The first-order energy of ${}^4\mathrm{LE}_{\mathrm{Ac}}$ is also the same as that of T_1 in the zeroth-order orbitals in Eq. (14). However, at higher levels of theory, the exchange term is non-vanishing and ${}^4\mathrm{LE}_{\mathrm{Ac}}$ is stabilised by approximately $\frac{1}{2}(K_{R0,A1'} + K_{A1,R0})$. This exchange energy is exactly zero for the zeroth-order orbitals as the radical SOMO and acene HOMO/LUMO are spatially disjoint.

Therefore the energy resonance criterion is equivalent to approximating

$$\frac{1}{2}K_{R0,A1'} + \frac{1}{2}K_{A1,R0} \simeq 0. \tag{38}$$

Therefore, provided this exchange stabilisation plus the D_1–T_1 energy gap is comparable to the thermal energy

$$\frac{1}{2}(K_{A1,R0} + K_{R0,A1'}) + E_{D_1} - E_{T_1} \simeq kT, \tag{39}$$

then quartet-mediated emission is energetically possible.

S2.3.2 ${}^2\mathrm{LE}_{\mathrm{Ac}}$–${}^4\mathrm{LE}_{\mathrm{Ac}}$ exchange energy

The difference between the energies of the ${}^2\mathrm{LE}_{\mathrm{Ac}}$ and ${}^4\mathrm{LE}_{\mathrm{Ac}}$ states is given by subtracting Eq. (10) from Eq. (9) which is

$$E(D) - E(Q) = E_{{}^2\mathrm{LE}_{\mathrm{Ac}}} - E_{{}^4\mathrm{LE}_{\mathrm{Ac}}} = \frac{3}{2}\big(K_{A1,R0} + K_{R0,A1'}\big). \tag{40}$$

$E(D) - E(Q)$ depends on the connectivity of the molecule as evidenced by CAS(3,3) + QD-NEVPT2 / TZVP calculations presented in Table 9. Molecules where the TTM is directly bonded to the acene (A-T-C connectivity) exhibit larger, more positive doublet-quartet gaps $E(D) - E(Q)$ compared to those where the TTM and acene are connected via carbazole (T-C-A connectivity). This is to be expected from simple orbital overlap arguments as the overlap between $R0$ (centered on TTM) and $A1$, $A1'$ (centered on the acene) is greater when TTM and acene moieties are directly bonded, so $K_{A1,R0}$ and $K_{R0,A1'}$ will be larger. This is also in agreement with the perturbation theory model as the integral [Eq. (22)] is dominated by $F_{R0,A1}$ and $F_{R0,A1'}$ terms which will be largest when the TTM and acene are directly bonded.

The doublet-quartet gap is largest for Acene-TTM-Carbazole type connectivity.

We also note the energy gap between the ${}^2\mathrm{LE}_{\mathrm{Ac}}$ and ${}^2[D_0S_1]$ states to be

$$E({}^2\mathrm{LE}_{\mathrm{Ac}}) - E({}^2[D_0S_1]) = 2K_{A1,A1'} - K_{R0,A1'} - K_{R0,A1} \tag{41}$$

in the dyad orbitals. When the acene HOMO and LUMO are in the same part of space and far separated from the radical SOMO, we would expect $K_{A1,A1'} \gg K_{R0,A1'}$ and $K_{A1,A1'} \gg K_{R0,A1}$ such that Eq. (41) is positive. In addition, at infinite separation, Eq. (41) reduces to

$$E({}^2\mathrm{LE}_{\mathrm{Ac}}^{(0)}) - E({}^2[D_0S_1]^{(0)}) = 2K_{A1,A1'} \tag{42}$$

which is the conventional singlet/triplet splitting.

Caution should be exercised in interpreting these equations in cases where the dyad orbitals can

Suppl. Tab. 9: Doublet–quartet gaps $E(D)-E(Q)$ for a series of molecules presented in this paper calculated by CAS(3,3) + QD-NEVPT2 / TZVP. The doublet–quartet gap is larger for molecules with A-T-C connectivity than for those with T-C-A connectivity.

Molecule	An-T-3PCz	An-T-1Cz	T-3Cz-An	T-3Cz-Acr
$E(D) - E(Q)$ / meV	1.70	1.95	-0.01	0.04

not be identified as belonging predominantly to the acene nor the radical. In such cases, perturbation theory would not be able to give reliable results. However, for all the molecules discussed in this work, the orbitals are separable between the acene or radical.

S2.3.3 Radical-acene coupling

To avoid non-radiative decay, the coupling between the $^2\mathrm{LE}_{\mathrm{Ac}}$ and $^2\mathrm{CT}_{\mathrm{Ac}}$ states should be small. We recall that the $^2\mathrm{CT}_{\mathrm{Cz}}$–$^2\mathrm{CT}_{\mathrm{Ac}}$ and $^2\mathrm{LE}_{\mathrm{Ac}}$–$^2\mathrm{CT}_{\mathrm{Ac}}$ couplings are moderated by F_{R1A1} and $\frac{3}{\sqrt{6}}F_{R0,A1'}$ terms, which are effectively the carbazole-acene and TTM-acene electronic couplings respectively (see Eq. (26a) and Eq. (26b)). We thus calculate the F_{R1A1} and $\frac{3}{\sqrt{6}}F_{R0,A1'}$ terms for the series of radical-acene dyads presented in this paper whose absolute magnitudes are shown in Table 10. We find that the relative size of the calculated TTM-acene and carbazole-acene electronic couplings depend on the connectivity, where molecules with A-T-C connectivity exhibit larger TTM-acene coupling, but conversely have smaller carbazole-acene coupling than those with T-C-A connectivity. This matches the relative proximity of TTM, carbazole and acene groups in the two connectivities. The trend in the TTM-acene electronic coupling specifically leads to the following design rule:

The coupling between $^2\mathrm{LE}_{\mathrm{Ac}}$–$^2\mathrm{CT}_{\mathrm{Ac}}$ states and thus non-radiative decay is minimised by adopting a TTM-carbazole-acene type connectivity.

Suppl. Tab. 10: TTM-acene coupling $\frac{3}{\sqrt{6}}F_{R0,A1'}$, carbazole-acene coupling F_{R1A1} and Coulomb stabilisation $J_{A1,R0}$ terms for a series of radical-acene dyads investigated in this paper calculated using localised orbitals with ExROPPP (for radical parts) and PPP-CIS (for acene parts).

Molecule	Connectivity	$\frac{3}{\sqrt{6}}\lvert F_{R0,A1'}\rvert$ / meV	$\lvert F_{R1A1}\rvert$ / meV	$J_{A1,R0}$ / eV
An-T-3PCz	A-T-C	29.6	7.0	1.17
An-T-1Cz	A-T-C	36.3	7.2	1.17
T-3Cz-An	T-C-A	6.0	56.3	0.75
T-3Cz-Acr	T-C-A	12.7	104.3	0.67

S2.3.4 Relative energy of CT states

We also want any dark charge-transfer states to be sufficiently high in energy above the quartet state such that they do not act as a dark trap. As mentioned earlier, the lowest energy radical-acene CT state is shown to be $^2\mathrm{CT}_{\mathrm{Ac}}$ which corresponds to $\left|\Psi_{A\bar{1}}^{R\bar{0}}\right\rangle$ [Eq. (12)].[13] The energy gap between the quartet state and lowest CT state is therefore Eq. (12) − Eq. (10), which is

$$E_{\mathrm{CT}} - E_Q = F_{R0,R0} - F_{A1',A1'} + K_{A1,R0} - J_{A1,R0} + J_{A1,A1'} + \frac{1}{2}K_{R0,A1'} + \frac{1}{2}J_{R0,R0}. \tag{43}$$

We notice that the most important terms will be $F_{R0,R0}$, $-F_{A1',A1'}$, $K_{A1,R0}$, $-J_{A1,R0}$ and $J_{A1,A1'}$. The first two terms $F_{R0,R0} - F_{A1',A1'}$ are the energy gap between the radical SOMO and acene LUMO which is assumed to be negative as the radical SOMO Φ_{R_0} is usually lower in energy than the acene LUMO $\Phi_{A1'}$. Therefore we want the magnitude of $F_{R0,R0} - F_{A1',A1'}$ to be small. However, minimising this orbital energy gap to destabilize the $^2\mathrm{CT}_{\mathrm{Ac}}$ state will in turn stabilise the other CT state $^2[R^+A^-]$ (where an electron is instead transferred from the radical to the acene).

The next two terms are the exchange and Coulomb energy of the interacting charge distributions of the radical SOMO and acene HOMO. As $J_{A1,R0}$ will always be larger than $K_{A1,R0}$ we want to minimise the magnitude of $K_{A1,R0} - J_{A1,R0}$. $J_{A1,R0}$ and $J_{A1,A1'}$ will always be positive so we want to minimise $J_{A1,R0}$ and maximise $J_{A1,A1'}$ in order for a large CT–quartet gap. $J_{A1,A1'}$ will generally be large for acenes as they are alternant hydrocarbons meaning that on each atom, the HOMO and LUMO have equal magnitude orbital coefficients. $J_{A1,R0}$ will depend on the distance between TTM and acene groups, as the SOMO is centered on TTM, and the acene HOMO centered on the acene. Therefore, altering the connectivity of acene, TTM and carbazole fragments should allow one to modulate the Coulomb stabilisation $J_{A1,R0}$.

This is numerically supported by the CT state energies of An-T-1Cz, An-T-3PCz, T-3Cz-An and T-3Cz-Acr from TD-DFT, presented in Tables 13–16. An-T-1Cz and An-T-3PCz have lower energy CT states than T-3Cz-An and T-3Cz-Acr as the former two molecules have TTM and acene groups closer together (A-T-C connectivity), whereas in the latter two molecules the TTM and

acene are bridged by the carbazole (T-C-A connectivity) and spatially separated, resulting in a larger Coulomb stabilisation $J_{A1,R0}$ of the CT state in the former case. We also calculated $J_{A1,R0}$ for these four molecules using the same PPP model, included in Table 10. Here we find $J_{A1,R0}$ values for T-3Cz-An and T-3Cz-Acr are lower than those of An-T-3PCz and An-T-1Cz, further supporting the conclusion that the Coulombic stabilisation of the CT state is modulated by the connectivity. This leads to the following design rule:

To raise the energy of the CT state relative to the quartet state, a TTM-carbazole-acene type connectivity is advantageous.

S2.4 Computational Details for ExROPPP calculations.

To predict the electronic interactions of radical-acene dyads in Sections S2.3.3 and S2.3.4 an approach combining PPP theory and perturbation theory was used, building on prior work on covalently bonded dyads.[11]

S2.4.1 PPP Hamiltonian

Pople's formulation of PPP theory defines a Fock matrix:[14]

$$F_{\mu\mu} = \epsilon_\mu + \frac{1}{2}P_{\mu\mu}\gamma_{\mu\mu} + \sum_{v\neq\mu}(P_{vv} - Z_v)\gamma_{\mu v}, \tag{44}$$

$$F_{\mu v} = t_{\mu v} - \frac{1}{2}P_{\mu v}\gamma_{\mu v}. \tag{45}$$

where ϵ_μ is the self-energy of an electron in a p-orbital on atom μ neglecting all other electrons, $t_{\mu v}$ is the hopping (or Hückel resonance) energy of an electron between p-orbitals on atoms μ and v, and $\gamma_{\mu v}$ parameterises the two electron integrals $(\mu v|\rho\sigma) = \delta_{\mu v}\delta_{\rho\sigma}\gamma_{\mu\rho}$ assuming the neglect of differential overlap. The Fock matrix has the same form for closed-shell (PPP) and radical (ExROPPP) formulations of PPP theory, provided the density matrix $\mathbf{P}$ is adjusted for closed- or open-shells, see Refs. 10,12.

S2.4.2 General procedure

In this approach, the radical-acene dyads are first divided into radical (TTM-carbazole) and acene fragments with separate molecular coordinate files starting from the optimised molecular geometries detailed in the Materials and Methods Section of the main text. The fragments are truncated to remove all sp^3 atoms before being input into PPP calculations. The electronic structures of these fragments are then solved separately using two versions of PPP theory: for acene fragments the conventional closed-shell PPP theory with Configuration Interaction Singles (CIS) was used;[14–17] and for doublet states of the radical fragments the recently devised ExROPPP method was used.[10]

Zeroth-order orbitals and integrals To calculate the radical-acene coupling and Coulombic stabilisation of the $^2CT_{Ac}$ states in Sections S2.3.3 and S2.3.4 the zeroth-order orbitals and integrals (Fock elements and two-electron integrals) for the acene fragments were obtained from a self-consistent closed-shell PPP calculation for the ground state. Zeroth-order orbitals and integrals for radical fragments were obtained from a self-consistent ExROPPP calculation for the ground state.[10,19]

The coupling Fock elements $F_{R0A1'}$ and F_{R1A1} were then approximated by considering the atomic Fock matrix elements F_{μ^*,ν^*} and coefficients $C_{Ri,\mu^*}, C_{Aj,\nu^*}$ of the zeroth-order orbitals on the connecting atoms μ^*, ν^* according to Eq. (27a) and Eq. (27b) and the Coulomb integrals $J_{A1,R0}$ were calculated in the zeroth-order orbitals from separate calculations on the two fragments. These one- and two-electron integrals prove key to explaining the optoelectronic behaviour of the different radical-acene dyad connectivities.

Calculation of $J_{A1,R0}$ The Coulomb stabilisation $J_{A1,R0}$ of the $^2CT_{Ac}$ states was calculated for the four radicals using the localised orbitals of the radical and acene using the standard expression for

the Coulomb integral

$$\begin{aligned} J_{A1,R0} &= (A1A1|R0R0) \\ &= \int dr_1 dr_2 \Phi^*_{A1}(r_1)\Phi^*_{A1}(r_1)\frac{1}{r_{12}}\Phi_{R0}(r_2)\Phi_{R0}(r_2) && (46) \\ &= \sum_{\mu}\sum_{\nu}\sum_{\rho}\sum_{\sigma} C^*_{\mu,A1}C^*_{\nu,A1}C_{\rho,R0}C_{\sigma,R0}(\mu\nu|\rho\sigma) && (47) \\ &= \sum_{\mu}\sum_{\nu} C^2_{\mu,A1}C^2_{\nu,R0}(\mu\mu|\nu\nu) && (48) \end{aligned}$$

with the neglect of differential overlap of atomic orbitals approximation.

Zeroth-order excited states Subsequently, to calculate $^2\mathrm{CT}_{\mathrm{Cz}}$–$^2\mathrm{LE}_{\mathrm{Ac}}$ coupling terms, the zeroth-order excited states for the acene fragments were obtained by diagonalisation of the CIS Hamiltonian for triplet states. The energy of the lowest triplet state of the acene fragment was used for $E(T_1)$, and the orbitals and eigenvalues of the self-consistent Fock matrix F_{A1A1} and $F_{A1'A1'}$ were carried forward for subsequent calculations. The excited doublet and quartet states for radical fragments at zeroth-order were similarly obtained by diagonalisation of the ExROPPP Hamiltonian, see Ref. 10,19. The energy of the first excited ExROPPP doublet state was taken as $E(D_1)$, and the orbitals and eigenvalues of the self-consistent Fock matrix F_{R0R0} (SOMO energy) and F_{R1R1} (HOMO energy) were carried forward for subsequent calculations. Interaction of the ground determinant with excited configurations was turned off in ExROPPP calculations so as not to introduce any correlation into the ground state which would lower its energy, to be consistent with CIS for closed-shell fragments.[20]

Calculation of $\langle ^2\mathrm{CT}_{\mathrm{Cz}} | \hat{H}^{(2)} | ^2\mathrm{LE}_{\mathrm{Ac}} \rangle$ coupling by 2nd order perturbation theory The energy of the $[R^- A^+]$ charge transfer states were approximated by the expression

$$E(\mathrm{CT}) = F_{R0,R0} - F_{A1,A1} + \frac{1}{2}J_{R0,R0} - J_{A1,R0} \tag{49}$$

using the integrals calculated earlier for acene and radical parts and $J_{A1,R0}$ which was also calculated in the zeroth-order orbitals. Finally, the coupling $\langle {}^2\mathrm{CT}_{\mathrm{Cz}} | \hat{H}^{(2)} | {}^2\mathrm{LE}_{\mathrm{Ac}} \rangle$ was evaluated using Eq. (31) with the Fock elements $F_{R0,A1'}$ and $F_{R1,A1}$ and energies of the D_1, T_1 and ${}^2\mathrm{CT}_{\mathrm{Ac}}$ states.

S2.4.3 PPP parameters

The same parameters were used for both closed-shell and radical parts, presented in Table 11 which were taken from our recent paper.[19] Hopping $t_{\mu\nu}$ terms are parameterised using an exponentially decaying function scaled by the cosine of the dihedral angle θ between two rings or groups

$$t_{\mu\nu} = A_{\mu\nu} \exp\left(-b_{\mu\nu} r_{\mu\nu}\right) \cos\theta, \tag{50}$$

using two independent parameters $A_{\mu\nu}$ and $b_{\mu\nu}$ which are atom pair dependent, where $r_{\mu\nu}$ is the scalar distance between atoms μ and ν.[19] Two-electron integrals are parameterised in the Mataga-Nishimoto scheme

$$\gamma_{\mu\nu} = \frac{\frac{1}{2}(U_\mu + U_\nu)}{1 + 2r_{\mu\nu}/(r_{0,\mu} + r_{0,\nu})} \tag{51}$$

where U_μ is the two-electron on-site Hubbard repulsion and $r_{0,\mu}$ is the distance scaling parameter.[21]

Suppl. Tab. 11: PPP parameters used for the ExROPPP simulations. These parameters have been optimised for predicting the excited states of organic radicals in previous work.[19]

Parameter	Value	Unit
ϵ_{C}	0	eV
ϵ_{N1}	−3.49	eV
ϵ_{N2}	−17.78	eV
ϵ_{Cl}	−10.34	eV
A_{CC}	−22.72	eV
A_{CN1}	−25.23	eV
A_{CN2}	−24.74	eV
A_{CCl}	−26.02	eV
b_{CC}	1.71	$Å^{-1}$
b_{CN1}	1.77	$Å^{-1}$
b_{CN2}	1.43	$Å^{-1}$
b_{CCl}	1.45	$Å^{-1}$
U_{C}	8.42	eV
U_{N1}	12.81	eV
U_{N2}	17.98	eV
U_{Cl}	9.64	eV
$r_{0,\mathrm{C}}$	1.17	Å
$r_{0,\mathrm{N1}}$	1.20	Å
$r_{0,\mathrm{N2}}$	1.11	Å
$r_{0,\mathrm{Cl}}$	2.25	Å

Quantum Chemical Calculations

Suppl. Tab. 12: T-An. Excitation energies, oscillator strengths, and expectation values of the S^2 operator are reported for toluene. Calculations were performed at the OT-SRSH TDA TDDFT ULC-ωhPBE/Def2-TZVP level of theory (ω = 0.088 Bohr^{-1}). Hole-particle natural transition orbitals for the entries highlighted in bold are shown in Suppl. Fig. 47.

	TOL			
	E (eV)	Osc. strength	$<S^2>$	Λ
$^{2,4}\mathbf{LE}_{Ac}$	**2.01**	**0**	**2.75**	**0.75**
$^{2}\mathbf{CT}_{Ac}$	**2.20**	**0.003**	**0.85**	**0.10**
	2.83	0.019	0.91	0.45
	2.84	0.022	0.92	0.53
	2.99	0.007	0.88	0.41

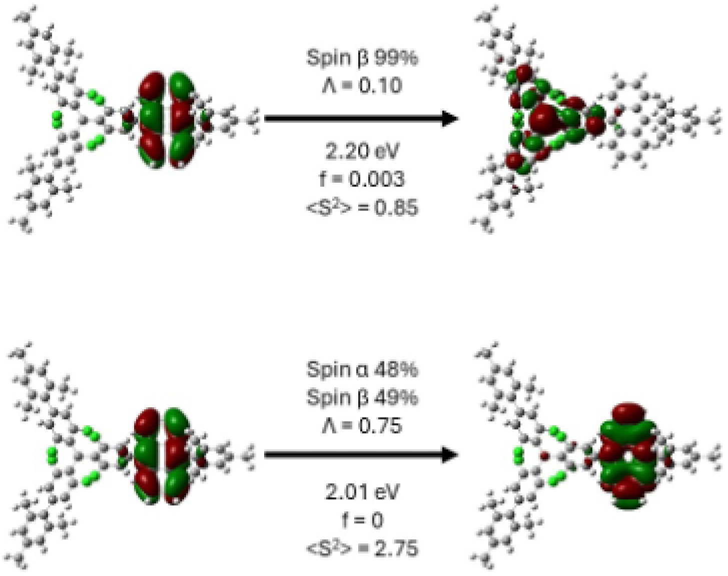

Suppl. Fig. 47: The T-An hole-particle natural transition orbitals as computed at the OT-SRSH TDA TDDFT ULC-ωhPBE/Def2-TZVP level in TOL.

Suppl. Tab. 13: An-T-1Cz. Excitation energies, oscillator strengths, and expectation values of the S^2 operator are reported for toluene and MeTHF. Calculations were performed at the OT-SRSH TDA TDDFT ULC-ωhPBE/Def2-TZVP level of theory (ω = 0.089 Bohr^{-1}). Hole-particle natural transition orbitals for the entries highlighted in bold are shown in Suppl. Fig. 48.

	TOL			
	E (eV)	Osc. strength	<S^2>	Λ
**^{2,4}LE$_{Ac}$**	**2.05**	**0.0003**	**2.66**	**0.78**
**^{2}CT$_{Ac}$**	**2.19**	**0.010**	**0.94**	**0.16**
**^{2}CT$_{Cz}$**	**2.27**	**0.081**	**0.87**	**0.30**
	2.76	0	0.84	0.10
	2.83	0.022	0.91	0.45

	MeTHF			
	E (eV)	Osc. strength	<S^2>	Λ
^{2}CT$_{Ac}$	1.98	0.005	1.07	0.20
^{2,4}LE$_{Ac}$	2.07	0.001	2.52	0.74
^{2}CT$_{Cz}$	2.12	0.079	0.85	0.30
	2.55	0	0.83	0.09
	2.79	0.021	0.90	0.44

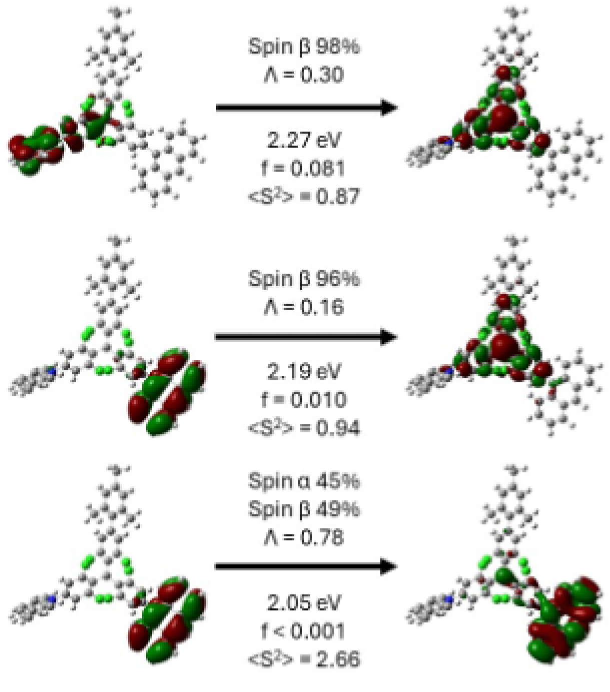

Suppl. Fig. 48: The An-T-1Cz hole-particle natural transition orbitals as computed at the OT-SRSH TDA TDDFT ULC-ωhPBE/Def2-TZVP level in TOL.

Suppl. Tab. 14: An-T-3PCz. Excitation energies, oscillator strengths, and expectation values of the S^2 operator are reported for toluene and MeTHF. Calculations were performed at the OT-SRSH TDA TDDFT ULC-ωhPBE/Def2-TZVP level of theory ($\omega = 0.086$ Bohr^{-1}). Hole-particle natural transition orbitals for the entries highlighted in bold are shown in Suppl. Fig. 49.

	TOL			
	E (eV)	Osc. strength	<S^2>	Λ
**^{2,4}LE$_{Ac}$**	**2.06**	**0**	**2.75**	**0.84**
**^{2}CT$_{Ac}$**	**2.28**	**0.007**	**0.90**	**0.30**
**^{2}CT$_{Cz}$**	**2.41**	**0.121**	**0.90**	**0.30**
	2.83	0.015	0.91	0.52
	2.88	0.002	0.89	0.40

	MeTHF			
	E (eV)	Osc. strength	<S^2>	Λ
^{2,4}LE$_{Ac}$	2.05	0.001	2.46	0.72
^{2}CT$_{Ac}$	2.11	0.004	1.14	0.23
^{2}CT$_{Cz}$	2.26	0.115	0.86	0.29
	2.73	0.004	0.007	0.27
	2.80	0.013	0.87	0.51

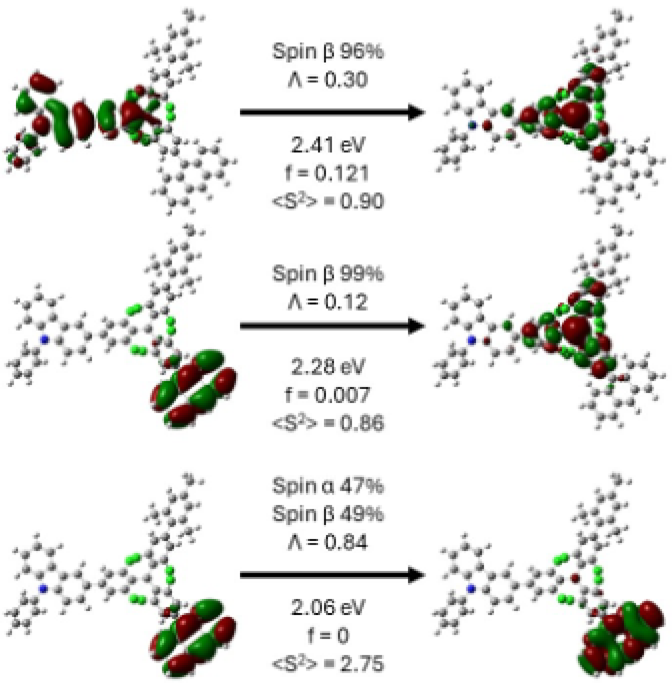

Suppl. Fig. 49: The An-T-3PCz hole-particle natural transition orbitals as computed at the OT-SRSH TDA TDDFT ULC-ωhPBE/Def2-TZVP level in TOL.

Suppl. Tab. 15: T-3Cz-An. Excitation energies, oscillator strengths, and expectation values of the S^2 operator are reported for toluene and MeTHF. Calculations were performed at the OT-SRSH TDA TDDFT ULC-ωhPBE/Def2-TZVP level of theory ($\omega = 0.098$ Bohr^{-1}). Hole-particle natural transition orbitals for the entries highlighted in bold are shown in Suppl. Fig. 50.

	TOL			
	E (eV)	Osc. strength	<S^2>	Λ
^{2,4}**LE**$_{Ac}$	**2.06**	**0**	**2.77**	**0.69**
^{2}**CT**$_{Cz}$	**2.34**	**0.130**	**0.89**	**0.26**
^{2}**CT**$_{Ac}$	**2.76**	**0.001**	**0.84**	**0.21**
	2.80	0.011	0.92	0.49
	2.85	0.006	1.62	0.27

	MeTHF			
	E (eV)	Osc. strength	<S^2>	Λ
^{2,4}LE$_{Ac}$	2.05	0	2.77	0.68
^{2}CT$_{Cz}$	2.14	0.113	0.85	0.24
^{2}CT$_{Ac}$	2.38	0.007	0.82	0.17
	2.59	0.007	0.84	0.21
	2.66	0.003	1.37	0.18

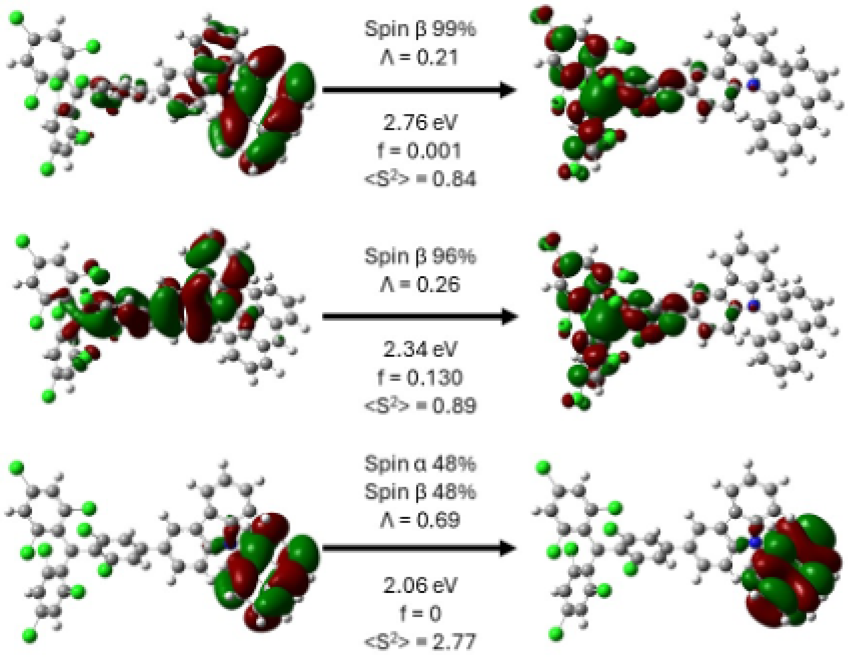

Suppl. Fig. 50: The T-3Cz-An hole-particle natural transition orbitals as computed at the OT-SRSH TDA TDDFT ULC-ωhPBE/Def2-TZVP level in TOL.

Suppl. Tab. 16: T-3Cz-Acr. Excitation energies, oscillator strengths, and expectation values of the S^2 operator are reported for toluene and MeTHF. Calculations were performed at the OT-SRSH TDA TDDFT ULC-ωhPBE/Def2-TZVP level of theory ($\omega = 0.098$ Bohr^{-1}). Hole-particle natural transition orbitals for the entries highlighted in bold are shown in Suppl. Fig. 51.

	TOL			
	E(eV)	Osc. strength	$\langle S^2 \rangle$	Λ
^{2,4}**LE**$_{Ac}$	**2.13**	**0**	**2.77**	**0.69**
^{2}**CT**$_{Cz}$	**2.41**	**0.128**	**0.90**	**0.31**
	2.67	0.018	1.67	0.29
	2.79	0.013	1.67	0.41
	2.80	0.012	0.93	0.51
	...			
^{2}**CT**$_{Ac}$	**3.12**	**0.005**	**1.11**	**0.27**

	MeTHF			
	E (eV)	Osc. strength	$\langle S^2 \rangle$	Λ
^{2,4}LE$_{Ac}$	2.12	0.0001	2.76	0.68
^{2}CT$_{Cz}$	2.23	0.117	0.86	0.29
	2.47	0.011	1.31	0.20
^{2}CT$_{Ac}$	2.64	0.009	0.85	0.20
	2.67	0.009	1.79	0.42

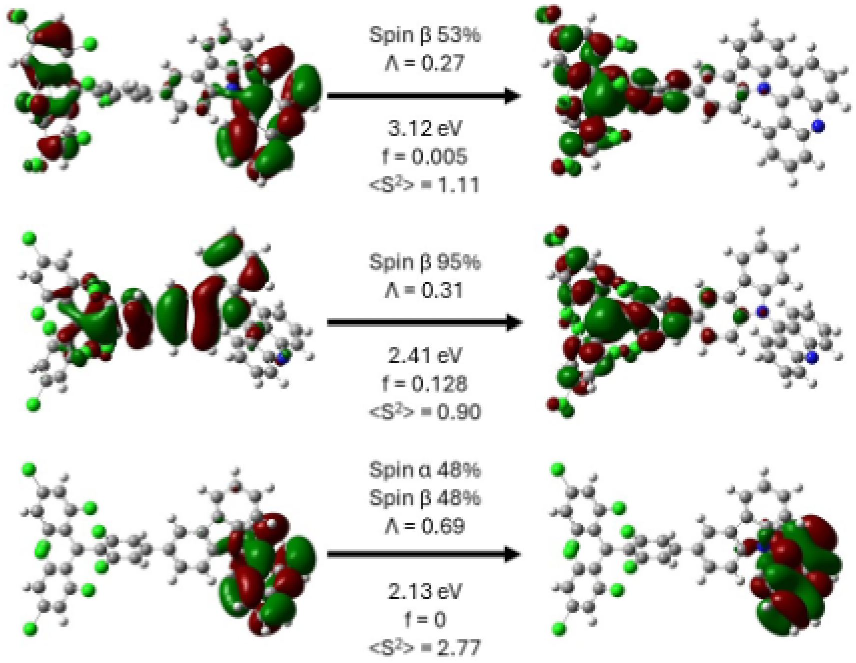

Suppl. Fig. 51: The T-3Cz-Acr hole-particle natural transition orbitals as computed at the OT-SRSH TDA TDDFT ULC-ωhPBE/Def2-TZVP level in TOL.

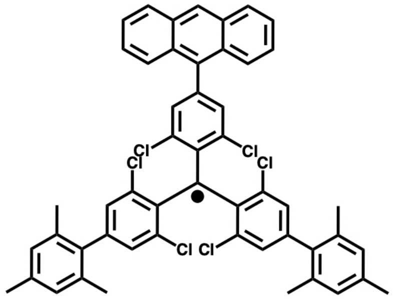

	TOL			
	Energy (eV)	Osc. strength	<S²>	Λ
**$^{2,4}LE_{Ac}$**	**2.06**	**1x10^{-4}**	**2.71**	**0.76**
**$^{2}CT_{Ac}$**	**2.26**	**0.008**	**0.89**	**015**
	2.82	0.019	0.91	0.48
	2.84	0.016	0.92	0.53
	2.99	0.007	0.88	0.42

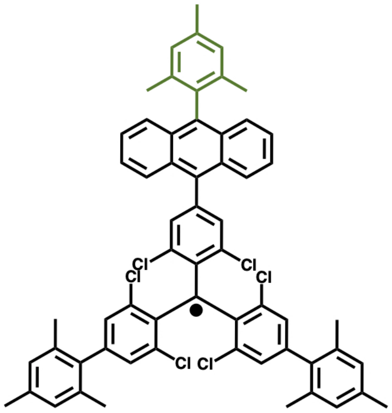

	TOL			
	Energy (eV)	Osc. strength	<S²>	Λ
**$^{2,4}LE_{Ac}$**	**2.01**	**0**	**2.75**	**0.75**
**$^{2}CT_{Ac}$**	**2.20**	**0.003**	**0.85**	**0.10**
	2.83	0.019	0.91	0.45
	2.84	0.022	0.92	0.53
	2.99	0.007	0.88	0.41

T-An

as synthesised

Suppl. Fig. 52: Effect of mesitylation of anthracene on electronic properties. Excitation energies, oscillator strengths, and expectation values of the S^2 operator are reported for toluene. Calculations were performed at the OT-SRSH TDA TDDFT ULC-ωhPBE/Def2-TZVP level of theory (ω = 0.088 Bohr^{-1}).

NMR Spectra

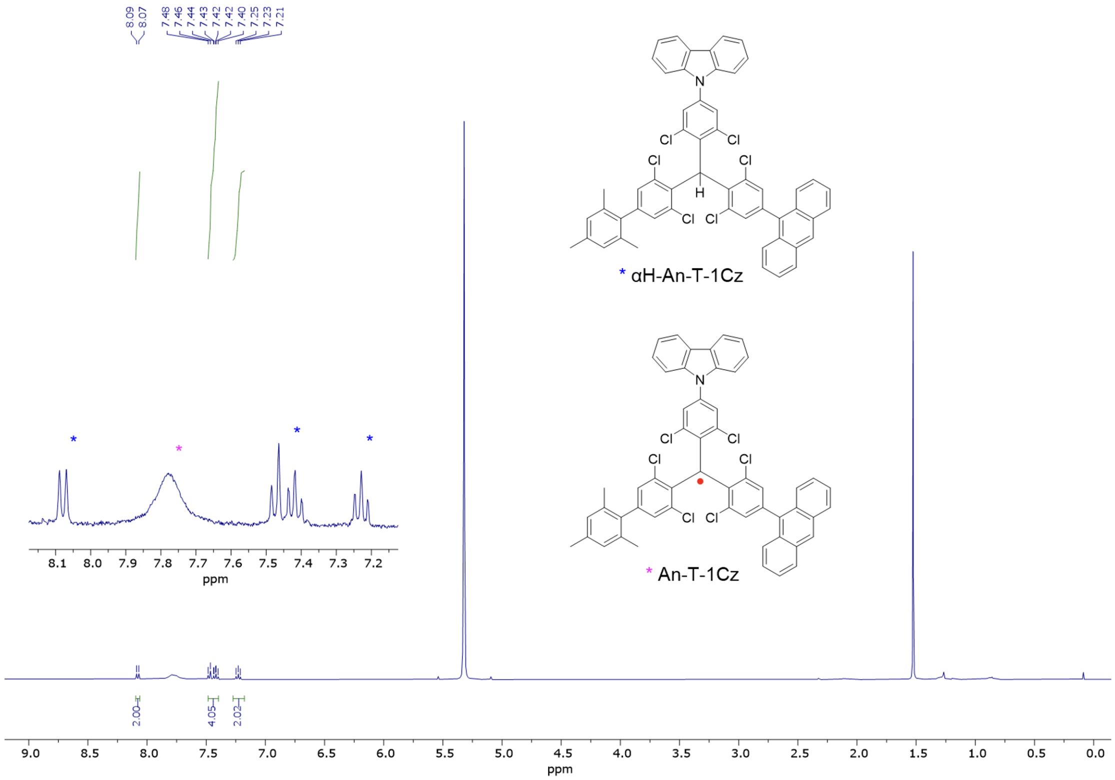

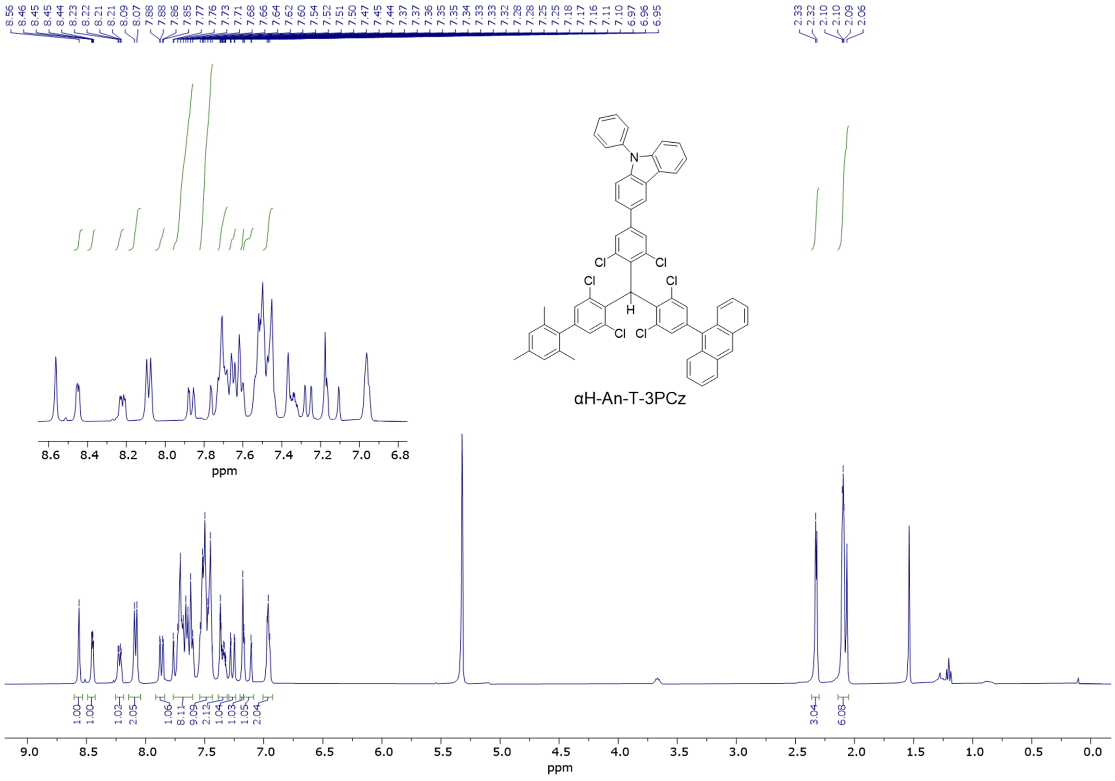

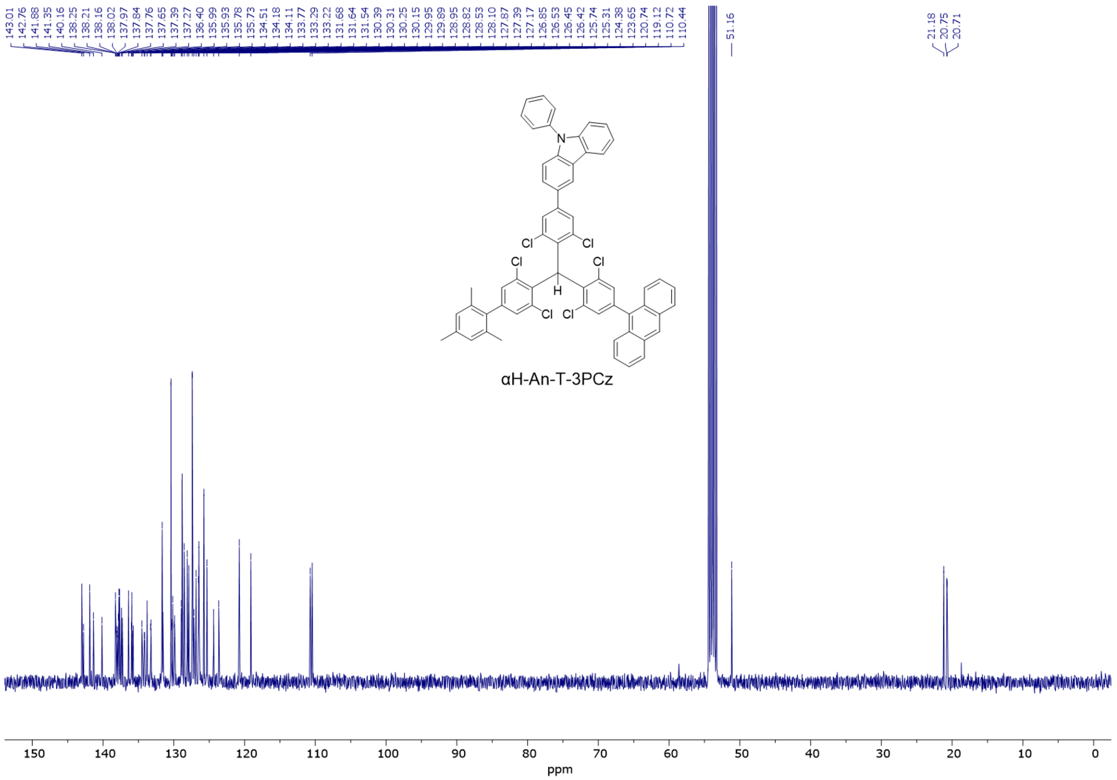
αH-An-T-3PCz
ppm

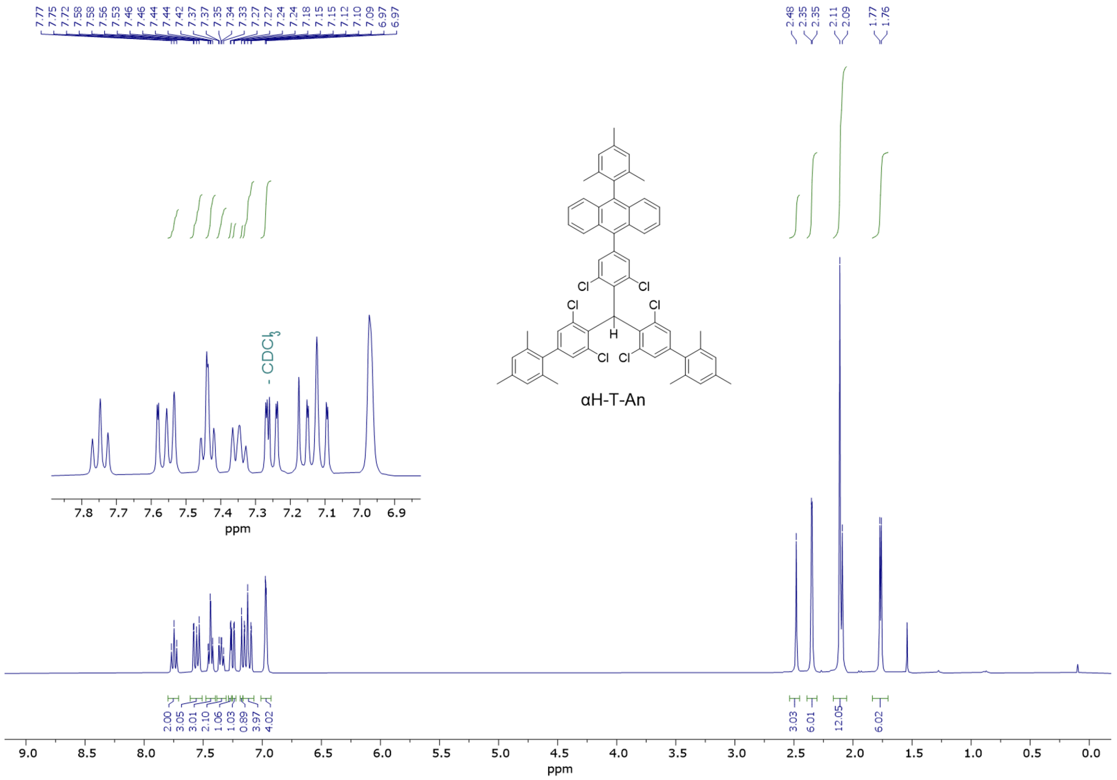
αH-T-An
- CDCl3
ppm
ppm

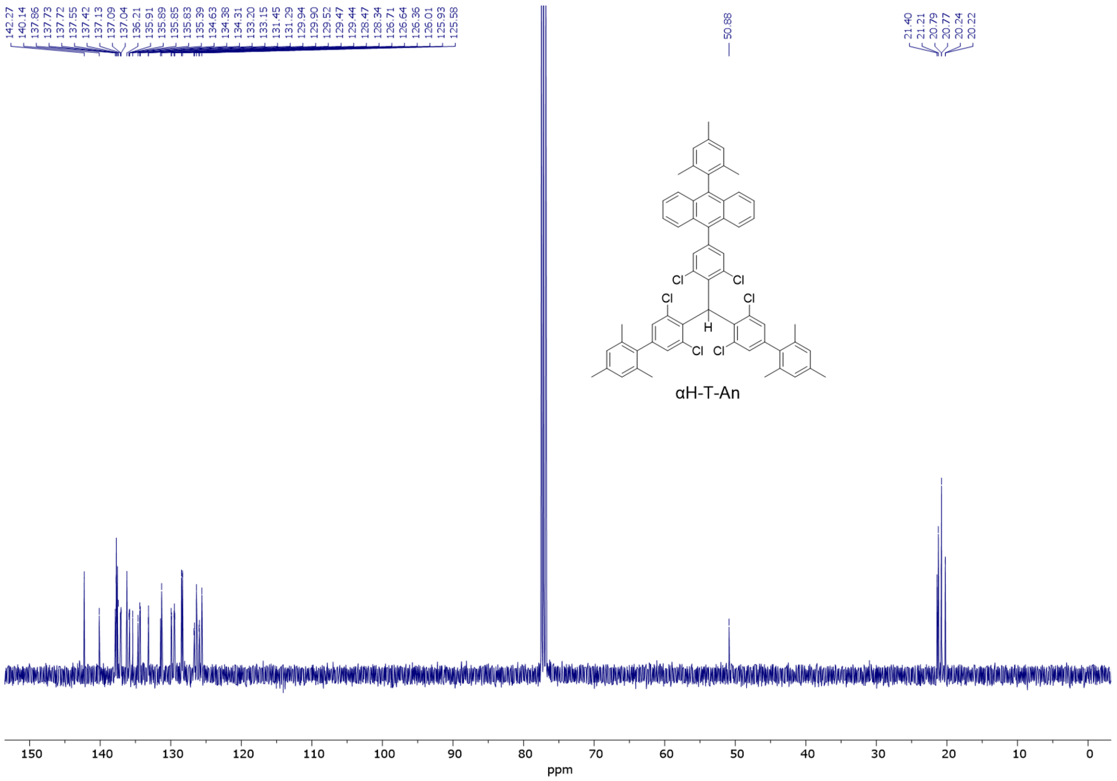

142.27
140.14
137.86
137.73
137.72
137.55
137.42
137.13
137.09
137.04
136.21
135.91
135.89
135.85
135.83
135.39
134.63
134.38
134.31
133.20
133.15
131.45
131.29
129.94
129.90
129.52
129.47
129.44
128.47
128.34
126.71
126.64
126.36
126.01
125.93
125.58
50.88
21.40
21.21
20.79
20.77
20.24
20.22
Cl
Cl
Cl
Cl
H
Cl
Cl
αH-T-An
150
140
130
120
110
100
90
80
70
60
50
40
30
20
10
0
ppm

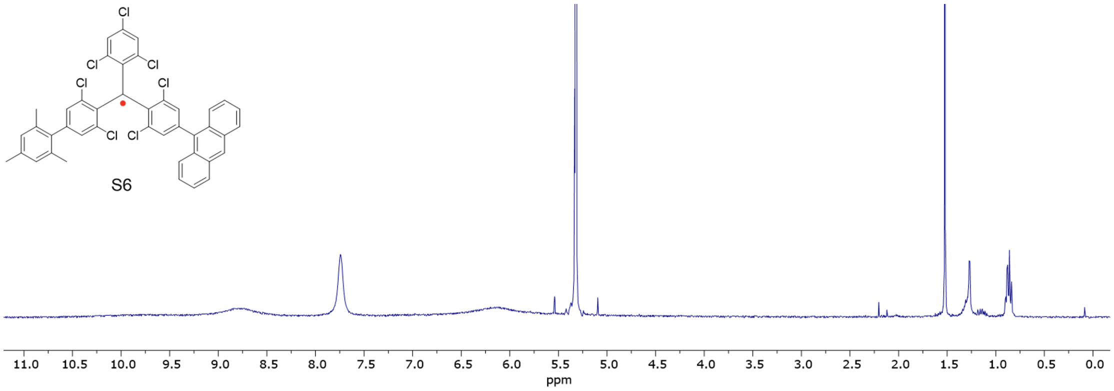
S6
ppm

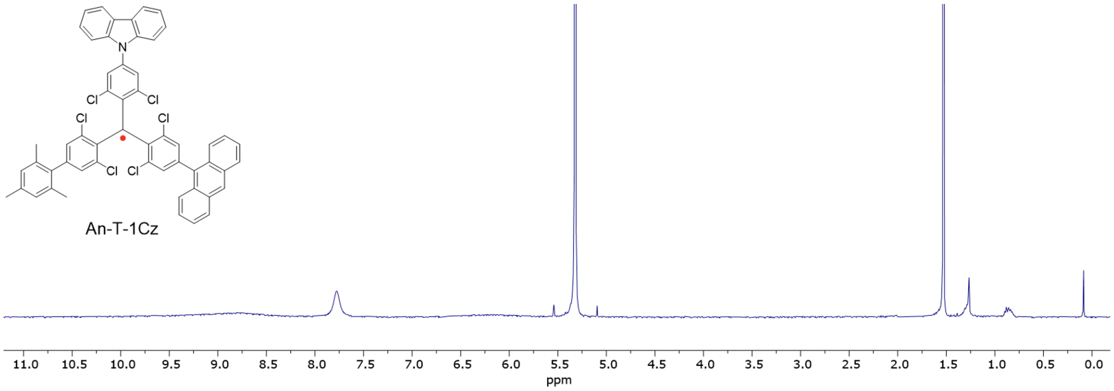
An-T-1Cz
ppm

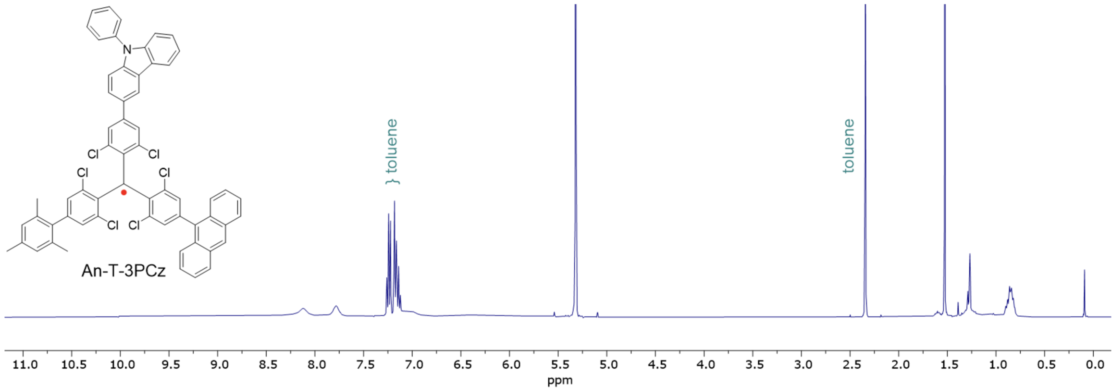
An-T-3PCz
toluene
toluene
ppm

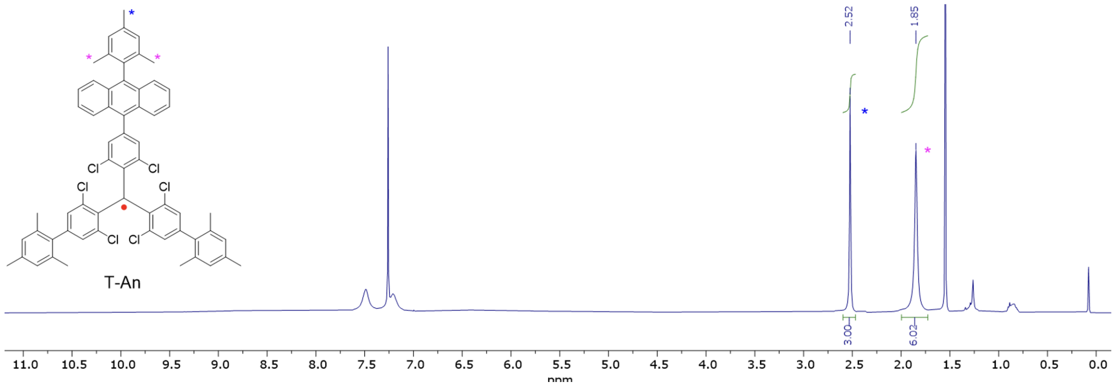
T-An
2.52
1.85
3.00
6.02

Supplementary References

(1) Murto, P. *et al.* Mesitylated trityl radicals, a platform for doublet emission: Symmetry breaking, charge-transfer states and conjugated polymers. *Nature Communications* **14**, 4147 (2023).

(2) Peng, Q., Obolda, A., Zhang, M. & Li, F. Organic light-emitting diodes using a neutral π radical as emitter: the emission from a doublet. *Angewandte Chemie* **127**, 7197–7201 (2015).

(3) Roques, N. *et al.* Three-Dimensional Six-Connecting Organic Building Blocks Based on Polychlorotriphenylmethyl Units—Synthesis, Self-Assembly, and Magnetic Properties. *Chemistry – A European Journal* **12**, 9238–9253 (2006).

(4) Lee, J. *et al.* Excimer emission based on the control of molecular structure and intermolecular interactions. *Journal of Materials Chemistry C* **4**, 2784–2792 (2016).

(5) Shapiro, N. D., Rauniyar, V., Hamilton, G. L., Wu, J. & Toste, F. D. Asymmetric additions to dienes catalysed by a dithiophosphoric acid. *Nature* **470**, 245–249 (2011).

(6) Boyd, T. J. & Schrock, R. R. Sulfonation and Epoxidation of Substituted Polynorbornenes and Construction of Light-Emitting Devices. *Macromolecules* **32**, 6608–6618 (1999).

(7) Yan, C. *et al.* Stable Diarylamine-Substituted Tris(2,4,6-trichlorophenyl)methyl Radicals: One-Step Synthesis, Near-Infrared Emission, and Redox Chemistry. *CCS Chemistry* **4**, 3190–3203 (2021).

(8) Murto, P. *et al.* Steric Control of Luminescence in Phenyl-Substituted Trityl Radicals. *Journal of the American Chemical Society* **146**, 13133–13141 (2024).

(9) Weller, A. Photoinduced electron transfer in solution: Exciplex and radical ion pair formation free enthalpies and their solvent dependence. *Zeitschrift für Physikalische Chemie* **133**, 93–98 (1982).

(10) Green, J. D. & Hele, T. J. H. ExROPPP: Fast, accurate, and spin-pure calculation of the electronically excited states of organic hydrocarbon radicals. *The Journal of Chemical Physics* **160**, 164110 (2024).

(11) Green, J. D., Fuemmeler, E. G. & Hele, T. J. H. Inverse molecular design from first principles: Tailoring organic chromophore spectra for optoelectronic applications. *The Journal of Chemical Physics* **156** (2022).

(12) Hele, T. J. H. On the electronic structure of alternant conjugated organic radicals for light-emitting diode applications. In *Physical Chemistry of Semiconductor Materials and Interfaces XX*, vol. 11799, 117991A. International Society for Optics and Photonics (SPIE, 2021).

(13) Gorgon, S. *et al.* Reversible spin-optical interface in luminescent organic radicals. *Nature* **620**, 538–544 (2023).

(14) Pople, J. A. Electron interaction in unsaturated hydrocarbons. *Trans. Faraday Soc.* **49**, 1375–1385 (1953).

(15) Pariser, R. & Parr, R. G. A Semi-Empirical Theory of the Electronic Spectra and Electronic Structure of Complex Unsaturated Molecules. I. *The Journal of Chemical Physics* **21**, 466–471 (1953).

(16) Pople, J. A. The electronic spectra of aromatic molecules ii: A theoretical treatment of excited states of alternant hydrocarbon molecules based on self-consistent molecular orbitals. *Proceedings of the Physical Society. Section A* **68**, 81 (1955).

(17) Pariser, R. Theory of the Electronic Spectra and Structure of the Polyacenes and of Alternant Hydrocarbons. *The Journal of Chemical Physics* **24**, 250–268 (1956).

(18) Szabo, A. & Ostlund, N. *Modern Quantum Chemistry: Introduction to Advanced Electronic Structure Theory*. Dover Books on Chemistry (Dover Publications, 1989).

(19) Shen, J. *et al.* Learning radical excited states from sparse data. *Chem. Sci.* **16**, 17356–17368 (2025).

(20) Maurice, D. & Head-Gordon, M. On the nature of electronic transitions in radicals: An extended single excitation configuration interaction method. *The Journal of Physical Chemistry* **100**, 6131–6137 (1996).

(21) Mataga, N. & Nishimoto, K. Electronic structure and spectra of nitrogen heterocycles. *Zeitschrift für Physikalische Chemie* **13**, 140–157 (1957).
