# Supplementary material for: Coulombic control of charge transfer in radicals with quartet recycling luminescence
Source: Nat Commun. 2026 May 22;17:7203. doi: 10.1038/s41467-026-72487-5 (PMC13396479; doi:10.1038/s41467-026-72487-5)
Supplement: Supplementary file 3 — Transparent Peer Review File [file 41467_2026_72487_MOESM3_ESM.pdf]

nature portfolio

Peer Review File

Coulombic control of charge transfer in radicals with quartet recycling luminescence

Corresponding Author: Dr Sebastian Gorgon

This file contains all reviewer reports in order by version, followed by all author rebuttals in order by version.

Version 0:

Reviewer comments:

Reviewer #1

(Remarks to the Author)
In this work, the authors explore the role of charge-transfer states on the mechanism of quartet formation and delayed luminescence in TTM-acene dyads. The primary focus is placed on how the radical-acene electronic coupling impacts the energetics of the CT state, which is proposed to act as a trap that non-radiatively deactivates the excited state. The TTM radical is of high interest in a broad range of different fields for its unique excited state properties. This work could potentially provide some much needed design principles for tuning the excited state behavior of TTM radicals.
However, many of the central claims made in the manuscript are only vaguely supported by the spectroscopic data, and some claims appear to have no evidence at all. Much of the data is also poorly analyzed. As a result, the presentation and discussion of the data is difficult to follow at times, and the conclusions made by the end of the manuscript are uncertain. In its current state, we believe the manuscript is not acceptable for publication in Nature Communications unless substantial revisions are made.
Comments and questions are listed below to justify our decision.

Major Concerns

1.) Many arguments in the manuscript are based on relative electronic coupling strengths between the TTM and anthracene sub-units, but only qualitative and unspecific discussion of the couplings is given. For example, the authors should define exactly what they mean when they invoke electronic couplings, as this interaction can manifest in many different ways. The design principles derived from this work would be much stronger if the electronic couplings could also be quantified. For example, what range would be designated “weak” as opposed to “strong” couplings? In this vein, comparing the electronic absorption spectra of the dyads with their corresponding monomer subunits would be extremely helpful for getting a sense of the change in electronic couplings.

2.) The author’s assignment of delayed emission in some instances is concerning. For example, it is clear from the bottom two panels of Figure S11 that T-3Cz-An and T-3Cz-Acr exhibit prompt and delayed emission originating from the same state. However, the emission profiles in the top two panels for An-T-1Cz and An-T-3PCz red-shift with time, possibly indicating the presence of a separate emitting state. This would not be “delayed emission” in the traditional sense, where the same emission channel is thermally repopulated. This issue appears to be worse for the data in THF in Figure S13. A brief explanation for the band shifting is given in the figure caption, but it is largely insufficient and does not address why the profile broadens so much in THF versus toluene. Does the emission spectrum of bare TTM-3Cz red-shift and broaden like this in THF? If not, given that the spectral shape is so different, is it reasonable to conclude that emission from a separate state (possibly CTAc) is being observed?

3.) Similar to the last point, the temperature-dependent “delayed emission” assignments in PMMA (Figures S14-17) are even more concerning. The authors acknowledge the gradual blue-shift of the spectra at later times in the main text, but do not address the drastic change in the spectral shape at later times compared to the prompt spectra (see Figure S14 in particular). It is more justifiable to assign these two emission components to entirely different species rather than the proposed delayed emission. It is possible that there is significant aggregation occurring in these films given the large radical loadings (5 w/w%), which may result in this broadened emission. The authors should provide a rigorous explanation for this inconsistency and why they have chosen to assign this as delayed emission. The energy barriers for delayed emission are also calculated from this data, so the corresponding Arrhenius plots for all compounds should be shown in the SI.

4.) The band assignments in the TA data are hard to follow, mainly because CTCz and CTAc both have broad absorbance bands from ~600-700 nm. For example, Figures 2b,e assign the band at ~625 nm to CTAc, even though it is very shifted from the assignment made for CTAc in Figure 2a and more in line with the band assigned to CTCz in Figures 2a,d. The exact data that is assigned to CTAc in Figures 2b,e is also assigned to CTCz in the figure caption of Figure S19. The authors should check the TA data and provide better explanation of the final assignments. For the CTAc state, there should be a TTM-Cz anion absorption band in addition to the anthracene cation absorption at ~650-700 nm. Is this anion spectrum known? If not, the authors should conduct spectro-electrochemistry experiments if available to help clarify the data. Finally, a significant amount of TA data is presented, yet there is very limited kinetic analysis. Can the authors specify why they have chosen not to analyze the kinetics? A basic kinetic analysis would likely help clarify the band assignments and mechanism. In particular, it is strange that the TA features all decay in 100 ps for An-T-1Cz and An-T-3PCz, yet photoluminescence with a 3-8% yield and ~16 ns prompt lifetime is observed for these compounds.

5.) The simulated trESR spectra of An-T-1Cz and T-3Cz-Acr should also be shown in the SI and included fits in Figure 3 don't seem particularly good. The experimental absorptive features seem considerably more intense than the emissive. On a related note, the simulation procedure is not described with enough detail other than a brief reference to the EasySpin simulation paper by Tait et. al. Enough information should be included in the methods section or the SI to reproduce the simulations. For example: what spin Hamiltonian was used; what doublet-triplet exchange couplings were used; how were the different orientations of the triplet zero-field splitting tensor and triplet-radical dipolar interaction tensors accounted for when calculating DQ, EQ, and DTR; the sublevel populations seem arbitrary at best, how were they determined, are they related to the mechanism?

6.) Many of the arguments made in the manuscript invoke energetic orderings of the various electronic states and their proximity to one another. However, the only detailed investigation of electronic state energies is based on TD-DFT calculations instead of experimental data. I am always hesitant to trust such arguments given the difficulties with DFT predicting relative energies of localized versus CT states. For example, the calculations in Table 3 overestimate the 2,4LE energy by ~0.2 eV based on the triplet energy of anthracene, while they overestimate the CTCz energy by ~0.4-0.5 eV based on the optical data in Figure S10. Were attempts made to experimentally determine the various CTAc state energies, such as with donor-acceptor redox potentials and the Weller equation? The energy of the CTCz state should be estimated from the steady-state optical data in Figure S10 and explicitly stated in the manuscript/SI.

7.) Discussion is given on lines 185-192 about the requirements for efficient ISC/RISC in these systems, however, there are many inconsistencies in the claims. First, the authors state that efficient SO-ISC requires states with different spatial wavefunctions, which is not necessarily true. It is often the case that weak SOCs between states with similar spatial wavefunctions is overcome by a very small energy gap between the states, leading to rapid ISC (such as transitions between singlet and triplet n π* states, where J is also small). Vibrationally-assisted ISC is also commonly seen to overcome weak SOCs, which is the known ISC mechanism in bare anthracene. As a result, it is not apparent why the CTAc state is being invoked as an intermediate to ISC/RISC, and the authors should further clarify if possible. Regardless, the authors claim that for the CTAc state to mediate ISC/RISC, it must be thermally accessible from the 4LE and CTCz states and concludes that this is the case for T-3Cz-An on line 191. However, the TD-DFT calculations in Table 3 show that CTAc is 0.4 eV above CTCz for T-3Cz-An in toluene, which is >15x thermal energy at room temperature. Significant thermal population of CTAc under these conditions is unrealistic, which necessarily means that either the mechanistic discussion provided on lines 185-192 is incorrect, or the energies calculated by TD-DFT are inaccurate. Since the energetics are not well understood in general (see point #6 above), it is recommended that lines 185-192 and all other related discussion be removed from the manuscript unless the energies are better quantified.

8.) On page S45 of the SI, the authors define the exchange integral, however, there appears to be a mistake in the expression. The position vectors are written in the order r1r1(1/r12)r2r2, but it should be written as r1r2(1/r12)r2r1 to represent an exchange integral. The authors should check to make sure if this is a mistake and fix the ensuing derivations. For example, this same ordering is given in equation 23 and in the first paragraph of page S47.

9.) On the bottom of page S53, the authors caution that their derivations are not accurate for systems where the dyad orbitals contain significant mixed contributions from both the acene and radical. However, the authors claim that the equations should apply to the molecules in this work. Can more reasoning be given for this? This would appear to be counterintuitive to the claims made throughout the manuscript that An-T-1Cz and An-T-3PCz exhibit strong acene-radical electronic coupling, and therefore their frontier orbitals should mix fairly significantly. This is another reason why it is important to define what is meant by "electronic couplings" and have estimates of the electronic coupling strengths between the relevant orbitals. Additionally, just from inspection of the NTOs in Figures S28-31, there seems to be quite a bit of delocalization of the dyad orbitals onto both the radical and acene fragments.

Minor comments

10.) The authors state on line 20 that the PLQYs of defects are typically low. What do they authors mean by low, plenty of reports as early as 1997 (Science 1997, 276, 2012) have demonstrated single-particle photoluminescence imaging and single photon emission from NV- centers which requires high PLQYs. Additionally, ref. #2 that is cited in the previous sentence includes discussion about the large PLQYs of defects in diamond.

11.) On line 27, the authors should be careful making the statement that typical quartet-forming chromophore-radical systems are non-luminescent. In at least one recent report they have been shown to be luminescent (Angew. Chem. 2023, 135, e202214668), the yields are just low since EISC and EIC are fast compared to radiative decay of the photoexcited

chromophore. Further, there are reports by Kandrashkin (J. Phys. Chem. A 2006, 110, 9607-9616) and Gouterman (J. Chem. Phys. 1970, 52, 3795-3802) that demonstrate luminescence directly from the 2,4LE states with moderate yields. These points should be addressed in the manuscript, and the statement on line 27 rephrased with them in mind.

12.) On line 31, the authors state that only a single design of TTM-chromophore is known, again the authors should be careful in making these claims. One recent example (Chem. Eur. J. 2024, 30, e202403244) even explored the structure-property relationship; a different one (J. Am. Chem. Soc. 2025, 147, 1, 1017), explored and explained in far better detail the mechanism proposed in this manuscript.

13.) The doublet-triplet resonance condition and why it's important should be explained in a sentence in the introduction. Otherwise, it is not clear why the resonance condition is important until the very end of the manuscript, which may make some of the earlier discussion difficult to follow for readers that are not familiar with the work in ref. #10.

14.) A reference to Figure 1d is made in the text, however, there is no panel D.

15.) Is there a reason the authors have chosen to mesitylate An-T-1Cz, An-T-3PCz, and T-An, but not T-3Cz-An and T-3Cz-Acr? On a related note, a reference to the T-An structure in the SI should be made on line 53, otherwise it was very difficult to find the structure. Additionally, why did the authors choose to mesitylate the 10-position of anthracene in T-An instead of leaving it bare like in all of the dyads discussed in the main text? This will certainly impact the electronics of the anthracene, which counters the purpose of using the compound as a direct control.

16.) The authors claim on line 61 that the dominance of the RISC channel is desired to allow time for quartet addressability, which does not seem to be correct. The main advantage of this pathway is that it maximizes the PLQY of the radiative channel while still forming high yields of quartet, which typically quenches emission (see point #11). The "time for quartet addressability" is only dictated by the quartet lifetime and T1 relaxation, which will necessarily always be short as long as RISC is efficient and competitive. Can the authors clarify?

17.) Line 72, what is the justification for the claim that conformationally locking the molecules in a rigid medium would lead to a time-dependent blue-shift of the emission as opposed to a static blue-shift, which is what is typically observed?

18.) Also line 72, the authors say "effective lifetime" but do not define what this is. This also appears in Tables S1-3, but I cannot find if it is mathematically defined anywhere.

19.) The authors claim in lines 86-87 that An-T-1Cz, An-T-3PCz, and T-An decay mostly from states of similar character. However, it is clear that the spectral features of T-An are quite different from the other two compounds. In Figure S18a, what are the TA features for T-An being assigned to? If the feature at ~625 is assigned to anthracene cation, why is it so shifted from the anthracene cation features (~700 nm) for An-T-1Cz and An-T-3PCz in Figures S18b,c? This point illustrates the need to perform spectro-electrochemistry on the compounds to correctly assign each ion band.

20.) On line 109, it says that "excited state" signals were not observed for T-An in the trESR data. The way this statement reads is a bit unclear because it does not rule out the observation of ground state polarization. The authors should clarify if no signal at all is observed.

21.) The inversion of the trESR signals at later times is likely due to a combination of spin-selective depopulation of the quartet sublevels, T1e relaxation processes, as well as saturation driven by the cw microwaves. Is the temperature-dependence of the population inversion only being ascribed to the RISC mechanism as with the delayed emission? If so, how do you know it doesn't have significant contributions from T1e decay between the sublevels as well?

22.) Lines 122-123, the authors assign the initial absorptive feature in the trESR spectrum of T-3Cz-An to population of the CTAc state during ISC to from 2,4LE. The evidence for this is the "similarities with timescales" for the optical and trESR data. This claim provides no rational and does not make sense. First, optical data shows a 4 ps rise of the 2,4LE feature in the TA (Figure 2d, line 92). The trESR experiment does not have sub-nanosecond resolution to observe this, IRF is likely closer to a 50-100 ns. The ~4 ps component would need slow down by ~10,000x at 80 K in order to be resolved by trESR. This is unlikely, but regardless, TA data was not collected at 80 K to provide evidence for this. Second, as stated above, it is unclear why the CTAc state is being invoked as an ISC intermediate in T-3Cz-An when there is no spectral evidence of its population and TD-DFT calculates it to be ~0.4 eV higher in energy than CTCz in toluene (Table 3).

23.) Line 124, the authors reference Figure S25 for their claim of high quartet yields, but that figure in the SI has nothing to do with quartet yields, it simply verifies the quartet state by displaying the double and triple quantum transitions. Otherwise, this figure isn't referenced in the main text, but it should be because it is some of the strongest evidence for quartet formation in these systems.

24.) Why was pulse-ESR only performed on T-3Cz-An and not T-3Cz-Acr? The other two dyads have poor S/N in trESR so it is understandable why it was not done for them.

25.) Line 132, there appears to be a typo. I believe the compound should be written as "T-3Cz-An" and not "T-An-3Cz."

26.) A general comment, the optical data would be much easier to follow if the TD-DFT calculations or other discussion of state energetics were presented first.

27.) Lines 174-176, the CTAc state is again invoked as a mediator for conversion of 2D1S0 to 2D0T1, although this time it is claimed that it does so “indirectly.” It is not clear how this fits into the rest of the discussion in the manuscript, where it is implied that CTAc is directly populated. Again, given that the energetics of these states are not thoroughly investigated, it is advised that the authors remove this and related discussion from the manuscript unless the energetics can be more reliably quantified, or more rigorous evidence is provided for the involvement of CTAc in the ISC/RISC mechanism.

28.) Line 201, the authors claim that the molecular topology modulates electronic rather than exchange couplings. However, exchange couplings are not calculated or measured in these systems, so this claim should be removed from the manuscript. Particularly because it is well known that molecular connectivity and orbital overlap drastically changes exchange couplings. On a similar note, what evidence is there for the claim on line 203 that strong exchange couplings can be maintained for distances exceeding 2 nm? This seems like an arbitrary statement.

29.) In the caption of Figure 4, the CTAc NTOs are discussed and the statement is made that hole-particle delocalization increases from An-T-3PCz to T-3Cz-An, which in turn enhances electronic coupling to other states through wavefunction overlap. This contradicts the claim made throughout the paper that T-3Cz-An has the weakest electronic coupling and is the reason why ISC/RISC is so effective. The authors should be clear with definitions of the various electronic couplings they discuss in the paper, as this may just be a case where two different types of electronic couplings are being invoked.

30.) What CW microwave power was used to collect trESR data? This should be specified in the methods section.

31.) At what time, following laser excitation, were the reported trESR spectra collected?

32.) Figure S11, top right panel. I believe the compound should read “An-T-3PCz” and not “An-T-3Cz”

33.) Why is the temperature-dependent emission data in PMMA for T-3Cz-Acr not shown in the SI as well?

34.) Figure S24 caption, I believe the compound should read “An-T-1Cz” and not “An-T-3Cz”

35.) Figure S26 caption, define “DAF”

36.) When NTOs are displayed, a corresponding λ value should be shown that designates the contribution of that hole-particle pair to the total excitation.

Reviewer #2

(Remarks to the Author)
Quartet states (S = 3/2), enabled by radical-triplet interactions, merge high-spin quantum properties with potential applications in quantum sensing and optoelectronics. However, such states are typically non-emissive, and only a few luminescent radical-chromophore dyads (e.g., TTM derivatives) are known to demonstrate stable quartet generation. As a result, a general structure-property design principle remains elusive, making the engineering of robust quartet systems a critical challenge.
In this work, Matasovic and coworkers designed two classes of compounds: one exhibiting weak radical-chromophore coupling and the other demonstrating strong coupling. The strongly coupled system achieves modest PLQY, while one of the weakly coupled variants (T-3Cz-An) displays predominantly delayed emission and remarkably slow quartet depletion dynamics. Based on TD-DFT calculations, the authors found that in weakly coupled systems, a bright carbazole-to-TTM charge-transfer state (2CTCz) lies above the quartet state. However, in strongly coupled systems, a dark acene-to-TTM charge-transfer state (2CTAc) lies above the quartet state and acts as a dark trap, significantly reducing efficiency. Using these insights, the authors developed general energetic guidelines to optimize quartet state generation in luminescent radical-chromophore systems. This work is very interesting and important! Before publication, the authors could need to address the following concerns.
1. The main text references 'Fig. 1d' in the discussion, but this panel does not appear in the provided Figure 1.
2. In T-3Cz-An, the authors observe a significant drop in delayed emission lifetimes (~10x) when moving from toluene to a polar solvent (MeTHF), which they initially attribute to excited-state energy reordering. However, their theoretical calculations suggest the polar solvent does not alter the relative ordering of states (2CTAc still remains ~0.24 eV above 2CTCz). If 2CTAc is still significantly higher in energy, its ability to act as an effective dark trap and strongly quench delayed emission becomes unclear and require further justification. Moreover, in T-3Cz-Acr, the 2CTCz - 2CTAc gap shrinks from 0.7 eV (toluene) to 0.4 eV (MeTHF), it needs to be clarified whether this reduced gap similarly influences the delayed lifetime. In general, it is essential to clarify how molecular topology tunes energy gaps and non-radiative decay for understanding structure-property relationship in this case.
3. Based on perturbation theory, the authors suggest that large orbital exchange interactions are beneficial for stabilizing the quartet state relative to the emissive radical state. Additionally, they propose that the energy of the 2CTAc state can be raised by designing systems with spatially separated radical SOMO and acene HOMO, alongside spatially proximate acene HOMO and LUMO. While these design principles may be theoretically useful, the manuscript would benefit from their further validation on the molecules discussed in this study. In particular, calculating the exchange and Coulombic interactions for the two classes of molecules would help substantiate the proposed energetic criteria.

Reviewer #3

(Remarks to the Author)
I co-reviewed this manuscript with one of the reviewers who provided the listed reports. This is part of the Nature Communications initiative to facilitate training in peer review and to provide appropriate recognition for Early Career Researchers who co-review manuscripts.

Reviewer #4

(Remarks to the Author)
In this work, the authors have reported syntheses and excited-state dynamics of luminescent radicals with long-lived quartet states and proposed a strategy for compounds with microwave-addressable high spin states. The clarification of the photo-excited states including charge-transfer states was carried out their prompt and delayed photoluminescence, transient absorption measurements, transient ESR, and pulse ESR with help of the computational work. The authors have revealed the role of molecular topology in luminescent quantum materials, which leads to the coulombic control of the charge transfer states. I consider that this work is very interesting and contains new important findings. However, there are some points that should be addressed. Therefore, I recommend publication after the revisions. Please consider the following points.

1) Comparison the photophysical data between solution and PMMA films is important as well as the solvent polarity dependence. Please add the photophysical data in PMMA in Table 1 if it is possible.

2) Authors describe: "The effective lifetime extends to around 2.3μs for films of T-3Cz-An. In contrast, it shortens in films of An-T-3PCz, indicating the promotion of deactivation channels in a more rigid environment." I could not find out the corresponding data in the supplemental information. In Table S2, solvent dependence are given only in the Toluene and THF. Please describe more clearly the data in PMMA of T-3Cz-An and An-T-3PCz like Table S2.

3) The definite assignment of the transient absorption spectra is crucial in the later discussions. Please denote more definitely the spectral assignment in Figure 2 Is the peak around 500 nm in Figure 2(a) assigned to $^{2}CT_{Cz}$ or the peak around 640 nm to $^{2}CT_{Cz}$? Both CT states ($^{2}CT_{Cz}$ and $^{2}CT_{Ac}$) can be stabilized in the polar solvent. Why is the transient absorption band attributed to $^{2}CT_{Cz}$ missing in MeCN? Authors describe that "the growing PIA at 430 nm is due to the anthracene triplet, arising from both $^{2}LE_{Ac}$ and $^{4}LE_{Ac}$ populations." Authors made this assignment based on the comparison with the time-profile of the GSB. This assignment is true because this assignment also confirmed because of the similar spectrum was observed in the simpler anthracene–radical linked systems (see Chem. Lett. 2016, 45, 1324). Please cite the corresponding literature. The expression that "This band is convolved with the GSB to varying extent" is slightly unclear. Does this mean that as signal attributed to 2,4LEAc decays, signal attributed to GSB also decays in the same way? If so, please describe this more clearly. Also, please add a figure showing the correlation between these two transition intensities to the ESI.

4) Authors carried out the spectral simulation of the trESR using EasySpin and the populations are assigned to Q+2/3, Q+1/2, Q-1/2, Q-3/2, D+1/2, and D-1/2, respectively. The expression of "Q+2/3, Q+1/2, Q-1/2, Q-3/2, D+1/2, and D-1/2" mean usually high-field spin sublevels. However, the intersystem crossing of the spin-orbit interaction normally leads to the selective population to the zero-field spin sublevels (see J. Chem. Phys. 1973, 58, 985 for Triplet-state, J. Am. Chem. Soc. 1998, 120, 3152 for quartet of ZnTPP-p-nipy, and J. Am. Chem. Soc. 2000, 122, 984 for quartet of Anthracene-radical linked system). For the selective population to the high-field spin sublevels, the spectral pattern of the quartet state is different to that of the zero-field spin sublevel's population (Angew. Chem. Int. Ed. 2006, 45, 4666 and J. Mater. Chem., 2008, 18, 381). As also shown in Fig. 7 of J. Magn. Reson. 2023, 349, 107410 (the paper of EasySpin), the dominant population to Q+1/2 and Q-1/2 gave a spectral pattern of the high-field spin-sublevels population, which is different to Figure 3(a). Figure 3(a) is a typical tr-ESR pattern for the selective population to the Q'±1/2 zero-field spin-sublevel (see J. Am. Chem. Soc. 2001, 123, 294). In addition, the spectral pattern of Figure 3(a) is opposite microwave absorption/emission to the triplet-state of anthracene itself. This is a typical pattern obtained by the radical-spin-orbit borrowing enhanced intersystem crossing (see also J. Am. Chem. Soc. 2001, 123, 294). Please check again the assignment of the population written in Table 2 and please cite the above reference because the tr-ESR pattern is quite like Figure 3(a).

5) The simulation spectrum shown in Figure 3(b) does not reproduce the observed spectrum especially the XY peak intensity. Please check the population as well as the assignment (high-field population is true?) written in Table 2.

6) Based on the conclusion of this paper, the Cz moiety is the source of luminescence. Therefore, it does not appear necessary for the radical moiety to be luminescent. It appears that as long as the energy relationship is satisfied, there should be no problem. If that is the case, it would be better to mention this point in the conclusion.

7) Although luminescent radicals without Cz moiety have been reported with very high quantum yields of luminescence, the differences between these systems and the present systems should be clearly mentioned.

8) The reason why the luminescence quantum yield of T-3Cz-Acr is slightly lower than that of T-3Cz-An has discussed based on the energy location of $^{2}CT_{AC}$. Can this phenomenon not be simply explained by the fact that the non-radiative decay efficiency of Acr itself is higher than that of An?

9) Line 101 in page 7: Authors describe that “$^{2}CT_{Ac}$ becomes the lowest excited state upon stabilization in all compounds”. However, the results of the MO calculation presented only for TOL and MeTHF. Please confirm the results of “2CTAc becomes the lowest excited state upon stabilization in all compounds” by the MO calculations.

The following is a list of minor points.
10) Line 40 in page 3: Authors describe “We identify four excited states required for their operation (Fig. 1d):” In Fig. 1, 1d is missing. Does it refer to Fig. 1c?

11) Line 42 in page 3: Does the latter refer to $^{2}CT_{Ac}$? Please identify more clearly.

12) Line 27 in page 3: About the citation in “Critically, such compounds have thus far been non-luminescent,[8,9] limiting their potential.”, please add “Chem-A Eur. J. 2020, 26, 980” because there is described a comparison between the non-luminescent and luminescent π-radicals.

13) $^{2}(D_1S_0)$ and $^{4}(D_0$T<sub>1) are better than $^{2}D_1S_0$ and $^{4}D_0$T<sub>1.

14) Line 202 in page 13: “Due to the contribution of the TTM SOMO to the delocalised π-system,…. Please cite appropriate references.

Version 1:

Reviewer comments:

Reviewer #1

(Remarks to the Author)
The authors have satisfactorily addressed the reviewer comments which has resulted in a significantly improved manuscript. The added discussion on electronic couplings and state energetics makes the work much more impactful. However, there are still a few points that should be addressed before publication:
1.) The new PL data for 0.5% doped PMMA films is a nice addition, and it’s encouraging that emission is possibly observed from CT(Ac). However, the new 0.5% data in Figure S23 (presumably recorded at room temperature) displays an emission profile centered at 750 nm for An-T-1Cz at 30 microseconds. Meanwhile, the corresponding data in Figure S24 for the 5% sample displays an emission spectrum centered at ~650 nm from 10-50 microseconds. The authors should clarify why this discrepancy exists and if it is a concentration effect.

2.) The updated state labels should be included with Fig. 2b,e so that readers are not confused by the assignments. My understanding is that in Fig. 2b, it can be labeled that both CT(Ac) and CT(Cz) are still present. In Fig. 2e, the feature can be labeled as just CT(Cz).

3.) Adding the TA kinetic fits to the SI is a huge improvement to the manuscript. However, a discussion of the fit results in the main text would be beneficial, rather than a non-specific reference to Figures S30-32 on Line 101. Direct references to data from the SI in the discussion would also be beneficial. For example, on Line 108: “T-An, An-T-1Cz and An-T-3PCz, display similar decay kinetics, with sub-50 ps decay lifetimes.” A reference to the newly added Table S6 is needed here so readers know where to look for this data.

4.) More generally, all figures in the SI should be referenced in the main text unless they are part of some discussion in the SI, which is then referenced in the main text.

5.) While the single-wavelength kinetic analysis is a nice addition, it is not clear why the SVD analysis is also being included if the authors believe it to have limited utility. We suggest removing it unless the authors believe it to be necessary.

6.) While the trESR now includes fits, the method and parameters which went into those fits still seem arbitrary.

• Why would there be a Boltzmann population present in a transient experiment? Does this suggest that T1 is on the order of 500 ns, which means that the ratio of polarized to Boltzmann spectra should change as a function of time over the course of the experiment. Further, why does it then change so drastically between samples?
• The polarized portion of simulations include population of the trip-doublet and trip-quartet manifolds
• How come the dipolar coupling is negative in An-T-1Cz and An-T-3PCz and why is there such a large rhombic component in all four?
• In table S8, how are the quartet populations and the triplet populations related? It is not apparent from the code snippet. Are you following a method similar to what is described in Appl. Magn. Reson. 2011, 40, 189 and more recently J. Am. Chem. Soc. 2025, 147, 38762?
• Both of those papers, Appl. Magn. Reson. 2011, 40, 189 and J. Am. Chem. Soc. 2025, 147, 38762, relating to quartets should be referenced.

7.) In J. Am. Chem. Soc. 2025, 147, 38762, which is another example of a TTM radical-chromophore dyad, it was shown that with the proper energetics, the CT state mediates quartet formation,. This should probably be discussed as in this work the

CT state was suggested to act as a dark trap, is that a result of an energetics mismatch?

8.) Lines 87-88, a reference is made to PMMA data in Figure S21, but the figure caption says the data was recorded in toluene.

9.) Figure S21: the plot in the top right is labeled as An-T-3Cz, but it should be An-T-3PCz. This seems to be a reoccurring error so double-check that all compound labels are correct.

10.) Line 179: there is a reference to Fig. 1d, but this panel does not exist. The reference should be to Fig. 1c.

Reviewer #2

(Remarks to the Author)
The authors have addressed all my questions well.
I recommend it can be accepted without any change.

Reviewer #3

(Remarks to the Author)
I co-reviewed this manuscript with one of the reviewers who provided the listed reports. This is part of the Nature Communications initiative to facilitate training in peer review and to provide appropriate recognition for Early Career Researchers who co-review manuscripts.

Reviewer #4

(Remarks to the Author)
The authors have appropriately revised the manuscript for many of the issues that I pointed out. However, the revised manuscript still contains several ambiguities and unresolved questions (not sufficiently addressed). The issues are listed below. Therefore, we recommend acceptance after the clarification of the following points.

(1) According to my remark, authors added more detailed photophysical data of both toluene solutions and dilute PMMA films in Tables S3 and S4. This data makes a comparison between the solution and film data is crucial for gaining an understanding of the mechanism. Based on this comparison, authors mention as follows in the main text.
“A smaller number of conformers can populate the $^{2,4}LE_{Ac}$ in films compared to solutions, as seen from the increased contribution of prompt emission in all compounds (Table S4).”
What does “conformer” mean here? Furthermore, judging from the data (PL efficiency) shown in Tables S3 and S4, the increased contribution of prompt radiation by using PMMA is observed only in An-T-3PCz and T-3Cz-Acr, not in all compounds. The description in the above sentence is ambiguous and difficult to understand, and it appears to be inconsistent with the experimental results (comparison of Tables S3 and S4).

(2) While it may be unavoidable that complexity arises because multiple distinct states can overlap to form candidate TA spectra, the assignment of TA spectra remains complex and inherently ambiguous. In the radical spin-orbit borrowing mechanism proposed for the anthracene-radical linked systems showed the quartet Tr-ESR spectra resembles Figure 3, a pathway via the CT state constructed from anthracene anion radical is proposed as one of the most possible candidates (JACS 2001, 123, 294. and JACS 2025, 147, 43358). In addition, the polarization transfer to the ground state like that described in this paper has also been observed in the latter paper (although the DEP direction in the central peak is opposite, it may come from the sign of the exchange coupling in the CT state).
Is not the possibility of anthracene anion radical, although the authors assign the band near 700 nm to anthracene radical cation? Does not exist the CT state with the anthracene anion radical nearly above the lowest quartet state based on the MO calculations?

(3) In the time-resolved ESR simulations, the DEP in the zero-field ground state can be clearly understood, but the DEP in the uncoupled basis is unclear. The DEP in the spin sublevels under external magnetic field should depend on the direction of the external field relative to the principal axes of the fine structure tensor. Since the DEP of the T0 sublevel in uncoupled basis equals that of Tz sublevel in the zero-field basis, the direction of the external magnetic field for the DEP in the uncoupled basis seems to be parallel to the z principal axis of the fine-structure tenses. Are these values for DEP in the magnetic field direction being cited as a representative example? This point is unclear. However, since the authors are not using a self-developed simulation program but rather the publicly available EasySpin program and this issue is not main subject of this paper, I will not require further detailed explanation for this point. However, it seems strange that the observed spectrum of An-T-3PCz shows aa/ee pattern (a/e : absorption/emission of microwave) but Ppol/PBoltz = 0.2 is used in the simulation. If Boltzmann population is dominant (80%), the spectrum should be fully absorption pattern. Please check the ratio again.

(4) Authors describe “This suggests that the radical-enhanced spin-orbit borrowing is active regard-less of strength of radical-acene electronic coupling”.
The efficiency of the radical-enhanced spin-orbit borrowing mechanism is not proportional directly to the radical-acene electronic coupling. The efficiency depends on the electron transfer rate to the CT state and the magnitude of SOC on the

radical site (see: JACS 2025, 147, 43358. In this paper, the spin-coupler dependence of J and DEP generation is described). Please revise the above sentence appropriately.

Version 2:

Reviewer comments:

Reviewer #1

(Remarks to the Author)
The authors have satisfactorily addressed most our previous comments aside from the EPR.
Perhaps you have misinterpreted J. Phys. Chem. A 2006, 110, 31, 9607, the Boltzmann contribution to the spectrum arises at long times and is a result of spin-lattice relaxation. Your TCW spectra are recorded at fairly early times in the kinetics and should have minimal contributions from T1 relaxation, not 20-30% of the observed spectrum. The polarization kinetics from figure S39 show that relaxation (and delayed emission) doesn’t influence the spectrum for many microseconds. So, something still doesn’t quite add up with respect to how the simulations were performed.
From the provided Matlab code snippet, the rotation matrices, States, provided from cgmatrix represent an uncoupled basis to coupled basis transformation. The transposes are incorrect when using the state matrices to transform from coupled to uncoupled basis.
With respect to the dipolar coupling, given that they are rooted in the structure, which is not mentioned in the text, why did you choose to present them in that form rather than including the tensor in its diagonal basis and the appropriate euler angles?

Couple minor points
1.) Figure S39 caption: 328 mT appears to be the maximum of the Y polarization, not the X polarization (see J. Magn. Reson. 2023, 349, 107410). Double check this assignment and correct it if needed.
2.) The title of the manuscript does not match the title written in the Supplementary Information.

Reviewer #3

(Remarks to the Author)
I co-reviewed this manuscript with one of the reviewers who provided the listed reports. This is part of the Nature Communications initiative to facilitate training in peer review and to provide appropriate recognition for Early Career Researchers who co-review manuscripts.

Reviewer #4

(Remarks to the Author)
The authors have adequately revised the manuscript for the issues that I pointed out. I have only one remark about author's opinion for the TA assignment as the anthracene cation. I think that the possibility via the CT state of the anthracene anion-radical species being involved cannot be completely ruled out as described below. For this reason, I believe it is preferable to leave a room for the assignment. However, I do not strongly object to the author's assignment of TA as a cation species. Therefore, the current revised manuscript is considered acceptable as it is, if authors want it, although there remains a room for the different assignment.

(1) According to the pairing property of the alternant hydrocarbons, the anion and cation of the anthracene make the spectra resemble each other (JACS 95, 3473 (1973)).
(2) According to the energy gap-law (Molecular Physics, 18, 145 (1970), the radiation-less transition (including CT) prefers in the energetically close levels. Therefore, the pathway via relatively higher energy CT state may be preferred.

RSO-EISC mechanism itself is expected to occur when the photoluminescence from the anthracene moiety is suppressed via the pathway to the CT state. Therefore, whether the CT state is anthracene cation or anion, this does not contradict the TrESR results. However, based on the above-mentioned points, unless the trityl cation spectrum can be definitively distinguished from that of the trityl anion and the possibility of a trityl anion can be completely ruled out, I think that the possibility via the CT state of an anthracene anion cannot be entirely excluded.

Version 3:

Reviewer comments:

Reviewer #1

(Remarks to the Author)
The authors have addressed all of our concerns.

Reviewer #3

(Remarks to the Author)
I co-reviewed this manuscript with one of the reviewers who provided the listed reports. This is part of the Nature Communications initiative to facilitate training in peer review and to provide appropriate recognition for Early Career Researchers who co-review manuscripts.

Open Access This Peer Review File is licensed under a Creative Commons Attribution 4.0 International License, which permits use, sharing, adaptation, distribution and reproduction in any medium or format, as long as you give appropriate credit to the original author(s) and the source, provide a link to the Creative Commons license, and indicate if changes were made.
In cases where reviewers are anonymous, credit should be given to 'Anonymous Referee' and the source.
The images or other third party material in this Peer Review File are included in the article’s Creative Commons license, unless indicated otherwise in a credit line to the material. If material is not included in the article’s Creative Commons license and your intended use is not permitted by statutory regulation or exceeds the permitted use, you will need to obtain permission directly from the copyright holder.
To view a copy of this license, visit https://creativecommons.org/licenses/by/4.0/

ANSWERS TO REVIEWER COMMENTS

We thank all four Reviewers for their thoughtful and constructive comments, which raised several important technical points. We have addressed each of these points, resulting in substantial improvements to the manuscript. In this document, the comments are in black, our responses are in blue, and changes to the main and supplementary texts are in green.

Reviewer #1 (Remarks to the Author):

In this work, the authors explore the role of charge-transfer states on the mechanism of quartet formation and delayed luminescence in TTM-acene dyads. The primary focus is placed on how the radical-acene electronic coupling impacts the energetics of the CT state, which is proposed to act as a trap that non-radiatively deactivates the excited state. The TTM radical is of high interest in a broad range of different fields for its unique excited state properties. This work could potentially provide some much-needed design principles for tuning the excited state behaviour of TTM radicals. However, many of the central claims made in the manuscript are only vaguely supported by the spectroscopic data, and some claims appear to have no evidence at all. Much of the data is also poorly analysed. As a result, the presentation and discussion of the data is difficult to follow at times, and the conclusions made by the end of the manuscript are uncertain. In its current state, we believe the manuscript is not acceptable for publication in Nature Communications unless substantial revisions are made. Comments and questions are listed below to justify our decision.

We thank both Reviewers for their thorough examination of our work, and for recognising the need for unveiling new design principles for luminescent radicals. We have included new results and made improvements to our analysis methods, which we believe resolve all the points raised.

Major Concerns

1.) Many arguments in the manuscript are based on relative electronic coupling strengths between the TTM and anthracene sub-units, but only a qualitative and unspecific discussion of the couplings is given. For example, the authors should define exactly what they mean when they invoke electronic couplings, as this interaction can manifest in many different ways. The design principles derived from this work would be much stronger if the electronic couplings could also be quantified. For example, what range would be designated “weak” as opposed to “strong” couplings? In this vein, comparing the electronic absorption spectra of the dyads with their corresponding monomer subunits would be extremely helpful for getting a sense of the change in electronic couplings.

We thank the referee for this suggestion. In the revised article, we have significantly expanded on our treatment of electronic couplings and interactions, as well as explicitly calculated these quantities using our semiempirical model. We stipulate that two different types of electronic interactions between radical and acene subunits play a role in dictating the optoelectronic properties of the dyads: i) the Coulomb attraction between an electron on the radical and hole on the acene (the $J_{A1,R0}$ term in eq. S12), and ii) the electronic coupling between $^{2}CT_{Cz}$ and $^{2}CT_{Ac}$ states via a first order Hamiltonian element (eq. S27b). Both the Coulomb interaction and $^{2}CT_{Cz}$ – $^{2}CT_{Ac}$ coupling are significantly larger for molecules with “strong” coupling (An-T-3PCz and An-

T-1Cz) than for those with "weak" coupling (T-3Cz-An, T-3Cz-Acr). All details are included in the expanded SI Section 5. We have changed the order of the Theoretical Insights and Quantum-Chemical Calculations sections in the main text, and summarise the quantitative results as: "We find that this radical-acene electronic coupling is between 2 and 6 times larger in An-T-1Cz and An-T-3PCz than in T-3Cz-An and T-3Cz-Acr (Table 2). Further calculation results are shown in Table S10."

Table 2. Interaction strength quantification. Coulomb stabilisation and radical-acene electronic coupling terms for the investigated dyads calculated using the semiempirical model described in Section S5.

Compound	Coulomb stabilisation / eV	Electronic coupling / meV
An-T-1Cz	1.17	36.3
An-T-3PCz	1.17	29.6
T-3Cz-An	0.75	6.0
T-3Cz-Acr	0.67	12.7

Detailed treatment of absorption spectra is presented in our answer to Point 6. The size of the interactions found from our calculations and through examining the UV-Vis spectra reveals that profound consequences in material performance are seen already under moderate differences in coupling strength.

2.) The author's assignment of delayed emission in some instances is concerning. For example, it is clear from the bottom two panels of Figure S11 that T-3Cz-An and T-3Cz-Acr exhibit prompt and delayed emission originating from the same state. However, the emission profiles in the top two panels for An-T-1Cz and An-T-3PCz red-shift with time, possibly indicating the presence of a separate emitting state. This would not be "delayed emission" in the traditional sense, where the same emission channel is thermally repopulated. This issue appears to be worse for the data in THF in Figure S13. A brief explanation for the band shifting is given in the figure caption, but it is largely insufficient and does not address why the profile broadens so much in THF versus toluene. Does the emission spectrum of bare TTM-3Cz red-shift and broaden like this in THF? If not, given that the spectral shape is so different, is it reasonable to conclude that emission from a separate state (possibly CTAc) is being observed?

The possibility of direct emission from $^2CT_{Ac}$ state is intriguing and we thank the Reviewer for prompting us to reconsider it. In this question we will address the solution case. The strong positive solvatochromism of emission from the $^2CT_{Cz}$ state in TTM radicals is well documented, e.g. Gamero et al., Tetrahedron Lett. 2006, 47, 2305–2309; Velasco et al., J. Org. Chem. 2007, 72, 20, 7523–7532; Ai et al., Nature 2018, 563, 536-540, which established that significant redshift and loss of PLQE is typical for all carbazole-substituted luminescent TTM radicals in polar media.

As requested, we performed measurements on bare T-3PCz (which is the name for TTM-3Cz we use for consistency), which is a reference compound for the structures in our paper. In THF solutions of T-3PCz, we observe a redshift over 0.1 eV of the emission peak, and subsequent time-dependent blueshift. We interpret that this originates from faster non-radiative decay in the lower-energy conformers in the molecular ensemble of solvated $^2CT_{Cz}$ excitons, as explored by Englman & Jorner, Mol. Phys. 1970, 18, 145-164 and Grabowski et al, Chem. Rev. 2003, 103,

3899–4031. We note that given the NIR emission region, the wavelength axis may increase the apparent broadening, compared to data in the visible region, or if plotted on an energy axis. Additionally, we do not observe emission in the T-An in any studied solutions where $^2CT_{Ac}$ state is the lowest energy excited state. This is consistent with its order of magnitude lower oscillator strength found by calculations as compared to $^2CT_{Cz}$. Taken together, it demonstrates that the ns-μs emission in polar solvents originates from the same stabilised $^2CT_{Cz}$ state in compounds with or without anthracene.

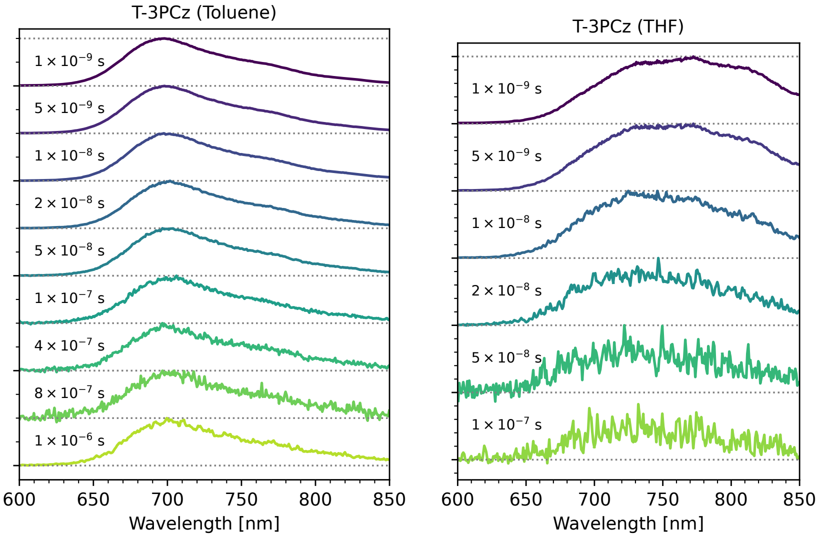

Fig S19. Time-resolved PL of the 0.1 mM T-3PCz solution in toluene (left) and THF (right) following 490 nm excitation. Significant broadening and red shift of the emission profile in polar solvents is typical for TTM-type D_1 emission. We also observe small conformation-dependent blue shifts at late times.

3.) Similar to the last point, the temperature-dependent "delayed emission" assignments in PMMA (Figures S14-17) are even more concerning. The authors acknowledge the gradual blue shift of the spectra at later times in the main text, but do not address the drastic change in the spectral shape at later times compared to the prompt spectra (see Figure S14 in particular). It is more justifiable to assign these two emission components to entirely different species rather than the proposed delayed emission. It is possible that there is significant aggregation occurring in these films, given the large radical loadings (5 w/w%), which may result in this broadened emission. The authors should provide a rigorous explanation for this inconsistency and why they have chosen to assign this as a delayed emission. The energy barriers for delayed emission are also calculated from this data, so the corresponding Arrhenius plots for all compounds should be shown in the SI.

To verify the absence of aggregation effects in the 5% films, we performed additional transient PL measurements on 0.5% PMMA films of An-T-3PCz and T-3Cz-An, as two representative compounds. The spectral evolution and kinetics are very similar to those found in the 5% films. Even at these low loadings, we observe a time-dependent blue shift and broadening of the emission in PMMA films, indicating that these effects are unlikely to stem from aggregation. We note that intermolecular radical-radical interactions would likely redshift, not blueshift, the emission at late times (Kimura et al. *Angew. Chem. Int. Ed.* **2018**, *57*, 12711).

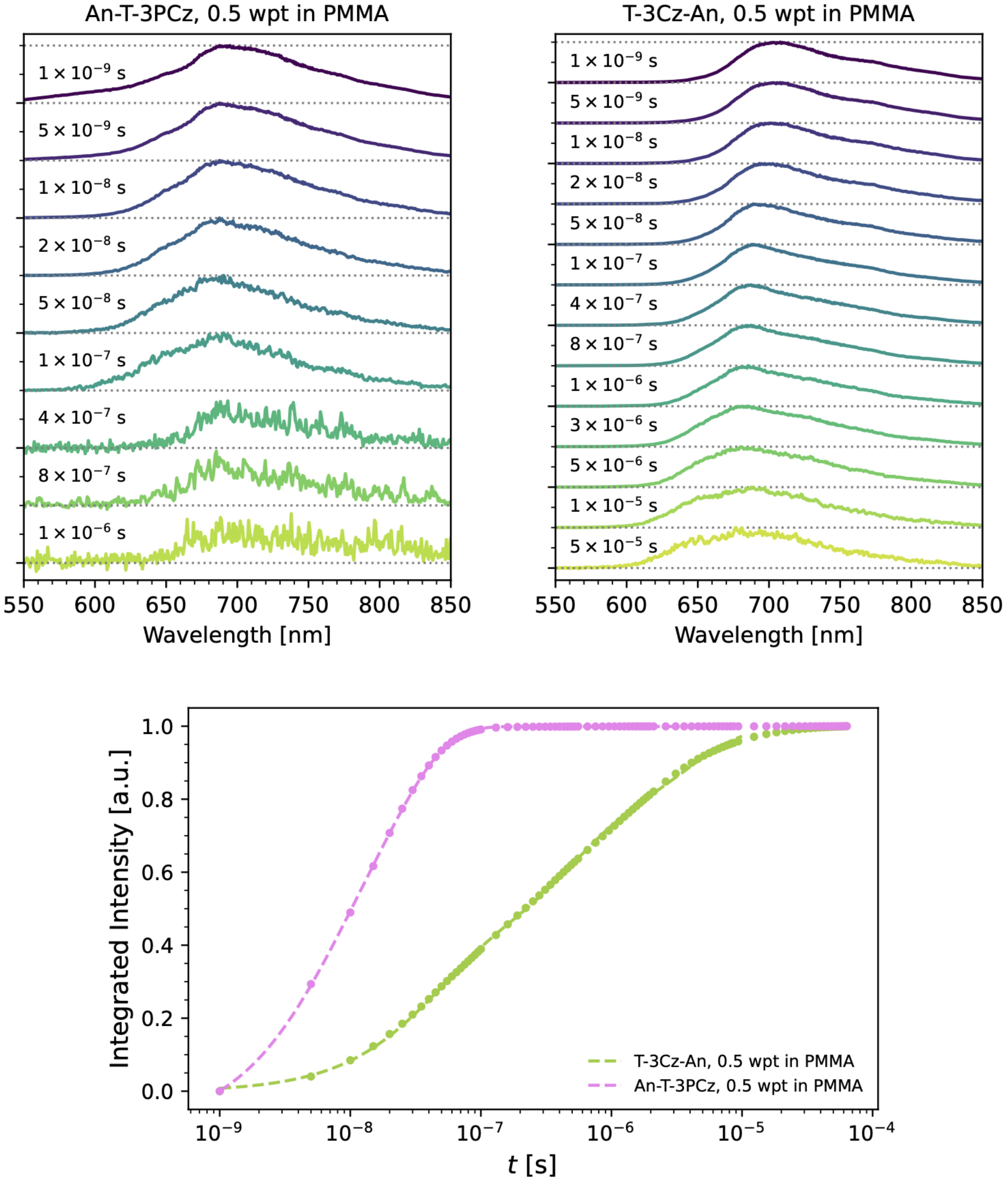

Fig S22. Time-resolved PL on 0.5% in PMMA films of An-T-3PCz and T-3Cz-An at room temperature following 490 nm excitation.

Now we turn to the possibility of direct emission from $^{2}CT_{Ac}$ state in films. We performed additional transient PL measurements on 0.5% PMMA films using long delays and exposure times, high gain and fluence (37 μJ/cm^2). We observe a very weak broad emission peak between 720 nm and 750 nm at late times in An-T-1Cz and An-T-3PCz. This slow emission profile is in turn blueshifted in T-3Cz-An and even further in T-3Cz-Acr. Furthermore, we detected very weak emission centred near 740 nm of a similar profile in thick T-An films. The delayed emission profile in T-An does not differ from its steady-state emission. We estimate that the PLQE of T-An films is below 0.1%, and we do not detect its emission in solution under the same experimental conditions.

We interpret these results as reporting on the energetics of the $^{2}CT_{Ac}$ state in near-orthogonal radical-acene conformations. However, we stress that this emission arises from a very small fraction of the initial photoexcitations. Since this residual channel contributes less than 0.1% of all emitted photons in all carbazole-containing materials, the main delayed emission channel follows the same $^{4}LE_{Ac} \rightarrow {}^{2}CT_{Cz}$ regeneration as in solutions, which allows us to robustly perform Arrhenius analysis (included as Fig. S29).

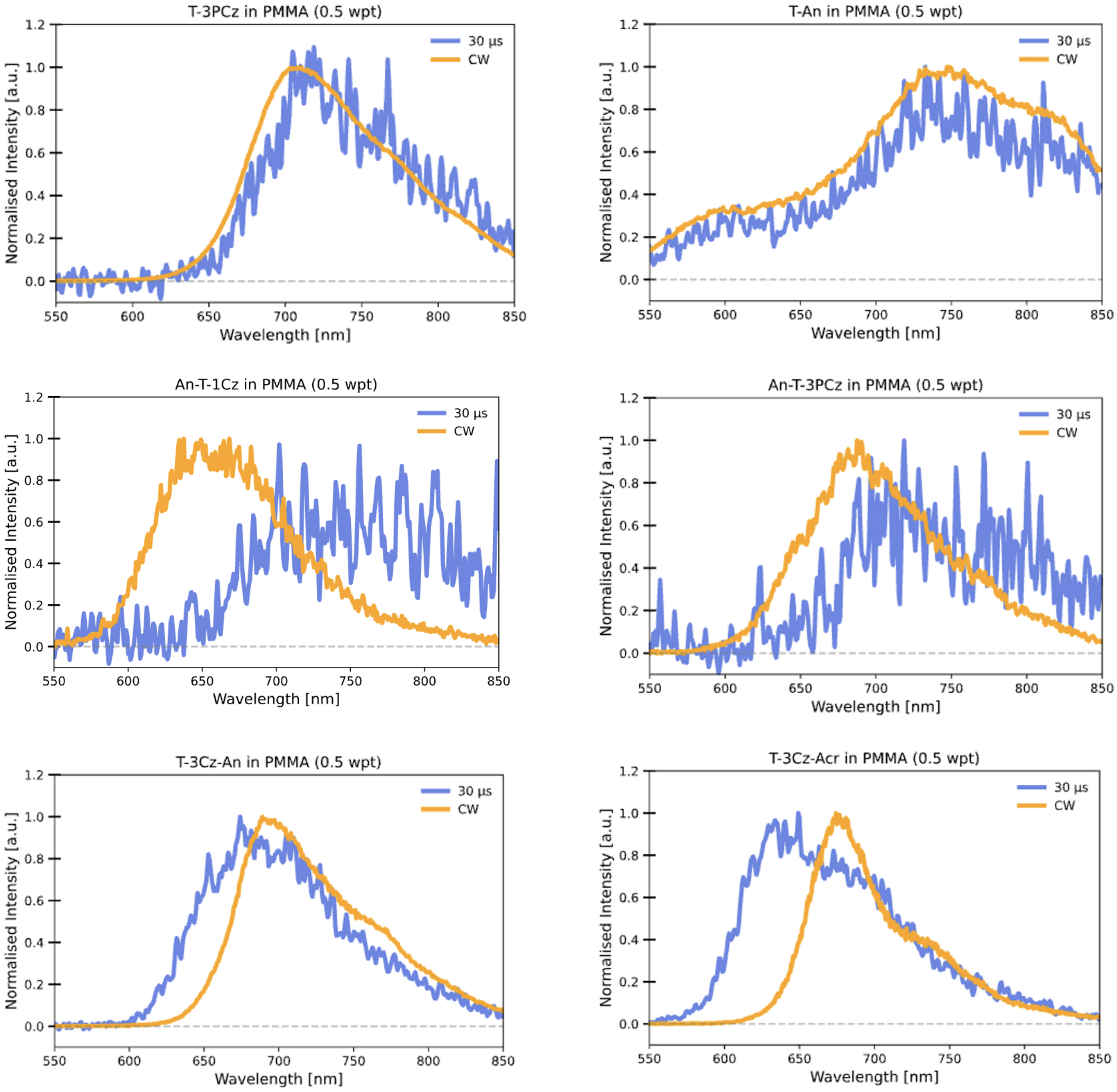

Fig S23. Steady-state and trailing emission profiles in 0.5% in PMMA films following 490 nm excitation.

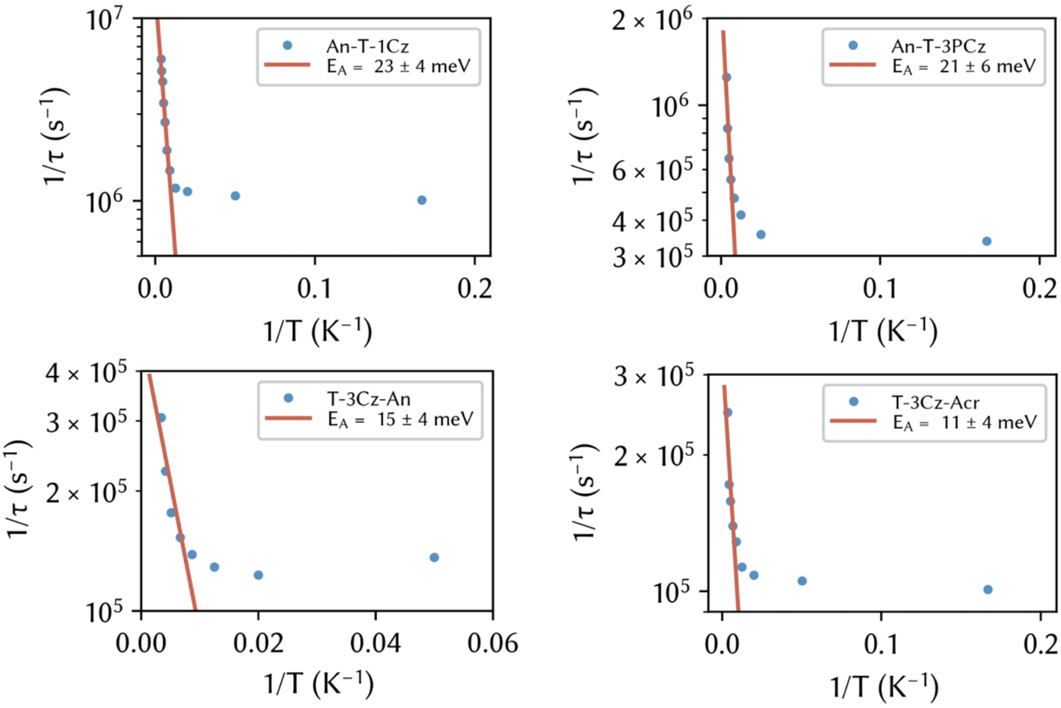

Fig S29. Arrhenius analysis of temperature-dependent transient photoluminescence data in Figs. S24-28.

4.) The band assignments in the TA data are hard to follow, mainly because CTCz and CTAc both have broad absorbance bands from ~600-700 nm. For example, Figures 2b,e assign the band at ~625 nm to CTAc, even though it is very shifted from the assignment made for CTAc in Figure 2a and more in line with the band assigned to CTCz in Figures 2a,d. The exact data that is assigned to CTAc in Figures 2b,e is also assigned to CTCz in the figure caption of Figure S19. The authors should check the TA data and provide a better explanation of the final assignments. For the CTAc state, there should be a TTM-Cz anion absorption band in addition to the anthracene cation absorption at ~650-700 nm. Is this anion spectrum known? If not, the authors should conduct spectro-electrochemistry experiments if available, to help clarify the data. Finally, a significant amount of TA data is presented, yet there is very limited kinetic analysis. Can the authors specify why they have chosen not to analyse the kinetics? A basic kinetic analysis would likely help clarify the band assignments and mechanism. In particular, it is strange that the TA features all decay in 100 ps for An-T-1Cz and An-T-3PCz, yet photoluminescence with a 3-8% yield and ~16 ns prompt lifetime is observed for these compounds.

This answer also addresses Referee 4 Comment 3.

We agree with the Reviewers on the importance of careful assignment of TA bands, so we performed additional experiments and expanded our data analysis. We inspect the visible TA spectra of the reference compounds, T-An and T-3PCz, which respectively mimic the $^2CT_{Ac}$ and $^2CT_{Cz}$ lineshapes. In toluene, both features are centred near 630 nm. In MeCN, both materials show similar lineshapes in the spectral features in this region, with an additional PIA below 500 nm. In both solvents we observe subtle differences in band evolution: $^2CT_{Ac}$ shows a small blueshift, while $^2CT_{Cz}$ shows a redshift. Additionally, $^2CT_{Ac}$ shows a broader shoulder near 700 nm. We use these differences to study early time dynamics in MeCN in the materials of interest.

For An-T-3PCz, the initial lineshape resembles the PIA of T-3PCz. We thus assign the initial population of An-T-3PCz to the $^2CT_{Cz}$ state. However, at 8 ps, the band starts resembling that of T-An. This is not the case for T-3Cz-An, which does not show significant spectral evolution, and the visible band remains similar to that of T-3PCz. We therefore remove the state labels in Fig. 2b,e; instead pointing to Figure S39 for discussion of state assignment in MeCN. The key result of these solvatochromic TA experiments is that while in toluene, T-3Cz-An and An-T-3PCz show differing behaviour, in MeCN their dynamics become very similar, indicating a change in excited state energetic ordering.

We preformed singular value decomposition (SVD) analysis (Fig. S32). In T-3Cz-An and T-3Cz-Acr, we find 2 significant components, corresponding to the correlated transformation of the $^2CT_{Cz}$ state into the $^{2,4}LE_{Ac}$ manifold. The extracted kinetics are similar to those found from sampling specific wavelengths. However, the energy transfer occurs simultaneously with the decay of the ground state bleach for other materials, and SVD recognises both as a single component, limiting its utility. Furthermore, all materials show non-negligible time-dependent spectral shifts, which break the assumptions of such matrix decomposition attempts.

We instead include an analysis of the TA kinetic traces for all studied systems in the SI. We model kinetic traces as multiexponential decays convolved with wavelength-dependent Gaussian-like IRF: $I(t) = \exp[-4\ln(2)(t-t_0)^2/\mathrm{FWHM}^2] \otimes H(t-t_0) \cdot \Sigma_{i=1}^{n} A_i \exp[-(t-t_0)/\tau_i]$. T-An bands follow biexponential decay with the prompt decay between 3.5 and 5.5 ps and the delayed decay of around 34 ps. An-T-1Cz and An-T-3PCz show similar kinetics across all bands. The exceptions are PIAs corresponding to the $^2CT_{Cz}$ state, where we find a component with a subpicosecond lifetime, which is not the IRF. We correlate this component to the internal conversion rate between $^2CT_{Cz}$ and $^2CT_{Ac}$ states. Kinetics of T-3Cz-An and T-3Cz-Acr materials resemble the kinetics of T-An in the early time with a fast component (3.6 ps for T-3Cz-An and 4.6 ps for T-3Cz-Acr) and a long-lived component corresponding to the delayed luminescence lifetime. Upon increasing the solvent polarity, the lifetimes of An-T-1Cz and An-T-3PCz do not show large changes, while for T-3Cz-An and T-3Cz-Acr they shorten significantly. We note that these fits only capture the dominant transfer rates, and some residual populations near our signal-to-noise level remain in An-T-1Cz and An-T-3PCz. These contribute to emission at few ns timescales, as seen from comparing film and solution TA data (Fig. S33).

To summarise, we introduce several changes in the experimental section and the subsequent discussion. Crucially, we correct our previous assumption about the state ordering in T-3Cz-An in MeCN. We rephrased the paragraph in question to:

"The $^2CT_{Ac}$ state becomes the lowest excited state in An-T-3PCz in acetonitrile (MeCN), with spectral profile matching that of T-An (Fig. S39). For T-3Cz-An, the $^{2,4}LE_{Ac}$ PIA is heavily in dichloromethane (Fig. S34) and in MeCN it is no longer observed, showing that the energetic ordering of the states is inverted in a high-polarity medium. The broad PIA band at 625 nm resembles that of T-3PCz in MeCN with lifetime matching An-T-3PCz. This reveals that a high polarity environment exerts a dual effect. It quenches $^2CT_{Cz}$ emission, as seen in other TTM-donor radicals. Additionally, in high-spin radicals it stabilises the CT states below the $^{2,4}LE_{Ac}$ manifold, which suppresses quartet generation."

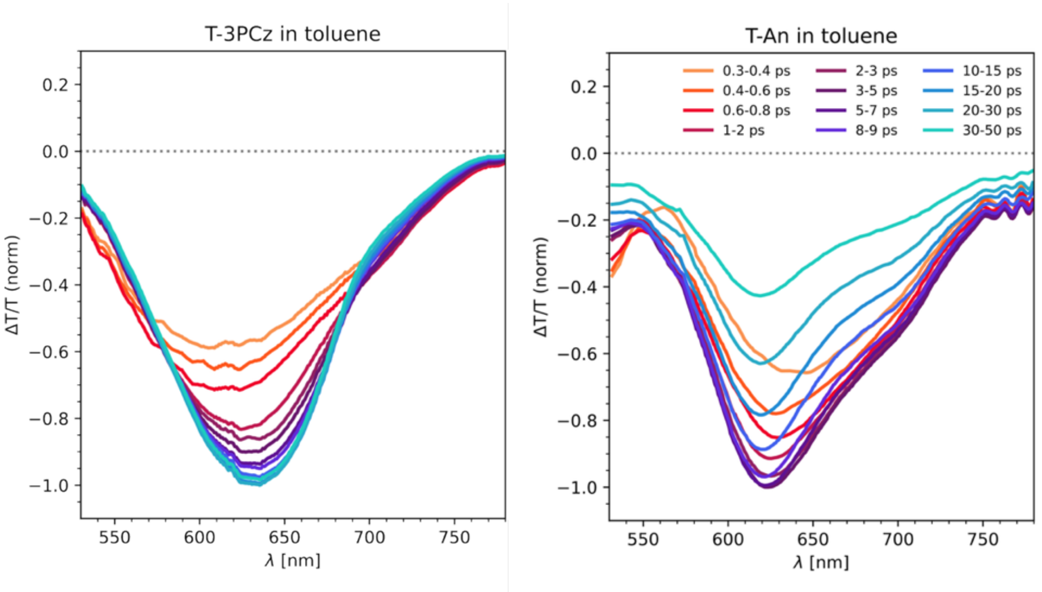

Fig S38. Early time spectral profile of 0.1 mM T-3PCz (left), and 0.2 mM T-An (right) in toluene, following 532 nm excitation. Time slices are identical across the panels.

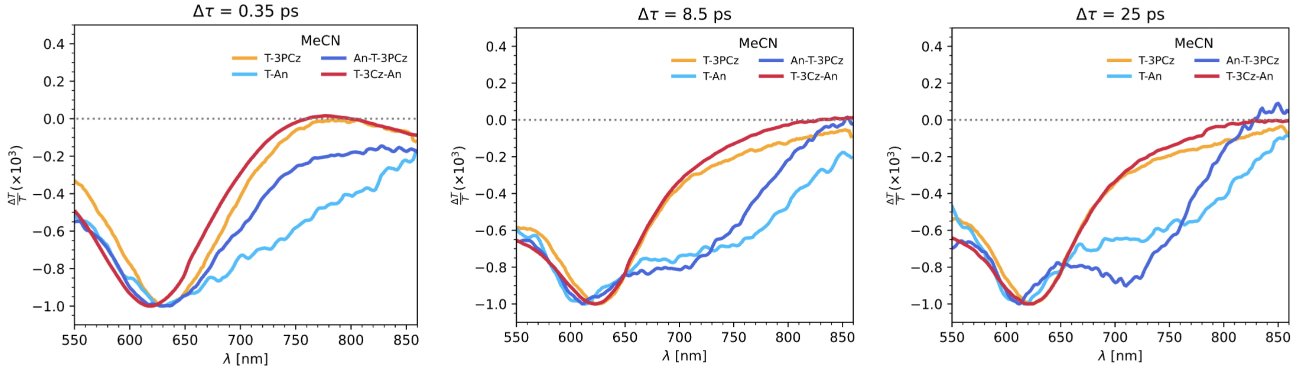

Fig S39. Normalised early time TA spectra of T-3PCz, T-An, An-T-3PCz and T-3Cz-An in MeCN. Presented slices are at 0.35 ps (left), 8.5 ps (middle), and 25 ps (right) following 490 nm photoexcitation.

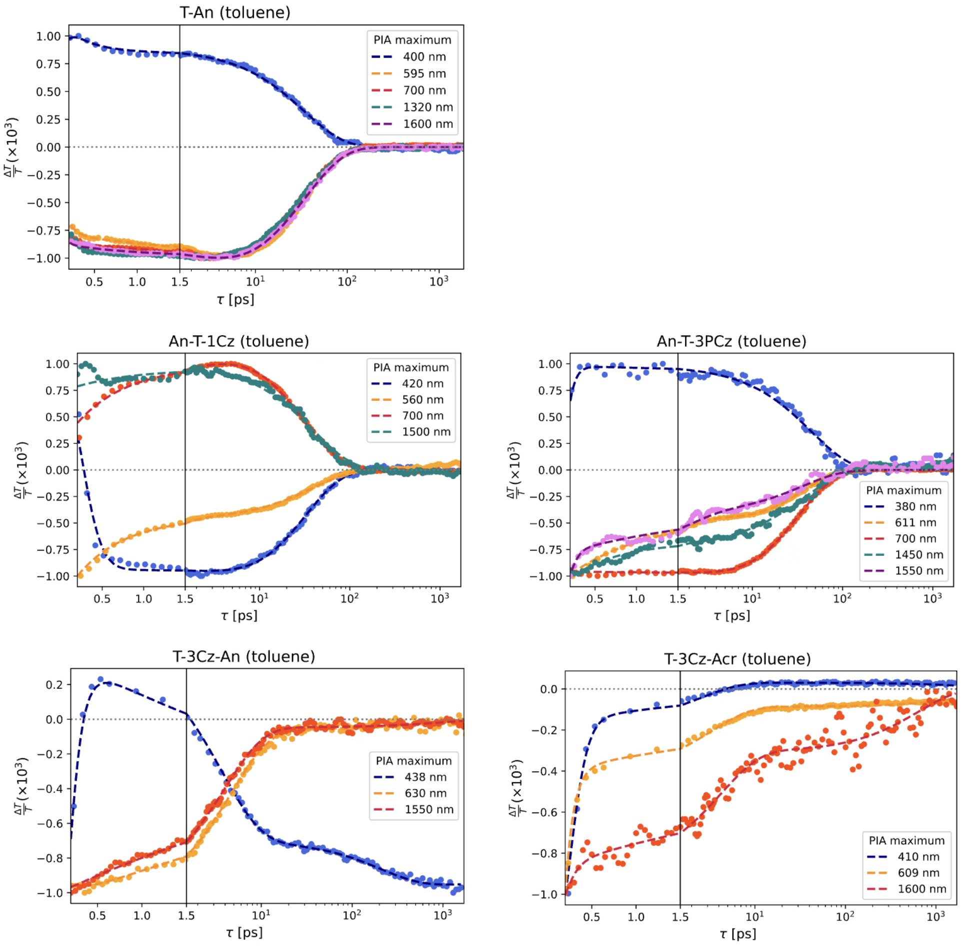

Fig S31. Kinetic traces extracted from picosecond transient absorption spectra of investigated materials in toluene, reported in Figure S30. Fitting parameters are reported in Table S6.

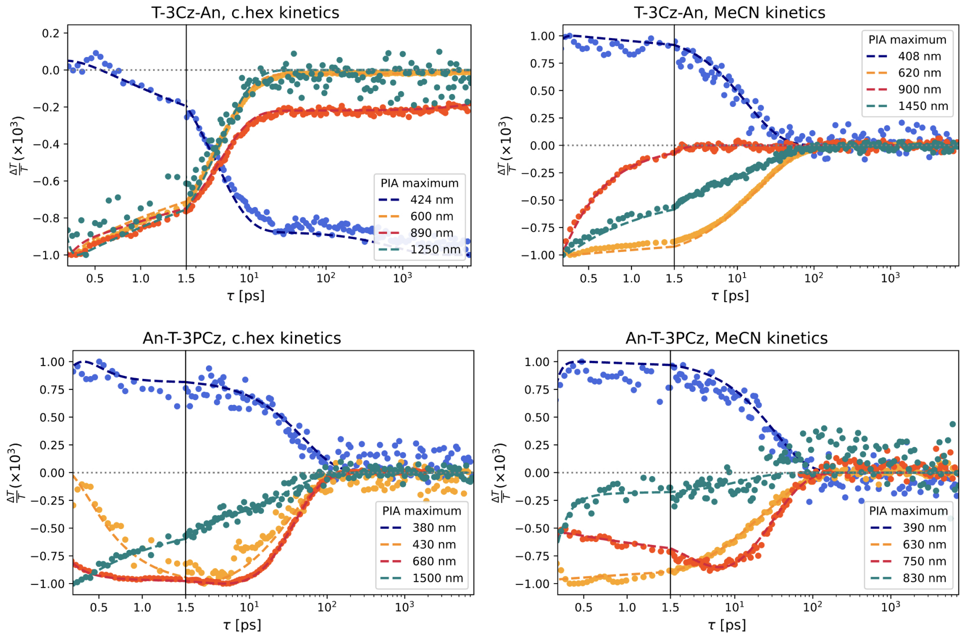

Fig S37. Kinetic traces extracted from picosecond transient absorption spectra of T-3Cz-An and An-T-3PCz in cyclohexane and acetonitrile (MeCN), reported in Figure S36. Fitting parameters are reported in Table S7.

Table S6. Fitting parameters of 0.1 mM toluene TA datasets.

System	λ (nm)	Comp.	τ [ps]
T-An	400	1	34.8 ± 0.1
	595	1	3.5 ± 0.1
		2	32.7 ± 0.1
	700	1	2.7 ± 0.3
		2	32.6 ± 3.4
	1320	1	3.6 ± 3.0
		2	32.8 ± 3.9
	1600	1	3.8 ± 0.9
		2	34.4 ± 5.5
An-T-1Cz	420	1	5.5 ± 2.9
		2	30.0 ± 3.0
	560	1	0.56 ± 0.2
		2	38.1 ± 4.1
	700	1	0.9 ± 0.6
		2	4.0 ± 1.3
		3	30.3 ± 1.4
	1500	1	0.9 ± 0.2
		2	39.5 ± 2.5
An-T-3PCz	380	1	45.1 ± 2.4
	610	1	0.95 ± 0.2
		2	8.9 ± 1.7
		3	28.6 ± 4.3
	700	1	5.7 ± 1.1
		2	30.0 ± 4.5
	1450	1	0.8 ± 0.4
		2	36.8 ± 2.2
	1550	1	1.6 ± 0.4
		2	35.8 ± 2.1
T-3Cz-An	438	1	3.7 ± 0.8
		2	>5e3
		3	>5e3
	630	1	4.6 ± 0.4
		2	>5e3
	1550	1	3.9 ± 0.6
		2	>5e3
T-3Cz-Acr	410	1	2.5 ± 0.1
		2	>5e3
	650	1	3.3 ± 0.9
		2	>5e3
	1600	1	4.2 ± 3.6
		2	>5e3

Table S7. Fitting parameters of cyclohexane and MeCN TA datasets.

System	λ (nm)	Comp.	τ [ps]
An-T-3PCz, Cyclohexane	390	1	49.6 ± 6.8
	430	1	34.0 ± 3.4
	680	1	7.5 ± 3.2
		2	31.2 ± 4.0
	1500	1	31.7 ± 4.0
An-T-3PCz, MeCN	390	1	32.1 ± 3.4
	420	1	7.4 ± 1.8
	630	1	7.4 ± 3.0
		2	36.4 ± 5.6
	750	1	3.5 ± 0.7
		2	30.3 ± 2.6
	830	1	12.7 ± 11.0
T-3Cz-An, C.hexane	424	1	3.7 ± 1.1
		2	>5e3
	600	1	4.2 ± 0.3
		2	>5e3
	890	1	4.0 ± 0.6
		2	>5e3
	1250	1	4.1 ± 2.4
T-3Cz-An, MeCN	408	1	7.8 ± 0.4
		2	14.8 ± 2.8
	620	1	6.5 ± 5.0
		2	28.5 ± 7.5
	900	1	0.5 ± 0.1
	1450	1	1.0 ± 0.2
		2	22.9 ± 6.1

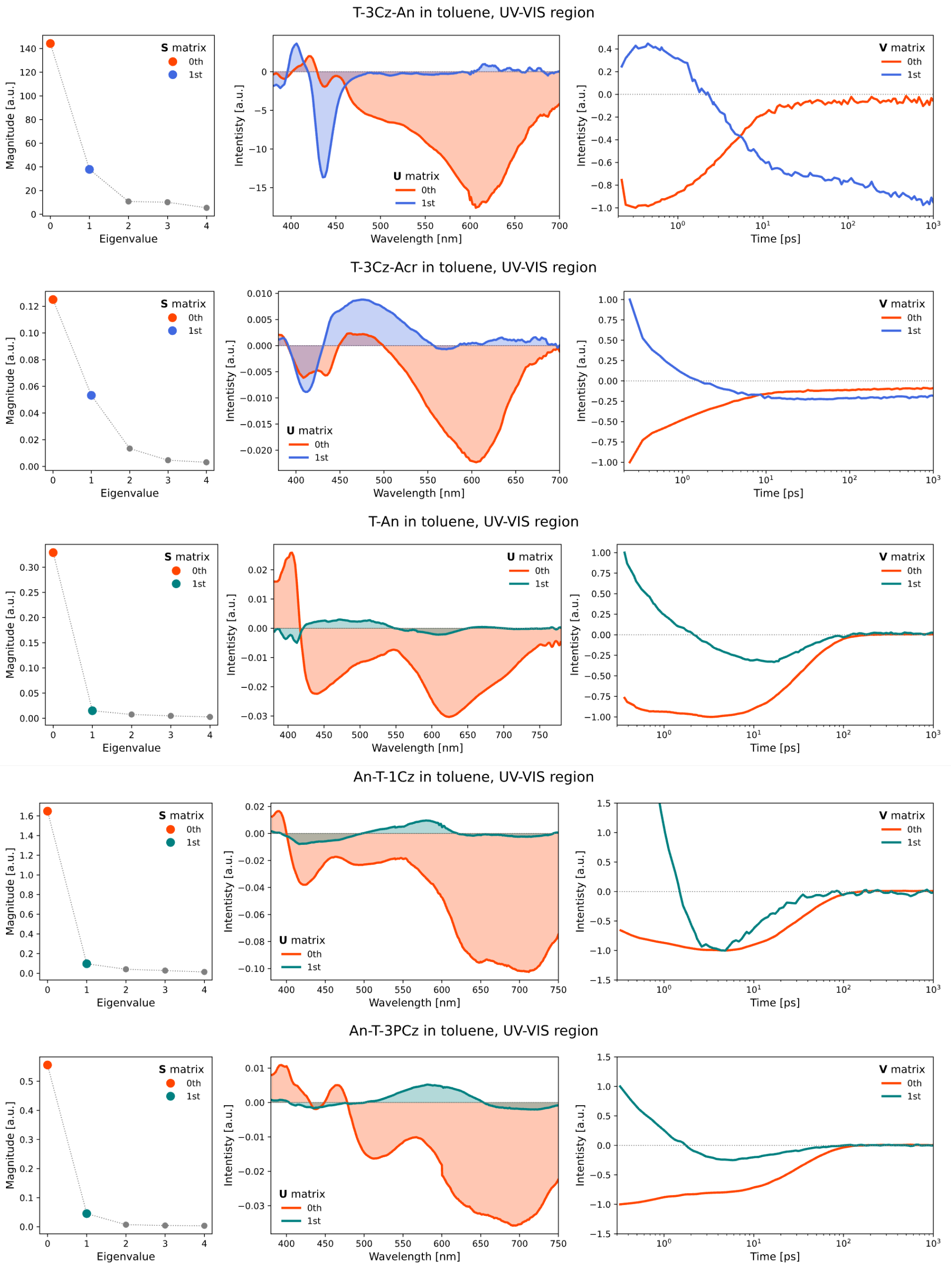

Figure S32. SVD analysis of the picosecond TA spectra presented in Figure S30.

5.) The simulated trESR spectra of An-T-1Cz and T-3Cz-Acr should also be shown in the SI, and the included fits in Figure 3 don't seem particularly good. The experimental absorptive features seem considerably more intense than the emissive. On a related note, the simulation procedure is not described with enough detail other than a brief reference to the EasySpin simulation paper by Tait et. al. Enough information should be included in the methods section or the SI to reproduce the simulations. For example: what spin Hamiltonian was used; what doublet-triplet exchange couplings were used; how were the different orientations of the triplet zero-field splitting tensor and triplet-radical dipolar interaction tensors accounted for when calculating DQ, EQ, and DTR; the sublevel populations seem arbitrary at best, how were they determined, are they related to the mechanism?

This answer also addresses Reviewer 4 Comments 4 and 5.

We agree with the Reviewers that our original trESR simulations could have been improved. We now report new simulations, obtained using the following method, adapted from Richert, Evans et al. (preprint DOI:10.26434/chemrxiv-2024-2gf9b). The spin system is composed of a triplet and radical coupled through a pair of dipolar parameters (dip) and an isotropic exchange interaction (J). The latter is fixed to a large ferromagnetic value ensuring strong coupling regime, as validated by pulsed nutation measurements. The triplet zero-field splitting (D) and isotropic g-constant values are fixed to those of anthracene or acridine, as appropriate.

To ensure correct treatment of degeneracies at zero-field, we construct the full density matrix using the code below. To account for the observed asymmetry in the full spectrum, we add a contribution from the Boltzmann populated quartet with the same spin Hamiltonian, which yields a fully absorptive signal (Sys_Bol). The polarised spectrum (Sys_Pol) is calculated in the coupled basis, and we provide equivalent populations converted into various representations in Table S8. The spectra are added, with a ratio parameter (P_Pol_to_P_Bol) controlling their relative weights. We add an additional absorptive doublet contribution that arises near g = 1.989. Without this contribution, the optimised fit of the broad polarisation cannot reproduce the experimental lineshape. To explore the full parameter space we iterate over the Hamiltonian (Fig. S40) using the *esfit* function using first a particle swarm and then Nelder-Mead simplex algorithms. For compounds with poor quartet yield (An-T-1Cz and An-T-3PCz), the intensity of the Boltzmann and polarised signals are comparable. For compounds with good quartet yield (T-3Cz-An, and T-3Cz-Acr) only a weak Boltzmann signal is detected. We note that for the An-T-1Cz dataset, the lineshape may be convolved with temperature-induced baseline oscillations, which were difficult to minimise due to the small signal size, consistent with the low quartet state yield in this compound.

The updated simulations reproduce the experimental lineshapes more closely:

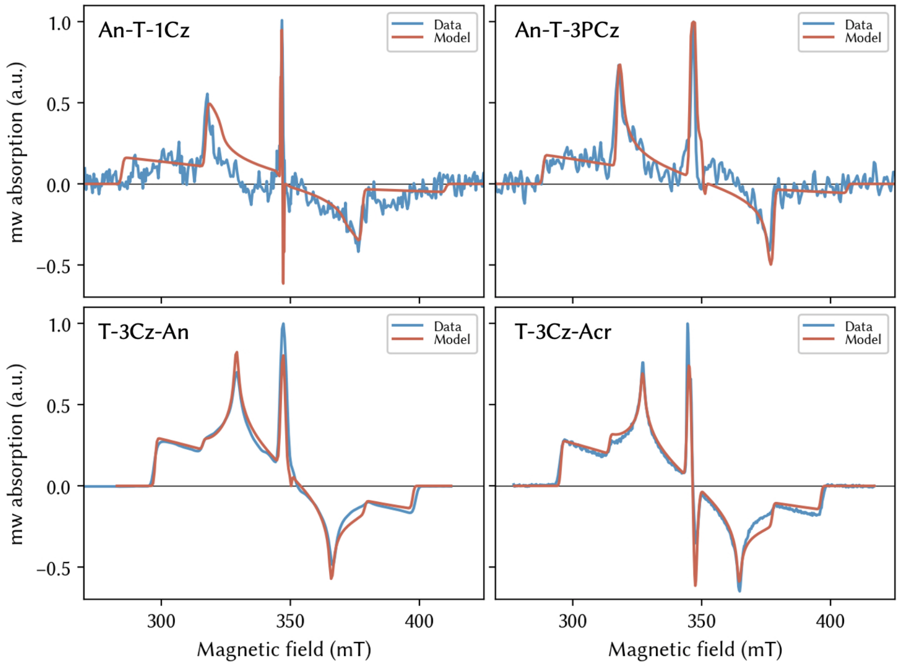

Fig S42. Simulations to the prompt (0.5-1.5μs) X-band transient ESR spectra on 100 μM frozen toluene solutions at 80 K, acquired after 600 nm 1 mJ pulses.

```
Sys.S    = [1 1/2];
Sys.g    = [g1_iso; g2_iso];
Sys.D    = [D_Ac E_Ac; 0 0];
Sys.dip  = [dip1 dip2];
Sys.J    = -2.5e6;
Sys.lwpp = 1;

[States, ~, ~] = cgmatrix(1, 1/2);

Pop_pol = [p_Qp32, p_Qp12, p_Qm12, p_Qm32, p_Dp12, p_Dm12];
Pop_pol = Pop_pol / sum(Pop_pol);

Sys_Bol = Sys;
[~, specBol] = pepper(Sys_Bol, Exp);

Sys_Pol = Sys;
Sys_Pol.initState = States * diag(Pop_pol) * States';
[~, specPol] = pepper(Sys_Pol, Exp);

specFull = P_Pol_to_P_Bol * specPol + specBol;
specFull = specFull / max(specFull);
```

Fig S40. Transient ESR Easyspin code snippet.

Table S8. Spin Hamiltonian parameters and populations found from trESR simulations.

Parameter		An-T-1Cz	An-T-3PCz	T-3Cz-An	T-3Cz-Acr
D_T, E_T (MHz)		2149, -250	2149, -250	2149, -250	2208, -261
dip_{RT} (MHz)		-183, 170	-112, 223	17, 68	29, 80
g_R, g_T		2.003, 2.005	2.003, 2.005	2.003, 2.005	2.003, 2.005
P_{pol} : P_{Boltz}		1.0	0.2	12.9	9.4
Coupled basis	$Q_{+3/2}$	0.013	0.058	0.122	0.111
	$Q_{+1/2}$	0.318	0.331	0.237	0.280
	$Q_{-1/2}$	0.267	0.298	0.237	0.274
	$Q_{-3/2}$	0.234	0.167	0.117	0.102
	$D_{+1/2}$	0.033	0.059	0.153	0.131
	$D_{-1/2}$	0.134	0.087	0.134	0.101
Uncoupled basis	T_{+1}	0.142	0.208	0.302	0.292
	T_0	0.446	0.468	0.412	0.447
	T_{-1}	0.412	0.324	0.286	0.261
Zero-field basis	T_x	0.277	0.266	0.294	0.277
	T_y	0.277	0.266	0.294	0.277
	T_z	0.446	0.468	0.412	0.447

6.) Many of the arguments made in the manuscript invoke energetic orderings of the various electronic states and their proximity to one another. However, the only detailed investigation of electronic state energies is based on TD-DFT calculations instead of experimental data. I am always hesitant to trust such arguments, given the difficulties with DFT predicting relative energies of localised versus CT states. For example, the calculations in Table 3 overestimate the 2,4LE energy by ~0.2 eV based on the triplet energy of anthracene, while they overestimate the CTCz energy by ~0.4-0.5 eV based on the optical data in Figure S10. Were attempts made to experimentally determine the various CTAc state energies, such as with donor-acceptor redox potentials and the Weller equation? The energy of the CTCz state should be estimated from the steady-state optical data in Figure S10 and explicitly stated in the manuscript/SI.

We share the concern about the challenges associated with energetics calculations. To comprehensively address the question of state energies, we conducted a series of new experiments. First, we performed molar extinction coefficient experiments in 5 aprotic solvents of varying polarity (Fig. S12, together with a Voigt deconvolution of the recorded data, Fig. S13-14). For the first absorption, relative to TTM-3PCz, we find a small (10 meV) blueshift in T-3Cz-An, which is more pronounced (80 meV) in An-T-3PCz, tracking the strength of electronic coupling. The band peaking around 2.0 eV all carbazole containing compounds shows a similar degree of positive solvatochromism as the reference TTM-3PCz compound (Fig. S15). This shows that this absorption band is largely composed of the $^2CT_{Cz}$ transition regardless of the strength of electronic coupling. The absorption at 2.25 eV in carbazole-free T-An does not show a corresponding solvatochromic response. This suggests it stems from a transition without CT character. Its spectral position matches the $^2LE_{TTM}$ seen in unsubstituted TTM radicals (Murto et al., Nat. Commun. 2023, 14, 4147). We note that this cannot originate from the $^2LE_{An}$ that involves the triplet (1.82 eV) due to the large energetic separation. For materials containing both anthracene and carbazole, the weak $^2CT_{Ac}$ band is buried in the broad absorption onset, consistent with the low calculated oscillator strength.

The examination of the spectra in toluene allows us to determine vertical excitation energies:

System	E (eV)	Assignment
T-3PCz	2.055	$^2CT_{Cz}$ pure
T-3Cz-An	2.063	$^2CT_{Cz}$ with weak electronic coupling
An-T-3PCz	2.141	$^2CT_{Cz}$ with strong electronic coupling
T-An	2.247	$^2LE_{TTM}$ pure

We introduce Photothermal Deflection Spectroscopy (PDS) to reach sufficient sensitivity to investigate the energetics of the band edge states (Fig. S16). T-3Cz-An shows a very similar absorption onset as the reference T-3PCz compound, with the band centred at 1.9 eV originating from the $^2CT_{Ac}$ absorption. T-An shows a spectrum with two resolved transitions: a weak absorption near 1.7 eV, and stronger absorption near 2.2 eV. Consistent with the UV-Vis data, we assign these to $^2CT_{Ac}$ and $^2LE_{TTM}$ absorptions, respectively. We write: "Photothermal deflection spectroscopy (PDS) allows us to investigate the energetic ordering near the band edge (Fig. S16). We resolve a weak band near 1.7 eV corresponding to the $^2CT_{Ac}$ state in T-An, indicating that it is present below $^2CT_{Cz}$ state in compounds with direct acene-radical linkage."

We analyse the PL data in PMMA films presented in Figure S23 to estimate the 0-0 state energies (Table S2). To obtain these from the adiabatic energies, we estimate the energy lost following emission during ground state vibrational relaxation using the T-3PCz reference compound (setting it to 0.15 eV). Due to the broadness of the spectra, we provide these values to 2 significant figures. In response to Reviewer 2 Comment 2 we provide ps-scale photoluminescence data which provides additional evidence of the energetic gaps and orderings.

To support these assignments, we have also carried out cyclic voltammetry on the carbazole and acene units and the radical structures to assess their experimental oxidation and reduction potentials (Fig. S10). In its caption, we write "Comparison of oxidation potentials of carbazole (1.05 V) and anthracene (1.04 V) confirms that both can function as electron donors and enable charge transfer excitation when coupled onto the radical. Acridine unit has a higher oxidation

potential (1.13 V) than the other two donors making it a weaker donor. In the radical coupled structures, oxidation of carbazole and acene overlap which means that the two processes cannot be distinguished electrochemically. Reduction of the radical site takes place at ca. –1 V, which again suggests that both carbazole and acene units can function as electron donors and the radical is as an electron acceptor in these structures. The radical site undergoes reversible oxidation and reduction processes in all structures, whereas oxidation of carbazole and acene is irreversible. The same features can be seen in the radical coupled structures; the peak current of carbazole and acene oxidation decreases after each consecutive cycle but the radical redox process remains reversible.”

Table S2. Experimentally estimated 0–0 state energies in low polarity environments.

System	$E(^2CT_{Cz})$ [eV]	$E(^2CT_{Ac})$ [eV]
T-3PCz	1.9	
T-An		1.8
An-T-1Cz	2.0	1.8
An-T-3PCz	1.9	1.8
T-3Cz-An	1.9	2.0
T-3Cz-Acr	2.0	2.1

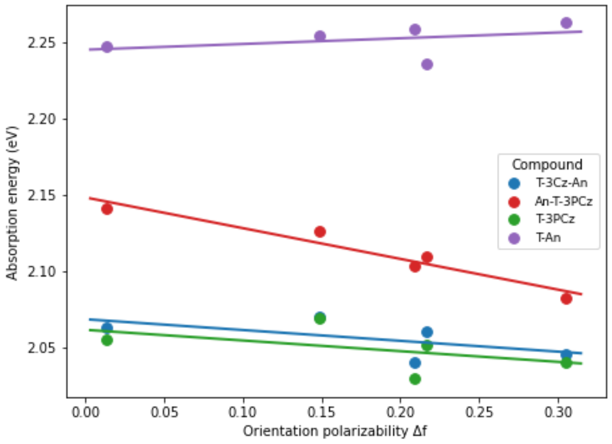

Fig S15. Lippert plot of lowest absorption band peak as a function of solvent polarizability.

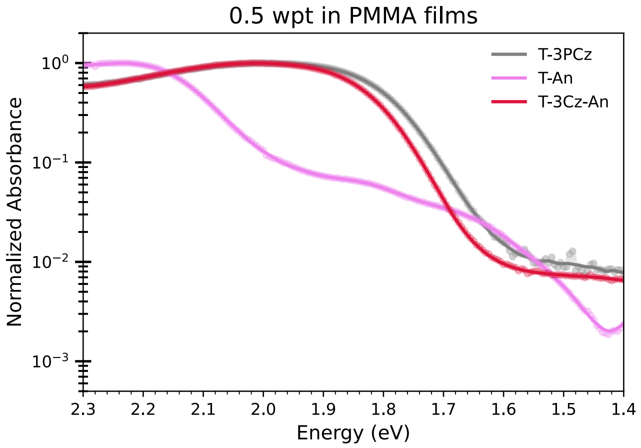

Fig S16. Photothermal deflection spectroscopy (PDS) of 0.5% films.

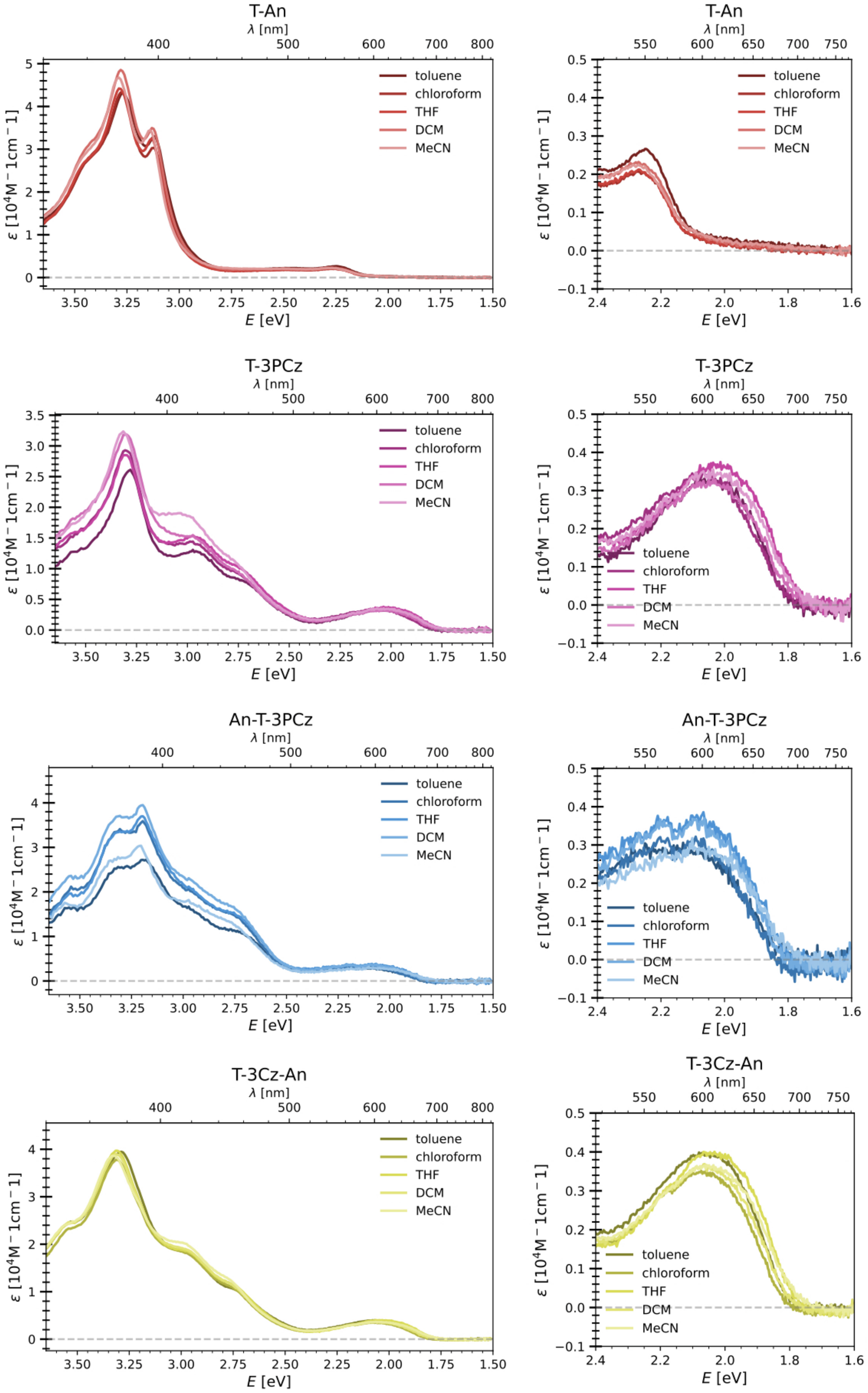

Fig S12. Steady-state absorption spectra for the solvent series of T-An, T-3PCz, An-T-3PCz and T-3Cz-An, measured using an integrating sphere. In T-An, the band at 2.25 eV we assign to the $^{2}LE_{TTM}$ transition. The $^{2}CT_{Ac}$ transition has vanishing oscillator strength and is low in energy, as predicted by the TD-DFT calculations and validated by PDS (*vide infra*). In T-An, we assign it to a long weak tail spanning the energy range between 2.1 eV and 1.7 eV. In other molecules, the $^{2}CT_{Cz}$ transition is bright.

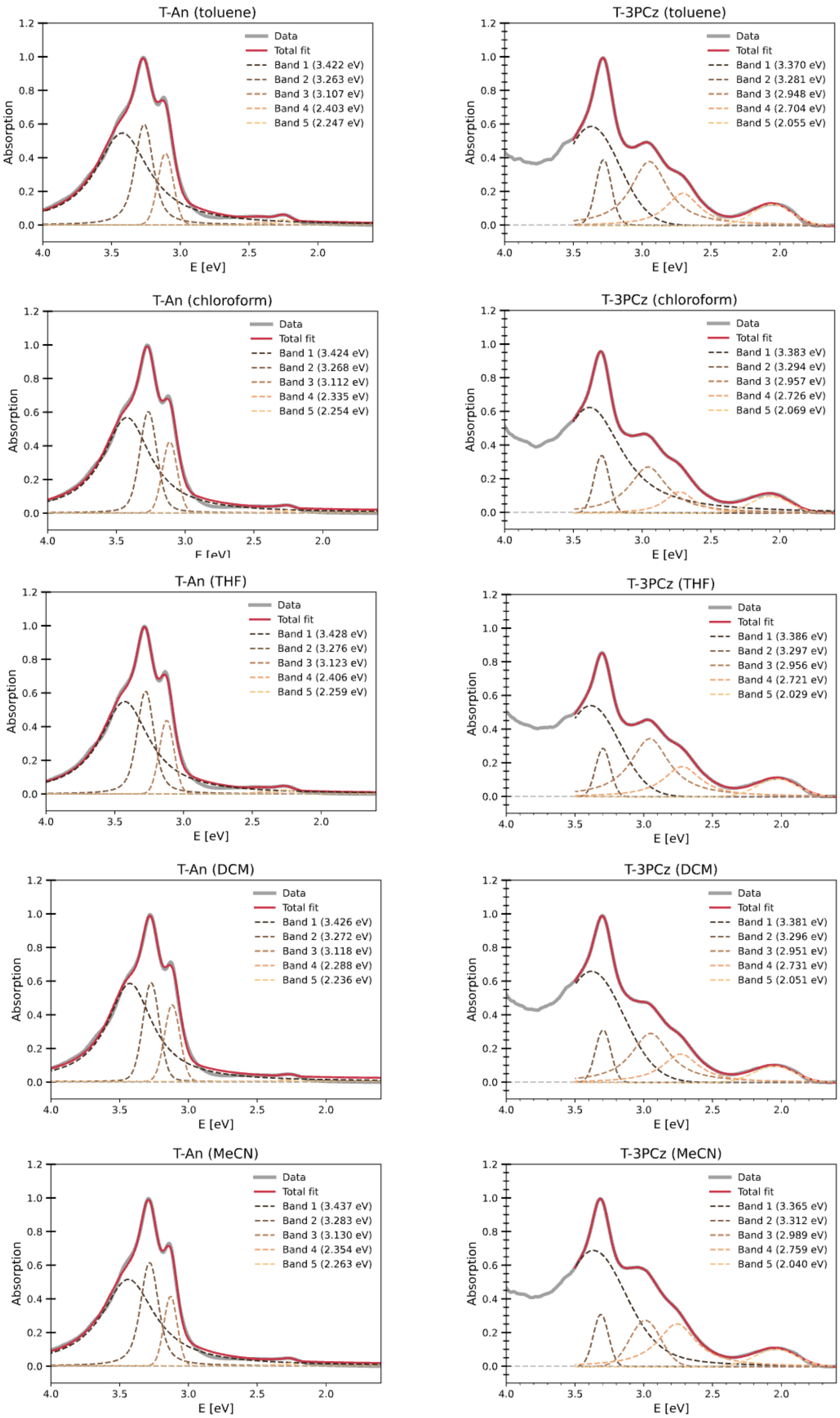

Fig S13. Voigt deconvolution of the absorption spectra for the solvent series of T-An and T-3PCz, measured using an integrating sphere.

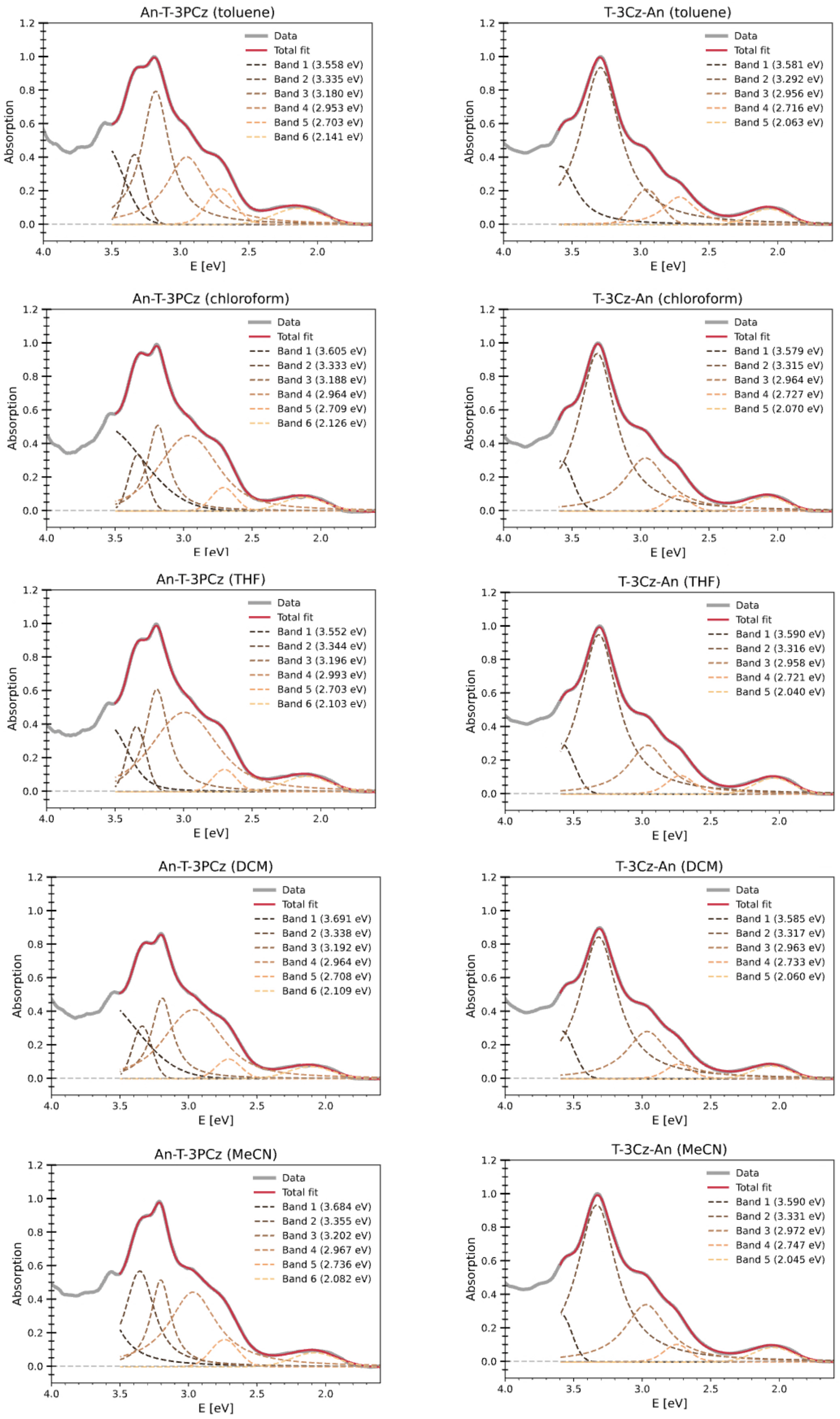

Fig S14. Voigt deconvolution of the absorption spectra for the solvent series of An-T-3PCz and T-3Cz-An, measured using an integrating sphere.

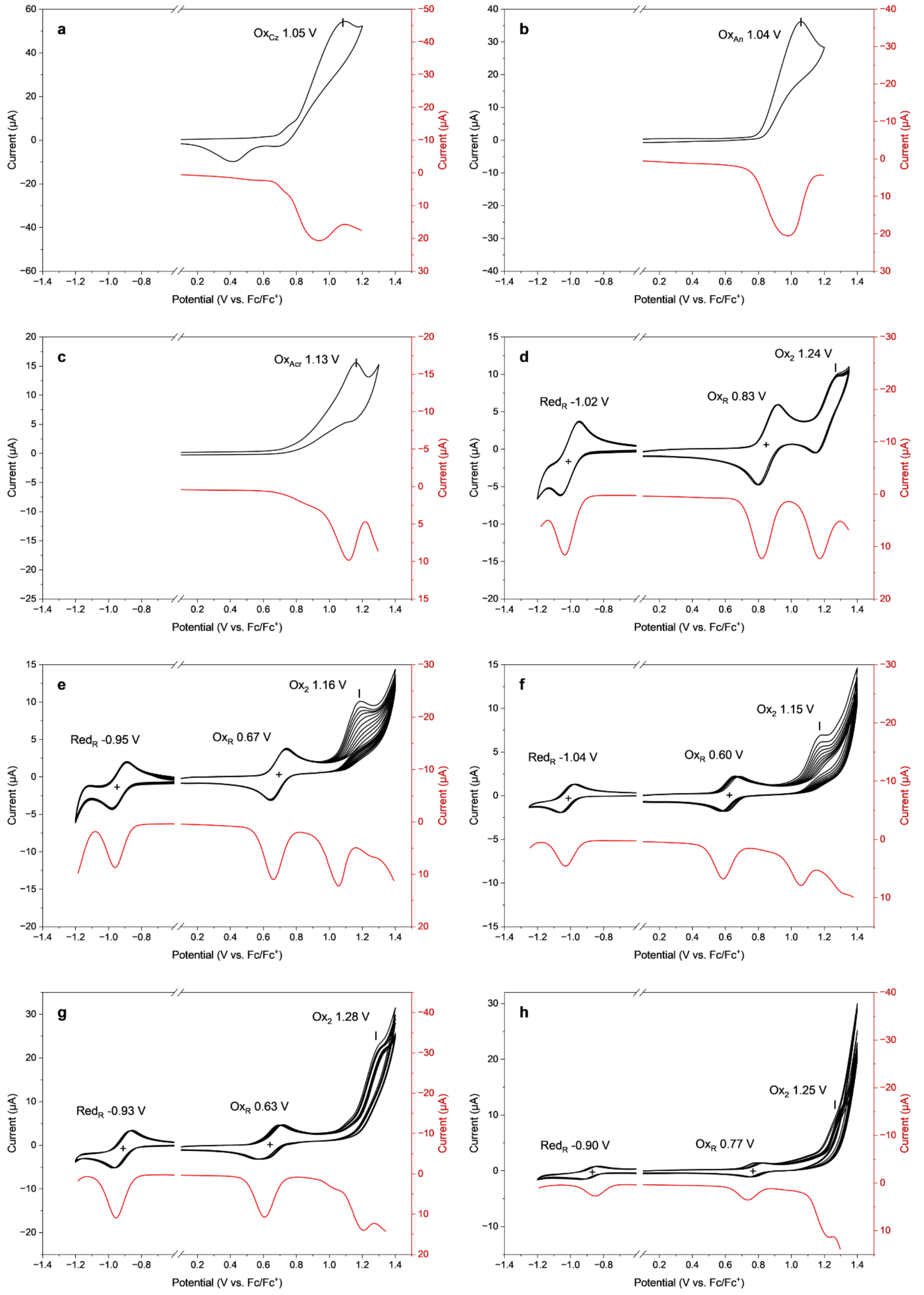

Fig S10. Electrochemistry for the radicals and their subunits. Cyclic voltammetry (black traces) and differential pulse voltammetry (red traces) for **a**, carbazole, **b**, anthracene and **c**, acridine units and **d**, T-An, **e**, An-T-1Cz, **f**, An-T-3PCz, **g**, T-3Cz-An and **h**, T-3Cz-Acr radicals. Cyclic voltammetry shows 10 reduction and oxidation cycles in the cathodic and anodic range, respectively, at a scan rate of 100 mV s^{-1}. The reversible reduction and oxidation waves are assigned to the radical site, R, in (**d–h**) and they are reported as half-wave potentials indicated by plus signs. The second, irreversible oxidation wave is that of carbazole (**a**), anthracene (**b**, **d**), acridine (**c**) or their combination (**e–h**) and it is reported as a peak potential indicated by a vertical line. Differential pulse voltammetry is scanned from the cathodic to the anodic range at a rate of 25 mV s^{-1} and the peaks align well with cyclic voltammetry.

We performed Weller analysis to estimate the energies of the CT states across the series, using the expression:

$$E(\mathrm{CT}) = e(E_{\mathrm{ox2}} - E_{\mathrm{red}}) + \frac{e^2}{4\pi\varepsilon_0}\left(\frac{1}{2r_{\mathrm{T}}} + \frac{1}{2r_{\mathrm{Ac}}}\right)\left(\frac{1}{\varepsilon_{\mathrm{tol}}} - \frac{1}{\varepsilon_{\mathrm{THF}}}\right) - \frac{e^2}{4\pi\varepsilon_0 d_{\mathrm{T-Ac}}\varepsilon_{\mathrm{tol}}}$$

The redox potentials were obtained from CV measurements, and the ionic radii (r_{T}, r_{Ac}) and the ion pair distances ($d_{\mathrm{T-Ac}}$) were obtained from the DFT-relaxed ground state geometries. For T-An, we estimate the energy of the $^2CT_{Ac}$ state using the first reduction and second oxidation potential, corresponding to the addition of an electron to the TTM and removal of an electron from the anthracene unit, respectively. For other materials, the second oxidation potential for carbazole and acene units is overlapped, and the resulting CT energies are an average of both the $^2CT_{Cz}$ and $^2CT_{Ac}$ states. Crucially, for strongly coupled materials, the estimated CT energies are lower compared to their weakly coupled analogues, since the low-lying $^2CT_{Ac}$ state moves the oxidation average to lower values.

Table S1. Redox potentials and the energy of the corresponding electron transfer process, estimated using the Weller equation.[9]

Material	E_{ox} [V]	E_{red} [V]	E(CT) [eV]
T-An	1.24	-1.02	2.24
An-T-1Cz	1.16	-0.95	2.09
An-T-3PCz	1.15	-1.04	2.09
T-3Cz-An	1.28	-0.93	2.33
T-3Cz-Acr	1.25	-0.90	2.27

7.) Discussion is given on lines 185-192 about the requirements for efficient ISC/RISC in these systems; however, there are many inconsistencies in the claims. First, the authors state that efficient SO-ISC requires states with different spatial wavefunctions, which is not necessarily true. It is often the case that weak SOCs between states with similar spatial wavefunctions are overcome by a very small energy gap between the states, leading to rapid ISC (such as transitions between singlet and triplet n-π* states, where J is also small). Vibrationally-assisted ISC is also commonly seen to overcome weak SOCs, which is the known ISC mechanism in bare anthracene. As a result, it is not apparent why the CTAc state is being invoked as an intermediate to ISC/RISC, and the authors should further clarify if possible. Regardless, the authors claim that for the CTAc state to mediate ISC/RISC, it must be thermally accessible from the 4LE and CTCz states and concludes that this is the case for T-3Cz-An on line 191. However, the TD-DFT calculations in Table 3 show that CTAc is 0.4 eV above CTCz for T-3Cz-An in toluene, which is >15x thermal energy at room temperature. Significant thermal population of CTAc under these conditions is unrealistic, which necessarily means that either the mechanistic discussion provided on lines 185-192 is incorrect, or the energies calculated by TD-DFT are inaccurate. Since the energetics are not well understood in general (see point #6 above), it is recommended that lines 185-192 and all other related discussion be removed from the manuscript unless the energies are better quantified.

We concur that our current experimental evidence does not permit us to conclusively determine the (R)ISC mechanism in these systems. We have removed that paragraph from the discussion. However, as explained in our other responses, the experimental evidence points to much smaller $^2CT_{Ac}$-$^2CT_{Cz}$ separations than those found in TD-DFT calculations.

8.) On page S45 of the SI, the authors define the exchange integral; however, there appears to be a mistake in the expression. The position vectors are written in the order r1r1(1/r12)r2r2, but it should be written as r1r2(1/r12)r2r1 to represent an exchange integral. The authors should check to make sure that this is a mistake and fix the ensuing derivations. For example, this same ordering is given in equation 23 and in the first paragraph of page S47.

We apologise for the confusion and we have double checked all the mentioned expressions. We confirmed that they are written as intended, since we have defined the two-electron integrals in "chemists' notation", where the position vectors are always in the order r1r1(1/r12)r2r2. In Szabo and Ostlund's "Modern Quantum Chemistry" (SI Reference 18) this definition is given given in Table 2.2. This has been clarified on page S61 of the revised SI in the discussion around Eq (15).

9.) On the bottom of page S53, the authors caution that their derivations are not accurate for systems where the dyad orbitals contain significant mixed contributions from both the acene and radical. However, the authors claim that the equations should apply to the molecules in this work. Can more reasoning be given for this? This would appear to be counterintuitive to the claims made throughout the manuscript that An-T-1Cz and An-T-3PCz exhibit strong acene-radical electronic coupling, and therefore their frontier orbitals should mix fairly significantly. This is another reason why it is important to define what is meant by "electronic couplings" and have estimates of the electronic coupling strengths between the relevant orbitals. Additionally, just from inspection of the NTOs in Figures S28-31, there seems to be quite a bit of delocalisation of the dyad orbitals onto both the radical and acene fragments.

We thank the referee for eluding to this limitation. As outlined in answer to question 1, we have now clarified what we mean by radical-acene electronic coupling with reference to the two types of interactions, the coulombic attraction and the TTM-acene electronic coupling. We have also quantified the strength of these interactions.

The Coulomb term is less sensitive to the small amount of mixing of radical and acene orbitals as for this term to be large it does not require that the electron and hole orbitals overlap, only that they are in close proximity. The argument that the Coulomb stabilisation of $^2CT_{Ac}$ is larger when the acene is directly linked to the TTM is supported by both energies from TD-DFT (for the whole molecule, accounting for mixing of orbitals) and values of Coulomb integrals extracted from our semiempirical model (using molecular fragments and localised orbitals), and is in line with electrostatic arguments concerning the separation of electron and hole orbitals.

The electronic coupling (Eq. S27b) on the other hand would have to be treated using a different method depending on the choice of localised orbitals (coupling via local orbitals interacting through bond) or delocalised orbitals (coupling via overlap of orbitals) and so would likely be more sensitive to this choice of orbitals. However, we have chosen to treat the coupling in Eq. S27b) using localised orbitals as a first approximation to the coupling as the simplest and most

practical way of capturing the qualitative picture of the physics of the system. Our results can be best explained with Coulomb stabilisation of $^{2}CT_{Ac}$ playing the primary role in determining the optoelectronic behaviour in these systems.

Minor comments

10.) The authors state on line 20 that the PLQYs of defects are typically low. What do the authors mean by low? Plenty of reports as early as 1997 (Science 1997, 276, 2012) have demonstrated single-particle photoluminescence imaging and single photon emission from NV centres, which requires high PLQYs. Additionally, ref. #2 that is cited in the previous sentence includes a discussion about the large PLQYs of defects in diamond.

Our intention was to draw attention to the fact that PLQYs of defects tend to degrade when integration attempts are made. This can be expected due to removing the encapsulation provided by the surrounding host crystal matrix. We now summarise the key extant challenges as follows: "However, their excellent optical properties may be degraded through nanofabrication required for their miniaturization.[3,4] Control of interactions between multiple defects and their deterministic formation also remains challenging.[5]"

11.) On line 27, the authors should be careful when making the statement that typical quartet-forming chromophore-radical systems are non-luminescent. In at least one recent report, they have been shown to be luminescent (Angew. Chem. 2023, 135, e202214668), the yields are just low since EISC and EIC are fast compared to the radiative decay of the photoexcited chromophore. Further, there are reports by Kandrashkin (J. Phys. Chem. A 2006, 110, 9607-9616) and Gouterman (J. Chem. Phys. 1970, 52, 3795-3802) that demonstrate luminescence directly from the 2,4LE states with moderate yields. These points should be addressed in the manuscript, and the statement on line 27 rephrased with them in mind.

We respect prior and concurrent work in the area of radical-chromophore dyads, and regret that our original text obscured this fact. We have clarified the statement in question to specifically refer to non-organometallic compounds: "… such compounds have thus far been non-luminescent <u>when constructed from fully-organic units,</u> …", which we believe now makes it accurate and appropriate. Kandrashkin 2006, Gouterman 1970 and others report weak emission in organometallic compounds, where the high SOC of the metal atoms can lead to increased spin decoherence, limiting scope for room temperature operation. Inclusion of the metals can also limit biocompatibility and chemical tunability. Therefore, we focus on fully organic structures. Of such compounds, as surveyed in Ref. 9 and reported since, none have shown delayed emission. Angew. Chem. 2023, 135, e202214668 reports a compound with a PLQE of 1%, with no time-resolved emission data available. Based on the observed TA lifetimes of BDPA-tpPDI and known luminescence lifetimes of PDI derivatives, this very likely originates from prompt emission that competes with processes leading to quartet generation on timescales of a few nanoseconds. The central message of our manuscript is the demonstration of how the luminescence yield can be boosted above 50% despite EISC, EIC and similar pathways being present. This is enabled by managing an energetically accessible back-pathway to the initially photoexcited state, leading to microsecond delayed emission lifetimes, which offers unique opportunities for interfacing as demonstrated in Ref. 23.

12.) On line 31, the authors state that only a single design of TTM-chromophore is known; again, the authors should be careful in making these claims. One recent example (Chem. Eur. J. 2024, 30, e202403244) even explored the structure-property relationship; a different one (J. Am. Chem. Soc. 2025, 147, 1, 1017) explored and explained in far better detail the mechanism proposed in this manuscript.

We apologise for the overly broad phrasing, and concur that the language in that line requires more precision. We have revised this line to read: "As only a singular design of TTM radical-chromophore dyads with efficient delayed luminescence is currently known..."

We respectfully disagree with the Reviewer regarding their view of how our manuscript relates to the two works mentioned. Chem. Eur. J. 2024, 30, e202403244 demonstrates a series of PDI-trityl dyads which are not luminescent. Additionally, the spin multiplicity of the excited states is not experimentally studied or discussed. We find that this work has limited relevance to our manuscript. J. Am. Chem. Soc. 2025, 147, 1, 1017 reports trityl-based upconversion, but does not provide experimental evidence for the spin multiplicity of the excited states. We show here that these spin properties are crucial for understanding the behaviour in TTM-chromophore materials, particularly the dynamics on microsecond timescales. Furthermore, their system relies on an intermolecular process, while ours features exclusively intramolecular behaviour. We foresee that further work might reveal interesting parallels between these compounds, but at present this has not been established.

13.) The doublet-triplet resonance condition and why it's important should be explained in a sentence in the introduction. Otherwise, it is not clear why the resonance condition is important until the very end of the manuscript, which may make some of the earlier discussion difficult to follow for readers that are not familiar with the work in ref. #10.

We agree that the introduction would benefit from this, and have added the following at Line 37: "Delayed luminescence was enabled by minimising the excited state energy gaps, through a resonance between the doublet and triplet excited states of the dyad units."

14.) A reference to Figure 1d is made in the text, however, there is no panel D.

We sincerely apologise for this, and other typos, and have updated the text there to reference Fig. 1c as intended.

15.) Is there a reason the authors have chosen to mesitylate An-T-1Cz, An-T-3PCz, and T-An, but not T-3Cz-An and T-3Cz-Acr? On a related note, a reference to the T-An structure in the SI should be made on line 53; otherwise, it was very difficult to find the structure. Additionally, why did the authors choose to mesitylate the 10-position of anthracene in T-An instead of leaving it bare, like in all of the dyads discussed in the main text? This will certainly impact the electronics of the anthracene, which counters the purpose of using the compound as a direct control.

Starting from the T-An structure, we introduced a mesityl group to the anthracene 10-position for steric stability. This is best seen in cyclic voltammetry (Fig. S10), where oxidation of anthracene remains reversible when mesitylated due to steric protection of the cationic An radical. Mesitylation of the radical site is a common strategy to enhance stability and

luminescence (*Nat. Commun.*, 2023, 14, 4147). Moving on to the carbazole coupled radicals, we employed similar strategy but deemed protection of the anthracene site less critical due to the introduction of another donor and a $^2CT_{Cz}$ state. Molecular weight of the structures is another important factor, and to keeping it as low as possible for support future vacuum deposition of solid-state systems, we decided to trim down the substituents where possible. The champion compounds T-3Cz-An and T-3Cz-Acr would exceed 1000 g/mol if both the radical and the acene sites were mesitylated. Bulky substituents smaller than mesityls could be studied in the future, but such screening goes beyond the scope of this work.

Inspection of the absorption spectra of mesitylated M_2TTM-3PCz (*Chem. Sci.*, 2025, 16, 17356-17368) and of non-mesitylated TTM-3PCz in toluene solutions (Fig. S12) reveals matching lineshapes with absorption onsets within 10 nm, indicating negligible effect of mesitylation on the electronic structure. The line now reads: “Throughout this work we also use the anthracene-free T-3PCz and the newly synthesised carbazole-free T-An as control molecules (Fig. S11).”

We specifically investigated the effect of mesitylation in T-An using TD-DFT in structures that differ only at the 10-position of anthracene: a proton in M_2T-An-H, or a mesityl group for M_2T-An-M (which is the experimentally investigated structure referred to throughout as T-An). The results (Fig. S53) reveal that the presence of the mesityl group does not affect the excited state energetics, state ordering, oscillator strengths, or their degree of LE/CT character. This confirms that T-An, as synthesised, represents a suitable control compound.

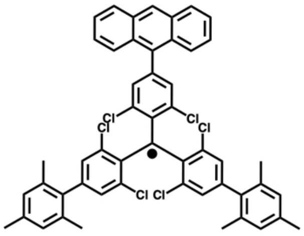

	TOL			
	Energy (eV)	Osc. strength	<S²>	Λ
$^{2,4}LE_{Ac}$	**2.06**	**1x10⁻⁴**	**2.71**	**0.76**
$^{2}CT_{Ac}$	**2.26**	**0.008**	**0.89**	**015**
	2.82	0.019	0.91	0.48
	2.84	0.016	0.92	0.53
	2.99	0.007	0.88	0.42

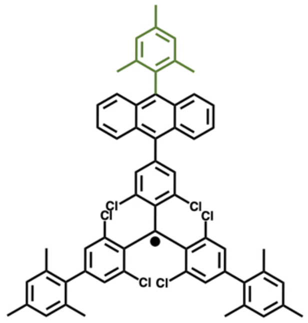

	TOL			
	Energy (eV)	Osc. strength	<S²>	Λ
$^{2,4}LE_{Ac}$	**2.01**	**0**	**2.75**	**0.75**
$^{2}CT_{Ac}$	**2.20**	**0.003**	**0.85**	**0.10**
	2.83	0.019	0.91	0.45
	2.84	0.022	0.92	0.53
	2.99	0.007	0.88	0.41

T-An
as synthesised

Fig S53. Effect of mesitylation of anthracene on electronic properties.

16.) The authors claim on line 61 that the dominance of the RISC channel is desired to allow time for quartet addressability, which does not seem to be correct. The main advantage of this pathway is that it maximises the PLQY of the radiative channel while still forming high yields of quartet, which typically quenches emission (see point #11). The “time for quartet

addressability" is only dictated by the quartet lifetime and T1 relaxation, which will necessarily always be short as long as RISC is efficient and competitive. Can the authors clarify?

We agree fully with the above statement by the Reviewer. Our intention was to point out that delayed emission is the key feature of our systems, and their applications would not be possible in materials with only prompt fluorescence. We revised the text to more directly point to the importance of this channel: "As this follows quartet-doublet reverse intersystem crossing (RISC), the dominance of this channel over prompt emission is desired to prolong access to the quartet exciton." We also explicitly refer to this trade-off in the Discussion, Line 213.

17.) Line 72, what is the justification for the claim that conformationally locking the molecules in a rigid medium would lead to a time-dependent blue-shift of the emission as opposed to a static blue-shift, which is what is typically observed?

We observe a small emission blue-shift on slow timescales in films. Due to a range of dihedral angles available between the T, An and Cz units, and the deep red/onset NIR emission wavelength region, we propose this is likely due to a conformational disorder mechanism. Faster nonradiative decay (due to the energy-gap law) and slower radiative rates (due to poorer orbital overlap for RISC) are seen in the low-energy tail of the molecular ensemble. We have added two new references (J. Mater. Chem. C, 2017,5, 11001-11009; J. Phys. Chem. Lett. 2022, 13, 7, 1839–1844) and expanded the explanation in the main text: "When the molecules are immobilized in a rigid medium, we observe small time-dependent hypsochromic shifts in all molecules, which we ascribe to conformational disorder modulating the rates of RISC and non-radiative decay across the energy range of the ensemble.[28,29]"

18.) Also, line 72, the authors say "effective lifetime" but do not define what this is. This also appears in Tables S2-4, but I cannot find if it is mathematically defined anywhere.

We include the following in the caption of Table S3: τ_{eff} (effective lifetime) is defined as an intensity-weighted average of decay times:

$$\tau_{\text{eff}} = \frac{\sum_i A_i \tau_i^2}{\sum_i A_i \tau_i} = \sum_i f_i \, \tau_i$$

where A_i, τ_i and f_i are the amplitudes, lifetimes and normalised weights of individual decay components, respectively.

19.) The authors claim in lines 86-87 that An-T-1Cz, An-T-3PCz, and T-An decay mostly from states of similar character. However, it is clear that the spectral features of T-An are quite different from the other two compounds. In Figure S18a, what are the TA features for T-An being assigned to? If the feature at ~625 is assigned to anthracene cation, why is it so shifted from the anthracene cation features (~700 nm) for An-T-1Cz and An-T-3PCz in Figures S18b,c? This point illustrates the need to perform spectro-electrochemistry on the compounds to correctly assign each ion band.

As explained above in answer to question 4, the PIAs of both the $^2CT_{Ac}$ and $^2CT_{Cz}$ states include a minimum near 625 nm but differ in their lineshape near 700 nm.

Following the reviewer's suggestion, we have attempted to measure spectroelectrochemistry on the radical structures. However, as mentioned in our response to comment 6, the overlapping oxidation of carbazole and acene sites in An-T-1Cz, An-T-3PCz, T-3Cz-An and T-3Cz-Acr prevents us from reliably distinguishing between absorption features of cationic carbazole and acene units in this method. We have also attempted measuring carbazole, anthracene and acridine monomers. This resulted in oxidative electrochemical polymerization which is well-known to occur via carbazole 3,6-positions and anthracene 9,10-positions, and thus free oxidized species could not be observed in our experiments. On a practical note, it makes sense that the donor molecules are reactive in their electrochemically oxidized cationic radical form, unlike TTM radical and its derivatives whose reversible oxidation and reduction allows spectroelectrochemistry (B. Li, et al., Nature Materials 2025, DOI 10.1038/s41563-025-02362-z). We would like to emphasize that electrochemical oxidation of the radical site (formation of a TTM cation) followed by oxidation of the donor site (TTM cation + donor radical cation) is distinctively different form photoexcitation of T-An, An-T-1Cz, An-T-3PCz, T-3Cz-An and T-3Cz-Acr in that the CT state is stabilized by a hole on the donor site and an electron on the radical site.

20.) On line 109, it says that "excited state" signals were not observed for T-An in the trESR data. The way this statement reads is a bit unclear because it does not rule out the observation of ground-state polarisation. The authors should clarify if no signal at all is observed.

We modified this line to: "We did not detect any excited or ground state polarised signals in the trESR of T-An, indicating that non-radiative relaxation outcompetes ISC, and quartet states are not formed."

21.) The inversion of the trESR signals at later times is likely due to a combination of spin-selective depopulation of the quartet sublevels, T1e relaxation processes, as well as saturation driven by the cw microwaves. Is the temperature dependence of the population inversion only being ascribed to the RISC mechanism as with the delayed emission? If so, how do you know it doesn't have significant contributions from T1e decay between the sublevels as well?

It is true that the inversion of the quartet polarisation pattern in trESR has the additional contributions listed. We verified that the measurements were performed in the limit of weak microwave drive. All measurements in Fig. 3e were collected at the same low microwave power (40 dB attenuation), and the tuned Q-factors were within 5% of each other. Measurements on T-3Cz-An performed at 80 K at the same field position as Fig. 3e reveal that the inversion time remains nearly unchanged at powers lower than 40 dB, while it shortens at higher mw powers. While the probed temperature range here is quite small, we cannot exclude a contribution from variation in T1e relaxation rates. Gaining a complete understanding of the polarisation inversion dynamics would require a large set of temperature dependent pulsed ESR measurements. The activation energy extracted from delayed doublet luminescence is the more accurate measurement, however we find it is useful to point out that the dynamics of the quartet sublevel populations qualitatively seem to follow a similar activation profile.

We revised the relevant lines to: "At later times, the polarization of the broad transient signal flips, as previously observed in quartet-bearing molecules. (Appl. Magn. Reson. 30, 619-636,

2006) This indicates that decay rates of the quartet sublevels are asymmetric, and in our systems is due to RISC favouring the $m_s = \pm 1/2$ sublevels. The polarization inversion occurs at earlier times at higher temperatures (Fig. 3e), following the temperature-activated delayed emission dynamics. However, contributions from other temperature-dependencies (primarily spin-lattice relaxation times) hinder its quantitative analysis."

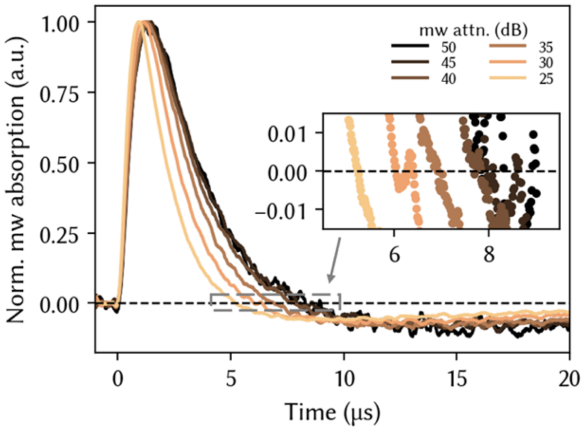

Fig S43. Microwave power dependence on recorded X-band trESR of T-3Cz-An at 80 K. Monitored at field of 328 mT, corresponding to the maximum of the X polarisation.

22.) Lines 122-123, the authors assign the initial absorptive feature in the trESR spectrum of T-3Cz-An to population of the CTAc state during ISC to from 2,4LE. The evidence for this is the "similarities with timescales" for the optical and trESR data. This claim provides no rational and does not make sense. First, optical data shows a 4 ps rise of the 2,4LE feature in the TA (Figure 2d, line 92). The trESR experiment does not have sub-nanosecond resolution to observe this, IRF is likely closer to a 50-100 ns. The ~4 ps component would need slow down by ~10,000x at 80 K in order to be resolved by trESR. This is unlikely, but regardless, TA data was not collected at 80 K to provide evidence for this. Second, as stated above, it is unclear why the CTAc state is being invoked as an ISC intermediate in T-3Cz-An when there is no spectral evidence of its population and TD-DFT calculates it to be ~0.4 eV higher in energy than CTCz in toluene (Table 3).

While optical methods are not able to distinguish between the ^{2}LE and ^{4}LE states due to their small energy, the 4 ps lifetime corresponds exclusively to the generation of the ^{2}LE which is the only state that can form with spin conservation. As evidenced by the TADF-like temperature dependent delayed luminescence dynamics, the forward process does not encounter an activation barrier at those 80 K. The remaining question then becomes the mechanism of ^{2}LE → ^{4}LE conversion. The timescale of this process is comparable to the prompt emission lifetime (tens of ns). We observe an additional doublet polarised signal in the ESR at these times, which are indeed close to our IRF. This quickly lost signal is unlikely to originate from ground state D_0 polarisation, as the T_{1e} time of the TTM radical is on the order of ms. Among the doublet states, $^2CT_{Ac}$ is the only unaccounted for excited state. As our additional optical experiments show it is located much closer than the calculated 0.4 eV to the other states, the best explanation we have for this signal is that it may be a signature of $^2CT_{Ac}$ polarisation, which may develop at some rotationally locked conformations in the frozen solution. We concede that further work on the conclusive identification of the ISC mechanism is required in the future, and have softened the language in the discussion to reflect that.

23.) Line 124, the authors reference Figure S25 for their claim of high quartet yields, but that figure in the SI has nothing to do with quartet yields, it simply verifies the quartet state by displaying the double and triple quantum transitions. Otherwise, this figure isn't referenced in the main text, but it should be because it is some of the strongest evidence for quartet formation in these systems.

We now refer to the half-field transient data in Line 127: "Their state multiplicity is validated by resolving a broad $\Delta m_S = 2$ and a narrow $\Delta m_S = 3$ half-field signals in T-3Cz-An (Fig. S41)."

24.) Why was pulse-ESR only performed on T-3Cz-An and not T-3Cz-Acr? The other two dyads have poor S/N in trESR, so it is understandable why it was not done for them.

We have performed pulsed ESR on T-3Cz-Acr. We include the following transient nutation as a function of field figure in the SI.

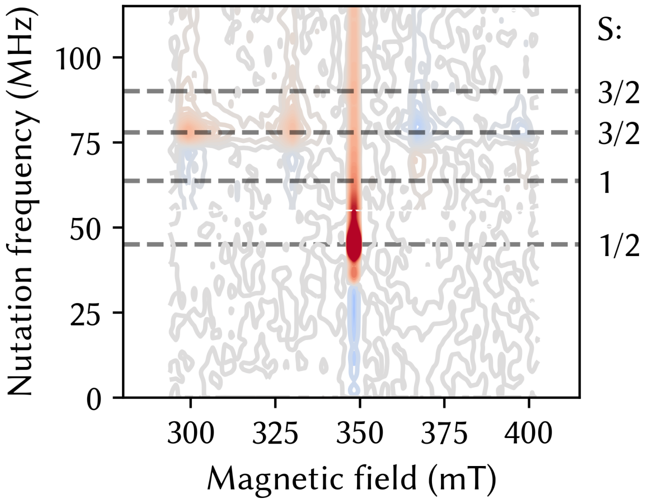

Fig S46. Transient nutation experiment on T-3Cz-Acr at 80 K.

25.) Line 132, there appears to be a typo. I believe the compound should be written as "T-3Cz-An" and not "T-An-3Cz."

We apologise for this mistake, and have corrected it to "T-3Cz-An".

26.) A general comment, the optical data would be much easier to follow if the TD-DFT calculations or other discussion of state energetics were presented first.

We agree that explaining the energetics earlier would be beneficial, however we believe presenting experiments before calculations is necessary. We now include a more extended text on state energetics at the end of the introduction:
"The 0-0 energies of both CT states are within 1.9±0.2 eV, but are highly sensitive to the molecular topology and environment polarity.[25] The closely energetically spaced pair of LE states is pinned to the level of its acene triplet component.[11] The energetic ordering of these four states is crucially important for the performance of our materials as we discover below."

27.) Lines 174-176, the CTAc state is again invoked as a mediator for conversion of 2D1S0 to 2D0T1, although this time it is claimed that it does so "indirectly." It is not clear how this fits into the rest of the discussion in the manuscript, where it is implied that CTAc is directly populated. Again, given that the energetics of these states are not thoroughly investigated, it is advised that the authors remove this and related discussion from the manuscript unless the energetics can be more reliably quantified, or more rigorous evidence is provided for the involvement of CTAc in the ISC/RISC mechanism.

We have removed the sentence in question.

28.) Line 201, the authors claim that the molecular topology modulates electronic rather than exchange couplings. However, exchange couplings are not calculated or measured in these systems, so this claim should be removed from the manuscript. Particularly because it is well known that molecular connectivity and orbital overlap drastically change exchange couplings. On a similar note, what evidence is there for the claim on line 203 that strong exchange couplings can be maintained for distances exceeding 2 nm? This seems like an arbitrary statement.

We agree that this paragraph required improvements in clarity. We certainly agree that exchange couplings are highly sensitive to connectivity. We add new calculations at NEVPT2 level of theory which show that closer linkage increases the magnitude of the triplet-radical exchange coupling (Table S9). Despite these differences, crucially, all 4 compounds are in the same strong exchange coupling regime, as shown by EPR experiments and simulations. Determining the regime of triplet-radical exchange coupling is well established in the literature on dyad systems (Quintes et al., Nat. Rev. Chem. 2023, 7, 75-90). Within the strong exchange regime, the X-band EPR spectra do not allow determining the exact value of the exchange coupling strength. In this line we thus wish to draw attention to the fact that, for the set of molecules presented here, it is the differences in electronic rather than exchange couplings that determine the key properties. We have removed the reference of a specific distance on the line in question. We now write: "Comparing An-T-3PCz and T-3Cz-An shows that molecular topology has greater consequences in modulating the electronic rather than exchange coupling landscape in these materials, since the strong exchange regime is reached already under a relatively small interaction. Due to the contribution of the TTM SOMO to the delocalised pi-system,[13,54] we expect strong exchange coupling can be maintained in larger molecular structures."

29.) In the caption of Figure 4, the CTAc NTOs are discussed and the statement is made that hole-particle delocalization increases from An-T-3PCz to T-3Cz-An, which in turn enhances electronic coupling to other states through wavefunction overlap. This contradicts the claim made throughout the paper that T-3Cz-An has the weakest electronic coupling and is the reason why ISC/RISC is so effective. The authors should be clear with definitions of the various electronic couplings they discuss in the paper, as this may just be a case where two different types of electronic couplings are being invoked.

We apologise for the confusion and agree this discrepancy needs to be clarified. We are referring to the extent of the delocalisation of the $^{2}CT_{Ac}$ state via carbazole, not the Coulombic stabilisation responsible for the energy of this state. We rephrased the caption to read:

"Calculated $^{2}CT_{Ac}$ state NTOs show that its extent of delocalisation is larger in T-3Cz-An than An-T-3PCz."

30.) What CW microwave power was used to collect trESR data? This should be specified in the methods section.

We added the following to the methods: "Unless otherwise specified, the trESR was collected at 40 dB microwave attenuation (ca. 20 μW mw power)."

31.) At what time, following laser excitation, were the reported trESR spectra collected?

We take as 'prompt' spectra the average signals from 0.5 until 1.5 μs following laser excitation. We have specified this in the text and figure captions.

32.) Figure S11, top right panel. I believe the compound should read "An-T-3PCz" and not "An-T-3Cz"

We corrected this to An-T-3PCz.

33.) Why is the temperature-dependent emission data in PMMA for T-3Cz-Acr not shown in the SI as well?

This is now included.

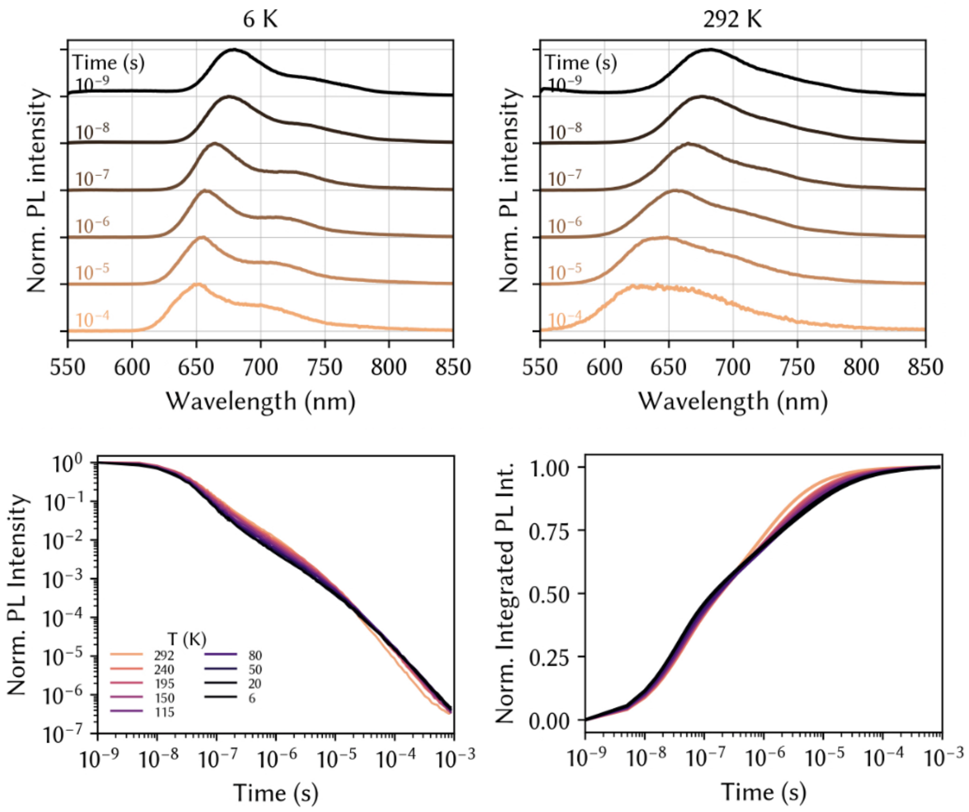

Fig S28. Temperature-dependent emission profile (top) and kinetics (bottom) of 5% T-3Cz-Acr in PMMA thin film.

34.) Figure S24 caption, I believe the compound should read "An-T-1Cz" and not "An-T-3Cz"

We corrected this to An-T-1Cz.
35.) Figure S26 caption, define "DAF"

We have expanded this to Delay After Flash and specified its value in the caption.

36.) When NTOs are displayed, a corresponding λ value should be shown that designates the contribution of that hole-particle pair to the total excitation.

The complete set of NTOs in the Supplementary Figures now all show the λ value.

****

Reviewer #2 (Remarks to the Author):

Quartet states (S = 3/2), enabled by radical-triplet interactions, merge high-spin quantum properties with potential applications in quantum sensing and optoelectronics. However, such states are typically non-emissive, and only a few luminescent radical-chromophore dyads (e.g., TTM derivatives) are known to demonstrate stable quartet generation. As a result, a general structure-property design principle remains elusive, making the engineering of robust quartet systems a critical challenge.
In this work, Matasovic and coworkers designed two classes of compounds: one exhibiting weak radical-chromophore coupling and the other demonstrating strong coupling. The strongly coupled system achieves modest PLQY, while one of the weakly coupled variants (T-3Cz-An) displays predominantly delayed emission and remarkably slow quartet depletion dynamics. Based on TD-DFT calculations, the authors found that in weakly coupled systems, a bright carbazole-to-TTM charge-transfer state (2CTCz) lies above the quartet state. However, in strongly coupled systems, a dark acene-to-TTM charge-transfer state (2CTAc) lies above the quartet state and acts as a dark trap, significantly reducing efficiency. Using these insights, the authors developed general energetic guidelines to optimise quartet state generation in luminescent radical-chromophore systems. This work is very interesting and important! Before publication, the authors may need to address the following concerns.

We thank the Reviewer for their careful reading of our work and are pleased to see that they find it exciting.

1. The main text references 'Fig. 1d' in the discussion, but this panel does not appear in the provided Figure 1.

We apologise for the confusion caused by this typo. We have updated this to reference Fig. 1c.

2. In T-3Cz-An, the authors observe a significant drop in delayed emission lifetimes (~10x) when moving from toluene to a polar solvent (MeTHF), which they initially attribute to excited-state energy reordering. However, their theoretical calculations suggest the polar solvent does not alter the relative ordering of states (2CTAc still remains ~0.24 eV above 2CTCz). If 2CTAc is still significantly higher in energy, its ability to act as an effective dark trap and strongly quench

delayed emission becomes unclear and requires further justification. Moreover, in T-3Cz-Acr, the 2CTCz-2CTAc gap shrinks from 0.7 eV (toluene) to 0.4 eV (MeTHF), it needs to be clarified whether this reduced gap similarly influences the delayed lifetime. In general, it is essential to clarify how molecular topology tunes energy gaps and non-radiative decay for understanding the structure-property relationship in this case.

We thank the reviewer for the comment, and we understand their point: DFT has its drawbacks, especially when used for excited-state calculations, and when using unrestricted Kohn-Sham formalism to describe open-shell systems. Our intent here was not to provide absolute values to match experimental ones, but to describe the different energy ordering in molecular systems with a different connectivity in the TTM group. For such reason, our calculations relied on an scheme which uses a range-separated hybrid functional (specifically designed for the characterization of CT states) and introduces screening effects to mimic the polarization given by the environment.

To clarify the state ordering, we performed additional ultrafast optical experiments. We use Kerr-gated fluorescence spectroscopy on dilute toluene solutions of T-3PCz and T-3Cz-An in non-polar (toluene) and polar (DCM) solvents. The experimental capabilities of our equipment allow us to probe the 0.3-150 ps time region and 630-810 nm luminescence region. The detectivity of our instrument only permits the study of bright chromophores. We select 460 nm excitation to minimize 0th and 1st order diffracted scatter in the probed fluorescence region.

In toluene, the spectra of both compounds show minimal and comparable evolution, and match the steady-state luminescence. Differences are visible in the kinetics, with T-3PCz showing approximately constant intensity of luminescence across this time window. This indicates that there are no additional dynamics involving the $^{2}CT_{Cz}$ state beyond minor stabilisation upon solvent reorganisation. In T-3Cz-An the intensity of PL decays with a lifetime of 6 ps, consistent with the depopulation of $^{2}CT_{Cz}$ during the generation of $^{2}LE_{Ac}$ as seen in transient absorption. Interestingly, the weak PL visible long after that process (60 ps) is blueshifted by 40 nm relative to the 10 ps spectrum. If this PL originates from $^{2}CT_{Cz}$ states which did not complete the energy transfer due to unfavourable TTM-Cz and Cz-An conformations, we expect the same spectrum as the solvated T-3PCz. Thus, this emission may have a contribution from the $^{2}CT_{Ac}$ state situated just above the $^{2}CT_{Cz}$ state.

This hypothesis is corroborated by the polar solvent data, which reveals important differences between the two materials. As expected from steady-state data, T-3PCz shows an approximately 50 nm redshift in DCM compared to toluene. This develops across the first 50 ps, but the emission lineshape is preserved. We observe a reduction in PL intensity reaching a minimum around 20 ps, which recovers to 80% of the prompt intensity by 100 ps. In T-3Cz-An, the prompt spectrum of the $^{2}CT_{Cz}$ state is similar to the one observed in T-3PCz. We observe an emergence of a second emission band, peaking around 760 nm, which emerges during the first few ps. This might indicate population transfer to a low lying $^{2}CT_{Ac}$ state. The spectrum at 60 ps is close to the instrument noise floor, matching the fast non-radiative decay dynamics observed in transient absorption.

We refer to this data in the main text: "Kerr-gated transient photoluminescence of T-3Cz-An reveals population of a red-shifted luminescence band on ps timescales in DCM (Fig. S20). This

band is absent in toluene or in T-3PCz, suggesting it originates from a rapidly populated, solvent-stabilised $^{2}CT_{Ac}$ state."

Further experiments on state energies are presented in response to Reviewer 1 Comment 6.

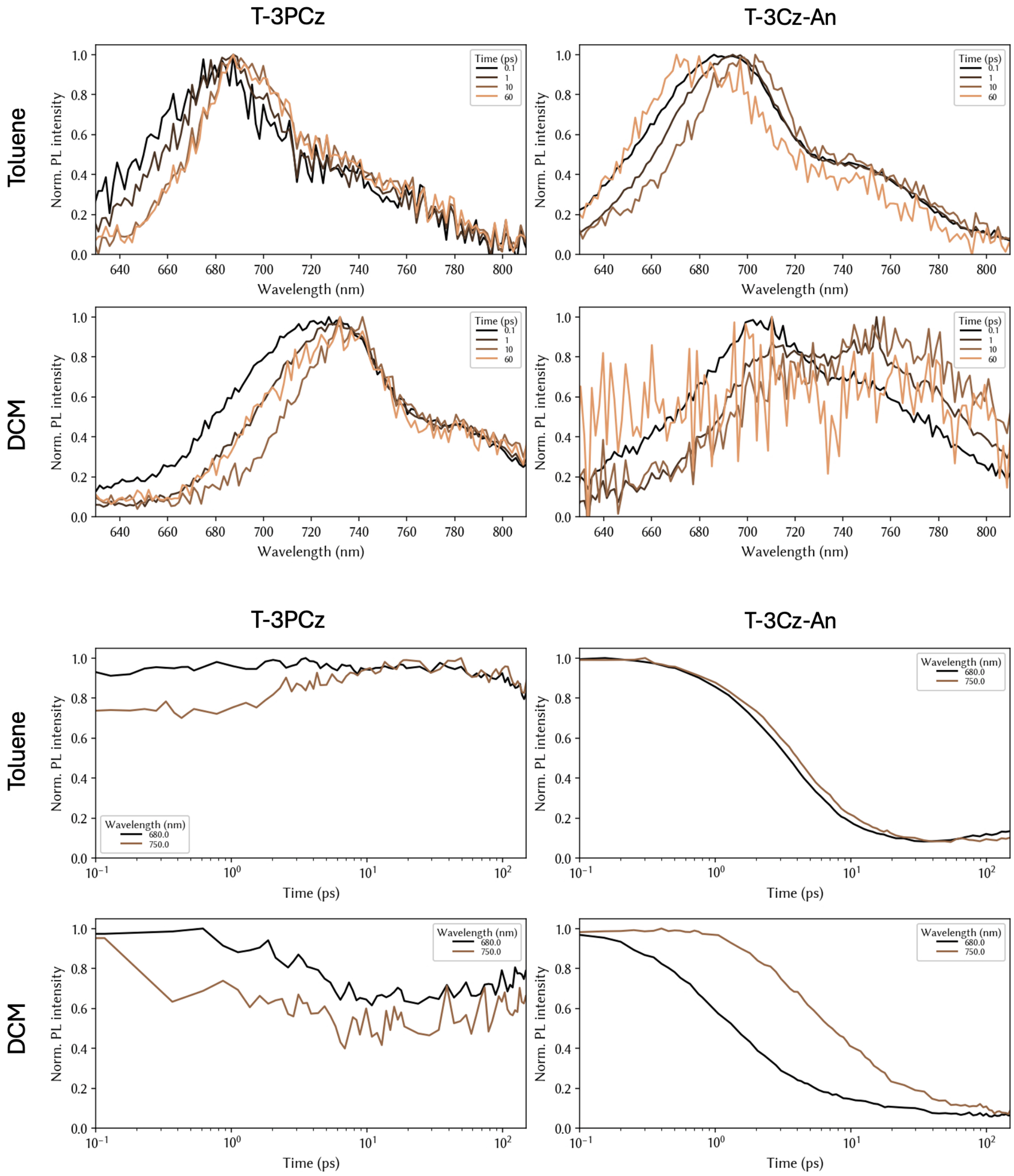

Fig S20. Kerr-gated transient PL spectra (top) and kinetics (bottom) of 0.1 mM toluene and DCM solutions of TTM-3PCz and T-3Cz-An following 460 nm excitation.

3. Based on perturbation theory, the authors suggest that large orbital exchange interactions are beneficial for stabilising the quartet state relative to the emissive radical state. Additionally, they propose that the energy of the 2CTAc state can be raised by designing systems with spatially separated radical SOMO and acene HOMO, alongside spatially proximate acene HOMO and LUMO. While these design principles may be theoretically useful, the manuscript would benefit

from their further validation on the molecules discussed in this study. In particular, calculating the exchange and Coulombic interactions for the two classes of molecules would help substantiate the proposed energetic criteria.

We thank the referee for this helpful suggestion. We have now calculated the exchange coupling (and the resulting energy splitting), Coulomb integrals $J_{A1,R0}$, and the electronic coupling strengths between the radical and acene units in the reported compounds. These are presented in the new main text Table 2, and Tables S9 and S10 in the Supplementary Material, and can be summarised as:

Molecule	Coulomb stabilisation / eV	Electronic coupling / meV	Exchange coupling / μeV
An-T-1Cz	1.17	36.3	1300
An-T-3PCz	1.17	29.6	1300
T-3Cz-An	0.75	6.0	-7
T-3Cz-Acr	0.67	12.7	23

We believe that these numerical results significantly strengthen the paper, by providing additional evidence that Coulombic interactions, modulated by the connectivity of the radical-acene dyads, control the behaviour of these systems.

****

Reviewer #3 (Remarks to the Author):

I co-reviewed this manuscript with one of the reviewers who provided the listed reports. This is part of the Nature Communications initiative to facilitate training in peer review and to provide appropriate recognition for Early Career Researchers who co-review manuscripts.

We thank this Reviewer for their detailed and constructive comments.

****

Reviewer #4 (Remarks to the Author):

In this work, the authors have reported syntheses and excited-state dynamics of luminescent radicals with long-lived quartet states and proposed a strategy for compounds with microwave-addressable high-spin states. The clarification of the photo-excited states, including charge-transfer states, was carried out through their prompt and delayed photoluminescence, transient absorption measurements, transient ESR, and pulse ESR with the help of computational work. The authors have revealed the role of molecular topology in luminescent quantum materials, which leads to the coulombic control of the charge transfer states. I consider that this work is very interesting and contains new, important findings. However, there

are some points that should be addressed. Therefore, I recommend publication after the revisions. Please consider the following points.

We thank the Reviewer for their helpful comments, and are glad they find our work novel.

1) Comparing the photophysical data between solution and PMMA films is important, as well as the solvent polarity dependence. Please add the photophysical data in PMMA in Table 1 if it is possible.

We agree that comparing the solution and film data is crucial for gaining an understanding of the mechanism. We therefore provide more detailed photophysical data of both toluene solutions and dilute PMMA films in Tables S3 and S4. Emission yields for most compounds are larger in solution compared to films. This can be interpreted as a result of a larger population of $^{2}CT_{Ac}$ states in films, where torsional freedom is restricts the state interconversion. We refer to the film data in the main text:
"A smaller number of conformers can populate the $^{2,4}LE_{Ac}$ in films compared to solutions, as seen from the increased contribution of prompt emission in all compounds. (Table S4)"

Table S3. Extended photophysical data of the investigated molecules in toluene solutions.

Material	Φ_{PL}	Φ_1	τ_1 (ns)	kr_1 ($\times 10^6$ s^{-1})	k_{nr_1} ($\times 10^6$ s^{-1})	Φ_2	τ_2 (ns)	kr_2 ($\times 10^6$ s^{-1})	k_{nr_2} ($\times 10^6$ s^{-1})	τ_{eff} (ns)
An-T-1Cz	0.08	0.07	15.6	4.6	64.1	0.01	437	0	2.3	68
An-T-3PCz	0.03	0.03	15.8	1.8	63.3	0.005	729	0	1.4	140
T-3Cz-An	0.55	0.03	31.6	1	31.6	0.52	1030	0.5	1	980
T-3Cz-Acr	0.50	0.18	31.8	5.7	31.4	0.32	216	1.5	4.6	150

Table S4. Extended photophysical data of the investigated in 0.5% in PMMA films.

Material	Φ_{PL}	Φ_1	τ_1 (ns)	kr_1 ($\times 10^6$ s^{-1})	k_{nr_1} ($\times 10^6$ s^{-1})	Φ_2	τ_2 (ns)	kr_2 ($\times 10^6$ s^{-1})	k_{nr_2} ($\times 10^6$ s^{-1})	τ_{eff} (ns)
An-T-1Cz	0.01	0.01	10	0.5	100.0	0.0005	986	0.0	1.0	59
An-T-3PCz	0.06	0.05	9	0.6	111.1	0.01	221	0.0	4.5	44
T-3Cz-An	0.50	0.17	51	3.3	19.6	0.34	2640	0.1	0.4	1800
T-3Cz-Acr	0.58	0.24	43	5.6	23.3	0.35	3830	0.1	0.3	2300

2) Authors describe: "The effective lifetime extends to around 2.3μs for films of T-3Cz-An. In contrast, it shortens in films of An-T-3PCz, indicating the promotion of deactivation channels in a more rigid environment." I could not find the corresponding data in the supplemental information. In Table S2, solvent dependence is given only in Toluene and THF. Please describe more clearly the data in PMMA of T-3Cz-An and An-T-3PCz, like Table S2.

In addition to the PMMA table above, we now also include the complete dataset of fitted lifetimes for the temperature dependent film data. We have referenced the new Table S5 and expanded that sentence to say "The effective lifetime extends to around 1.8 μs and 2.3 μs for films of T-3Cz-An and T-3Cz-Acr, respectively."

Table S5. Fitted lifetimes and weights for temperature dependent trPL measurements of 5% in PMMA films.

	An-T-1Cz				An-T-3PCz				T-3Cz-An				T-3Cz-Acr			
Temp (K)	τ_1 (ns)	w_1 (%)	τ_2 (ns)	w_2 (%)	τ_1 (ns)	w_1 (%)	τ_2 (ns)	w_2 (%)	τ_1 (ns)	w_1 (%)	τ_2 (ns)	w_2 (%)	τ_1 (ns)	w_1 (%)	τ_2 (ns)	w_2 (%)
6	19	95	400	5	21	96	374	4	35	42	7320	58	33	48	9290	52
20	20	95	443	5	20	95	327	5	36	43	7240	57	33	48	8680	52
50	–	–	–	–	–	–	–	–	37	42	6850	58	34	47	8280	53
80	18	96	405	4	20	96	359	4	39	39	6720	61	34	45	8190	55
115	–	–	–	–	–	–	–	–	42	38	6110	62	37	43	7130	57
150	15	97	430	3	16	96	292	4	45	36	5640	64	38	42	6390	58
195	–	–	–	–	–	–	–	–	47	36	4780	64	40	42	5780	58
220	10	99	235	1	12	97	252	3	–	–	–	–	–	–	–	–
240	–	–	–	–	–	–	–	–	49	36	3760	64	43	41	5390	59
292	10	99	986	1	9	98	221	2	51	33	2640	67	43	41	3830	59

3) The definite assignment of the transient absorption spectra is crucial in the later discussions. Please denote more definitely the spectral assignment in Figure 2. Is the peak around 500 nm in Figure 2(a) assigned to $^2CT_{Cz}$ or the peak around 640 nm to $^2CT_{Cz}$? Both CT states ($^2CT_{Cz}$ and $^2CT_{Ac}$) can be stabilised in the polar solvent. Why is the transient absorption band attributed to $^2CT_{Cz}$ missing in MeCN? Authors describe that "the growing PIA at 430 nm is due to the anthracene triplet, arising from both $^2LE_{Ac}$ and $^4LE_{Ac}$ populations." The authors made this assignment based on the comparison with the time profile of the GSB. This assignment is true because this assignment is also confirmed because of a similar spectrum was observed in the simpler anthracene–radical-linked systems (see Chem. Lett. 2016, 45, 1324). Please cite the corresponding literature. The expression "This band is convolved with the GSB to varying extent" is slightly unclear. Does this mean that, as a signal attributed to 2,4LEAc decays, the signal attributed to GSB also decays in the same way? If so, please describe this more clearly. Also, please add a figure showing the correlation between these two transition intensities to the ESI.

We provide a detailed explanation of our revised TA analysis in the answer to Referee 1 Comment 4. In particular, the assignment of features in MeCN is explained with relation to new Fig. S39. We agree with the reviewer that our discussion of the GSB feature was unclear. We rephrased the line in question to: "A growing PIA at 430 nm is attributed to the anthracene triplet, arising from both $^2LE_{Ac}$ and $^4LE_{Ac}$ populations.(Chem. Lett. 2016, 45, 1324) For An-T-1Cz and An-T-3PCz, this band is convolved with the GSB." The kinetic analysis of all TA datasets is included as Figures S31 and S37.

4) Authors carried out the spectral simulation of the trESR using EasySpin and the populations are assigned to Q+2/3, Q+1/2, Q-1/2, Q-3/2, D+1/2, and D-1/2, respectively. The expression of "Q+2/3, Q+1/2, Q-1/2, Q-3/2, D+1/2, and D-1/2" mean usually high-field spin sublevels. However, the intersystem crossing of the spin-orbit interaction normally leads to the selective population to the zero-field spin sublevels (see J. Chem. Phys. 1973, 58, 985 for Triplet-state, J. Am. Chem. Soc. 1998, 120, 3152 for quartet of ZnTPP-p-nipy, and J. Am. Chem. Soc. 2000, 122, 984 for quartet of Anthracene-radical linked system). For the selective population to the high-field spin sublevels, the spectral pattern of the quartet state is different to that of the zero-field

spin sublevel's population (Angew. Chem. Int. Ed. 2006, 45, 4666 and J. Mater. Chem., 2008, 18, 381). As also shown in Fig. 7 of J. Magn. Reson. 2023, 349, 107410 (the paper of EasySpin), the dominant population to Q+1/2 and Q-1/2 gave a spectral pattern of the high-field spin-sublevels population, which is different to Figure 3(a). Figure 3(a) is a typical tr-ESR pattern for the selective population to the Q'±1/2 zero-field spin-sublevel (see J. Am. Chem. Soc. 2001, 123, 294). In addition, the spectral pattern of Figure 3(a) is opposite microwave absorption/emission to the triplet-state of anthracene itself. This is a typical pattern obtained by the radical-spin-orbit borrowing enhanced intersystem crossing (see also J. Am. Chem. Soc. 2001, 123, 294). Please check again the assignment of the population written in Table 2 and please cite the above reference because the tr-ESR pattern is quite like Figure 3(a).

We agree that our account of the spin polarisation was deficient, and we appreciate the Reviewer's detailed comment. We have incorporated this explanation into our revised text:
"The lineshapes of the prompt (averaged 0.5-1.5 μs after pump) full-field spectra are typical of an acene covalently tethered to a radical, within the strong triplet-radical exchange coupling regime.(Teki 2001) The polarisation patterns extracted from simulations (Fig. S40) reveal that the m_s = ±1/2 zero-field sublevels are favoured during ISC,(Ishii 1999) due to the dominant z-component of spin orbit coupling (SOC) within the anthracene triplet (Table S8).(Tait 2019) This suggests that the radical-enhanced spin-orbit borrowing is active regardless of strength of radical-acene electronic coupling."

5) The simulation spectrum shown in Figure 3(b) does not reproduce the observed spectrum especially the XY peak intensity. Please check the population as well as the assignment (high-field population is true?) written in Table 2.

We describe our revised EPR simulation method as in response to Reviewer 1 Comment 5 above. The XY intensity fit is improved by adding a contribution from the thermalised (i.e. Boltzmann populated) quartet state with the same spin Hamiltonian.

6) Based on the conclusion of this paper, the Cz moiety is the source of luminescence. Therefore, it does not appear necessary for the radical moiety to be luminescent. It appears that as long as the energy relationship is satisfied, there should be no problem. If that is the case, it would be better to mention this point in the conclusion.

It is true the quartet-mediated delayed luminescence can be observed even in systems based on non-luminescent radicals, as was recently reported in TADF-TEMPO systems (Gorgon et al., *Adv. Mater.* 2025, 2501164). We have amended this statement near the end of the discussion to reflect it: "The results presented here, together with the recently reported Radical Thermally Activated Delayed Fluorescence (TADF) molecules, show that quartet-derived emission can be achieved provided that any bright state is in energetic proximity to the high spin state."

7) Although luminescent radicals without Cz moiety have been reported with very high quantum yields of luminescence, the differences between these systems and the present systems should be clearly mentioned.

We have revised the second paragraph of the introduction to expand on how our new compounds fit into the broader area of radical emitter research. We include three new and

recent references of important doublet emitters without Cz-type units (*Org. Lett.* 2025, 27, 23, 6019–6024; *Angew. Chem. Int. Ed.* 2022, 61, 25, e202201965; *Angew. Chem. Int. Ed.* 2025, 64, 6, e202418762). The new text is:
Luminescent organic radicals are a rapidly developing class of materials with a doublet emission channel offering unique spin-optical opportunities.[14] While many molecular design strategies are being pursued,[15–18] donor-substituted tris(2,4,6-trichlorophenyl)-methyl (TTM) radicals are currently the most performant platform, and have been utilized in organic light emitting diodes with record efficiencies.[19-21] Their high emission quantum yields and stability have been attributed to their donor-to-TTM charge transfer (CT) lowest excited state.[22]

8) The reason why the luminescence quantum yield of T-3Cz-Acr is slightly lower than that of T-3Cz-An has been discussed based on the energy location of $^2CT_{AC}$. Can this phenomenon not be simply explained by the fact that the non-radiative decay efficiency of Acr itself is higher than that of An?

As seen in Tables S3-4, the non-radiative decay rates in T-3Cz-Acr are indeed slightly higher than in T-3Cz-An. We removed the statement in question from the end of the Quantum Chemical Calculations section.

9) Line 101 in page 7: Authors describe that "$^2CT_{Ac}$ becomes the lowest excited state upon stabilization in all compounds". However, the results of the MO calculation are presented only for TOL and MeTHF. Please confirm the results of "2CTAc becomes the lowest excited state upon stabilisation in all compounds" by the MO calculations.

We revised this sentence to more precisely refer to the experimental observations to read: "…showing that the energetic ordering of the states is inverted in a high-polarity medium, becoming the same as in compounds with strong electronic coupling."

The following is a list of minor points.

10) Line 40 in page 3: Authors describe "We identify four excited states required for their operation (Fig. 1d):" In Fig. 1, 1d is missing. Does it refer to Fig. 1c?

We apologise for the confusion caused by this typo. It does refer to panel c. We have updated the line.

11) Line 42 in page 3: Does the latter refer to $^2CT_{Ac}$? Please identify more clearly.

We changed that sentence to explicitly mention the $^2CT_{Ac}$ state.

12) Line 27 in page 3: About the citation in "Critically, such compounds have thus far been non-luminescent,[8,9] limiting their potential.", please add "Chem-A Eur. J. 2020, 26, 980" because there is describes a comparison between the non-luminescent and luminescent π-radicals.

We agree that this is a helpful reference here.

13) $^2(D_1S_0)$ and $^4(D_0T_1)$ are better than $^2D_1S_0$ and $^4D_0T_1$.

We have made this change in Fig. 1c. To improve clarity, we have unified all state labels to the $^2CT_{Cz}$, $^4LE_{Ac}$ notation throughout the paper.

14) Line 202 in page 13: “Due to the contribution of the TTM SOMO to the delocalised π-system,.... Please cite appropriate references.

We have added references to a primary result (J. Am. Chem. Soc. 1958, 80, 9, 2057–2059) and a relevant review (Chem. Eur. J. 2020, 26, 980).

ANSWERS TO REVIEWER COMMENTS

We thank all Reviewers for their constructive feedback. In this document, the comments are in black, our responses are in blue, and changes to the main text are in green.

Reviewer #1:

The authors have satisfactorily addressed the reviewer comments which has resulted in a significantly improved manuscript. The added discussion on electronic couplings and state energetics makes the work much more impactful. However, there are still a few points that should be addressed before publication:

We are grateful for this positive assessment of our changes.

1.) The new PL data for 0.5% doped PMMA films is a nice addition, and it's encouraging that emission is possibly observed from CT(Ac). However, the new 0.5% data in Figure S23 (presumably recorded at room temperature) displays an emission profile centered at 750 nm for An-T-1Cz at 30 microseconds. Meanwhile, the corresponding data in Figure S24 for the 5% sample displays an emission spectrum centered at ~650 nm from 10-50 microseconds. The authors should clarify why this discrepancy exists and if it is a concentration effect.

We attribute the difference in long-time emission profile for 0.5% and 5% films of An-T-1Cz primarily to an effect of film morphology. The higher loading for temperature-dependent trPL measurements was chosen to ensure sufficient signal intensity given the light path in our cryostat, and some contribution to the very slow emission arising from aggregated domains cannot be excluded at this loading.

However, we remain cautious in interpreting the μs emission features in films of the weakly emissive materials. Less than 0.01% of photogenerated excitons emit after 10 μs in films of An-T-1Cz, making late spectral shifts particularly sensitive to film morphology. Accordingly, the more dilute data in Fig. S23 are given as an observation of possible weak emission from the $^{2}CT_{An}$ state. This assignment is made in conjunction with other spectroscopic evidence, and is used solely to indicate the relative energetic position of this state with respect to other excited states.

We have updated the SI figure captions with the missing room temperature designations.

2.) The updated state labels should be included with Fig. 2b,e so that readers are not confused by the assignments. My understanding is that in Fig. 2b, it can be labelled that both CT(Ac) and CT(Cz) are still present. In Fig. 2e, the feature can be labelled as just CT(Cz).

We agree that this would improve clarity, and have relabelled Figure 2 to show this assignment.

3.) Adding the TA kinetic fits to the SI is a huge improvement to the manuscript. However, a discussion of the fit results in the main text would be beneficial, rather than a non-specific reference to Figures S30-32 on Line 101. Direct references to data from the SI in the discussion would also be beneficial. For example, on Line 108: "T-An, An-T-1Cz and An-T-3PCz, display

similar decay kinetics, with sub-50 ps decay lifetimes." A reference to the newly added Table S6 is needed here so readers know where to look for this data.

We are pleased that the addition of TA fits is seen as an improvement, and agree the analysis can benefit from them further. We have expanded the discussion of the kinetics throughout the main text. For the line above, this becomes:
"T-An, An-T-1Cz and An-T-3PCz, display similar decay kinetics, with sub-50 ps decay lifetimes (Tab. S6). For T-An, the dynamics follow a biexponential decay, with a prompt component of 3.5–5.5 ps and a delayed component of approximately 34 ps. An-T-1Cz and An-T-3PCz exhibit comparable kinetics across all bands, with the exception of PIAs associated with the $^{2}CT_{Cz}$ state, where an additional sub-picosecond component is observed. We assign this ultrafast process to internal conversion from $^{2}CT_{Cz}$ to $^{2}CT_{Ac}$."

4.) More generally, all figures in the SI should be referenced in the main text unless they are part of some discussion in the SI, which is then referenced in the main text.

We now refer to all SI items within the main text.

5.) While the single-wavelength kinetic analysis is a nice addition, it is not clear why the SVD analysis is also being included if the authors believe it to have limited utility. We suggest removing it unless the authors believe it to be necessary.

We agree that the SVD is better left out, and have removed it from the SI.

6.) While the trESR now includes fits, the method and parameters which went into those fits still seem arbitrary.

- Why would there be a Boltzmann population present in a transient experiment? Does this suggest that T1 is on the order of 500 ns, which means that the ratio of polarized to Boltzmann spectra should change as a function of time over the course of the experiment. Further, why does it then change so drastically between samples?
- The polarized portion of simulations include population of the trip-doublet and trip-quartet manifolds
- How come the dipolar coupling is negative in An-T-1Cz and An-T-3PCz and why is there such a large rhombic component in all four?
- In table S8, how are the quartet populations and the triplet populations related? It is not apparent from the code snippet. Are you following a method similar to what is described in Appl. Magn. Reson. 2011, 40, 189 and more recently J. Am. Chem. Soc. 2025, 147, 38762?
- Both of those papers, Appl. Magn. Reson. 2011, 40, 189 and J. Am. Chem. Soc. 2025, 147, 38762, relating to quartets should be referenced.

The presence of a Boltzmann population has often been observed in quartet systems, e.g. Kandrashkin et al. (J. Phys. Chem. A 2006, 110, 31, 9607) or Nolden et al. (Chem. Eur. J. 2021, 27, 2683). The spectra of T-3Cz-An become more symmetric at later times (Fig. 3f), which may be due to faster non-radiative decay of those quartet states which yield the Boltzmann polarisation. We thank the Reviewer for drawing our attention to the discrepancies in the polarised to Boltzmann signal ratio, and apologise for a normalisation mistake that was present in obtaining it from our simulations. The corrected values are between 3 and 6 for all materials.

The values for the triplet-radical dipolar coupling and rhombicity are consistent with the molecular structures of the two sets of compounds. The sign of the D_{RT} is determined by the projection of the radical-triplet interspin vector onto the principal axes of the anthracene triplet ZFS tensor, and can be approximated to vary as $D \propto \frac{1}{r^3}(1 - 3cos^2\theta)$. In An-T-1Cz and An-T-3PCz, the radical and anthracene are axially aligned, so $\theta \rightarrow 0$. The rhombicity arises from the twisted conformations adopted by the compounds due to steric hindrance.

We confirm that we use the method described in the suggested literature to relate the populations, and have added the citations. The relationship between populations is detailed below in response to Reviewer 4 Comment 3.

7.) In J. Am. Chem. Soc. 2025, 147, 38762, which is another example of a TTM radical-chromophore dyad, it was shown that with the proper energetics, the CT state mediates quartet formation. This should probably be discussed as in this work the CT state was suggested to act as a dark trap, is that a result of an energetics mismatch?

We agree with the Reviewer that the role of charge-transfer states in mediating quartet formation depends on their energetic alignment. In our initial work (Nature 2023, 620, 538), we proposed that the $^2CT_{Ac}$ state can mediate quartet formation by enabling spin-flip processes and provided computational results at a high level of theory which support this mechanism. Experimentally confirming their role was a central motivation of the present study.

The interesting system reported by Chen et al. (JACS 2025, 147, 38762) explores a different energetic regime. In TTM-NDI, the CT state is located 0.3 eV below the quartet state and 0.5 eV below the photogenerated radical doublet state at room temperature, but is brought higher in energy in frozen solution (85 K). The key consequence of these energetics is the order and direction of the excited state processes. In TTM-NDI, formation of the quartet is irreversible (because of the large D_1-T_1 gap) and the $^2CT_{TTM\text{-}NDI}$ state is generated first. In our systems, which feature D_1-T_1 energetic resonance, the $^2LE_{Ac}$ is generated first. The different quartet generation pathways are visible in the spin polarisation pattern in ESR, and the observation of quartet-recycled delayed luminescence in our systems. Taken together, it emerges that while ^2CT states can mediate spin-flip processes, their effectiveness critically depends on the energetics.

We have expanded the Discussion section to better reflect the prior art on energetic requirements for CT-mediated quartet formation.
"Direct conversion between $^2LE_{Ac}$ and $^4LE_{Ac}$ states is spin-forbidden to first order, but can be mediated by a state with a different spatial wavefunction. (El-Sayed 1968) It has been previously demonstrated that a doublet charge transfer state between the dyad components can fulfil this role provided it is energetically accessible.(Gorgon 2023, Teki 2025, Chen 2025) This condition is satisfied in T-3Cz-An, consistent with the high efficiency of delayed emission and the early-time signals in transient ESR measurements. However, as observed for An-T-3PCz and An-T-1Cz, when the $^2CT_{Ac}$ state lies below both the quartet and the emissive $^2CT_{Cz}$ states, non-radiative decay outcompetes ISC, suppressing quartet formation."

8.) Lines 87-88, a reference is made to PMMA data in Figure S21, but the figure caption says the data was recorded in toluene.

We have corrected the text to refer to the toluene data as intended. We have updated the order of SI figures to maintain sequential numbering in the revised text; this is now Fig. S17.

9.) Figure S21: the plot in the top right is labelled as An-T-3Cz, but it should be An-T-3PCz. This seems to be a reoccurring error so double-check that all compound labels are correct.

We have corrected this, and checked all labels in the manuscript.

10.) Line 179: there is a reference to Fig. 1d, but this panel does not exist. The reference should be to Fig. 1c.

We apologise for the typo, and have corrected the reference.

Reviewer #2:

The authors have addressed all my questions well. I recommend it can be accepted without any change.

We thank this Reviewer for recommending acceptance of our work.

Reviewer #3:

I co-reviewed this manuscript with one of the reviewers who provided the listed reports. This is part of the Nature Communications initiative to facilitate training in peer review and to provide appropriate recognition for Early Career Researchers who co-review manuscripts.

We are grateful for the helpful comments in both revision rounds.

Reviewer #4:

The authors have appropriately revised the manuscript for many of the issues that I pointed out. However, the revised manuscript still contains several ambiguities and unresolved questions (not sufficiently addressed). The issues are listed below. Therefore, we recommend acceptance after the clarification of the following points.

We thank the Reviewer for their advice, and supporting acceptance following clarifying their final points.

(1) According to my remark, authors added more detailed photophysical data of both toluene solutions and dilute PMMA films in Tables S3 and S4. This data makes a comparison between the solution and film data is crucial for gaining an understanding of the mechanism. Based on this comparison, authors mention as follows in the main text.
"A smaller number of conformers can populate the ${}^{2,4}LE_{Ac}$ in films compared to solutions, as seen from the increased contribution of prompt emission in all compounds (Table S4)." What does "conformer" mean here? Furthermore, judging from the data (PL efficiency) shown in Tables S3 and S4, the increased contribution of prompt radiation by using PMMA is observed only in An-T-3PCz and T-3Cz-Acr, not in all compounds. The description in the above sentence

is ambiguous and difficult to understand, and it appears to be inconsistent with the experimental results (comparison of Tables S3 and S4).

We thank the Reviewer for carefully comparing the data presented in Tables S3 and S4. By conformers, we refer to distinct molecular geometries that differ predominantly in the torsional angles between the main molecular units (trityl, acene, carbazole). These torsional degrees of freedom strongly influence the relative energies of the $^{2}CT_{CZ}$, $^{2}CT_{Ac}$ and $^{2,4}LE_{Ac}$ states. The differences in emission efficiency between solution and film are governed by the interplay of two competing effects: (i) suppression of non-radiative decay in a rigid medium, and (ii) changes in the relative rates of energy transfer between thermally accessible states. The former effect is known to enhance emission in radical systems in rigid media (Liu et al. Angew. Chem. 2020, 59, 23030), while the latter is specific to systems with thermally accessible dark states. Although a higher percentage of all photons is emitted from the prompt channel in films compared to solutions, the total PLQE changes vary across the materials, making the comparison difficult. Our original statement was ambiguous, so we have removed it from the manuscript.

(2) While it may be unavoidable that complexity arises because multiple distinct states can overlap to form candidate TA spectra, the assignment of TA spectra remains complex and inherently ambiguous. In the radical spin-orbit borrowing mechanism proposed for the anthracene-radical linked systems showed the quartet Tr-ESR spectra resembles Figure 3, a pathway via the CT state constructed from anthracene anion radical is proposed as one of the most possible candidates (JACS 2001, 123, 294. and JACS 2025, 147, 43358). In addition, the polarization transfer to the ground state like that described in this paper has also been observed in the latter paper (although the DEP direction in the central peak is opposite, it may come from the sign of the exchange coupling in the CT state).
Is not the possibility of anthracene anion radical, although the authors assign the band near 700 nm to anthracene radical cation? Does not exist the CT state with the anthracene anion radical nearly above the lowest quartet state based on the MO calculations?

We agree with the Reviewer that owing to the presence of several energetically proximate states, the assignment of TA spectra is complex. To improve clarity, we have relabelled Fig. 2 as recommended by Reviewer 1.

We have carefully considered the possibility that an anthracene anion–based CT state could act as an intermediary in the ISC, as proposed in JACS 2001, 123, 294 and JACS 2025, 147, 43358. The TA spectra in the present work and previous study (Extended Data Fig. 1, Nature 2023, 620, 538) match anthracene cations (absorption near 690–700 nm) and trityl anions (around 600 nm, as seen recently in Nature Materials 2026, 25, 100). We do not observe features characteristic of anthracene anion radicals. Furthermore, calculations at various levels of theory identify the state involving charge transfer from anthracene to trityl as the energetically lowest configuration. We acknowledge that we cannot categorically exclude contributions from other CT intermediates, including anthracene anion–based configurations. Nevertheless, we emphasise that the central conclusion of this work lies in demonstrating how molecular topology dictates radiative, non-radiative, and spin-flip pathways that ultimately control the efficiency of quartet-derived emission. A more definitive study of all CT intermediates is an intriguing direction for future investigation.

(3) In the time-resolved ESR simulations, the DEP in the zero-field ground state can be clearly understood, but the DEP in the uncoupled basis is unclear. The DEP in the spin sublevels under external magnetic field should depend on the direction of the external field relative to the principal axes of the fine structure tensor. Since the DEP of the T0 sublevel in uncoupled basis equals that of Tz sublevel in the zero-field basis, the direction of the external magnetic field for the DEP in the uncoupled basis seems to be parallel to the z principal axis of the fine-structure tenses. Are these values for DEP in the magnetic field direction being cited as a representative example? This point is unclear. However, since the authors are not using a self-developed simulation program but rather the publicly available EasySpin program and this issue is not main subject of this paper, I will not require further detailed explanation for this point. However, it seems strange that the observed spectrum of An-T-3PCz shows aa/ee pattern (a/e : absorption/emission of microwave) but Ppol/PBoltz = 0.2 is used in the simulation. If Boltzmann population is dominant (80%), the spectrum should be fully absorption pattern. Please check the ratio again.

We thank the Reviewer for pointing out the ambiguity in our populations. It is true that the polarisation of the high-field triplet sublevels in the uncoupled basis depends on the orientation of the external magnetic field relative to the molecular frame. The values listed in Table S8 for the uncoupled basis are cited for the canonical orientation where the external magnetic field is parallel to the principal z-axis of the zero-field splitting tensor. We selected this orientation to illustrate the selectivity of ISC into the T_z state. We have amended Table S8 to explicitly state that the uncoupled basis values refer to the $B_0 \parallel z$ case. We note that the ESR spectra are simulated with orientation-dependent DEP for all angles during the powder integration, so are unaffected by this change.

We apologise for a normalisation mistake that was present in extracting the polarisation ratio from the simulations shown in Fig. S41. The corrected ratios are now included in Table S8, with all materials showing Ppol > PBoltz, as clearly visible in the experiments.

(4) Authors describe “This suggests that the radical-enhanced spin-orbit borrowing is active regardless of strength of radical-acene electronic coupling”.
The efficiency of the radical-enhanced spin-orbit borrowing mechanism is not proportional directly to the radical-acene electronic coupling. The efficiency depends on the electron transfer rate to the CT state and the magnitude of SOC on the radical site (see: JACS 2025, 147, 43358. In this paper, the spin-coupler dependence of J and DEP generation is described). Please revise the above sentence appropriately.

We have rephrased this sentence and included the reference:
“This suggests that the dominant quartet formation pathway in these four materials is radical-enhanced spin-orbit borrowing.(Teki et al. JACS 2025)”

ANSWERS TO REVIEWER COMMENTS

We thank all Reviewers for their feedback. In this document, the comments are in black, our responses are in blue, and changes to the main text are in green.

Reviewer #1:

The authors have satisfactorily addressed most our previous comments aside from the EPR. Perhaps you have misinterpreted J. Phys. Chem. A 2006, 110, 31, 9607, the Boltzmann contribution to the spectrum arises at long times and is a result of spin-lattice relaxation. Your TCW spectra are recorded at fairly early times in the kinetics and should have minimal contributions from T1 relaxation, not 20-30% of the observed spectrum. The polarization kinetics from figure S39 show that relaxation (and delayed emission) doesn't influence the spectrum for many microseconds. So, something still doesn't quite add up with respect to how the simulations were performed.
From the provided Matlab code snippet, the rotation matrices, States, provided from cgmatrix represent an uncoupled basis to coupled basis transformation. The transposes are incorrect when using the state matrices to transform from coupled to uncoupled basis.
With respect to the dipolar coupling, given that they are rooted in the structure, which is not mentioned in the text, why did you choose to present them in that form rather than including the tensor in its diagonal basis and the appropriate euler angles?

We are grateful for the positive assessment of the bulk of our previous changes. Regarding the EPR, we appreciate the Reviewer noticing an error in the linear algebra. We agree that the contribution of a Boltzmann-polarised quartet is not justified, and have removed it from the simulations. We now use a minimal model which yields good fits to experiments. As suggested, we link the radical-triplet dipolar coupling to the chemical structures. The Methods text reads:
Transient cw ESR spectra were simulated using EasySpin version 6.0.11, with the following Hamiltonian:

$$\hat{H} = \hat{H}_{\text{Zeeman}} + \hat{H}_{\text{ex}} + \hat{H}_{\text{ZFS},T} + \hat{H}_{\text{dip},RT}$$

$$\hat{H}_{\text{Zeeman}} = \mu_B \mathbf{B}_0 \cdot (g_T \hat{\mathbf{S}}_T + g_R \hat{\mathbf{S}}_R)$$

$$\hat{H}_{\text{ex}} = J(\hat{\mathbf{S}}_T \cdot \hat{\mathbf{S}}_R)$$

$$\hat{H}_{\text{ZFS},T} = D_T\left(\hat{S}_{z,T}^2 - \frac{2}{3}\right) + E_T\left(\hat{S}_{x,T}^2 - \hat{S}_{y,T}^2\right)$$

$$\hat{H}_{\text{dip},RT} = \frac{\mu_0 g_T g_R \mu_B^2}{4\pi r_{RT}^3}\left[\hat{\mathbf{S}}_T \cdot \hat{\mathbf{S}}_R - 3(\hat{\mathbf{S}}_T \cdot \hat{\mathbf{n}})(\hat{\mathbf{S}}_R \cdot \hat{\mathbf{n}})\right]$$

$$\hat{\mathbf{n}} = \sin\theta_{RT}\cos\phi_{RT}\hat{\mathbf{x}} + \sin\theta_{RT}\sin\phi_{RT}\hat{\mathbf{y}} + \cos\theta_{RT}\hat{\mathbf{z}}$$

A fixed value of isotropic exchange was used to set the strong exchange regime as determined from nutation experiments. The radical-triplet dipolar interaction strength is fixed using the distance obtained from the TD-DFT calculations and the point dipole approximation [Chem Sci 2023, 14, 5361]. The density matrix was populated in the triplet zero-field basis by explicitly constructing the projection operators, as shown in Fig. S42. The spectra were fitted by optimising the triplet and radical populations, isotropic *g*-values, triplet dipolar constants, radical-triplet orientation angles and an additional weight W_R of a thermally polarised doublet component.

The revised treatment of dipolar coupling yields a new observation in the Main text:

We observe more axial symmetry and larger deviations from the acene triplet ZFS values in compounds with direct radical-acene coupling, suggesting that this linkage induces a saddle-shaped distortion of the anthracene.

The updated Figures and Table are reproduced below:

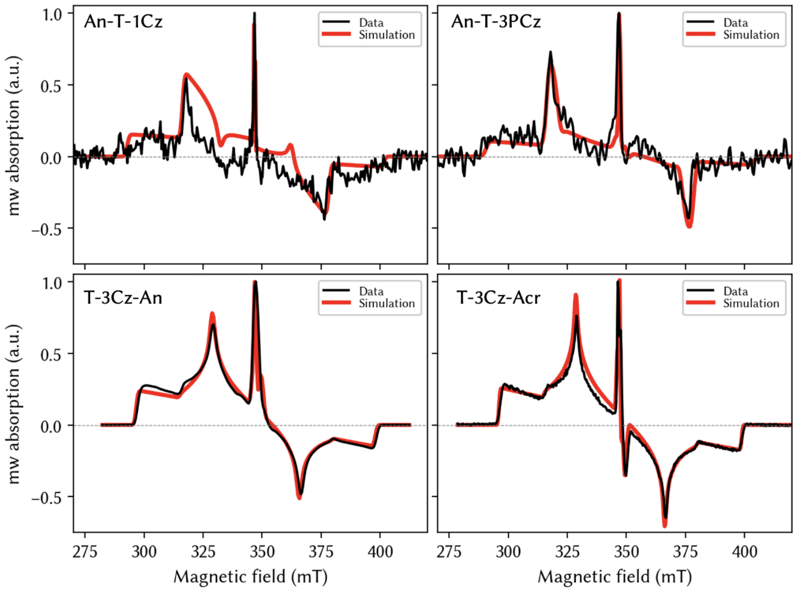

Figure S40: Simulations to the prompt (0.5-1.5 μs) X-band transient ESR spectra on 100 μM frozen toluene solutions at 80 K. The same data is presented in the main text Fig. 3a,b.

```
Sys.S = [1 1/2];
Sys.g = [g_T; g_R];
Sys.D = [D_T E_T; 0 0];
Sys.J = -1e5;
Sys.lwpp = 1.2;
D_RT = (mu0/(4*pi)) * (g_T * g_R * muB^2 * 1e-6) / (h*r_RT^3);
Sys.dip = [-D_RT/2, -D_RT/2, D_RT];
Sys.eeFrame = [phi_RT theta_RT 0] * pi/180;

Sx_T = sop(Sys, 'x1'); Sy_T = sop(Sys, 'y1'); Sz_T = sop(Sys, 'z1');
Sz_R = sop(Sys, 'z2'); Id = eye(size(Sx_T));
P_Tx = Id - Sx_T^2; P_Ty = Id - Sy_T^2; P_Tz = Id - Sz_T^2;
P_RA = 0.5*Id + Sz_R; P_RB  = 0.5*Id - Sz_R;
Rho_T = (w_Tx * P_Tx) + (w_Ty * P_Ty) + (w_Tz * P_Tz);
Rho_R = (w_RA * P_RA) + (w_RB * P_RB);
Sys.initState = Rho_T * Rho_R;
[~, specDyad] = pepper(Sys, Exp);

Sys2.S = 1/2; Sys2.g = g_R; Sys2.lwpp = 0.6;
[~, specR] = pepper(Sys2, Exp);
specFull = ((1 - abs(W_R)) * specDyad) + (W_R * specR);
specFull = specFull / max(specFull);
```

Figure S42: Transient ESR EasySpin code snippet.

Parameter	An-T-1Cz	An-T-3PCz	T-3Cz-An	T-3Cz-Acr
g_R, g_T	2.003, 2.005	2.003, 2.005	2.003, 2.005	2.003, 2.005
D_T, E_T (MHz)	2487, -100	2415, 0	2150, -203	2169, -201
r_{RT} (nm)	0.72	0.72	1.01	1.02
ϕ_{RT}, θ_{RT} (°)	91, 90	68, 61	35, 115	33, 114
W_R	0.40	0.55	0.55	0.31
D_β, D_α	0.522, 0.478	0.513, 0.487	0.529, 0.471	0.509, 0.491
T_z, T_y, T_x	0.37, 0.36, 0.27	0.37, 0.38, 0.25	0.41, 0.26, 0.33	0.39, 0.28, 0.33

Table S8: Spin Hamiltonian parameters and populations found from trESR simulations.

Couple minor points
1.) Figure S39 caption: 328 mT appears to be the maximum of the Y polarization, not the X polarization (see J. Magn. Reson. 2023, 349, 107410). Double check this assignment and correct it if needed.

We apologise for the incorrect label. We have corrected the assignment to Y.

2.) The title of the manuscript does not match the title written in the Supplementary Information.

Thank you for spotting this. We have corrected the title of the SI.

Reviewer #3:

I co-reviewed this manuscript with one of the reviewers who provided the listed reports. This is part of the Nature Communications initiative to facilitate training in peer review and to provide appropriate recognition for Early Career Researchers who co-review manuscripts.

We are grateful for the comments throughout the peer review process.

Reviewer #4:

The authors have adequately revised the manuscript for the issues that I pointed out. I have only one remark about author's opinion for the TA assignment as the anthracene cation. I think that the possibility via the CT state of the anthracene anion-radical species being involved cannot be completely ruled out as described below. For this reason, I believe it is preferable to leave a room for the assignment. However, I do not strongly object to the author's assignment of TA as a cation species. Therefore, the current revised manuscript is considered acceptable as it is, if authors want it, although there remains a room for the different assignment.
(1) According to the pairing property of the alternant hydrocarbons, the anion and cation of the anthracene make the spectra resemble each other (JACS 95, 3473 (1973)).
(2) According to the energy gap-law (Molecular Physics, 18, 145 (1970), the radiation-less transition (including CT) prefers in the energetically close levels. Therefore, the pathway via relatively higher energy CT state may be preferred.
RSO-EISC mechanism itself is expected to occur when the photoluminescence from the anthracene moiety is suppressed via the pathway to the CT state. Therefore, whether the CT state is anthracene cation or anion, this does not contradict the TrESR results. However, based on the above-mentioned points, unless the trityl cation spectrum can be definitively distinguished from that of the trityl anion and the possibility of a trityl anion can be completely ruled out, I think that the possibility via the CT state of an anthracene anion cannot be entirely excluded.

We thank the Reviewer for the supportive assessment, and agree that the TA spectra of TTM and anthracene ions appear at similar wavelengths, making conclusive assignment difficult. We have removed the mention of the cation on line 108.
